# Single-cell RNA sequencing of mid-to-late stage spider embryos: new insights into spider development

**DOI:** 10.1186/s12864-023-09898-x

**Published:** 2024-02-07

**Authors:** Brenda I. Medina-Jiménez, Graham E. Budd, Ralf Janssen

**Affiliations:** Department of Earth Sciences, Palaeobiology, Uppsala University, Villavägen 16, 75236 Uppsala, Sweden

**Keywords:** Single-cell sequencing, Spider development, Nervous system, Genetic fingerprint, *Parasteatoda tepidariorum*

## Abstract

**Background:**

The common house spider *Parasteatoda tepidariorum* represents an emerging new model organism of arthropod evolutionary and developmental (EvoDevo) studies. Recent technical advances have resulted in the first single-cell sequencing (SCS) data on this species allowing deeper insights to be gained into its *early* development, but mid-to-late stage embryos were not included in these pioneering studies.

**Results:**

Therefore, we performed SCS on mid-to-late stage embryos of *Parasteatoda* and characterized resulting cell clusters by means of *in-silico* analysis (comparison of key markers of each cluster with previously published information on these genes). *In-silico* prediction of the nature of each cluster was then tested/verified by means of additional *in-situ* hybridization experiments with additional markers of each cluster.

**Conclusions:**

Our data show that SCS data reliably group cells with similar genetic fingerprints into more or less distinct clusters, and thus allows identification of developing cell types on a broader level, such as the distinction of ectodermal, mesodermal and endodermal cell lineages, as well as the identification of distinct developing tissues such as subtypes of nervous tissue cells, the developing heart, or the ventral sulcus (VS). In comparison with recent other SCS studies on the same species, our data represent later developmental stages, and thus provide insights into different stages of developing cell types and tissues such as differentiating neurons and the VS that are only present at these later stages.

**Supplementary Information:**

The online version contains supplementary material available at 10.1186/s12864-023-09898-x.

## Introduction

Arthropod EvoDevo studies, i.e. the investigation of the development and the evolution of arthropods, still relies mainly on data from a single model organism, the vinegar fly *Drosophila melanogaster* (reviewed in e.g. [15]. *Drosophila* development is in many aspects derived however, and the processes and underlying genetic networks that lead to the development from the fertilized egg to the imago (the adult form) cannot easily be compared with the development of other arthropods (e.g. [38]. The introduction of new model organisms, and especially the gaining of deeper insights into existing model organisms that represent less derived modes of development is thus of the greatest interest for arthropod EvoDevo research.

The common house spider *Parasteatoda tepidariorum* (formerly known as *Achaearanea tepidariorum*) represents an emerging new model organisms that is often used for comparative developmental studies and the study of arthropod evolution in general (reviewed in [70, 139]. In the last two decades, many standard and advanced molecular biological methods have been established for this species, leading to a large number of comparative studies. Most of these studies, however, have relied on classic candidate gene approaches in which genes that are known to play a certain role during *Drosophila* development (or other established model organisms) are investigated in non-model organisms or emerging model organisms including *Parasteatoda* (e.g. [87, 138, 162, 163].

In order to investigate genes (and thus gene regulatory networks) beyond the classic candidate gene approach, genomics and embryonic and tissue-specific transcriptomics have been applied in the recent past, leading to the investigation of complete gene families, orphan genes, fast evolving genes and tissue-specific genes (e.g. [20, 77, 89, 142, 155]. A relatively recent new technical innovation now allows investigation of the transcriptome of every cell of a given organism or a developmental stage of a given organism separately. This so-called single-cell sequencing (SCS) technology can thus give much more detailed insights into gene transcription leading to the identification of genetic fingerprints that are specific for a certain cell type, or the elucidation of trajectories (consecutive developing stages) of differentiating cells (e.g. reviewed in [59, 114].

**Fig. 1:**
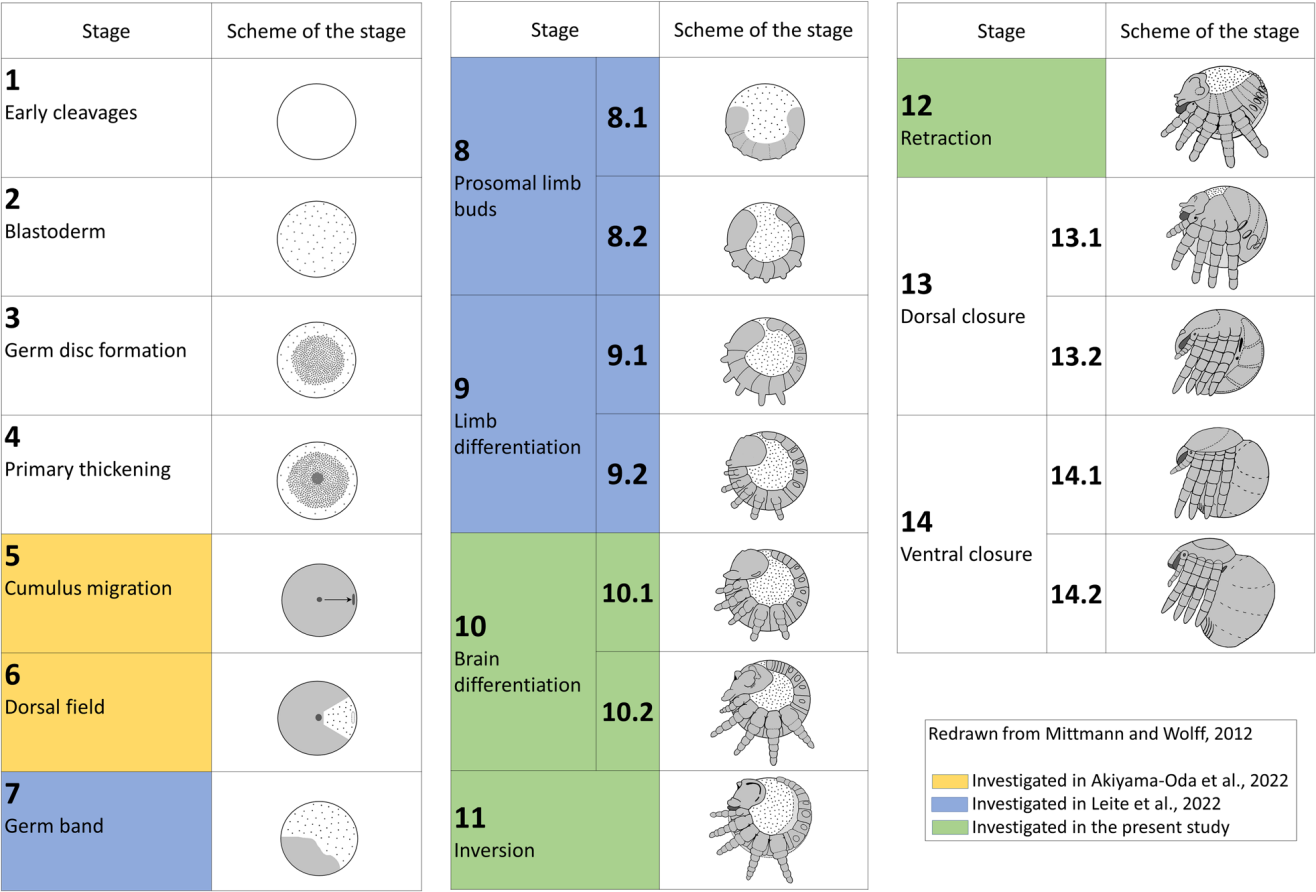
Stages of spider development. Redrawn after Mittmann and Wolff [130]. The 14 previously described developmental stages of the spider *Parasteatoda tepidariorum* are shown. Stages covered by SCS-analysis of Akiyama-Oda et al. [2] is highlighted in orange. Stages covered by Leite et al. [111] are highlighted in blue, and the stages addressed in our study are highlighted in green

Two very recent studies applying SCS on embryos of *Parasteatoda* covered the early stages of spider development. The first paper [2] investigated the so-called germ-disc stage of spider development (stage 5, staging after Mittmann and Wolff [130] (Fig. 1). Early during development, a spherical disc forms that in a subsequent step transforms into a bilaterally-symmetrical germ band (e.g. [130]. The second paper [111] investigated the earliest stages of germ band development (stages 7–9). These stages include the posterior addition of segments, the onset of nervous system development and the beginning of limb bud formation (Fig. 1).

**Fig. 2:**
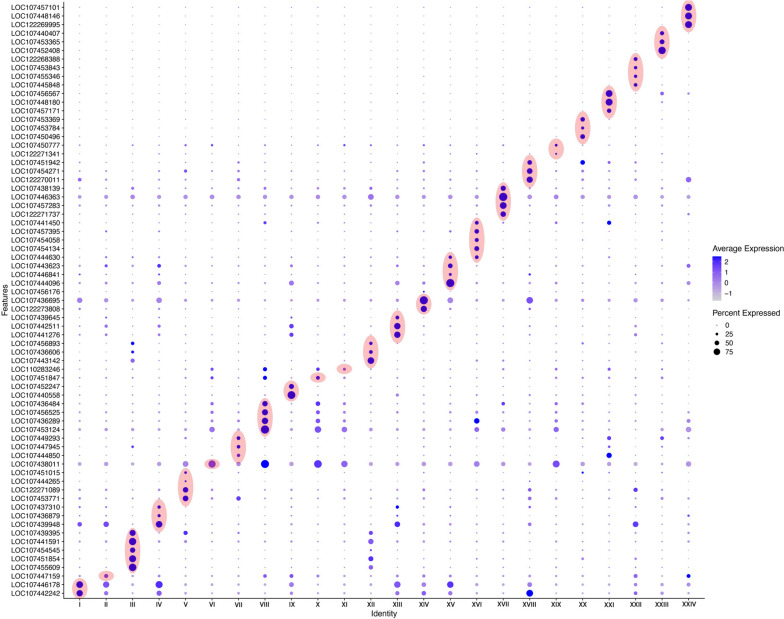
Dotplot showing all genes (Y axis) per cluster (X axis) for which whole mount *in situ* hybridisation was conducted in this study

In our study, we investigated developmental stages 10–12. These stages are characterized by for example the end of segment addition, the outgrowth and development of the appendages, nervous system differentiation, heart formation, dorsal closure and ventral splitting of the germ band (Fig. 1). In summary, developmental stages 10–12 likely represent a higher degree of cell-type specification and organogenesis compared with the previous studies on earlier developmental stages. The previous work on the earlier stages of development and our data complement each other and thus each contribute to a larger project driven by the international spider research community to cover all developmental stages of spider development by means of SCS [2, 111]. Our data contribute to this goal by delivering new insights into the differentiation of the spider’s central nervous system (CNS), the patterning of the appendages (including the highly-derived opisthosomal (posterior) appendages), morphogenic movements of cells, and organogenesis. During the course of our investigation, we first characterized the 24 identified cell clusters by means of *in-silico* analysis (literature analysis) using previously known information on marker genes of each cluster from other arthropods and, whenever available, also spiders including *Parasteatoda*. In order to test and verify the *in-silico* based predictions, we then investigated the embryonic expression patterns of 68 genes representing prominent markers of each cell cluster that (for the most) have not been studied previously in *Parasteatoda *(Fig. 2).

## Methods

Embryonic tissue dissociation, cell capture, cDNA library preparation, and single-cell RNA sequencing were performed at the Department of Developmental Biology and Gene Core facilities of the European Molecular Biology Laboratory (EMBL), Heidelberg, Germany.

### Embryonic tissue dissociation, cell capture, cDNA library preparation, and single-cell mRNA sequencing

In a watch glass, stage 10–12 embryos of *Parasteatoda tepidariorum* were submerged in phosphate buffered saline (PBS), and the vitelline membranes were removed using tweezers. As much yolk as possible was removed manually with tweezers and a pipette to prevent it from clogging the device used for later cell capture. The tissue-suspension was transferred into 1 ml of PBS and collected in a 1.5 ml low protein binding tube. To reduce the number of fat-droplets, the tissue-suspension was shaken on a shaking-platform at 1100 rpm for 5 min, then 100 µl of surface liquid collecting the fat-droplets was discarded and replaced with fresh PBS. To get rid of any remaining fat-droplets, the suspension was centrifuged at 800 rpm for 4 min, the supernatant was discarded, and the pellet was re-suspended in PBS. The latter centrifugation step was repeated one more time.

Cells were disaggregated (cell dissociation) by intermittent pipetting and swirling of the remaining embryonic tissue in an enzymatic mixture of 2% pronase and 2% sodium thioglycolate in PBS. The dissociation progress was constantly monitored under a microscope. When cell aggregates were no longer visible, the suspension was filtered using a 40 µm cell strainer to remove remaining tissue clumps, yolk granules and debris (e.g. strings of leaked DNA). This cell suspension was then centrifuged at 1100 rpm for 5 min to collect intact cells. The supernatant containing leaked RNA molecules and remaining small pieces of debris was removed. Finally, the cell suspension was filtered through a 20 µm cell strainer twice, and collected into a new 1.5 ml low protein binding tube. Cells were counted using a hemocytometer and cell viability was assayed using Fluorescein Diacetate and Propidium Iodide. A detailed tissue dissociation protocol is available in the supplementary data (Supplementary File 1).

The cell suspension was loaded on a 10X Genomics Chromium Controller™, and cell capture was performed following the Chromium Next GEM Single Cell 3’ version 3.1 protocol, a process by which transcripts from every cell are labelled with a barcoded oligonucleotide. RT-PCR allowed the synthesis of cDNA from each cell’s transcriptome. cDNA quality was checked via electrophoresis on an agarose 2% E-gel precast system (Invitrogen, Cat. No. G402022) (Supplementary Fig. 1). Subsequently, a cDNA library was prepared for sequencing. Paired-end sequencing was performed on an Illumina NextSeq500 platform. Sequenced short reads were de-multiplexed and converted into compressed “(fastq)”-formatted files.

### Quality control and mapping of raw data

The bioinformatics tool FastQC (https://www.bioinformatics.babraham.ac.uk/projects/fastqc/) was used to check the quality of the sequencing. A report is available in the supplementary data (Supplementary File 2).

The *Parasteatoda* genome (version Ptep 3.0) and corresponding annotation were downloaded from NCBI (https://www.ncbi.nlm.nih.gov/genome/?term=Parasteatoda+tepidariorum).

High levels of mitochondrial transcripts indicate cell stress, which would render the cells unfit for downstream analysis. To identify and remove such cells from our data, a nearly complete mitochondrial genome was assembled and concatenated to the genome prior to mapping. For its assembly, raw DNA sequencing reads (SRR891584) were used. These reads come from the BioProject PRJNA167405 conducted to assemble the *Parasteatoda* genome [166]. The mitochondrial genes present in this dataset were searched for and assembled using GetOrganelle v1.7.3.3, a toolkit for de novo assembly of organelle genomes (https://github.com/Kinggerm/GetOrganelle), with kmer sizes 21, 45, 65, 85, 105. The scaffold was annotated using the MITOS server [17], and this information was added to the gene coordinates file (in GTF format) of the genome. The assembled mitochondrial scaffold (“.txt”) is available in the supplementary data (Supplementary File 3).

Mapping of the reads to the reference genome was done using the CellRanger ‘mkref’ and ‘count’ pipelines from the Single Cell Software Suite offered by 10 × Genomics (version 6.0.2). The output included a web summary in “(.html)” format (Supplementary File 3), and a folder containing three compressed files in “(.gz)” format: a list of putative genes and cells (“features.tsv” and “barcodes.tsv”, respectively), and a matrix that displays the number of unique transcripts (UMIs) per cell and per gene (“matrix.mtx”). This information is available in a compressed format “(.zip)” in the supplementary data (Supplementary File 3).

### Data processing and downstream analysis

The gene expression matrix was loaded into Seurat ver. 4.1.0 [156] to be processed for downstream analysis (i.e. dimensional reduction, clustering of cells by differential gene expression, identification of cluster markers). In order to filter out low quality cell barcodes (i.e. background RNA encapsulated in droplets, and cells undergoing stress), a subset was made that consisted of cells that express between 250 and 2500 genes, and contained between 1400 and 3500 transcripts, and in which less than 5 percent of transcripts were representing mitochondrial genes. Normalization and variance stabilization of the dataset were performed with the R package SCTransform [30, 61] following the ‘glmGamPoi’ method. A principal component analysis (PCA) was run for linear dimensional reduction. To visualize the data, 50 principal components (PCs) were selected for Uniform Manifold and Projection (UMAP) analysis. To construct a Shared Nearest Neighbours (SNN) graph, 50 PCs were used. Cells were clustered together using the Seurat function “FindClusters” at a resolution of 2. The Seurat function ‘FindAllMarkers’ was used to identify the differentially expressed genes (DEGs) to be used for cluster identification. Only the DEGs that are upregulated (only.pos = TRUE), and those that are expressed in at least 1% of the cells in a cluster (min.pct = 0.01) were considered. For more specific details regarding the parameters followed for cell filtering, clustering, and marker selection, we provide an R notebook file (“.Rmd”) and R object (“.rds”) containing our data analysis, as well as a gene annotation table (“.tsv”). These files are available in the supplementary data (Supplementary File 4). The top most differentially expressed markers per cluster were selected for in silico cell type identification (i.e. by literature review). A spreadsheet containing the markers per cluster is available in the supplementary data (Supplementary File 5). Beyond the *in-silico* analysis we also conducted additional whole-mount in-situ hybridization (WISH) experiments to a) verify *in-silico* based cluster-identification of marker genes for which WISH data were not available for *Parasteatoda*, and b) to investigate the spatial expression of marker genes for which comparative data from spiders and other organisms were not available at all, or not in sufficient extent and quality (and thus were not conclusive in the *in-silico* analysis).

### Gene amplification, probe synthesis, in-situ hybridization, nuclear staining, and data documentation

Total RNA was extracted from a mix of embryos of different developmental stages using TRIZOL (Invitrogen, Cat. No. 15596029). mRNA was isolated from this total RNA using the Dynabeads mRNA Purification Kit (Invitrogen, Cat. No. 61006). cDNA was synthesized from mRNA using RevertAid H Minus First Strand cDNA Synthesis Kit (Thermo scientific, Cat. No. K1631). For fragments of most genes, we performed an initial polymerase chain reaction (PCR), and a subsequent nested (or semi-nested) PCR with a set of internal primers (primer sequences are listed in Supplementary File 5). All backward primers were equipped with a 5´-T7-RNA promotor sequence (gggTAATACGACTCACTATAG) [37]). The three extra guanines serve as protection against degradation of the PCR fragments prior to probe synthesis. PCRs were purified using the QIAGEN PCR purification kit (QIAGEN, Cat. No. 28104). The purified PCRs directly served as templates for subsequent probe synthesis with T7 RNA polymerase (ROCHE, Cat. No. 10881767001). Synthesized probes were purified using the QIAGEN RNeasy Kit (QIAGEN, Cat. No. 74104). Whole mount *in-situ* hybridization was performed as previously described [85]. Whenever indicated, for better display, embryos were incubated in glycerol and flat-mounted. Appendages were dissected from glycerol-incubated embryos. SYBR Green (incubation of stained embryos in 1:10,000 SYBR Green in phosphate buffered saline with 0.1% Tween-20 (PBST-0.1%) for 20–30 min) was used to better visualize the morphology of embryos. Stained embryos were photographed under a MZ-FLIII Leica dissection microscope equipped with a Leica DC490 digital camera and an external UV-light source. Linear adjustments on colour, contrast and brightness were performed applying the image-processing software Adobe Photoshop 2022.

## Results and discussion

### Single-cell mRNA sequencing of stage 10–12 spider embryos

Embryonic tissue dissociation and single cell isolation were conducted using a mix of 50 *Parasteatoda tepidariorum* embryos at stages 10–12. This yielded 70 µl of suspension containing nearly 90% of live cells with a concentration of 920 cells/µl. Single cell mRNA sequencing was performed using equipment and reagents kits provided by the droplet-based technology Chromium Single Cell 3’ Gene Expression™ sold by 10xGenomics®. Following the specifications in the User Guide, 30 000 cells were loaded on the Chromium Controller™. After cell capture, the RT-PCR generated cDNA had a concentration of 1.43 ng/µl. Quality control performed via electrophoresis showed that the cDNA was of good quality, with little degradation of mRNAs (Supplementary Fig. 1). The subsequent cDNA library had a concentration of 37.6 ng/µl. Paired-end sequencing generated 330,047,610 short reads and a sequencing saturation of 11.1%. Quality control via FastQC indicated an overall good base calling process (i.e. high Phred scores for all reads).

**Fig. 3:**
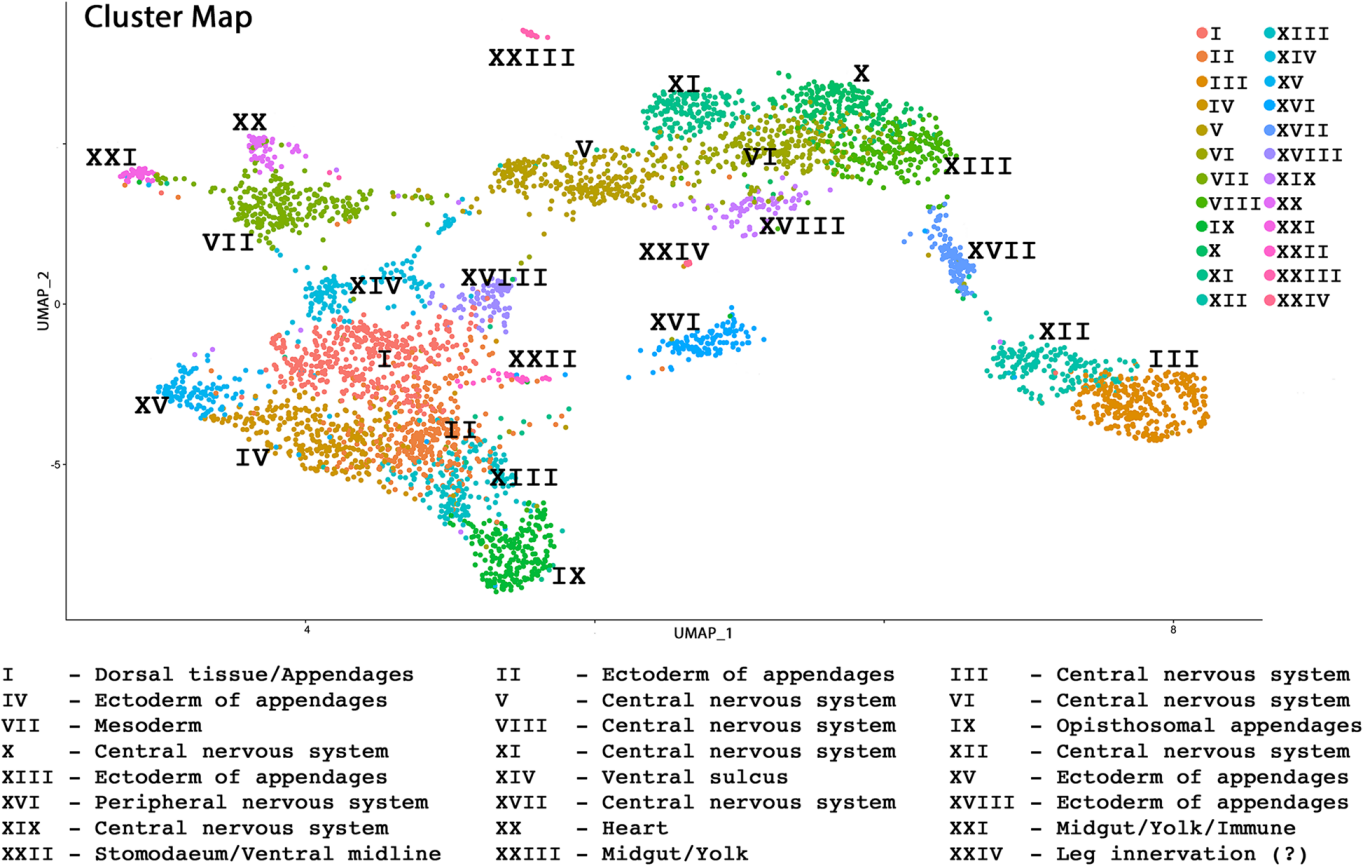
Integrated data UMAP showing 24 identified clusters. A short description of the nature of each cluster is given below the UMAP data set

Mapping the reads onto the genome, filtering and processing the resulting gene expression matrix using Cell Ranger pipelines and Seurat v4.1.0 yielded a set of 4103 cells expressing 16 669 genes. These cells were grouped into 24 clusters (indicated by Roman Numerals I-XXIV), with 2784 markers in total (Fig. 3; Table 1); note that some genes represent markers for more than one cluster.

**Table 1:** Number of markers and cells per cluster. Clusters listed in Roman numerals

Cluster	No. markers	No. cells
I	247	393
II	57	318
III	409	304
IV	148	289
V	118	281
VI	72	269
VII	110	264
VIII	144	223
IX	221	218
X	106	212
XI	43	187
XII	80	172
XIII	199	168
XIV	46	140
XV	75	122
XVI	114	120
XVII	205	108
XVIII	150	84
XIX	42	79
XX	173	58
XXI	239	41
XXII	151	27
XXIII	361	21
XIV	395	5
	**3905**	**4103**

Marker genes for each cluster were determined using a “cluster against all-other-clusters” approach, including all genes that were expressed in at least 1% of the cells of their associated cluster and a return threshold *p*-value of 0.01. “Top-marker genes” are defined as genes with the best in-cluster against all-other-clusters values, i.e. genes with the lowest *p*-value.

### Cluster analysis

#### Cluster-I: Dorsal tissue, and ectoderm patterning of developing appendages (1)

The top markers of Cluster-I have not been investigated in great detail previously, and it is therefore difficult to determine the nature of Cluster-I cells *by in-silico* analysis. Somewhat further down the list of markers, however, we find genes that have previously been studied in *Parasteatoda* and other spiders: *Frizzled*-*4.1* (*Fz4.1*) (LOC107442148) and *Frizzled-4.2* (*Fz4.2*) (LOC107441380) [82], *optomotor-blind* (*omb*)*/Tbx3* (LOC107450980) [81], *decapentaplegic* (*dpp*) (LOC107441097) [149], and *irx2* (LOC107439315) [110]. *Fz4.1* and *irx2* are strongly expressed in dorsal tissue of the developing *Parasteatoda* embryo. All of these genes have in common that they are expressed in various patterns in the developing appendages.

**Fig. 4:**
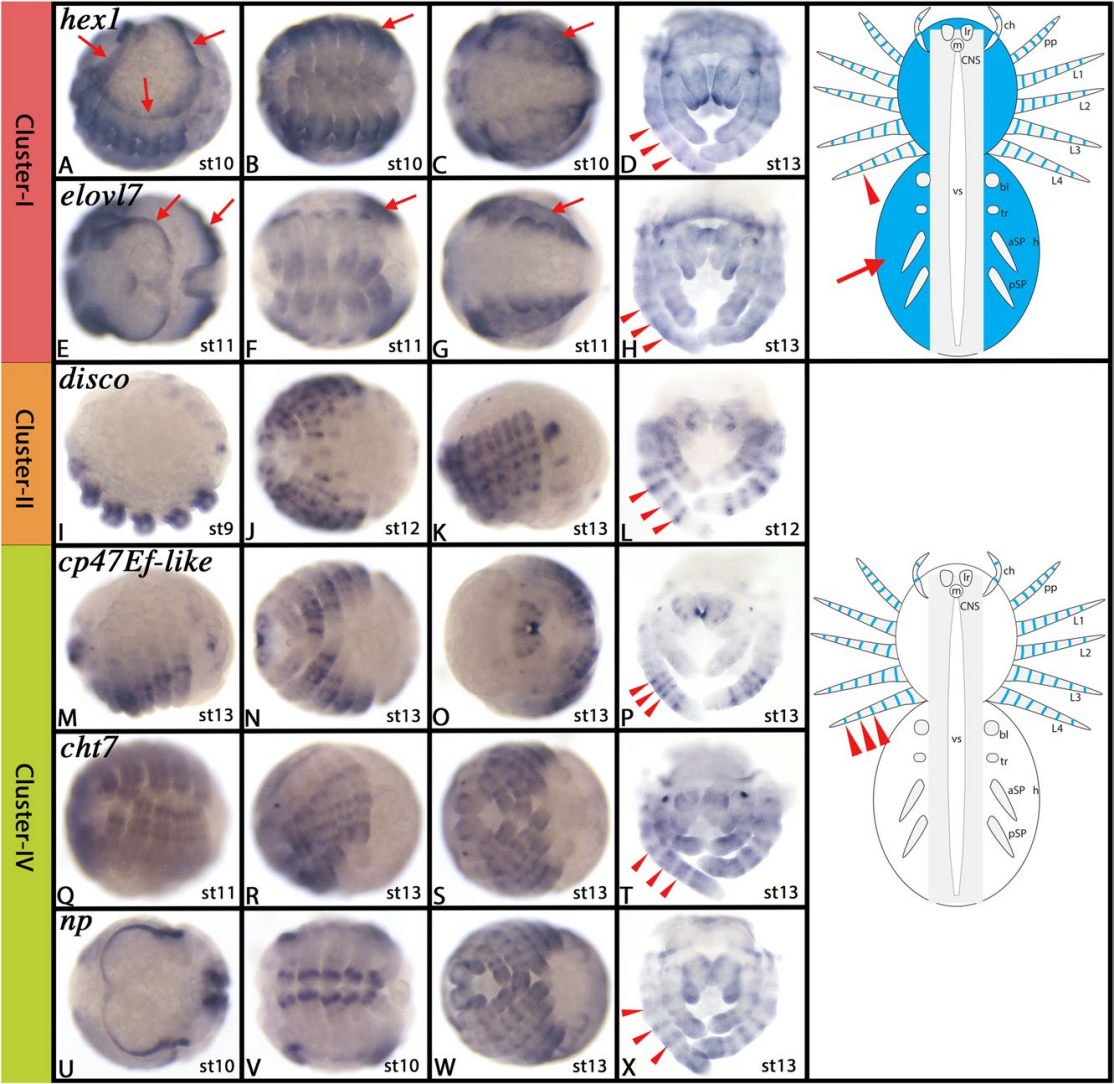
Gene expression of Cluster-I, -II, and -IV markers. In all panels, anterior is to the left, except panels D, H, L, P, T and X where anterior is up. Panels A, I, K, M and R represent lateral views. Panels B, C, E, F, G, J, N, Q, S, V and W represent ventral views. Panels D, H, L, P, T and X represent anterior views. Schematic drawings in the right column summarize the main gene expression characteristic for a given cell cluster (blue tissue). Arrows and arrowheads in schematic drawings point to comparable expression as marked by the same symbols in the original photographs. Abbreviations of gene names is indicated in the upper left corner. Developmental stages are indicated in the bottom right corner. For further information, see Supplementary Figs. 2–4 and 9–11, and Supplementary File 6. Abbreviations used in schematic drawings: aSP, anterior pair of spinnerets; bl, book lung; ch, chelicera; CNS, central nervous system; h, heart; L1-L4, first to fourth leg; lr, labrum; m, mouth; pp, pedipalp; pSP, posterior pair of spinnerets; tr, trachea; vs, ventral sulcus

We tried to substantiate the suggestion that Cluster-I cells could represent dorsal derivatives of the spider embryo and/or the developing appendages by additional whole-mount *in-situ* hybridization (WISH) experiments choosing markers that are high up in the list, and that are expressed as tissue (cluster) specific as possible. Therefore, we chose *hexosaminidase-1* (*hex1*) (LOC107442242) and *elongation of very long chain fatty acids protein 7* (*elovl7*) (LOC107446178). These two genes first are expressed in complementing leg gap-gene like patterns proximally and distally respectively and later in rings in the appendages suggesting a function in joint formation. Importantly, however, both genes are also dominantly expressed in dorsal tissue of both the pro- and opisthosoma, while ventral tissue does not express these genes (Fig. 4A-H and Supplementary Figs. 2 and 3). This pattern is very similar to the patterns of *irx2* and *Fz4.1* in dorsal tissue supporting the suggestion that the Cluster-I represents dorsal tissue.

With respect to the somewhat later expression of Cluster-I genes in what we believe are the developing joints, it is worth mentioning that also the Cluster-I marker *dpp* is expressed in a comparative pattern in the developing joints [149]. It is thus likely that Cluster-I indeed also harbours cells that are involved in spider joint formation.

#### Cluster-II: Ectoderm patterning of developing appendages (2)

Most top markers of Cluster-II represent uncharacterized genes. Among the characterized markers, however, range a number of genes that have previously been described for *Parasteatoda*, other spiders and/or other arthropods. Notably, a large number of these markers are expressed in the ectoderm of developing appendages, and are involved in appendage development, such as *disconnected* (*disco*) (aka *basonuclin*) (LOC107447159) (Fig. 4I-L and Supplementary Fig. 4), *spineless2* (*ss2*) (LOC107457395) (Fig. 10F-H, cf. Cluster-XVI), *aristaless* (*al*) (LOC107448374), *AP2.2* (LOC107443623) (Fig. 9U-X, cf. Cluster-XV) (note that Leite et al. [111] investigated another paralog of this gene, *AP2* (LOC107452006)), *Fz4.1* (LOC107442148), *Fz4.2* (LOC107441380), and *unchoordinated-5.1* (*unc5.1*) (LOC107445619) (e.g. [4, 6, 45, 64, 82, 83, 88, 109, 111, 132, 134, 141, 150, 160, 167].

We therefore believe that Cluster-II cells generally contribute to the developing appendages. At least some of the investigated genes are expressed in the form of rings that could indicate a function in proximal–distal axis patterning and thus joint formation.

#### Cluster-III: The developing central nervous system (CNS) (1) – Differentiating and differentiated neurons (1)

*In-silico* analysis of Cluster-III gene markers strongly suggests that these cells represent part of the developing nervous system. Best markers are *sax3* (LOC107455609), and two paralogs of *neural-cadherin* (*Ncad1* (LOC107454545) and *Ncad2* (LOC107451854)). Other high-ranked markers are *follistatin-related protein 5* (*fstl5*) (LOC107441591), *Dscam-2* (LOC107456604), *pikachurin* (*pika*) (LOC107439395), and *ELAV-like protein 3* (*Elavl3*) (LOC107436216). Of these markers, *sax3, pika*, *fstl5*, and *Elavl3* are unique markers for this cell cluster (see Supplementary Fig. 5). *Ncad1*, *Ncad2* and *Dscam2* are markers of both Cluster-III and the closely related Cluster-XII (cf. chapter on Cluster-XII). These genes are all involved in neuron development, neuron differentiation, and neuronal pathfinding, and thus also in differentiated neurons (e.g. [36, 43, 66, 75, 92, 140, 151, 191, 203]. Beyond that, many of the lesser markers of this cluster indeed also represent genes typically expressed in neurons, such as the Dscams (e.g. LOC107456604, LOC107444681, LOC107450135, LOC107445852, LOC107452025, LOC107448201, LOC107443039, LOC107455980) (e.g. reviewed in [171], and synaptotagmins (LOC107455395, LOC107449342) (e.g. reviewed in [195]. It is therefore reasonable to suggest that Cluster-III cells represent differentiating and mature neurons.

**Fig. 5:**
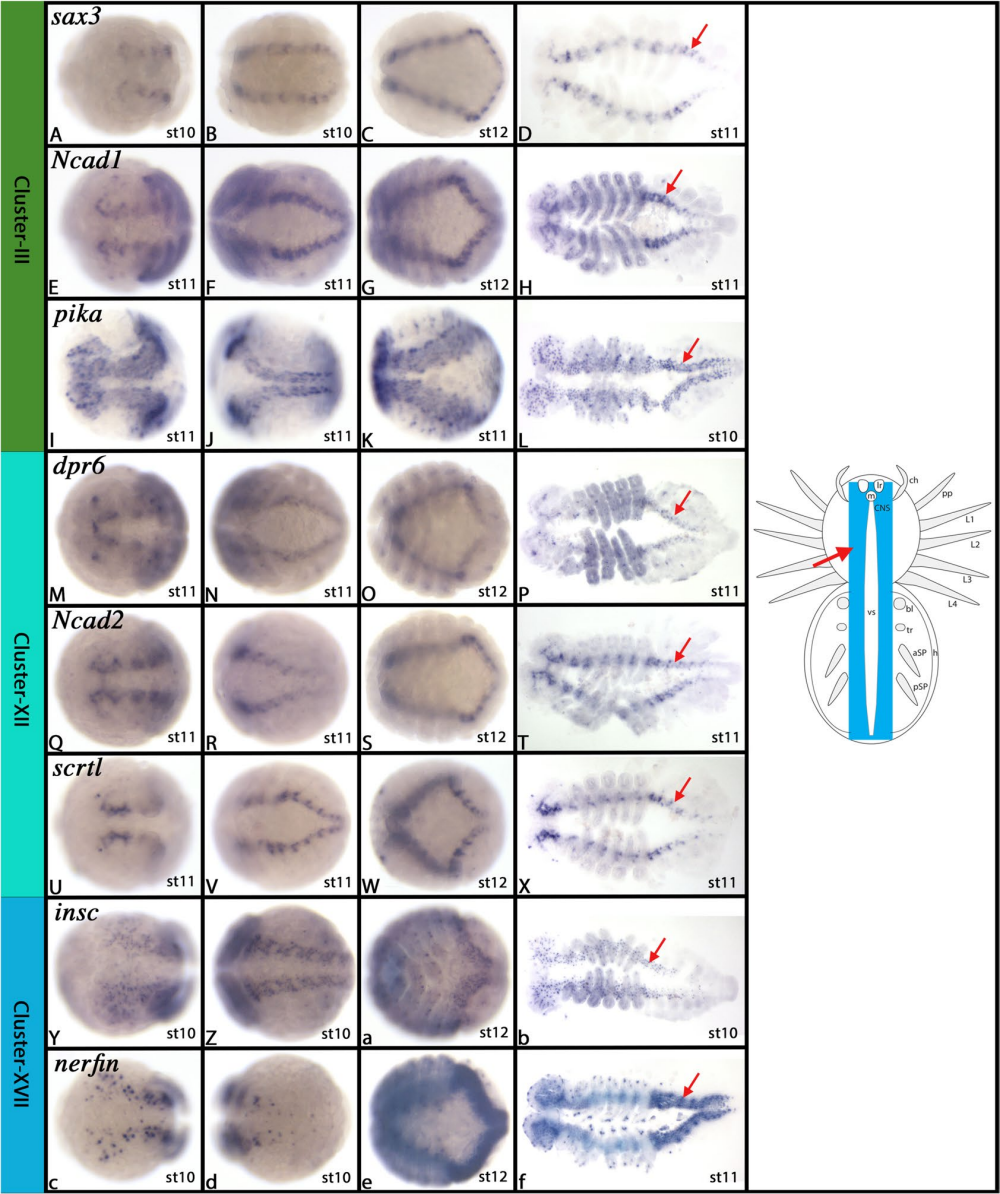
Gene expression of Cluster-III, XII, and -XVI markers. In all panels, anterior is to the left. All panels represent ventral views. Panels D, H, L, P, T, X, b, and f show flat-mounted embryos. Schematic drawings in the right column summarize the main gene expression characteristic for a given cell cluster (blue tissue). Arrow in schematic drawings point to comparable expression as marked by the same symbol in the original photographs. Abbreviations of gene names is indicated in the upper left corner. Developmental stages are indicated in the bottom right corner. For further information, see Supplementary Figures B5-B8, B28-B31 and B46-B48, and Supplementary File 6. For abbreviations used in schematic drawings see legend of Fig. 4

The expression and function of most of these genes has not been studied in great detail in arthropods other than *Drosophila*, and thus data from spiders are not available. *In-situ* hybridization of some of these genes show, as expected, that they are expressed in the developing central nervous system (CNS), and some are expressed in the peripheral nervous system (PNS) too (Fig. 5A-L and Supplementary Figs. 5–8).

#### Cluster-IV: Ectoderm patterning of developing appendages (3)

Most of the top markers of Cluster-IV have not been studied in great detail, or not at all. Two of these markers, *cuticular protein 47Ef-like* (*cp47EF-like*) (LOC107436879) and *chitinase-7* (*cht7*) (LOC107437310), are exclusively expressed in the developing joints (Fig. 4M-T and Supplementary Figs. 9 and 10). Another marker, the protease *notopleural* (*np*) (LOC107439948), is expressed in dorsal tissue and what we believe are the developing joints of the appendages, very similar like the markers of the closely-related Cluster-I and the aforementioned markers of Cluster-IV (Fig. 4U-X and Supplementary Fig. 11). Interestingly, another marker of Cluster-IV is *trachealess* (*trh*) (LOC110283103) which has been studied in *Parasteatoda* and other chelicerates such as a harvestman and a scorpion, is also expressed in the form of rings in the developing appendages suggesting a possible role in joint formation [168]. Likewise, *AP2.2* (LOC107443623) (cf. Cluster-XV) is expressed in the form of rings in the appendages (Fig. 9U-X). Other previously investigated and less prominent markers of this cluster (e.g. the two *Fz4* ohnologs (LOC107442148, LOC107441380) [82], *Wnt6*, *Wnt5*, and *Wnt1* (LOC107438387, LOC107445649, and LOC107438386) [87], and *unc5* [88] are also expressed in the developing appendages, albeit not in the form of rings. Gene expression analysis of the high-ranked markers of this cluster, however, suggests that these cells could contribute to the development of the joints of the spider appendages.

#### Cluster-V: The developing central nervous system (CNS) (2) – EMT-like processes in neural precursor determination?

Top markers of this cluster are *neurotactin* (*nrt*) (LOC107441543), *magu* (LOC107453771), *zinc finger protein 395-like* (*zfp395-like*) (LOC122271089), *fasciclin*-2 (*fas2*) (LOC107436358), *noggin* (*nog*) (LOC107444265) and *otopetrin-2-like* (*otop2*) (LOC107451015).

In *Drosophila, nrt* is involved in morphogenic movements and is expressed in dynamic patterns in both mesodermal and ectodermal tissues including the developing nervous system where it is expressed strongly [39]. The expression of *Drosophila magu* is not known, but over-expression of *magu* in *Drosophila* causes an elongated life span, especially when it is overexpressed in the nervous system [112]. To our knowledge, there are no data on *zfp395-like*. In *Drosophila*, the cell adhesion molecule *fas2* is an important factor of synaptic growth and maintenance [57, 165]. In vertebrates, *nog*1 induces neural tissue development (reviewed in e.g. [98]. These genes have not been studied in spiders prior to this study, except for the recent data on *nog* showing expression in earlier developmental stages [111].

**Fig. 6:**
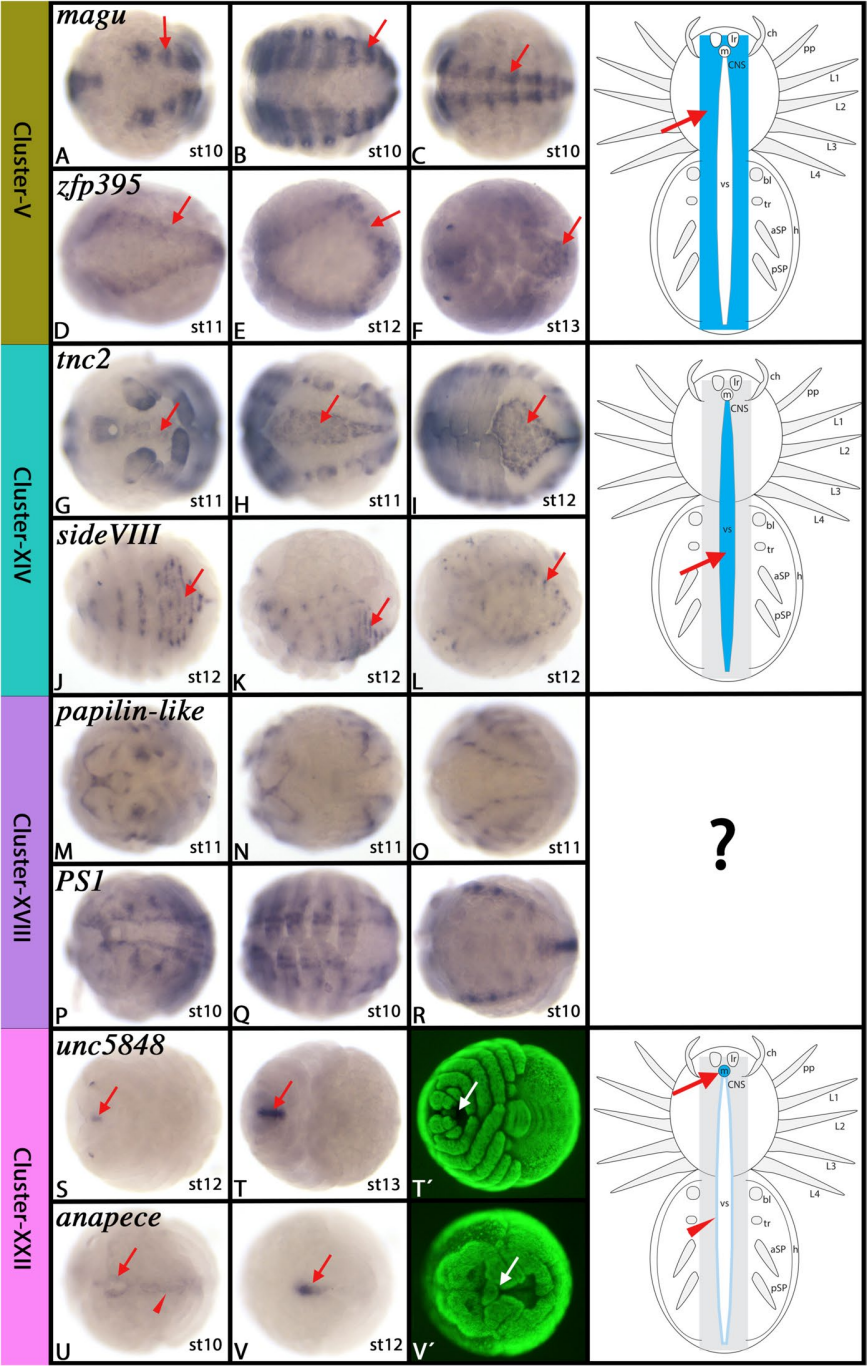
Gene expression of Cluster-V, -XIV, -XVIII and -XXII markers. In all panels, anterior is to the left. All panels represent ventral views, except panels K (lateral view), N (dorsal view), and V/V´ (anterior view). T´ and V´ represent SYBR-green staining of the embryos shown in panels T and V. Schematic drawings in the right column summarize the main gene expression characteristic for a given cell cluster (blue tissue). Arrows and arrowheads in schematic drawings point to comparable expression as marked by the same symbol in the original photographs. For Cluster-XVIII, it was not possible to determine a specific tissue marked by these genes. Abbreviations of gene names is indicated in the upper left corner. Developmental stages are indicated in the bottom right corner. For further information, see Supplementary Figs. 12–15, 35–37, 49–51, and 60–63, and Supplementary File 6. For abbreviations used in schematic drawings see legend of Fig. 4

Some other significant markers of Cluster-V, however, have been investigated previously in *Parasteatoda* or other species of spiders. *netrin-2* (*net2*) (LOC107450632) for example is prominently expressed in the developing nervous system, the heart, and the appendages [88, 118], and *snail* (*sna*) (LOC107443696) is prominently expressed in the developing nervous system of the American wandering spider *Cupiennius salei* [177, 193]. In summary, these data suggest that Cluster-V cells are involved in the development of the nervous system. Our gene expression analysis supports this as all investigated genes of this cluster are expressed *inter alia* in the developing CNS (Fig. 6A-F and Supplementary Figs. 12–15).

Unlike the expression of other CNS-Cluster genes (e.g. clusters VI, X, XII, and XVII), markers of Cluster-V are also expressed in other tissues such as the visceral mesoderm, the heart and the dorsal field. These aspects of expression are very similar to the pattern of Cluster-VII (discussed below) and may hint to a function in visceral mesoderm development and epithelial-to-mesenchymal transition (EMT). Although Cluster-V cells still show a neural fingerprint, this fingerprint hints to a “near” transition of these cells into a possible mesenchymal nature. Alternatively, Cluster-V cells represent another developmental state of CNS development in which future neural precursors are selected and delaminate from the “neural epithelium”, in an EMT-related process (e.g. [7].

#### Cluster-VI: The developing central nervous system (CNS) (3) – Early differentiating neural cells (1)

**Fig. 7:**
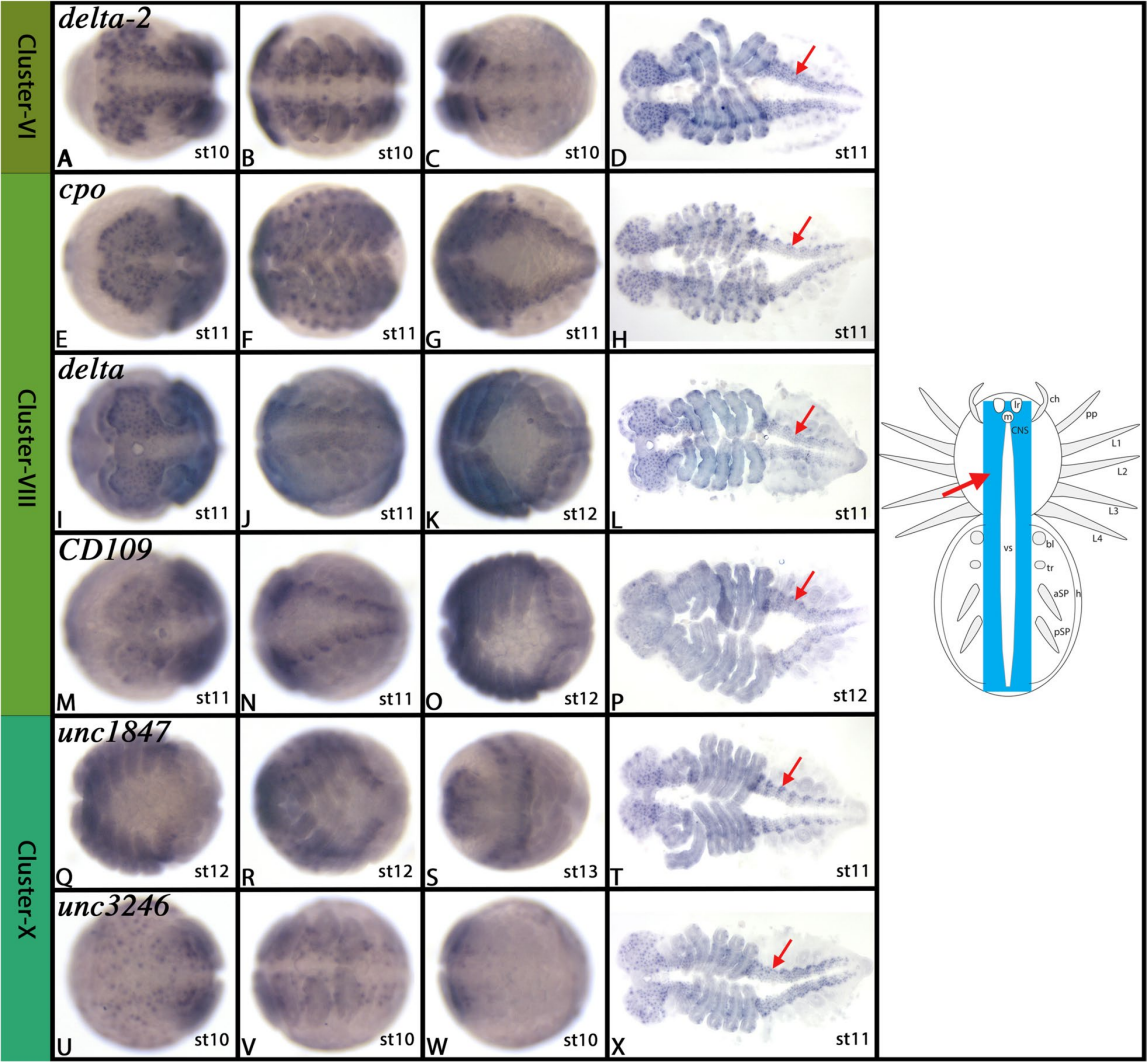
Gene expression of Cluster-VI, -VIII, and -X markers. In all panels, anterior is to the left. All panels represent ventral views. Panels D, H, L, P, T and X represent flat-mounted embryos. Schematic drawings in the right column summarize the main gene expression characteristic for a given cell cluster (blue tissue). The arrow in the schematic drawing points to comparable expression as marked by the same symbol in the original photographs. Abbreviations of gene names is indicated in the upper left corner. Developmental stages are indicated in the bottom right corner. For further information, see Supplementary Figs. 16, 20–23, 26, and 27, and Supplementary File 6. For abbreviations used in schematic drawings see legend of Fig. 4

Top markers of Cluster-VI are *delta-2* (LOC107438011), and the uncharacterized gene *unc1852* (LOC107451852). *delta* is a well-known neurogenic gene (e.g. [60, 190]. Two other genes that are markers of Cluster-VI, *cyclin D* (*cycD*) (LOC107457517) and *cyclin dependent kinase 1* (*CDK1*) (LOC107436497) are known factors of cell cycle regulation and have been reported to be highly expressed in the developing nervous system (e.g. [58, 71, 86]. Further down the list of markers, we also find the genes *dachshund* (*dac*) (LOC107453438) and *Nkx6.2* (LOC107450777) (the latter has recently been studied by [111]. We confirm in this study that *Nkx6.2* (Fig. 10Q-T, cf. Cluster-XIX) and *delta-2* (Fig. 7A-D and Supplementary Fig. 16) are expressed exclusively in the developing nervous system of the spider. Previous studies have shown that *dac* is expressed in the developing nervous system of *Parasteatoda* and other spiders [144, 149, 187]. These data and the fact that Cluster-VI is encircled by other cell clusters that likely represent cells of the developing spider nervous system (cf. clusters V, VIII, X, XI, and XIX), strongly suggest that Cluster-VI represents a subtype of cells of the developing nervous system. Notably, none of the detected Achaete-Scute complex (ASH-C) genes is expressed in Cluster-VI, but are expressed in the neighbouring clusters XIII, X and XVII (Supplementary File 5), suggesting that Cluster-VI cells do not represent the earliest steps of CNS development.

#### Cluster-VII: The developing mesoderm (1) – EMT-like processes in visceral mesoderm development?

*In-silico* analysis of Cluster-VII markers gives some insight into the possible nature of the cells represented by this cluster. Top markers are *integrin alpha-PS2* (*PS2*) (LOC107444850), *collagen alpha-1(I) chain* (LOC107442626), its paralog (LOC107443413), several other collagen genes (LOC107442286, LOC107441250, LOC107442289), several laminin genes (LOC107439460, LOC107452433, LOC107448892), *papilin* (LOC107447945), and a *fibrinogen-like* gene (LOC107449293).

Many of these genes represent cell adhesion molecules needed for cell migration as present during epithelial-to-mesenchymal transition (EMT) (reviewed in e.g. [14, 73]. These and other genes such as *ECM protein 3-like* (LOC122271235) and *ECM organizing protein Fras1* (LOC107444379) are components of the extracellular matrix (ECM) that is used as a substrate for cell migration (reviewed in e.g. [181]. Interestingly, in *Drosophila*, the visceral mesoderm serves as a substrate for many populations of migrating cells (e.g. [22, 153], thus connecting cell migration, visceral mesoderm and the ECM. Among the integrins, *PS2*, the top marker of this cluster, is specifically expressed in the visceral mesoderm (e.g. [21, 128]. Another group of genes that are among the top markers of this cluster are the fibroblast growth factor receptors (FGFRs) (LOC107437526, LOC107438841, LOC107445728) which are involved in the development of the visceral mesoderm, somatic muscles and the heart (e.g. [122, 172]. *papilin* is an important factor of basement membrane development of somatic and visceral muscles, interacts with collagen genes, and is generally involved in cell movement [28, 97], reviewed in [48]. Finally, we also found *FoxF1* (LOC107456534) to be a marker of this cell cluster, a gene that is known to be involved in the development of the visceral mesoderm in *Drosophila* (e.g. [78, 146]. A recent study has shown that in *Parasteatoda*, *FoxF1* is almost exclusively expressed in the visceral mesoderm of the opisthosoma [89].

**Fig. 8:**
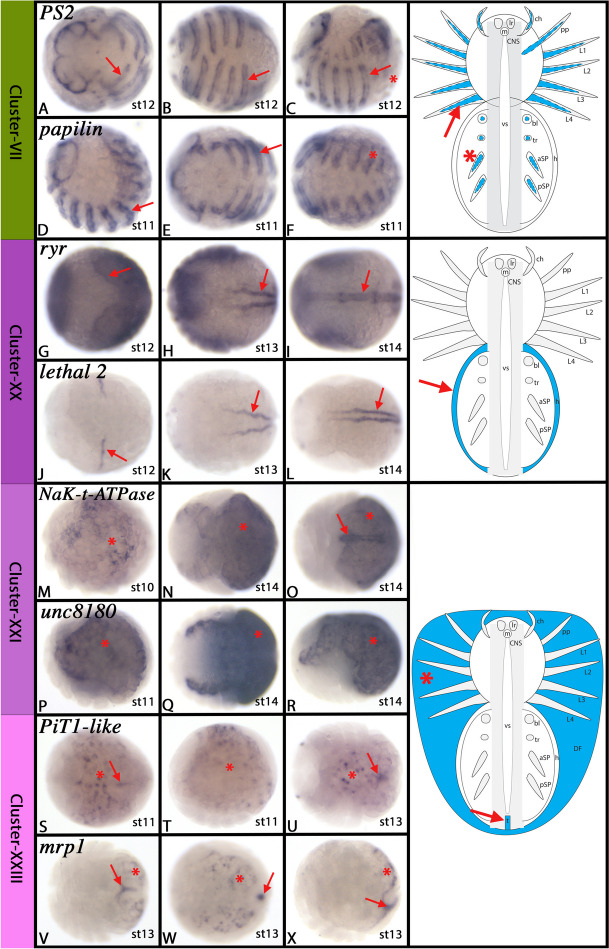
Gene expression of Cluster-VII, -XX, -XXI and -XXIII markers. In all panels, anterior is to the left. Panels A, B, E, F, U and V represent ventral views. Panels C, D, M, P, R, T and X represent lateral views. Panels G-L, N, O, Q, S and W represent dorsal views. Schematic drawings in the right column summarize the main gene expression characteristic for a given cell cluster (blue tissue). Arrows and asterisks in schematic drawings point to comparable expression as marked by the same symbol in the original photographs. Abbreviations of gene names is indicated in the upper left corner. Developmental stages are indicated in the bottom right corner. For further information, see Supplementary Figs. 17, 18, 54–56, 57–59, and 64–66, and Supplementary File 6. For abbreviations used in schematic drawings see legend of Fig. 4

We performed *in-situ* hybridization experiments with top markers of this cluster and show that they all are expressed *inter alia* in the visceral mesoderm of the trunk, the mesoderm of the appendages, and the developing heart (Fig. 8A-F and Supplementary Figs. 17–19). It is thus likely that Cluster-VII genes represent cells that are in the process of EMT and that are developing towards becoming cells of the visceral mesoderm (cf. Cluster-V).

#### Cluster-VIII: The developing central nervous system (CNS) (4) – Early differentiating neuronal cells

The top marker of this cluster is *couch potato* (*cpo*) (LOC107453124), a gene that in *Drosophila* is involved in the development of neuronal precursors, and the sensory nervous system (e.g. [16, 54]. Other top markers are *Chronophage* (*Cph*) (LOC107438166), the two ohnologs of *delta* (LOC107456525, LOC107438011), *CD109 antigen* (*CD109*) (LOC107436484) and *epidermal growth factor receptor* (*egfr*) (LOC107446048). *Cph* has recently been identified as a temporal switch of nerve cell subtype specification [51]. One of the two paralogs of *Parasteatoda delta* (LOC107456525) has been described previously, and it has been shown to be expressed in the developing nervous system around stage 9 (later stages were not presented in this study) [138]. In another spider, the American wandering spider *Cupiennius salei*, both ohnologs of *delta* have been investigated, and it has been shown that both are expressed early in the developing nervous system [176]. In *Drosophila*, *delta* represents a neurogenic gene, and is thus involved in cell fate determination within the developing nervous system (reviewed in e.g. [136]. *CD109* is involved in *Drosophila* septate junction formation, but to our knowledge there is no data about its potential function during nervous system development [10]. Finally, *egfr* represents an important factor of nervous system development as EGFR-signalling interacts with TOR-signalling and thereby contributes to neuronal differentiation [93, 94]. *In-silico* analysis thus suggests that Cluster-VIII cells represent nerve cells that are in the (relatively) early process of differentiation. We investigated the embryonic gene expression profiles of *cpo*, *delta*, *delta-2*, *CD109*, and an uncharacterized marker of this cluster (*unc6289*) (LOC107436289). These genes all are expressed almost exclusively in the developing nervous system, thereby supporting the conclusion drawn from *in-silico* analysis (Fig. 7E-P and Supplementary Figs. 20–23).

#### Cluster-IX: Opisthosomal appendages

**Fig. 9:**
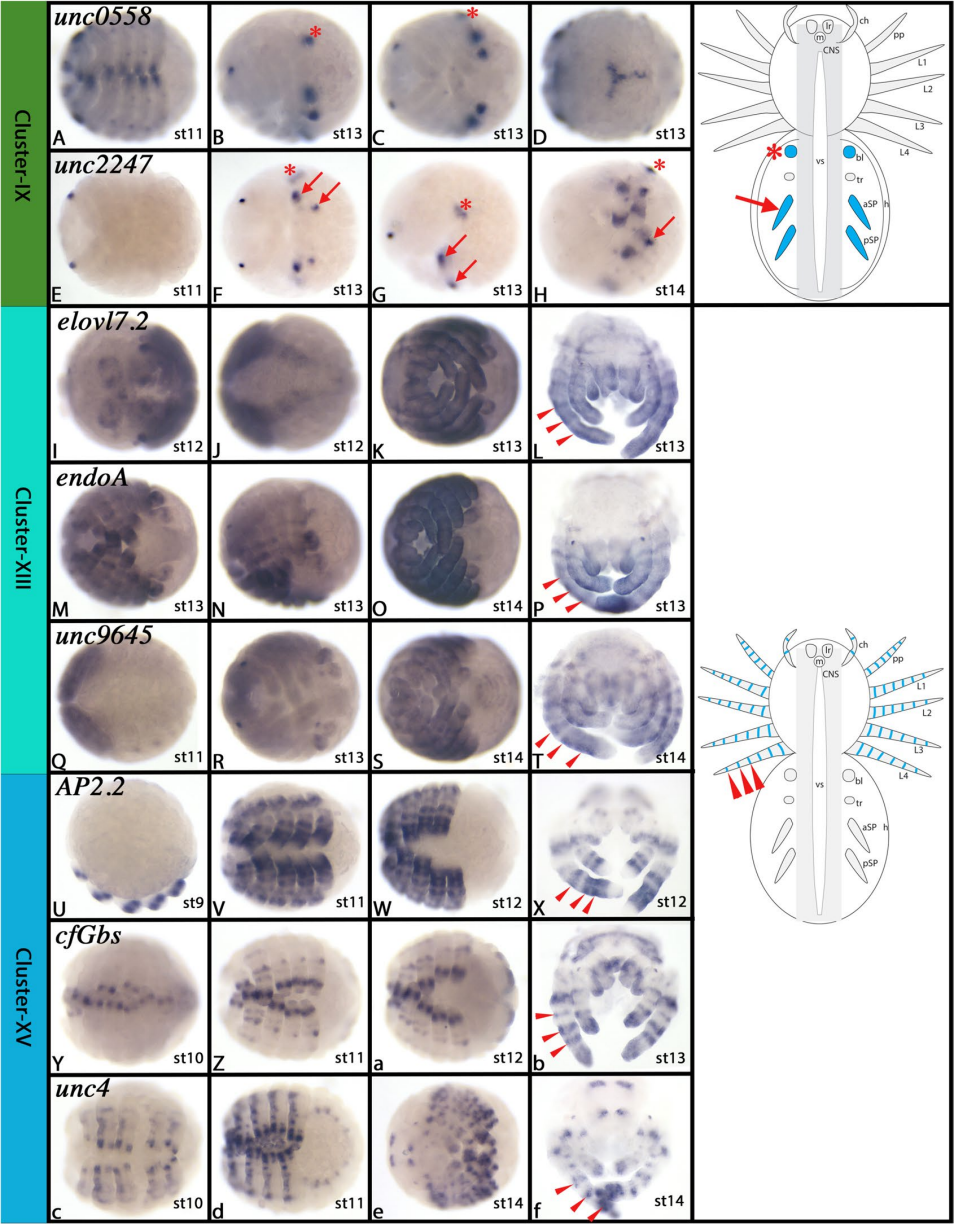
Gene expression of Cluster-IX, -XIII, and -XV markers. In all panels, anterior is to the left. Panels A, C, E, F, H, I-K, M, O, Q-S, V, W, Y-a, and c-e represent ventral views. Panels B, G, and N represent lateral views. Panel D represents dorsal view. Panels L, P, T, X, b, and f represent anterior views on flat-mounted head regions. Schematic drawings in the right column summarize the main gene expression characteristic for a given cell cluster (blue tissue). Arrows, arrowheads and asterisks in schematic drawings point to comparable expression as marked by the same symbol in the original photographs. Abbreviations of gene names is indicated in the upper left corner. Developmental stages are indicated in the bottom right corner. For further information, see Supplementary Figs. 24, 25, 32–34, and 38–41, and Supplementary File 6. For abbreviations used in schematic drawings see legend of Fig. 4

The two top markers of this cluster represent the uncharacterized genes *unc0558* (LOC107440558) and *unc2247* (LOC107452247). These genes, and other markers of this cluster do not allow any *in-silico* prediction. These two genes are expressed in the developing spinnerets and book lungs (Fig. 9A-H and Supplementary Figs. 24 and 25). Cluster-IX could thus represent cells of the highly-modified opisthosomal appendages. Another previously studied gene of this cluster is one of the two ohnologs of *trachealess* (*trh*) (LOC107455153) which is expressed more specifically in the developing tracheae of spiders than its paralog (LOC110283103) which itself is expressed in the developing tracheae, but also strongly in legs and dorsal tissue (a marker of clusters IV, XIII, XV, and XVIII) (Zhang (née Turetzek) 2016).

#### Cluster-X: The developing central nervous system (CNS) (5) – Early differentiating neural cells (2)

One of the two ohnologs of the neurogenic gene *delta*, *delta-2* (LOC107438011) represents the strongest marker of this cluster, followed by *CD109* (OC107436484) (cf. Cluster-VI). Other high-ranked markers are the uncharacterized genes *unc1847* (LOC107451847) (cf. clusters VI and XIII) and *unc3246* (*LOC110283246*) (cf. clusters VI, VIII and XI), and the *histone-lysine N-methyltransferase MECOM* (LOC107436282) (cf. clusters V, VI, VIII, XI, XVII, and XIX). Cluster-X cells thus clearly share a large number of markers with its neighbouring clusters VI, VIII and XI. One potentially crucial difference between Cluster-X and clusters VI and VIII could be the presence of *cyclin-dependent kinase inhibitor 1* (*CKI1*) (LOC107443706) which likely interacts with *cycD* (another high-ranked marker of Cluster-X) (LOC107457517) (cf. clusters VI and VIII) [170]. Clusters VI, VIII and X could thus represent cells in a different stage of cell cycle (see distribution of *cycD*, *CDK1*, *CKI1* genes) in the developing nervous system (reviewed in [117]. Interestingly, *CKI1* is also involved in neural stem cell differentiation and neuron induction by silencing *Sox2* (= *SoxN*), a top marker of cluster-XI [113, 126], reviewed in [117]. Unfortunately, we do not have gene expression data on *cycD*, *CDK1* and *CKI1*, but it has recently been proposed that *cycD* and *CDK1* (and other cycs and CDKs) are expressed in waves of expression in the nervous system of the onychophoran *Euperipatoides kanangrensis* [86]. Cluster-X or clusters VI, X and VIII could thus represent early differentiating neural cells, and Cluster-XI, in which *SoxN* (LOC107457313) is highly expressed, could represent neural precursors (discussed below). In line with this assumption is the fact that the proneural gene *achaete scute homolog 1* (*ash1*) (LOC107451231) is also among the higher-ranked markers of Cluster-X. In *Drosophila*, achaete-scute complex genes are expressed in segregating neuroblasts (neuronal precursors), but not in neurons [25, 174, 175]. Interestingly, however, studies in insects other than *Drosophila*, such as the beetle *Tribolium castaneum*, and in crustaceans, have shown that the function of *ash* as the first gene to be expressed in developing neuroblasts is not conserved, but instead there seems to be more flexibility in the patterning of the early nervous system (e.g. [188, 194]. What all these studies show, however, is that *ash* is an early factor of nervous system development.

*In-situ* hybridization analysis of previously unstudied genes that are among the top markers of this cluster reveals that they, as expected, are expressed predominantly in the developing nervous system (Fig. 7Q-X and Supplementary Figs. 26 and 27) (cf. Cluster-VIII for *CD109* expression and Cluster-VI for *delta-2* expression).

#### Cluster-XI: The developing central nervous system (CNS) (6) – Neural precursors

Two highly-ranked markers of Cluster-XI are *SoxN* (LOC107457313), a well-known factor of stem cell maintenance in the developing nervous system [113, 126], reviewed in [47], and *snail* (*sna*) (LOC107443696), an important factor of stem cell maintenance in the developing nervous system (e.g. [9, 27]. Both genes are prominently expressed in the developing CNS of spiders [20, 177, 193]. Most interestingly, both genes represent proneural genes that link neural progenitor (neuroblasts in *Drosophila*) selection with epithelial-to-mesenchymal transition (EMT) [7] (cf. *sna* as a marker of Cluster-V). Another highly-ranked marker of this cluster is another potential proneural gene in *Drosophila*, *goosecoid* (*gsc*) (LOC107439785) [62]. *gsc* is involved in neuroblast delamination in vertebrates (e.g. [96]. The homeobox gene *Dbx* (LOC122269629) is expressed in the developing nervous system of *Parasteatoda* [110], and in vertebrates, its two paralogs are expressed in neural progenitors (e.g. [148]. In *Drosophila*, the single *Dbx* gene is expressed in interneurons that form from a subset of neural progenitor cells (neuroblasts) [106]. Finally, the trunk-determining zinc finger transcription factor *tiptop/teashirt* (*tio/tsh*) (LOC107441771) is expressed in the CNS of the trunk of *Parasteatoda* [125, 129]. In *Drosophila*, *tio* and *tsh* are expressed in the trunk CNS as well, and it has been shown that both genes can mediate the survival of neurons [107, 135]. Given the genetic fingerprint of Cluster-XI it is possible that these cells represent neural precursor cells.

#### Cluster-XII: The developing central nervous system (CNS) (7) – Differentiating and differentiated neurons (2)

Cluster-XII is closely related to Cluster-III. Top markers of Cluster-XII are the uncharacterized gene *unc3142* (*LOC107443142*), *defective proboscis extension response 6* (*dpr6*) (LOC107436606), *neural-cadherin 2* (*Ncad2*) (LOC107451854), *collier* (*col*) (aka *knot*) (LOC107437107) and a *scratch-like* (*scrtl*) gene (LOC107456893).

*col* has previously been studied in spiders, and indeed a wide range of arthropods, showing that it has a conserved role in nervous system development and neuronal differentiation [13, 35, 41, 158]. Among arthropods, *scrt* has only been investigated in *Drosophila* (*CG1130*) where it is expressed in most (or all) neural precursors promoting neuronal development [154]. Information on *Drosophila scrtl* (*CG12650*) is restricted to gene expression data, showing that it is expressed in the developing CNS (BDGP in situ homepage: https://insitu.fruitfly.org/cgi-bin/ex/report.pl?ftype=3&ftext=LP01683). *dpr6* and *Ncad2* are also key-factors of *Drosophila* nervous system development that are involved in the self-organization of neurons and the specification of neuronal subtypes (e.g. [3, 19, 34, 186]. Together, this strongly suggests that Cluster-XII represents cells of the developing nervous system, and indeed, *in-situ* hybridization experiments confirm this suggestion: In *Parasteatoda*, *unc3142*, *scrtl, dpr6* and *Ncad2* all are expressed exclusively in the CNS (Fig. 5M-X and Supplementary Figs. 28–31). Beyond that, Cluster-XII is like its neighbouring cluster (Cluster-III) characterized by a significant number of Dscams (LOC107450135, LOC107445852, LOC107444681, LOC107456604). In summary, it is therefore likely that Cluster-XII cells represent differentiating and differentiated neurons (cf. Cluster-III).

#### Cluster-XIII—Ectoderm patterning of developing appendages (4)

Among the markers of Cluster-XIII are very few previously investigated genes. Top markers are *elongation of very long chain fatty acids protein 7.2* (*elovl7.2*) (LOC107441276) and *endochitinase A* (*endoA*) (LOC107442511). We investigated the *in-situ* hybridization patterns of these two markers and another top marker, the uncharacterized gene *unc9645* (*LOC107439645*). All three marker genes have in common that they are expressed in the form of rings in the developing appendages, indicating a function in appendage ectoderm patterning and thus likely also joint formation (F9g. 9I-T and Supplementary Figs. 32–34).

#### Cluster-XIV: The ventral sulcus (VS)

The top markers of Cluster-XIV represent two Tenectin-like genes, *Tenectin1* (*Tnc1*) (LOC107436695) and *Tenectin1* (*Tnc2*) (LOC122273808). Additional top markers of this cluster are a *sidestep* (*side*)/*hemicentin-2* like gene hereafter called *sidestep VIII* (*sideVIII*) (LOC107456176), *netrin-1* (LOC107455212) and *slit* (LOC107443293 & LOC122270376). *netrin-1* has previously been investigated in spiders. It is expressed *inter alia* in the ventral sulcus (VS) (*aka* the ventral midline epithelium [119] where it is likely involved in axon guidance [88, 118]. *slit* genes encode ligands of the Roundabout receptors and are thus important key players in axon guidance as well (reviewed in [199]. Likewise, *hemicentin-2/sidestep* functions as an important guidance cue for growing axons (e.g. [173]. Finally, also *Tnc* is involved in nervous system development in *Drosophila* where it is expressed in both longitudinal and commissural axon tracts that span the ventral midline [52, 192]. We therefore assume that Cluster-XIV cells represent cells of the VS, a suggestion that is backed-up by *in-situ* hybridization that confirmed that top markers of this cluster (*Tnc1, Tnc2,* and *sideVIII*) indeed are expressed in either the complete VS, or, like *netrin-1*, in transverse stripes spanning the VS (Fig. 6G-L and Supplementary Figs. 35–37). Another high-ranked marker of this cluster, *vitamin K-dependent protein C* (*vitK-C*) (LOC107451660) has recently been studied by Leite et al. [111] showing that it is exclusively expressed in the ventral midline region prior to ventral splitting,data on developmental stages that possess the split ventral midline and thus the VS are unfortunately not shown in their paper.

#### Cluster-XV: Ectoderm patterning of developing appendages (5)

Top markers of Cluster-XV are the uncharacterized gene *unc4096* (*LOC107444096*), *clotting factor G subunit* (*cfGbs*) (LOC107446841), the transcription factor *AP2.2* (LOC107443623) and the homeobox containing gene *unc-4 like* (LOC107444630) (*Drosophila DPHD-1*). Information about the two top markers *unc4096* and *cfGbs* is not available. *AP2*, however, has been investigated in some detail. In *Drosophila*, this gene is expressed *inter alia* in the leg and antennal discs [12, 99]. A previous study in another spider, *Cupiennius salei*, has shown that *AP2* is expressed in the appendages and functions during leg development [150]. The *unc-4 like* gene has been studied in *Drosophila* where it is expressed in the developing nervous system, in a segment-polarity like pattern in the epidermis, and in the eye-antennal disc [179].

A closer look at the Cluster-XV markers also reveals a large number of genes that are known factors of arthropod (and indeed spider) appendage development, such as *clawless/C15* (LOC107451627) Zhang [201] 2016, *dally* (LOC107446074) [72], *distal-less* (LOC107450100) [1, 145, 164], *dachsous* (LOC107449611) [124], the Hox gene *Deformed* (LOC107444120) [166], *unc5.1* (LOC107445619) [88], *optomotor-blind* (LOC107450980) [81], *SP6/9* (LOC107448645) [102] and *Fz4.2* (LOC107441380) [82]. *In-situ* hybridization of the top markers in this cluster revealed that these genes all are expressed strongly in the ectoderm of the development appendages, many of them in the form of rings, indicating a role in proximal–distal axis patterning and joint formation (Fig. 9U-f and Supplementary Figs. 38–41).

#### Cluster-XVI: The peripheral nervous system (PNS)

**Fig. 10:**
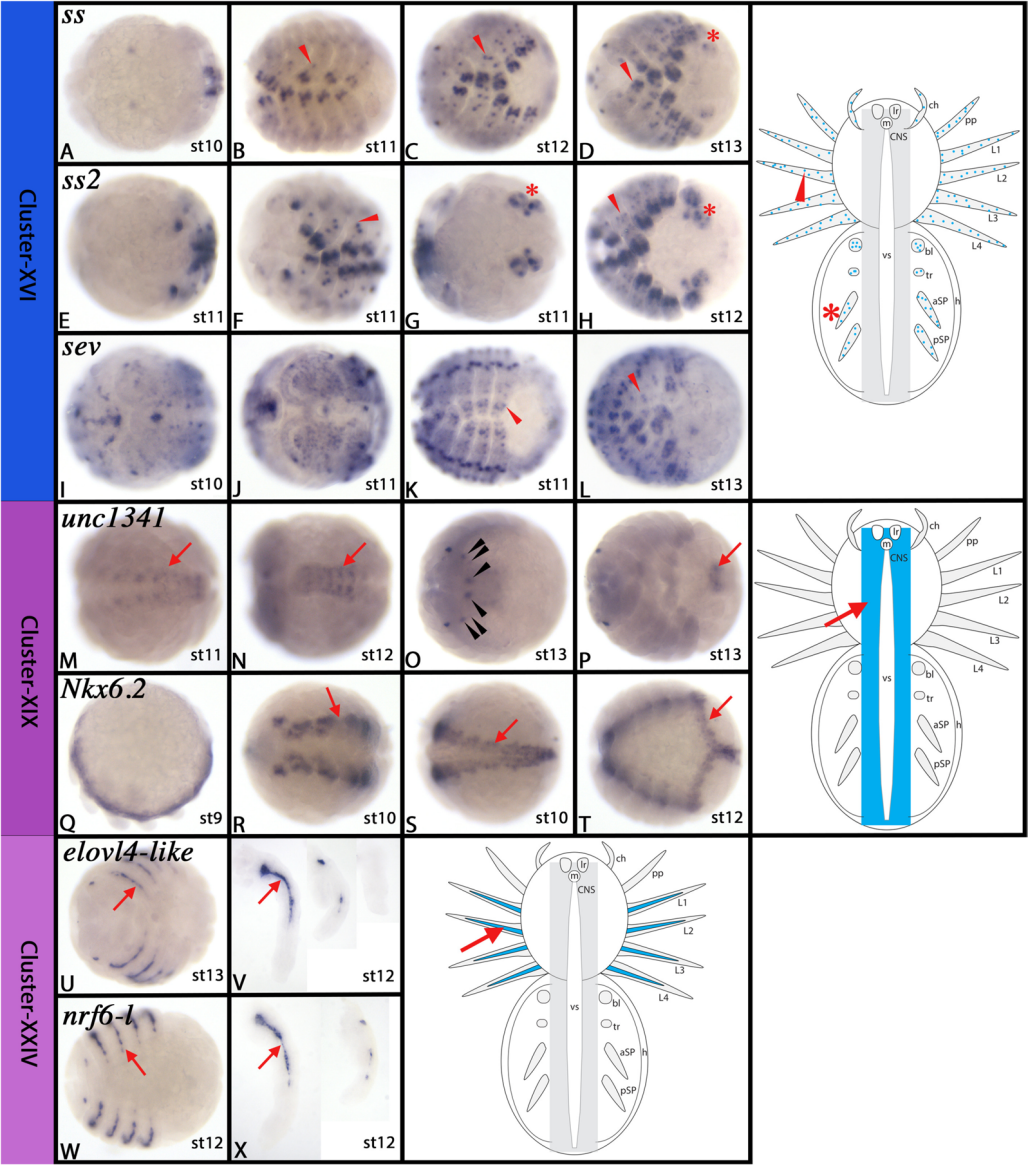
Gene expression of Cluster-XVI, -XIX, and -XXIV markers. In all panels, anterior is to the left. Panels A-M, P, R-U, and W represent ventral views. Panel N represents dorsal view. Panel O represents anterior view. Panel Q represents lateral view. Panels V and X show isolated walking legs (left), pedipalps (middle) and chelicerae (right), lateral views. Schematic drawings in the right column summarize the main gene expression characteristic for a given cell cluster (blue tissue). Arrows, arrowheads and asterisks in schematic drawings point to comparable expression as marked by the same symbol in the original photographs. Black arrowheads in panel O point to expression in the developing eyes. Abbreviations of gene names is indicated in the upper left corner. Developmental stages are indicated in the bottom right corner. For further information, see Supplementary Figs. 42–45, 52, 53, and 67–69, and Supplementary File 6. For abbreviations used in schematic drawings see legend of Fig. 4

Two top markers of this cluster represent ohnologs of the aryl hydrocarbon receptor *spineless* (*ss*) (LOC107454134, LOC107457395). In *Drosophila*, *ss* causes the transformation of the distal antenna into a distal leg [24, 44], but beyond that *ss* also is involved in sensory neuron development [40, 100, 116]. Both functions appear to be conserved in other insects [182]. Other top markers of this cluster are a *VEGF receptor like* gene (LOC107454058), *eagle* (LOC107450205), *sevenless* (*sev*) (LOC107441450) and *Pax2* (LOC107444558). In *Drosophila*, *VEGF receptor-like* is involved in mechanical nociception and normal axon branching, is expressed in sensory neurons, and overexpression leads to mechanical hypersensitivity [108, 120]. *eagle* represents a steroid receptor that is *inter alia* involved in nervous system development [69, 121]. *Drosophila sev* is a well-known factor of photoreceptor specification (reviewed in e.g. [183]. Finally, *Pax2* has been shown to be expressed in the developing peripheral nervous system of *Parasteatoda* [79]. In summary, this suggests that cells of Cluster-XVI represent the developing peripheral nervous system. *In-situ* hybridization experiments of the aforementioned genes supports this suggestion as they are all expressed in distinct cells or groups of cells in the periphery of the developing embryo such as the appendages that display a large number of sensory structures (e.g. [11, 55, 79, 178] (Fig. 10A-L and Supplementary Figs. 42–45). This is further supported by two genes that have been investigated as top markers of other clusters, *unc-4 like* (LOC107444630) (Fig. 9c-f, cf. Cluster-XV) and the uncharacterized gene *unc6289* (LOC107436289) (Supplementary Fig. 23, cf. Cluster-VIII). Both are expressed in similar patterns in the appendages suggesting a function during peripheral/sensory nervous system development.

#### Cluster-XVII: The developing central nervous system (CNS) (8) – Early differentiating neurons

The top markers of Cluster-XVII are *inscuteable* (*insc*) (LOC107457283 & LOC122271737), *nervous fingers* (*nerfin*) (LOC107446363), *prospero* (*pros*) (LOC107448306), and (less high-ranked) *brain tumor* (*brat*) (LOC107438139). *insc* is a neural precursor gene in *Drosophila* that is involved in defining neuroblast identity [5, 104]. Beyond that, *insc* is involved in controlling asymmetric cell division during nervous system development where it acts via* miranda* (*mira*) upstream of *pros* [76, 169]. Interestingly, *pros*, another confirmed factor of spider and arthropod nervous system development [23, 101, 185, 193], is one of the highest-ranking markers of Cluster-XVII as well. In *Drosophila*, *pros* functions as a factor of early neural differentiation by repressing neural stem cell markers such as *snail* (*sna*) (e.g. [200]. *brat* is involved in the regulation and distinction of intermediate progenitor cells from mitotically active neuroblasts, again interacting with the scaffolding protein *mira* [18]. Finally, *nerfin* prevents reversion and dedifferentiation of neurons into neural stem cells [53, 196]. *In-situ* hybridization of *insc*, *nerfin*, *pros* (in *Cupiennius salei*)*,* and *brat* reveals relatively early expression in the developing CNS suggesting that the cells represented by Cluster-XVII are early differentiating neurons (Fig. 5Y-f and Supplementary Figs. 46–48).

#### Cluster-XVIII: Ectoderm patterning of developing appendages (6) – EMT-like processes in the appendage epithelium

Among the top markers are *papilin-like* (LOC122270011), a paralog of the aforementioned *papilin* (LOC107447945) (cf. Cluster-VII), *integrin alpha-PS1* (*PS1*) (LOC107454271) (cf. Cluster-V), and *nord* (LOC107451942).

*papilin-like* is an extracellular matrix protein encoding gene that is a likely factor of basement membrane development and cell movement (reviewed in [48]. Comparing the expression patterns of *papilin-like* and *papilin* in *Parasteatoda* (cf. Cluster-VII) reveals only little similarity (Figs. 6M-O and 8D-F). This suggests that these two paralogs have undergone neo- or sub-functionalization, and thus represent markers of different but related cell clusters (clusters XVIII and VII respectively). Unlike *PS2* that is expressed in the visceral mesoderm (discussed above,cf. Cluster-VII), *PS1* is expressed in epithelial cells and mediates cell migration [189]. Notably, a number of laminin-subunit encoding genes (LOC107448892, LOC107452433), the putative ligands of the integrins [67, 127], and a collagen gene (LOC107442289), an interaction partner of *papillin* [97], are also among the strongest markers of Cluster-XVIII, and so is the extracellular matrix (ECM) protein encoding gene *nord* (LOC107451942) [198]. The genetic fingerprint of Cluster-XVIII is thus very similar to that of Cluster-VII, suggesting that its cells are actively involved in cell migration and thus likely in EMT-related processes.

*In-situ* hybridization of Cluster-XVIII marker genes, however, is not conclusive. All investigated top markers appear to have quite complex and in many aspects different expression patterns, and also the markers of Cluster-XVIII that have been studied previously in *Parasteatoda* such as *Wnt16* (LOC107457243) [87], *dally* (LOC107446074) [72], *FoxB* (LOC107443349) [65], and *hex1* (cf. Cluster-I) show a variety of different expression pattern (Fig. 6M-R and Supplementary Figs. 49–51). What these genes have in common, however, is expression in the developing appendages. It is thus likely that Cluster-XVIII represents cells that undergo an EMT-like process in the developing appendages.

#### Cluster-XIX: The developing central nervous system (CNS) (9) – The sensory nervous system of the head

Many of the top markers of Cluster-XIX have previously been studied in *Parasteatoda*. Among those are *irx-4* (LOC107456088) [110], *six3* (LOC107436457) [111, 157], *orthodenticle* (*otd/otx*) (LOC107457564) [163] and *eyes-absent* (*eya*) (LOC107452693) [163]. Although these genes are predominantly expressed in the developing nervous system of the head, including the developing eyes, they are also expressed (albeit at a lower level) in the developing CNS of the trunk [110, 157, 163]. Expression data of *irx-4* in later developmental stages are not shown in Leite et al. [110], but the putative homologs of *irx-4* in *Drosophila*, *araucan* (*ara*) and *caupolican* (*caup*) both are expressed in the sensory nervous system of the head including the developing eyes (e.g. [56, 147]. The second highest-ranked marker of this cluster is a *scavenger receptor class B member 1-like* gene (LOC122271437) that shows weak sequence similarity with *Drosophila croquemort* and *Sensory neuron membrane protein 1* (*Snmp-1*), a gene that is involved in the function of sensory pheromone receptors [91]. We investigated the embryonic expression of the uncharacterized gene *unc1341* (LOC122271341) and the homeobox gene *Nkx6.2* (LOC107450777) (cf. Cluster-VI) and show that both genes are expressed in the CNS including part of the most anterior region of the CNS (Fig. 10M-T and Supplementary Figs. 52 and 53). Notably, within the developing brain, *unc1341* is specifically expressed in the developing eyes (Fig. 10M-P). Cells representing Cluster-XIX thus clearly contribute to the developing CNS, possibly representing developing sensory structures in the head.

#### Cluster-XX: The developing heart

The top marker of this cluster is a *ryanodine receptor* (*ryr*) (LOC107450496) encoding gene that is typically found in muscle tissue (e.g. [63, 180]. The second-best marker is a *lethal 2* like gene (LOC107453784) and the third-best marker is *rho-associated protein kinase 2* (*rapk2*) (LOC107453369). Other high-ranked markers are two *titin/twitchin/bent* ohnologs (LOC107453137, LOC107453633), *filamin* (LOC107438849), *myosin heavy chain* (LOC107457063), *myocardin-related transcription factor* (LOC107443971) and *kon-tiki* (*kon*) (LOC107436488), all of which are involved in muscle and heart development (e.g. [105, 123, 133, 161, 202]. A lower-ranked marker, *myocyte enhancer factor 2* (*Mef2*) (LOC107445920) is also expressed in the developing heart of *Parasteatoda* [111] and another spider [80]. *In-silico* analysis thus clearly suggests that Cluster-XX cells represent developing muscle tissue, including the developing heart. We show by *in-situ* hybridization analysis that all three previously unstudied top markers also are expressed almost exclusively in the heart, strongly suggesting that Cluster-XX cells indeed represent the developing heart (Fig. 8G-L and Supplementary Figs. 54–56).

#### Cluster-XXI: Midgut development, yolk metabolism, hematopoiesis, and immune response

Among the top markers of Cluster-XXI are few previously investigated genes. The top markers are an *aquaporin-7-like* (*aqp7l*) gene (LOC107457171), the uncharacterized gene *unc8180* (LOC107448180), and a *Na*^+^*/K*^+^ *transporting ATPase subunit* (*NaK-t-ATPase*) (LOC107456567). The majority of markers appear to represent enzymes (e.g. *alkaline phosphatase* (LOC107438694) and *snake venom 5’-nucleotidase* (LOC107439410)), transporters (e.g. the aforementioned *NaK-t-ATPase* and *organic cation transporter protein* (LOC107457207)), and channel-forming proteins (e.g. the aforementioned aquaporin gene and *apolipophorin* (LOC107450568)), suggesting that these cells are involved in yolk metabolism and uptake. Another group of Cluster-XXI marker genes is involved in the vertebrate immune response (e.g. the aforementioned *snake venom 5’-nucleotidase, equilibrative nucleoside transporter 1* (LOC107445032) and *venom phosphodiesterase 2* (LOC107442458)) suggesting that this may be another function of Cluster-XXI cells (e.g. [74, 95, 159]. Indeed, in arthropods, the so-called extraembryonic tissues (sensu lato) are involved in providing immune response (recently reviewed in e.g. [143, 184]. *In-situ* hybridization shows that the three top markers of this cluster all exclusively are expressed in cells underlying the germ band (and possibly also the dorsal field and ventral sulcus), and thus the cells/tissue that connects the developing embryo (sensu stricto) with the yolk (Fig. 8M-R and Supplementary Fig. 57–59). Interestingly, we also find a midgut marker of *Parasteatoda* in this cluster, the GATA transcription factor *serpent* (*srp*) (LOC107456523), supporting that these cells may also contribute to midgut development that appears to go hand in hand with nutrition uptake in spiders [46]. Beyond that, hematopoiesis, a process that is closely linked with innate immune defence, depends on the interplay of the aforementioned GATA transcription factor *srp* with the ‘friend of GATA’ factor *U-shaped* (*Ush*) (e.g. [49, 50, 152], another unique marker of this cell cluster (LOC107440842).

#### Cluster-XXII: The stomodaeum and the ventral midline

The top gene markers of this cluster are a number of uncharacterized genes (e.g. *unc 5848* (LOC107445848), *unc3843* (LOC107453843), *unc5174* (LOC107455174) and *atrial natriuretic peptide-converting enzyme* (*anapece*) (LOC107455346). The only previously investigated spider genes that represent markers of Cluster-XXII are the two forkhead domain transcription factors *FoxC*/*crocodile* (*croc*) (LOC107456536) and *FoxA*/*forkhead* (*fkh*) (LOC107452746), *six3* (LOC107450741), and *visual system homeobox* (*vsx*) (LOC122268388). In *Parasteatoda* and other spiders, these genes are expressed in the stomodaeum and along the split ventral midline [84, 89, 111, 163]. Expression of *unc5848*, *unc3843*, and *anapece*, however, shows that these top markers are expressed exclusively in the stomodaeum, or strongly in the stomodaeum and faintly at the edges of the split ventral midline (Fig. 6S-V and Supplementary Figs. 60–63). It appears thus that Cluster-XXII predominantly represents cells of the stomodaeum and possibly also cells along the ventral midline.

#### Cluster-XXIII: Midgut development and yolk metabolism

Cluster-XXIII forms a well-separated small group of cells. The best marker of this cluster is the uncharacterized gene *unc7981* (LOC107437981). The second-best marker is the *Na*^+^*/H*^+^*-exchanger beta* (*nhe2*) (LOC107452408). This gene is expressed and functions in the epithelium of the mammalian gut (e.g. [33, 42]. To the best of our knowledge, there are no data on the expression or function of this gene in any arthropod species. Other markers of this cluster are the *Na-dependent phosphate transporter 1A* (*PiT1-like*) (LOC107453365) and the *multidrug resistance-associated protein 1* (*mrp1*) (LOC107440407). The former gene is involved in the uptake of inorganic phosphate, displays kidney functions, and is often expressed in the intestine (e.g. [68, 131]. *Mrp1* represents a universal transporter, including the transport of lipid derivatives (reviewed in [31, 32]. *In-silico* analysis thus suggests that these cells are involved in metabolic processes, possibly including the uptake of nutrition (yolk metabolism), and the development of the midgut, similar to the predicted functions of Cluster-XXI cells. Detection of gene expression supports this assumption as all of these genes are expressed exclusively in cells of (or underneath) the dorsal field, underneath the embryo proper, and in the tail region that appears to be a key-connective tissue between the yolk and the embryo proper (Fig. 8S-X and Supplementary Fig. 64–66) (cf. Cluster-XXI). The fact that Cluster-XXI and -XXIII cells are separated into two clusters suggest that they represent different cell populations of this tissue. In this context, it is interesting to note that one endodermal midgut gene, *serpent* (*srp*) (LOC107456523), is expressed in Cluster-XXI cells, but another midgut marker, *hepatocyte nuclear factor 4* (*hnf4*) (LOC107439273) is expressed in Cluster-XXIII cells [46].

#### Cluster-XXIV: A mini-cluster consisting of only 5 cells that suggests a function in leg-innervation

Some of the top markers of this cluster are *RYamide receptor-like* (LOC107443440), *elongation of very long chain fatty acids protein 4-like* (*elovl4-l*) (LOC122269995), *nose resistant to fluoxetine protein 6-like* (*nrf6-l*) (LOC107448146) and *prostaglandin reductase-3* (*ptgr3*) (LOC107457101).

*In-silico* analysis of these genes is difficult because there is little or no information about the function of these genes in *Drosophila* and other arthropods. Only the *RYamide receptor-like* gene hints to a function in nervous system development. RYamide peptides have been identified in at least arthropods and tardigrades [26, 103], and represent the orthologs of lophotrochozoan *Luqin* genes [90]. RYamides/Luqins are involved in a wide range of physiological function, including feeding behaviour and locomotion (e.g. [115]. We failed to PCR-amplify *RYamide receptor-like,* but the other aforementioned markers all are expressed exclusively inside the developing legs, and with lower intensity the developing pedipalps (Fig. 10U-X and Supplementary Figs. 67–69), suggesting a function in locomotion, possibly innervation of these appendages.

### Mega-clusters and sequencing depth

Overall, the achieved SCS data appear to be of acceptable quality, i.e. most of the predicted cell clusters indeed represent specific tissues of the developing spider embryos such as the developing heart, the ventral sulcus or the peripheral nervous system (PNS). These are also the clusters that are most clearly separated from other clusters, and thus are most well-defined. Other clusters appear to be sub-clusters of larger clusters, often part of what we would like to call mega-clusters (Mega-Cluster-A (Ectoderm patterning of developing appendages; clusters I, II, IV, XIII, XV, and XVIII), Mega-Cluster-B (The developing central nervous system (CNS); clusters III, V, VI, VIII, X, XI, XII, XIV, XVI, XVII, XIX, and XXIV), and Mega-Cluster-C (Epithelial-to-mesenchymal transition (EMT)); clusters V, VII, XIV, and XVIII); note that some clusters such as Cluster-XVIII appear to be part of two mega clusters (summarized in Table 2). Naturally, the underlying data that subdivide those closely-related and physically connected (in the UMAP) clusters is less strong (i.e. there are less many markers that are specifically expressed in cells of each given cluster, and that are expressed in more *vs* less cells of a given cluster). The mega-clusters could represent the actual situation in developing embryos in terms that many related cell types or developmental stages of the same cell type indeed express very similar sets of marker genes. Since our SCS data stem from embryos and thus developing tissues and differentiating cell types, this assumption is not unlikely.

**Table 2:** Cluster overview

Cluster	Mega Cluster	Title	Tissue
I	A	Dorsal tissue, and ectoderm patterning of developing appendages (1)	Ectoderm
II	A	Ectoderm patterning of developing appendages (2)	Ectoderm
III	B	The developing central nervous system (CNS) (1) – Differentiating and differentiated neurons (1)	CNS
IV	A	Ectoderm patterning of developing appendages (3)	Ectoderm
V	B/C	The developing central nervous system (CNS) (2) – EMT-like processes in neural precursor determination?	CNS
VI	B	The developing central nervous system (CNS) (3)—Early differentiating neural cells (1)	CNS
VII	C	The developing mesoderm (1) – EMT-like processes in visceral mesoderm development?	EMT (Mesoderm)
VIII	B	The developing central nervous system (CNS) (4)—Early differentiating neurons?	CNS
IX	–-	Opisthosomal appendages	Ectoderm
X	B	The developing central nervous system (CNS) (5): Early differentiating neural cells (2)	CNS
XI	B	The developing central nervous system (CNS) (6)—Neural precursors	CNS
XII	B	The developing central nervous system (CNS) (7) – Differentiating and differentiated neurons (2)	CNS
XIII	A	Ectoderm patterning of developing appendages (4)	Ectoderm
XIV	B/C	The ventral sulcus (VS)	Ventral Sulcus
XV	A	Ectoderm patterning of developing appendages (5)	Ectoderm
XVI	B	The peripheral nervous system (PNS)	PNS
XVII	B	The developing central nervous system (CNS) (8)—Early differentiating neurons	CNS
XVIII	A/C	Ectoderm patterning of developing appendages (6): EMT-like processes in the appendage epithelium	EMT (Ectoderm)
XIX	B	The developing central nervous system (CNS) (9) – The sensory nervous system of the head	CNS
XX	–-	The developing heart	Mesoderm
XXI		Midgut development, yolk metabolism, hematopoiesis, and immune response	Yolk + Blood cells /Endoderm
XXII	–-	The stomodaeum and the ventral midline	Ectoderm
XXIII	–-	Midgut development and yolk metabolism	Yolk cells /Endoderm
XXIV	B	A mini-cluster consisting of only 5 cells that suggests a function in leg-innervation	PNS

Mega Cluster A: Ectoderm patterning of developing appendages

Mega Cluster B: The developing central nervous system (CNS)

Mega Cluster C: Epithelial-to-mesenchymal transition (EMT)

Alternatively, however, mega-clusters may represent “artefacts” as a result of the shortcomings of our analysis. These cells may indeed be much more different from each another, and thus the corresponding clusters could be much more defined than shown in our analysis. A possible reason for such a scenario could be the relatively low sequencing saturation in our analysis (ca. 11%). Low sequencing depth could have let to the lack of detection of more specific, but less numerous transcripts.

### A bias towards ectodermal tissues?

We recognized that the majority of all detected tissues, and thus dissociated and captured cells represent ectodermal derivatives, while less mesodermal, and few endodermal cells were detected in our analysis. This could either represent the natural correlation of ectodermal *vs* mesodermal *vs* endodermal cells in the developing spider embryo at the investigated stages, or it could be a bias introduced by our methodology, i.e. dissociation, cell capture and downstream analysis. Additionally, we likely removed a fraction of the cells that cover the yolk outside the embryo proper (i.e. cells of the ventral sulcus and the dorsal field) prior to dissociation and cell capture.

We assume, however, that ectodermal cells represent the majority of cells in embryos of the stages 10–12, followed by mesodermal cells and finally endodermal cells. We base this assumption on the fact that most tissues indeed are ectodermal, followed by mesodermal tissues/cells. This has also been shown previously by the expression profiles of typical ectodermal, mesodermal and endodermal markers (e.g. [46, 87, 88, 110, 111]. The study by Feitosa et al. [46], for example, has shown that only relatively few cells of the so called dorsal extraembryonic tissue (the dorsal field in this study) likely contribute to the later (endodermal) midgut [46]. Therefore, we believe that the distribution of captured ectodermal, mesodermal and endodermal cells likely represents the true distribution of these cell types in developing spider embryos of stage 10–12. The recent SCS-study by Leite et al. [111] on earlier stages of *Parasteatoda* further support this assumption.

### Whole genome duplication in the spider: How reliable is “in-silico” analysis?

One part of the analysis of cell markers is what we call “*in-silico*” analysis, the comparison of known data from other arthropods, usually the vinegar fly *Drosophila melanogaster*. One potential problem of this kind of comparative analysis, however, is the fact that many genes are duplicated in *Parasteatoda* (e.g. [87, 110, 166], which likely represents the result of a whole genome duplication (WGD) in this species that dates back to a WGD in the last common ancestor of Arachnopulmonata (e.g. [137, 166]. *In-silico* analysis therefore often compares one (of two) paralogs/ohnologs of a given marker gene of a particular cell cluster in *Parasteatoda* with the one ortholog in *Drosophila* (or other previously investigated organisms). Only in a few cases, both *Parasteatoda* ohnologs of a given gene (if present as two ohnologs) represent markers of the same cell cluster and are ranked similarly as markers of this cluster. Examples are the two *spineless* ohnologs in Cluster-XVI, or the two *delta* ohnologs in Cluster-VIII.

For all *Parasteatoda* genes, however, that are present in the form of two ohnologs and that are not both markers of a given cell cluster, this may cause a problem. We know that many ohnologs have undergone sub-functionalization and neo-functionalization. It is thus possible that a marker, if this particular *Parasteatoda* ohnolog has been the subject of neo-functionalization, may have a completely different function than its ortholog in other organisms. Likewise, spatial sub-functionalization (expression in different tissues) and temporal sub-functionalization (expression in the same tissue/cell type at different time points during development) may lead to misinterpretation of the function of a given marker gene. This problem can only be addressed beyond doubt by comprehensive WISH analysis of every considered marker (as partially done in this study).

### Cell-type vs developmental stage of a cell

Interpretation of the obtained data must consider that an identified cell cluster may either represent a certain cell type (as defined by a cell-type specific genetic fingerprint), or a developmental stage of a certain cell type that therefore may also be represented by other, typically accompanying cell cluster(s). Possible examples are represented by the mega-clusters. Some clusters of Mega-Cluster B (e.g. clusters VI, VIII, X and XI) all harbour cells of the early developing CNS, representing either different (differentiating) cell types, different developmental stages of the same general cell type (that at all times during their development are represented by a more or less stable and specific genetic fingerprint), or cells that represent the same future cell type and/or developmental stage (of that cell type), but show variation (heterogeneity) in their expression profile (e.g. [29]. Indeed, suggestions have been made how to overcome this apparent problem [8], but we currently do not have the tools to apply them to our current data set. Thus, although we can make predictions on this matter, we do not believe that it is currently possible to distinguish the one from the other beyond considerable doubt.

### Our data compared to previously published SCS-projects in *Parasteatoda tepidariorum*

Two previous studies on SCS in *Parasteatoda* embryos covered the developmental stages 5 [2] and stages 7–9 [111]. At stage 5, a germ disc and a cumulus have formed. The cumulus represents later endomesodermal cells and the disc represents the ectodermal tissues of the developing germ band. Additional endodermal and mesodermal tissue come from the rim of the disc (e.g. [138, 197]. The centre of the disc represents the later posterior of the embryo and its segment addition zone (SAZ), and the periphery of the disc represents the anterior of the later germ band (e.g. [2]. Cells from outside the disc represent the yolk and part of the later-forming dorsal field (DF). At this point, the embryo consists of approximately 2000 cells representing mainly undifferentiated cells of the three germ layers [2]. Already at this stage of development, the genetic fingerprint of endodermal cells appears to be quite different from the ectoderm and mesoderm, the latter two which form clusters that are in close proximity to each another (i.e. having related genetic fingerprints) [2]. Interestingly, the study by Akiyama-Oda and colleagues presents two endodermal cell clusters, one single mesodermal cell cluster, and a much larger number of ectodermal cell clusters that represent different regions along the centre-to-periphery-axis of the disc [2].

The study by Leite et al. [111] targets the stages 7–9 each represented by separated dissociation and SCS experiments that were then combined into a single SCS-atlas. Given the more advanced developmental stages that are addressed in this study compared to the study by Akiyama-Oda et al. [2], there are considerably more and more distinct cell clusters [111]. Like in our study, Leite et al. [111] also identified 23 distinct cell clusters, but the nature of these clusters is in many cases quite different from the 23 cell clusters identified in our study. Therefore, it is not possible to directly compare the clusters recovered in Leite et al. [111] with the clusters that we identified in our study. Indeed, there are obvious explanations for these differences. One main difference between the two studies is the higher number of CNS-representing clusters in our analysis which may easiest be explained by the more differentiated (and differentiating) state of the central nervous system in stage 10–12 *vs* stage 7–9 embryos, where in the latter, development and differentiation of the CNS may only just have begun [111]. Some clusters identified by Leite et al. [111] like the segment addition zone (SAZ) and the segment maturation zone (SMZ) have not been identified in our study, most easily explained by the lack or miniaturization of such tissues in later stage embryos (e.g. [130]. On the other hand, Leite et al. [111] did not detect cells specific for the ventral sulcus (VS), simply because this structure has not yet formed in the developmental stages studied by them. Another eye-striking difference in the outcome of the previous studies compared with our study is the apparent lack of EMT-related cell types in their studies. This, so we believe, can also be explained by the stages of the sequenced embryos because main morphogenetic events may take place at later developmental stages and after the development of the complete AP axis, rather than early during the processes of axis formation, germ band formation, and segment addition. Finally, Leite et al. [111] also identify a cluster representing putative stem cells, but we do not find a corresponding cell population in our study, most likely because the number of stem cells is low in the later and thus more differentiated developmental stages that we investigated. The paper by Leite et al. [111] uses Hox genes as regional markers and defines some of the recovered clusters according to the expression/distribution of Hox genes. In this way, they for example discriminate between the first two pairs of legs (L1 and L2) and the remaining legs, L3 and L4. Likewise they identify a cluster representing the pedipalp-bearing segment (or merely the developing pedipalp). We did not do this in our analysis because we believe that regional markers are potentially misguiding. It is unlikely, for example, that the cells that build L1 and L2 are fundamentally different than those involved in the development of L3 and L4, especially because these segments and their appendages all form from the early germ disc, and do not represent segments/appendages that are partially from the germ disc, and partially added sequentially from the SAZ. Using regional markers such as *labial* (*lab*) to discriminate the pedipalp-bearing segments from the leg-bearing segments, however, makes sense if the genes involved in pedipalp development indeed differ somewhat from those in leg development.

### Mega-Cluster-A: Ectoderm patterning of developing appendages

According to our data and subsequent analysis, six clusters clearly are associated with the ectoderm of developing appendages (i.e. clusters I, II, IV, IX, XIII, and XV). Although both *in-silico* analysis of known genes representing markers of these clusters and accompanying *in-situ* hybridization analysis show that these cells come from the ectoderm of developing appendages, characterization of each cluster turned out to be rather complicated. Firstly, the detected patterns in the appendages are complex and dynamic making it difficult to find common patterns of cells of a given cluster. Secondly, even top markers of certain clusters such as Cluster-XIII showed to be expressed in several tissues further complicating analysis. It is obviously necessary to further investigate these cell clusters, either by applying an improved SCS data set, or by more detailed *in-situ* hybridization studies either comparing marker gene expression in the appendages of exactly staged embryos, or applying double (or multi) staining. We assume that such data could reveal the interplay(s) of genes involved in appendage patterning.

An interesting finding of our study is the presence of multiple genes that are expressed like genes that are involved in joint formation, i.e. in neatly spaced rings along the proximal–distal (PD) axis of the appendages. The number of cell clusters, and the accompanying large number of markers, that are involved in PD-appendage patterning and (likely) also joint formation is remarkably high, possibly reflecting the complexity of the crucial process of leg-patterning and joint development. Our data thus provide further insight and the possibility to study multiple new genes that likely are involved in these processes.

Another interesting outcome of our study is the finding that Cluster-IX cells are apparently specific for the heavily modified opisthosomal appendages of spiders, the breathing organs (tracheae and book lungs) and the possibly most characteristic feature of spiders, the spinnerets. This may allow a comparison between the genetic networks involved in the developing of the more basal locomotory prosomal legs and the derived appendages of the opisthosoma.

### Mega-Cluster-B: The developing CNS

Several of the identified cell clusters represent cells of the developing CNS. Interestingly, the genetic fingerprint of these clusters also enables us to predict their specific identity beyond their mere involvement in nervous system development. We identify proneural genes predominantly in Cluster-XI, suggesting that these cells represent neural progenitors. The adjacent clusters VI and X both likely represent early differentiating neural cells based on the presence of the neurogenic *delta* genes, the proneural gene *ash* that in *Drosophila* is involved in neuroblast segregation (Cluster-X), and a cassette of nervous system cell cycle controlling factors that also block the action of the proneural gene *SoxN* found in Cluster-XI (neural progenitors). Adjacent to clusters VI and X lies Cluster-VIII which possibly represents early differentiating neuronal cells including neurons. Although the cluster is marked by a number of the aforementioned markers of clusters VI and X it also expresses markers such as *cph* that is involved in the early differentiation of neural cell types, and *cpo* that *inter alia* is involved in neuronal precursor development. Cells of Cluster-VIII thus likely represent a later step in nervous system development and differentiation than cells of clusters VI, X and XI. Closely connected to Cluster-VIII lies Cluster-XVII which, based on its genetic fingerprint, appears to represent early differentiating neurons: *insc* is involved in neuronal progenitor determination, *pros* inhibits neural stem cell development and initiates neuronal differentiation, *brat* defines intermediate neuronal progenitors from primary neuronal progenitors, and *nerfin* blocks reversion and dedifferentiation of neurons into neural precursors. In the periphery of the CNS mega-cluster we find two closely-connected clusters, Cluster-III and Cluster-XII, that very likely represent developing and/or mature neurons because they both express genes that are key-factors of neuron development, neuron differentiation, and neuronal pathfinding. Compared to Cluster-XII, Cluster-III expresses more neuron-specific markers such as the Dscams. It may thus be that Cluster-XII indeed represents mostly late developing neurons, and Cluster-III mostly mature neurons.

Remarkably, we can follow CNS development from neural progenitor determination (Cluster-XI), over the early differentiation of these cells (clusters VI, X and VIII) towards the determination of neuronal precursors and early developing neurons (Cluster-XVII) up to the late stages of neuron development and finally mature neurons (clusters III and XII).

A second branch of neuronal cells is represented by clusters XVI, XIX, XXIV, and possibly even XXII. Closest to clusters VI and X, the early differentiating neural cells, lies Cluster-XIX which we believe represents mainly developing sensory nerve cells of the head. Close by lies the mini-cluster Cluster-XXIV that only contains 5 cells. These cells, however, appear to be part of the appendage-innervation system. Cluster-XVI is represented by cells that express typical markers of the peripheral nervous system such as *Pax2*, *sev* and *eagle*. Finally, Cluster-XXII that represents cells of the developing stomodaeum (and ventral midline?) may also represent a neuronal cell type. While the developmental trajectory of Cluster-XI to Cluster-III appears to represent the development from neuronal precursors towards mature neurons, the possible trajectory of Cluster-XI to Cluster-XXII appears less straightforward and thus may need further investigation, probably with the help of an improved data set.

A third short trajectory of CNS-related clusters appears to be the connection of Cluster-XI to its neighbouring Cluster-V. Both clusters express *sna*, a gene that in *Drosophila* regulates the delamination of neuroblasts (neuronal precursors) and that is thus involved in EMT-like processes during neurogenesis [7]. Other genes of Cluster-V indeed are involved in EMT and the expression patterns of many markers of Cluster-V shows that they are not only expressed in the CNS but also various other tissues, likely as a result of the delamination and morphogenic movements of these cells. The trajectory from Cluster-XI to Cluster-V and the involvement of EMT-like processes in CNS development is thus closely related to other cell types such as mesodermal cells (Cluster-VII) or the ectoderm of the developing appendages (Cluster-XVIII) that share an EMT-related genetic fingerprint (discussed below).

### Mega-Cluster-C: Epithelial-to-mesenchymal transitions (EMTs)

Several of the identified gene clusters are marked by an EMT-related genetic fingerprint. The main difference between these cells is the underlying second genetic fingerprint that is for example neuronal (clusters V and XIV) (discussed above), mesodermal (Cluster-VII), or appendage-ectoderm specific (Cluster-XVIII). We believe that the reason for detecting a large number of cells that show an EMT-related genetic fingerprint is likely correlated with the relatively late developmental stages we investigated in this study. This goes in line with the other *Parasteatoda* SCS studies that investigate earlier developmental stages and that did not specifically identify cells that are undergoing EMT [2, 111]. Indeed, processes like CNS differentiation, the formation of neuronal networks, and the formation of the mesoderm and mesodermal organs such as the heart most certainly require EMT and morphogenic movement.

### Future perspectives

The recent SCS-based papers published on the development of *Parasteatoda* clearly highlight the interest in this model system and the interest in SCS data [2, 111], this study). In the future, the existing data including our own will, we hope, be supplemented and extended towards earlier as well as later developmental stages, including nymphs, juveniles and adults of both sexes, to develop a comprehensive overview over cell types and their developmental trajectories in this model spider. Detailed knowledge about the changes of the genetic fingerprints of developing cell types and the identification of definite cell types will significantly improve our knowledge about the development of spiders. This will include the identification of novel genes, and the untangling of new and conserved gene regulatory networks (GRNs). These data can then be used to study newly identified marker genes (or complete GRNs) in classic candidate gene approaches beyond spiders, possibly enabling us to reconstruct the development and evolution of Arthropoda as a whole. The current study aims to contribute to this goal.

### Supplementary Information

**Additional file 1.**

**Additional file 2.**

**Additional file 3.**

**Additional file 4.**

**Additional file 5.**

**Additional file 6.**

**Additional file 7.**

**Additional file 8.**

**Additional file 9.**

**Additional file 10.**

**Additional file 11.**

**Additional file 12.**

**Additional file 13.**

**Additional file 14.**

**Additional file 15.**

**Additional file 16.**

**Additional file 17.**

**Additional file 18.**

**Additional file 19.**

**Additional file 20.**

**Additional file 21.**

**Additional file 22.**

**Additional file 23.**

**Additional file 24.**

**Additional file 25.**

**Additional file 26.**

**Additional file 27.**

**Additional file 28.**

**Additional file 29.**

**Additional file 30.**

**Additional file 31.**

**Additional file 32.**

**Additional file 33.**

**Additional file 34.**

**Additional file 35.**

**Additional file 36.**

**Additional file 37.**

**Additional file 38.**

**Additional file 39.**

**Additional file 40.**

**Additional file 41.**

**Additional file 42.**

**Additional file 43.**

**Additional file 44.**

**Additional file 45.**

**Additional file 46.**

**Additional file 47.**

**Additional file 48.**

**Additional file 49.**

**Additional file 50.**

**Additional file 51.**

**Additional file 52.**

**Additional file 53.**

**Additional file 54.**

**Additional file 55.**

**Additional file 56.**

**Additional file 57.**

**Additional file 58.**

**Additional file 59.**

**Additional file 60.**

**Additional file 61.**

**Additional file 62.**

**Additional file 63.**

**Additional file 64.**

**Additional file 65.**

**Additional file 66.**

**Additional file 67.**

**Additional file 68.**

**Additional file 69.**

**Additional file 70.**

**Additional file 71.**

**Additional file 72.**

**Additional file 73.**

**Additional file 74.**

**Additional file 75.**

## Data Availability

The data generated and analysed during this study are included in this published article and its supplementary information files. Raw sequencing reads in fastq.gz format are available from the corresponding authors on reasonable request.
