# Supplementary figures and images for: Single-cell RNA sequencing of mid-to-late stage spider embryos: new insights into spider development
Source: BMC Genomics. 2024 Feb 7;25:150. doi: 10.1186/s12864-023-09898-x (PMC10848406; doi:10.1186/s12864-023-09898-x)

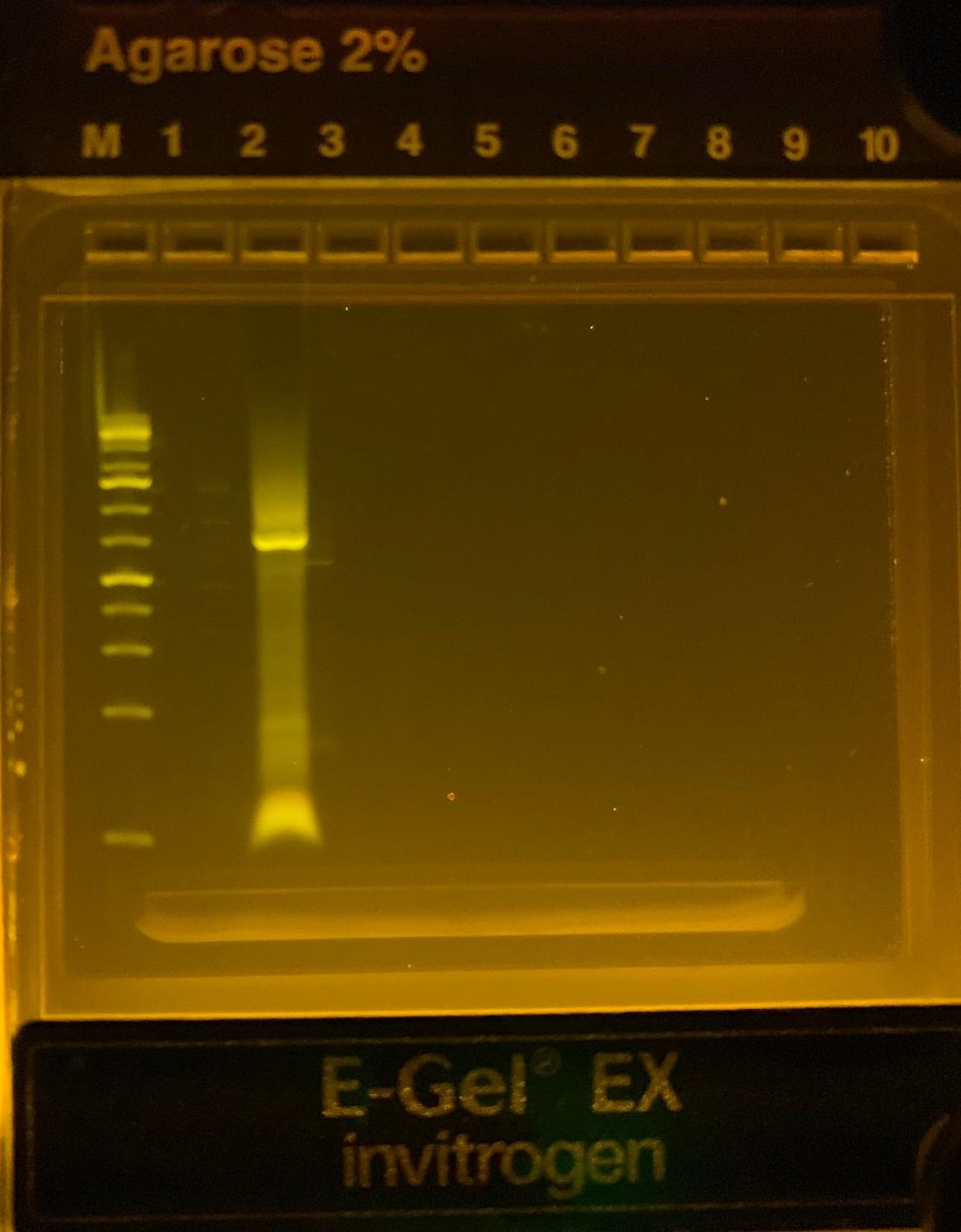

Supplement: Supplementary file 1 — Additional file 1. [file 12864_2023_9898_MOESM1_ESM.jpg]

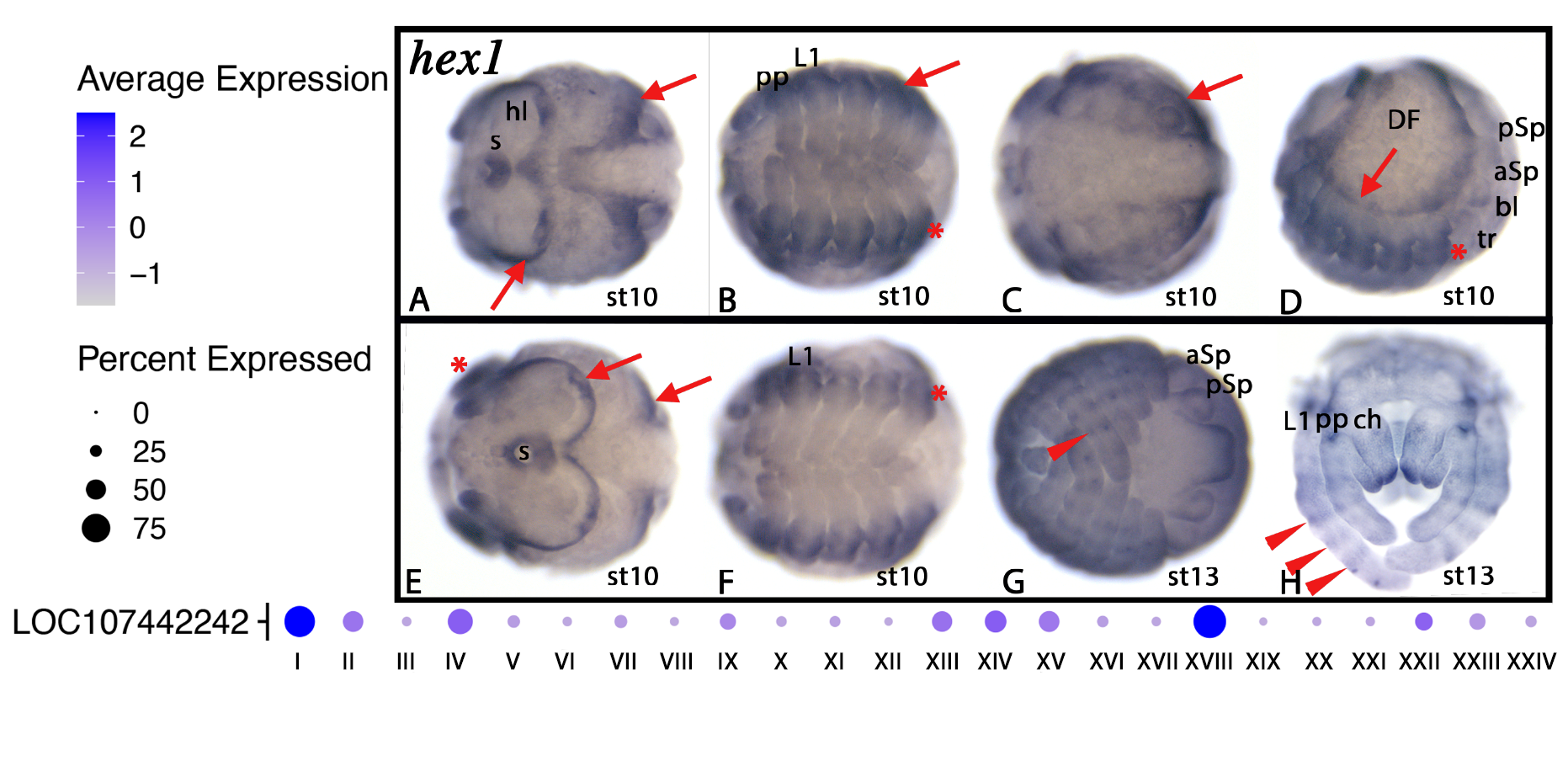

Supplement: Supplementary file 2 — Additional file 2. [file 12864_2023_9898_MOESM2_ESM.tif]

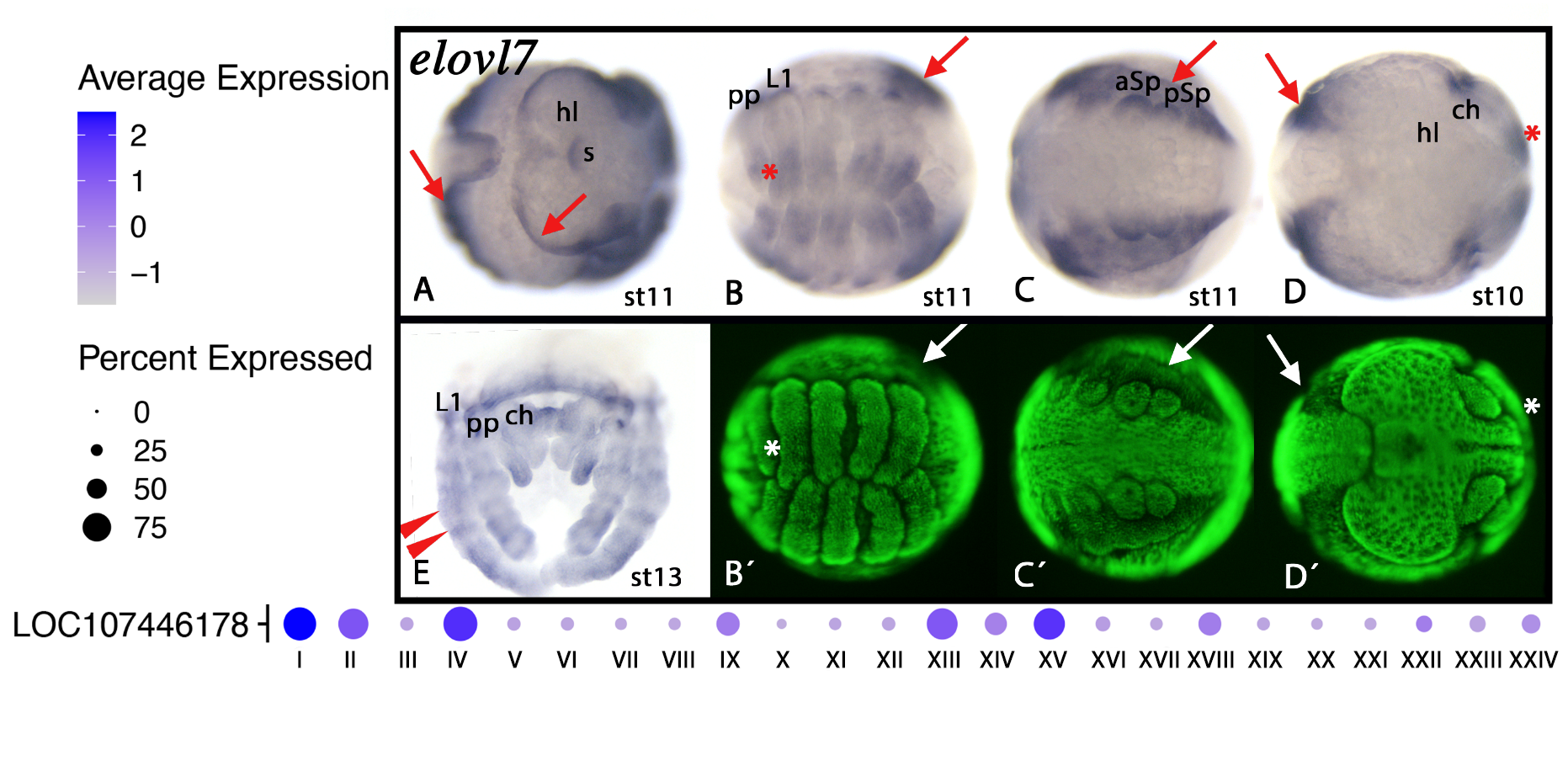

Supplement: Supplementary file 3 — Additional file 3. [file 12864_2023_9898_MOESM3_ESM.tif]

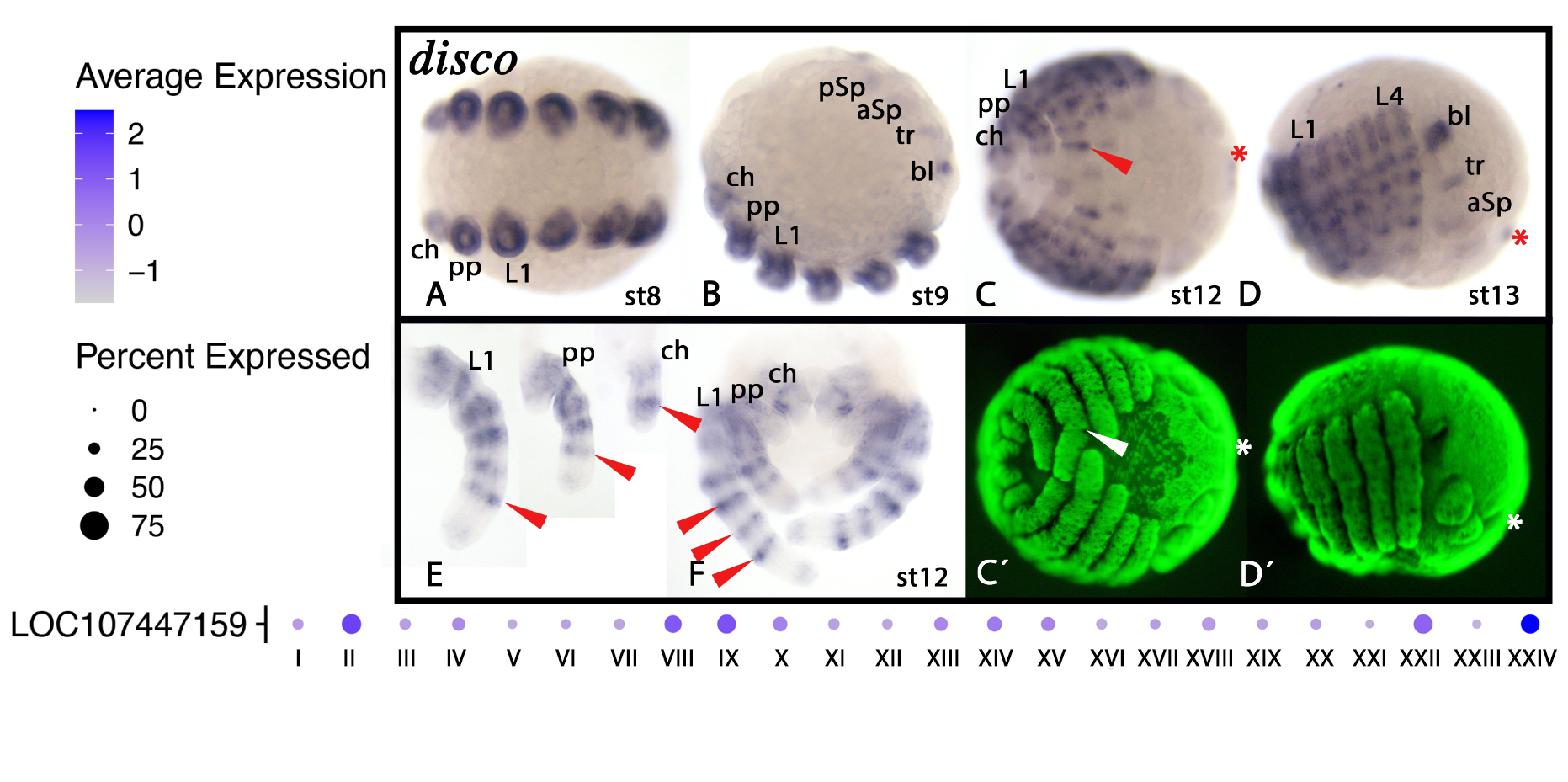

Supplement: Supplementary file 4 — Additional file 4. [file 12864_2023_9898_MOESM4_ESM.tif]

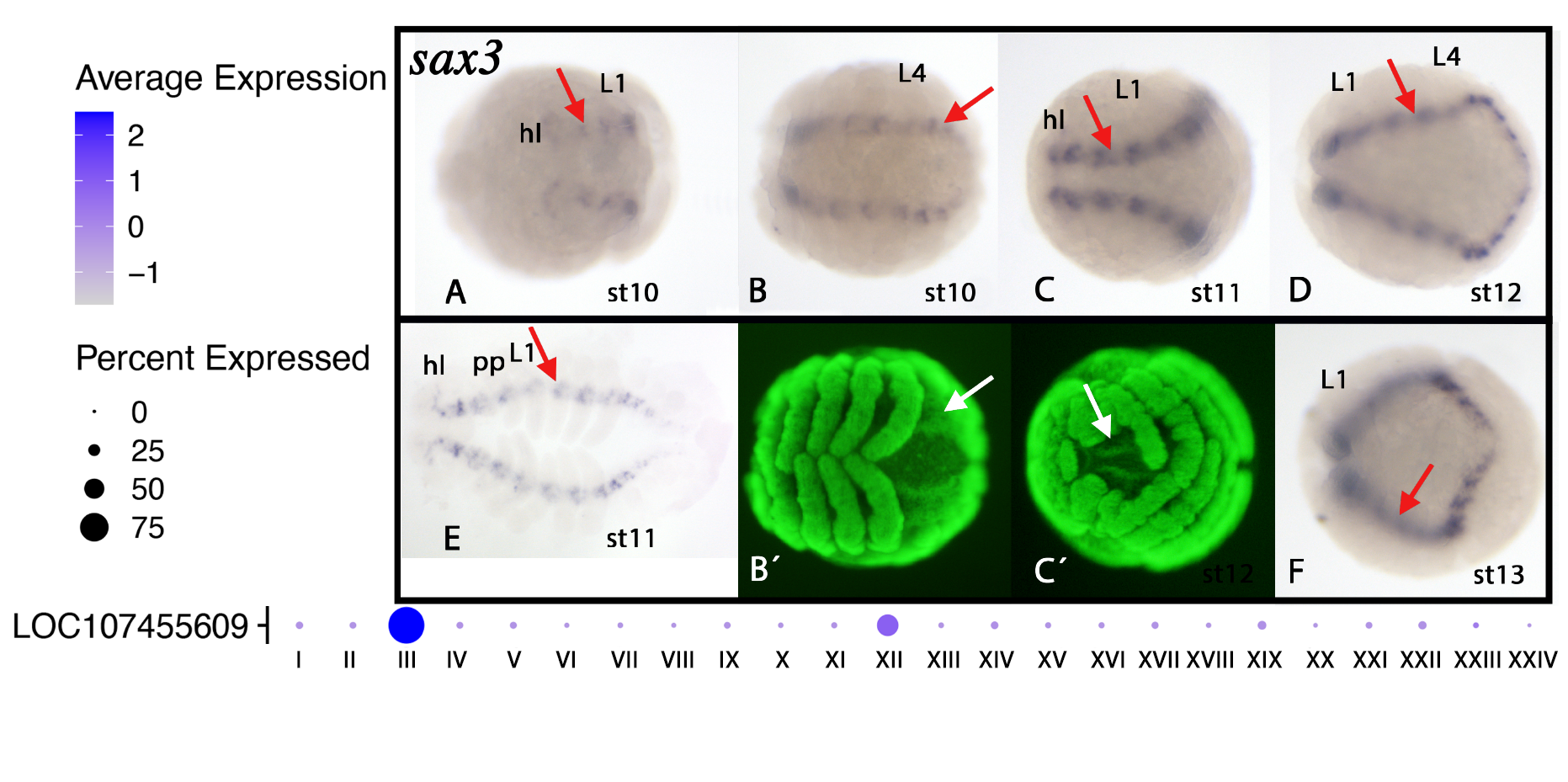

Supplement: Supplementary file 5 — Additional file 5. [file 12864_2023_9898_MOESM5_ESM.tif]

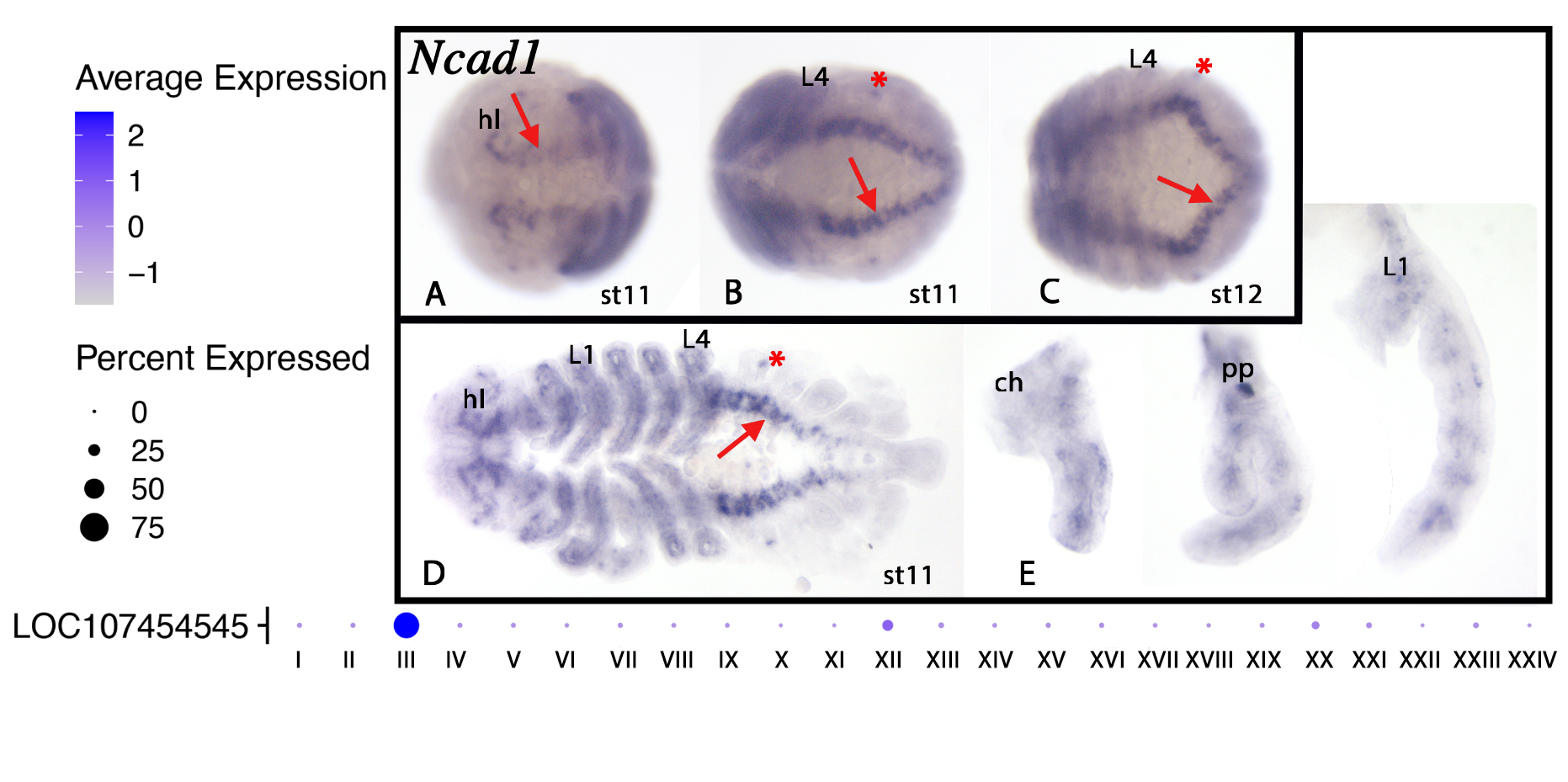

Supplement: Supplementary file 6 — Additional file 6. [file 12864_2023_9898_MOESM6_ESM.tif]

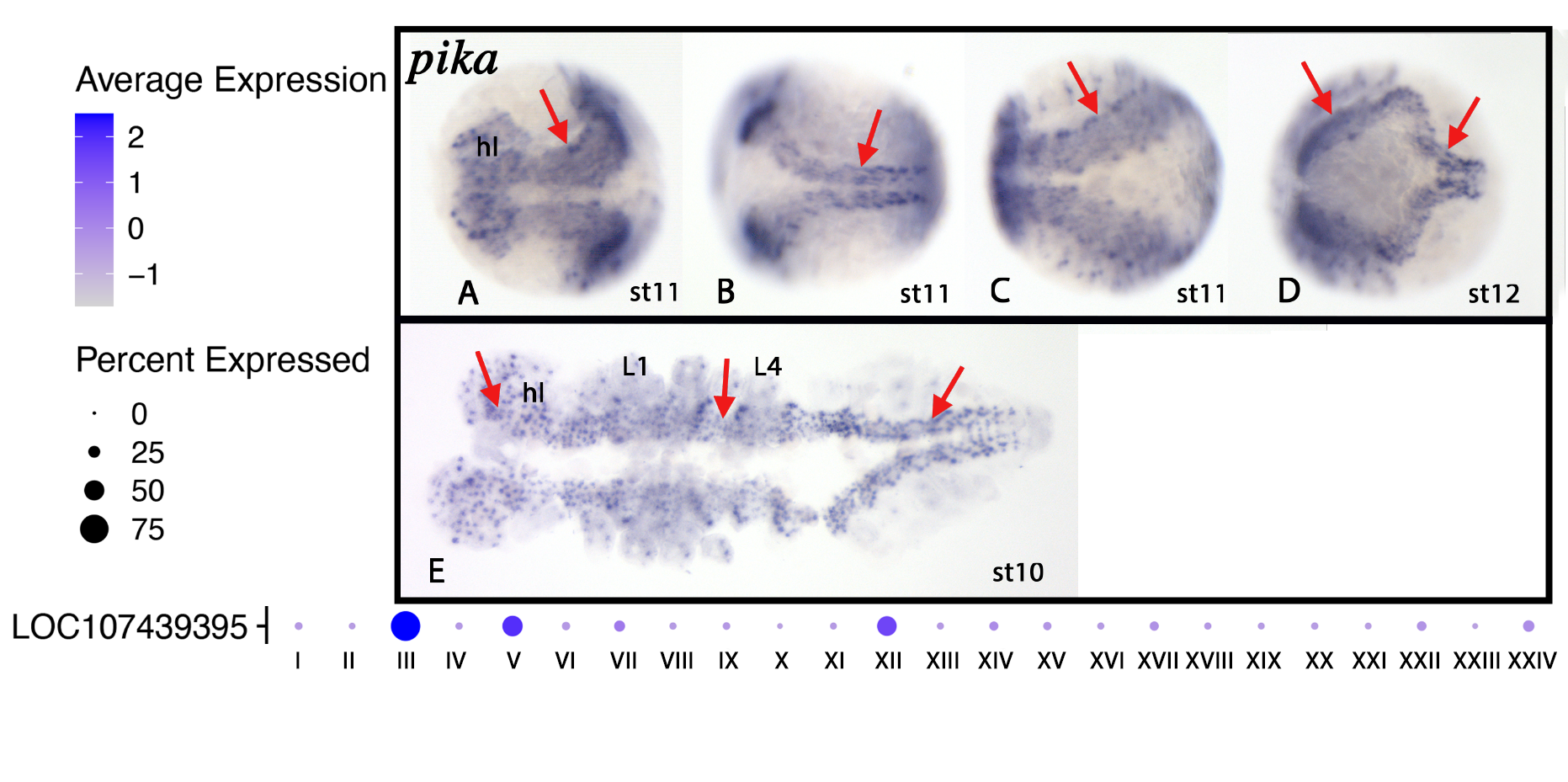

Supplement: Supplementary file 7 — Additional file 7. [file 12864_2023_9898_MOESM7_ESM.tif]

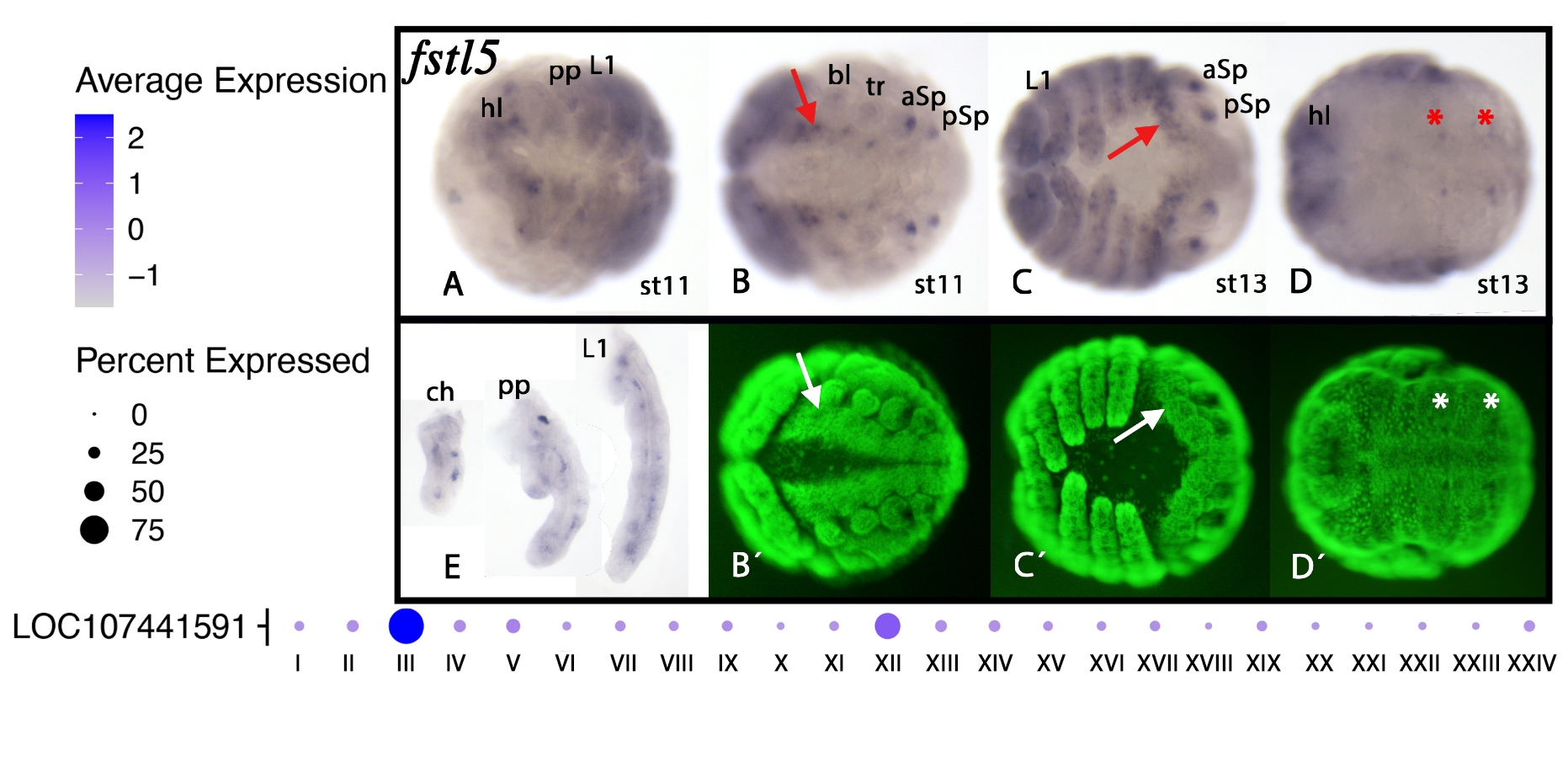

Supplement: Supplementary file 8 — Additional file 8. [file 12864_2023_9898_MOESM8_ESM.tif]

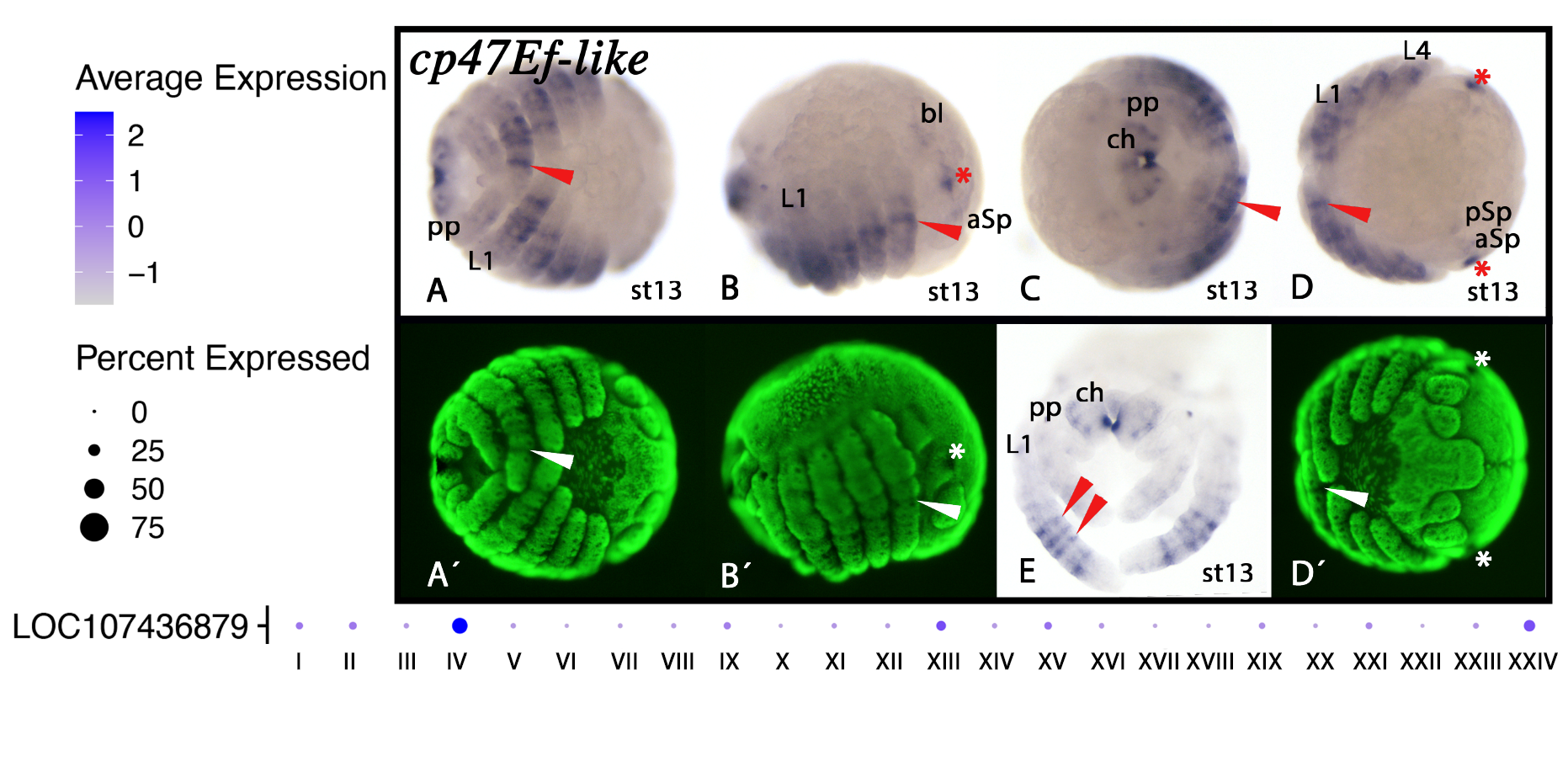

Supplement: Supplementary file 9 — Additional file 9. [file 12864_2023_9898_MOESM9_ESM.tif]

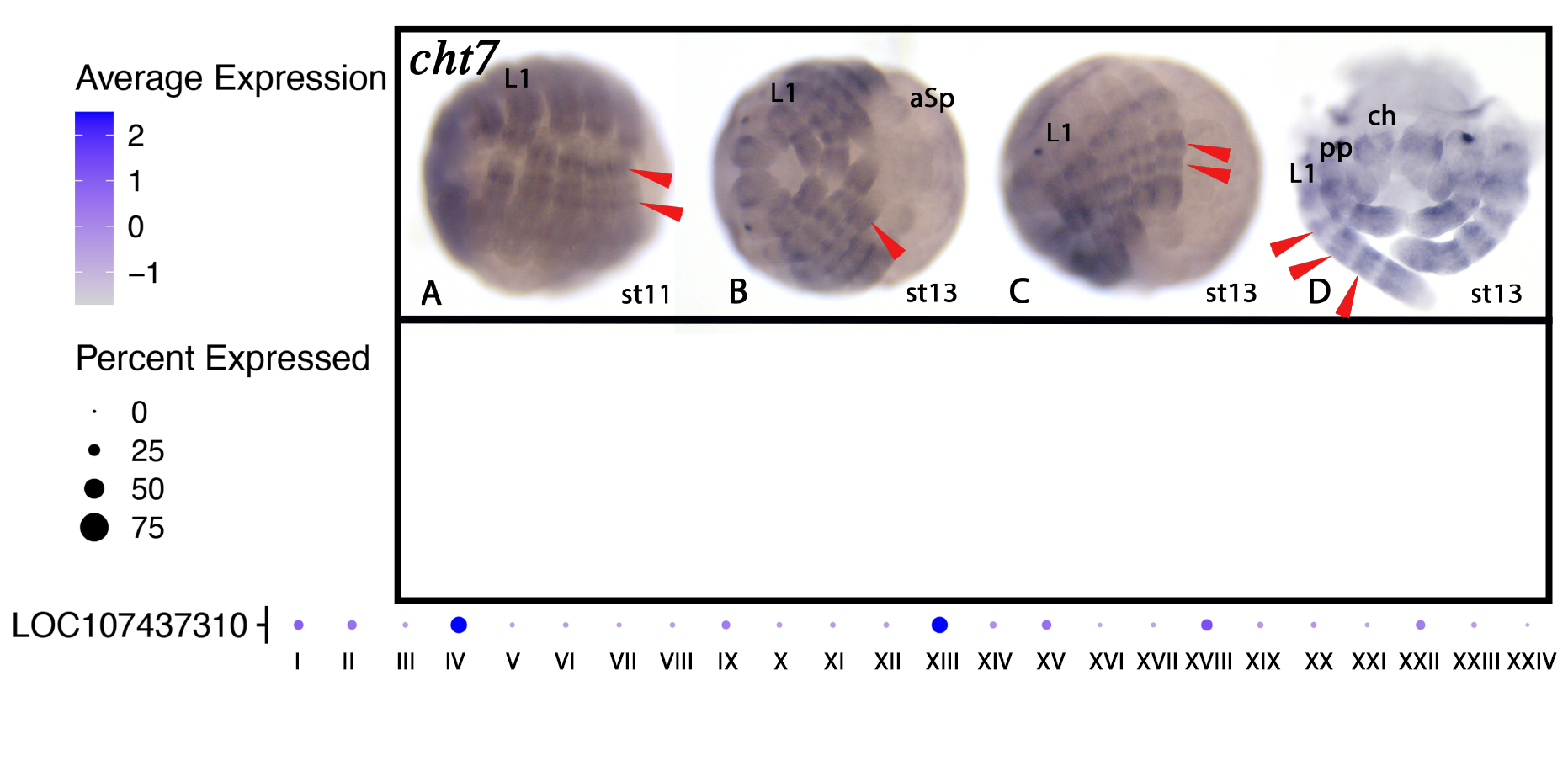

Supplement: Supplementary file 10 — Additional file 10. [file 12864_2023_9898_MOESM10_ESM.tif]

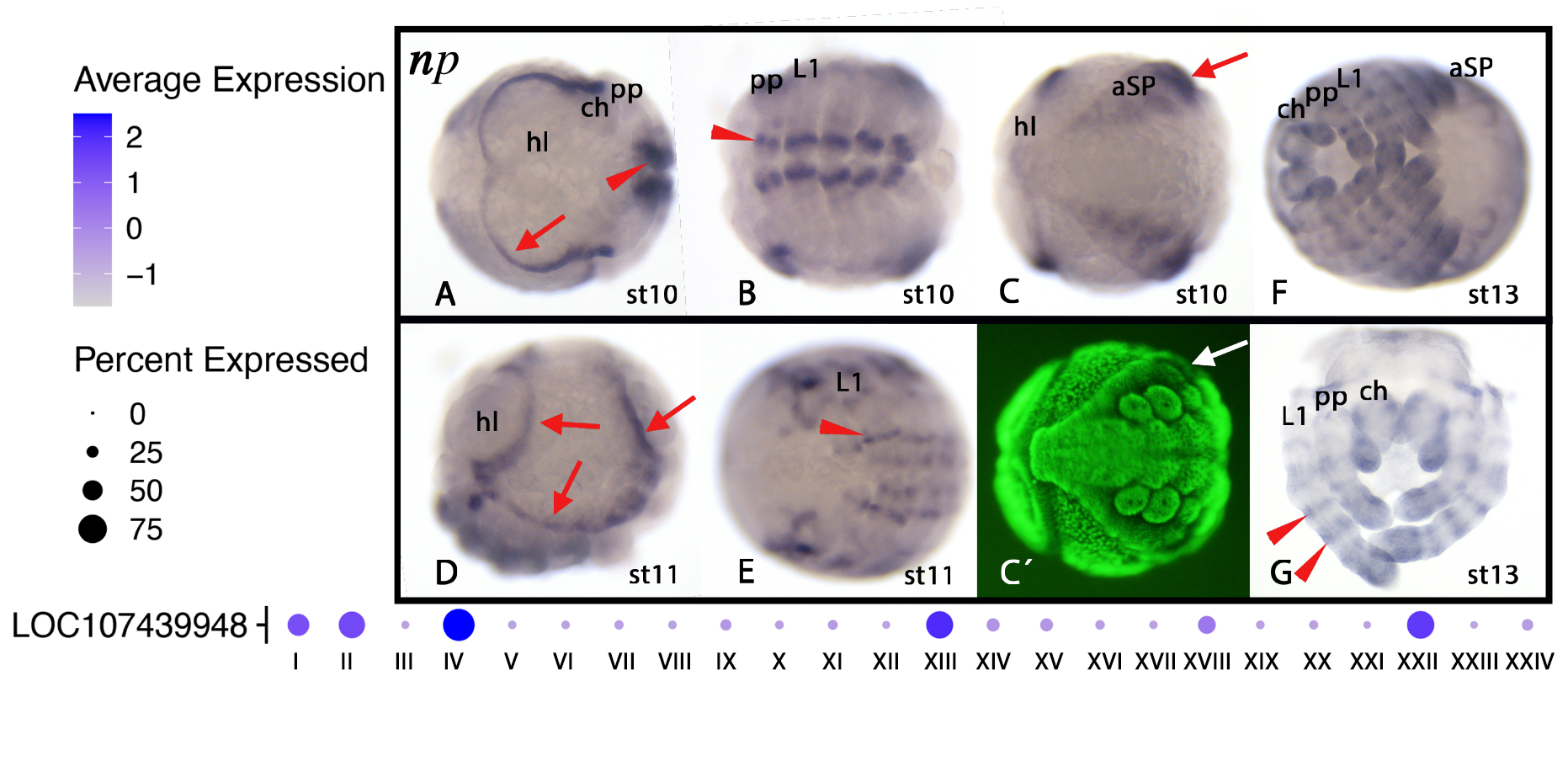

Supplement: Supplementary file 11 — Additional file 11. [file 12864_2023_9898_MOESM11_ESM.tif]

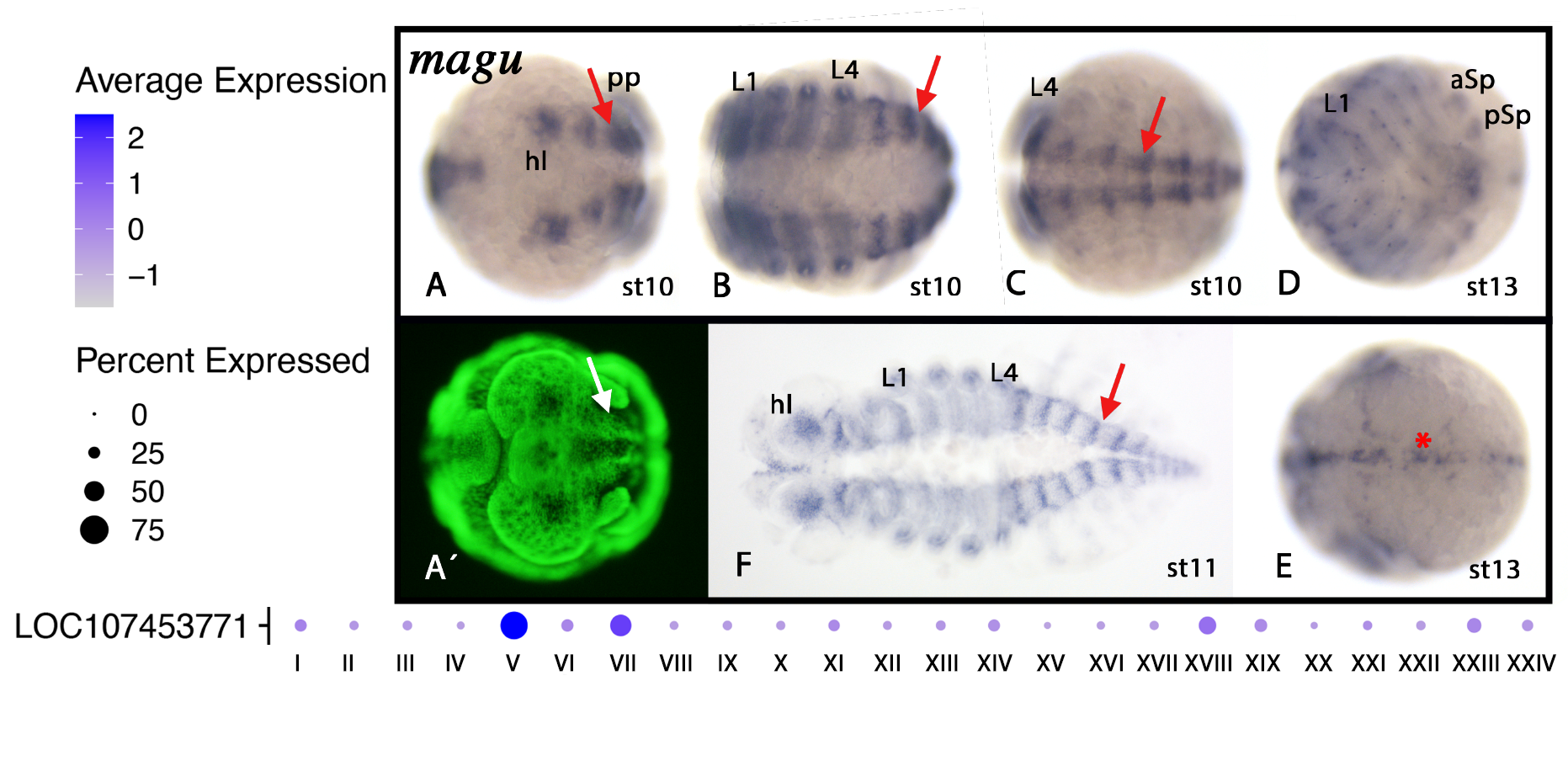

Supplement: Supplementary file 12 — Additional file 12. [file 12864_2023_9898_MOESM12_ESM.tif]

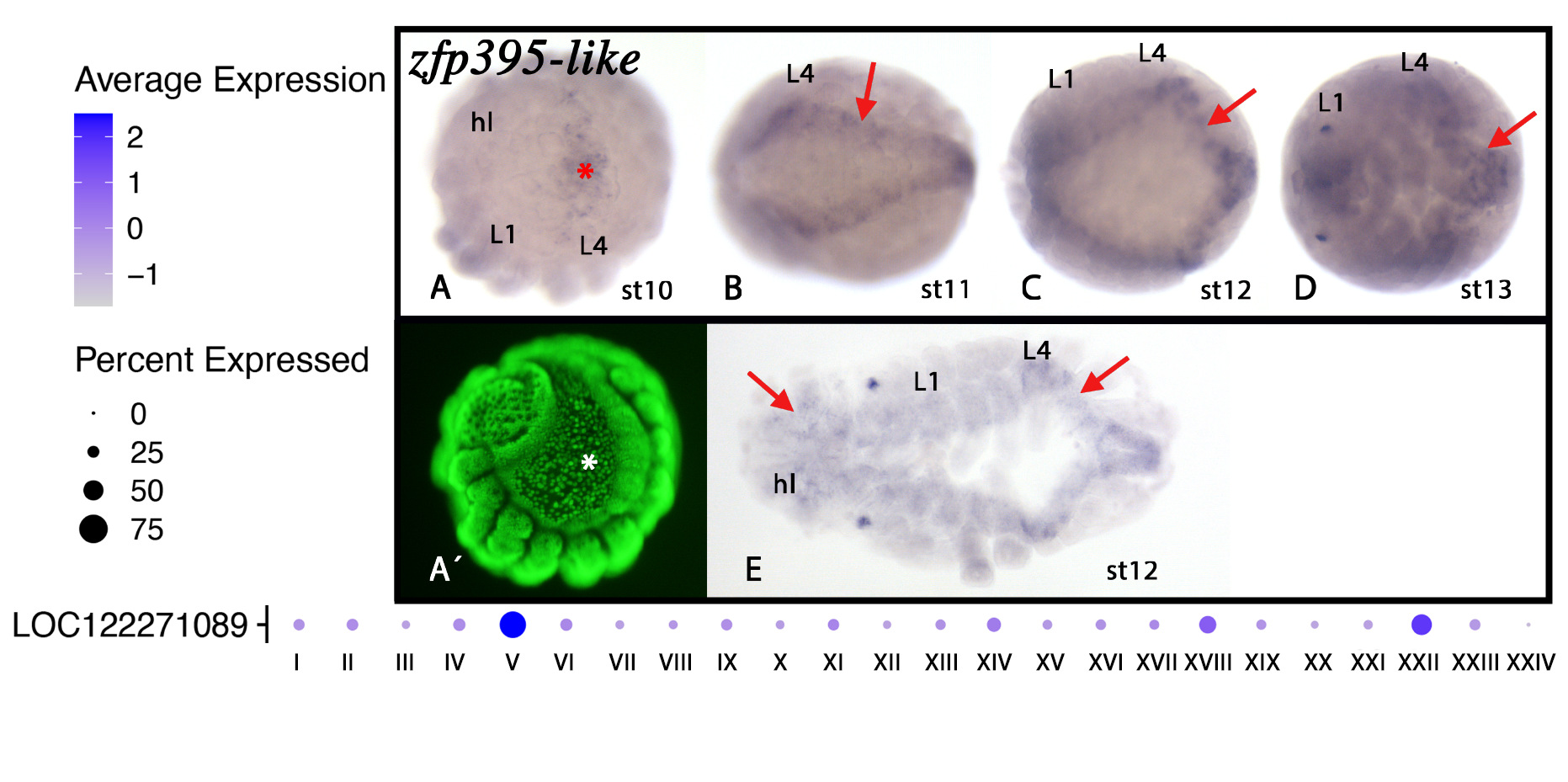

Supplement: Supplementary file 13 — Additional file 13. [file 12864_2023_9898_MOESM13_ESM.tif]

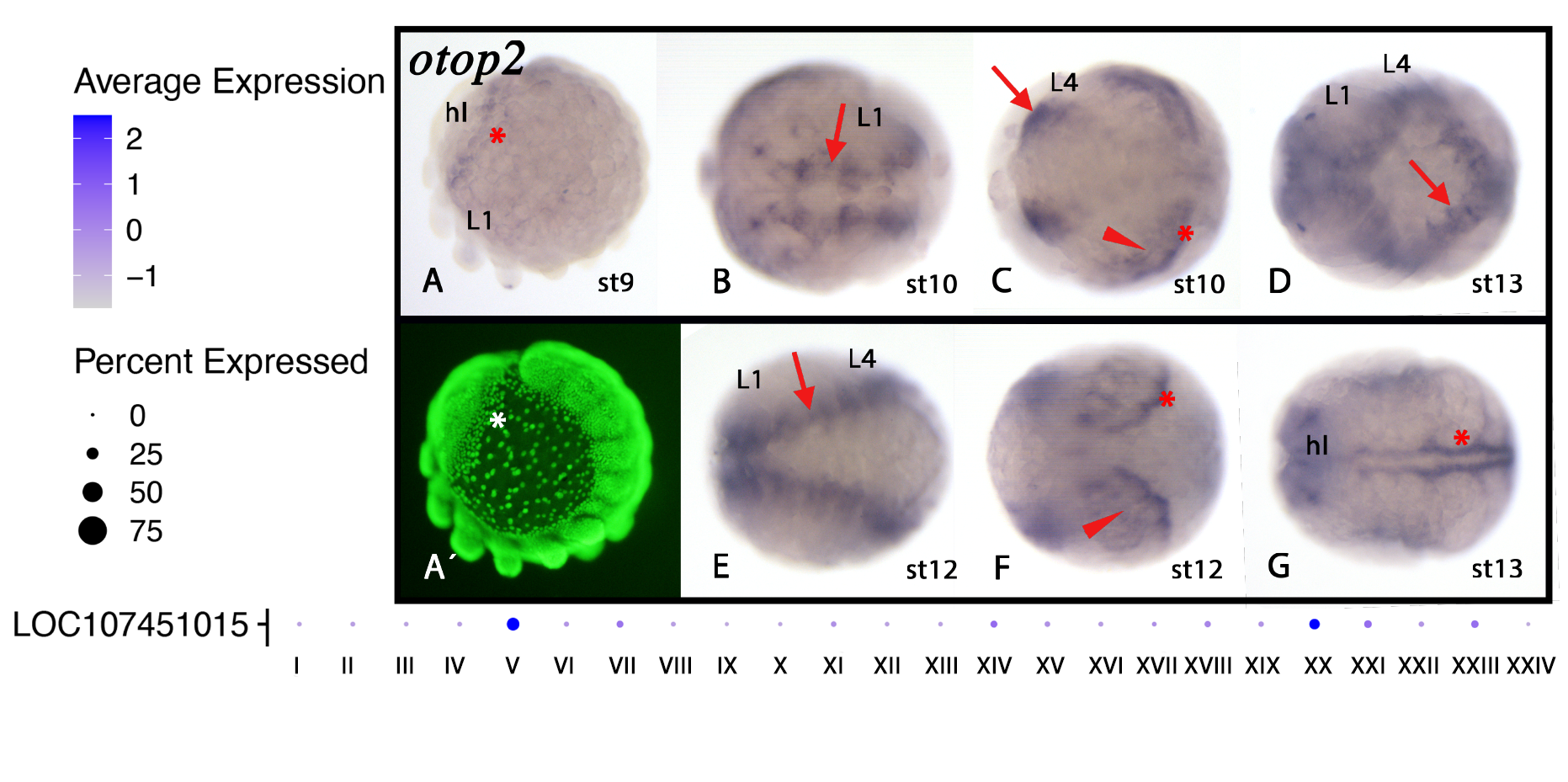

Supplement: Supplementary file 14 — Additional file 14. [file 12864_2023_9898_MOESM14_ESM.tif]

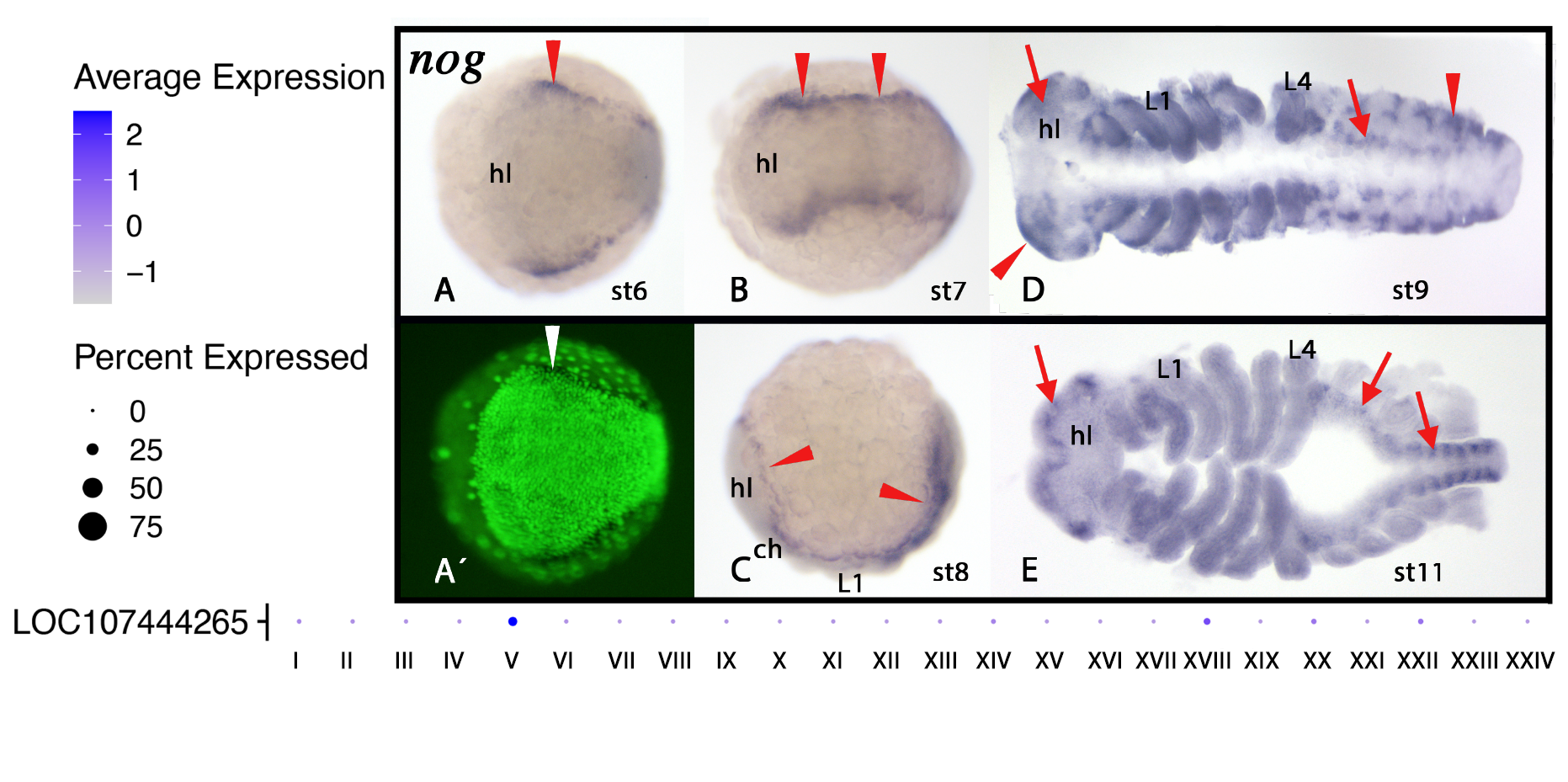

Supplement: Supplementary file 15 — Additional file 15. [file 12864_2023_9898_MOESM15_ESM.tif]

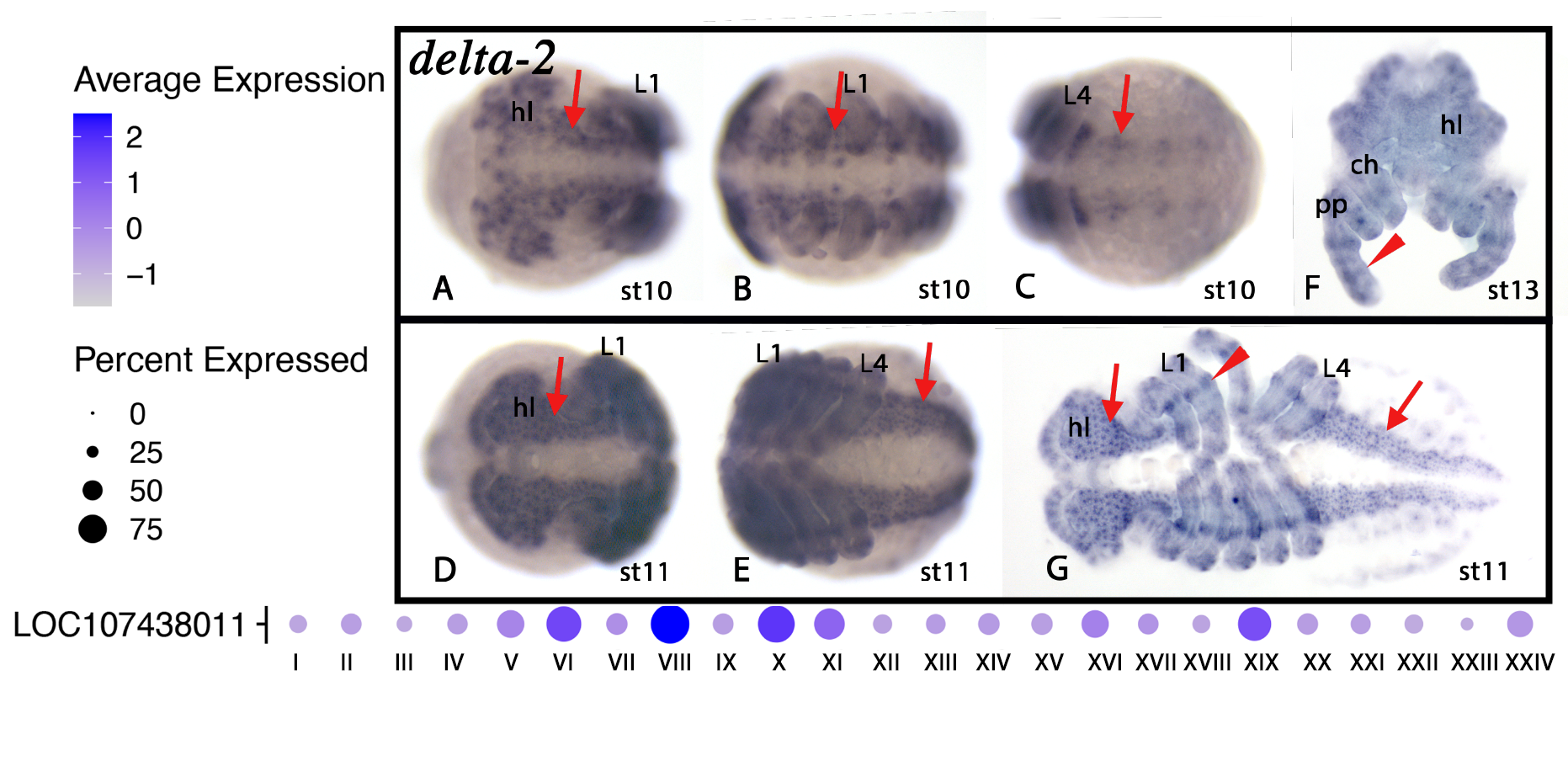

Supplement: Supplementary file 16 — Additional file 16. [file 12864_2023_9898_MOESM16_ESM.tif]

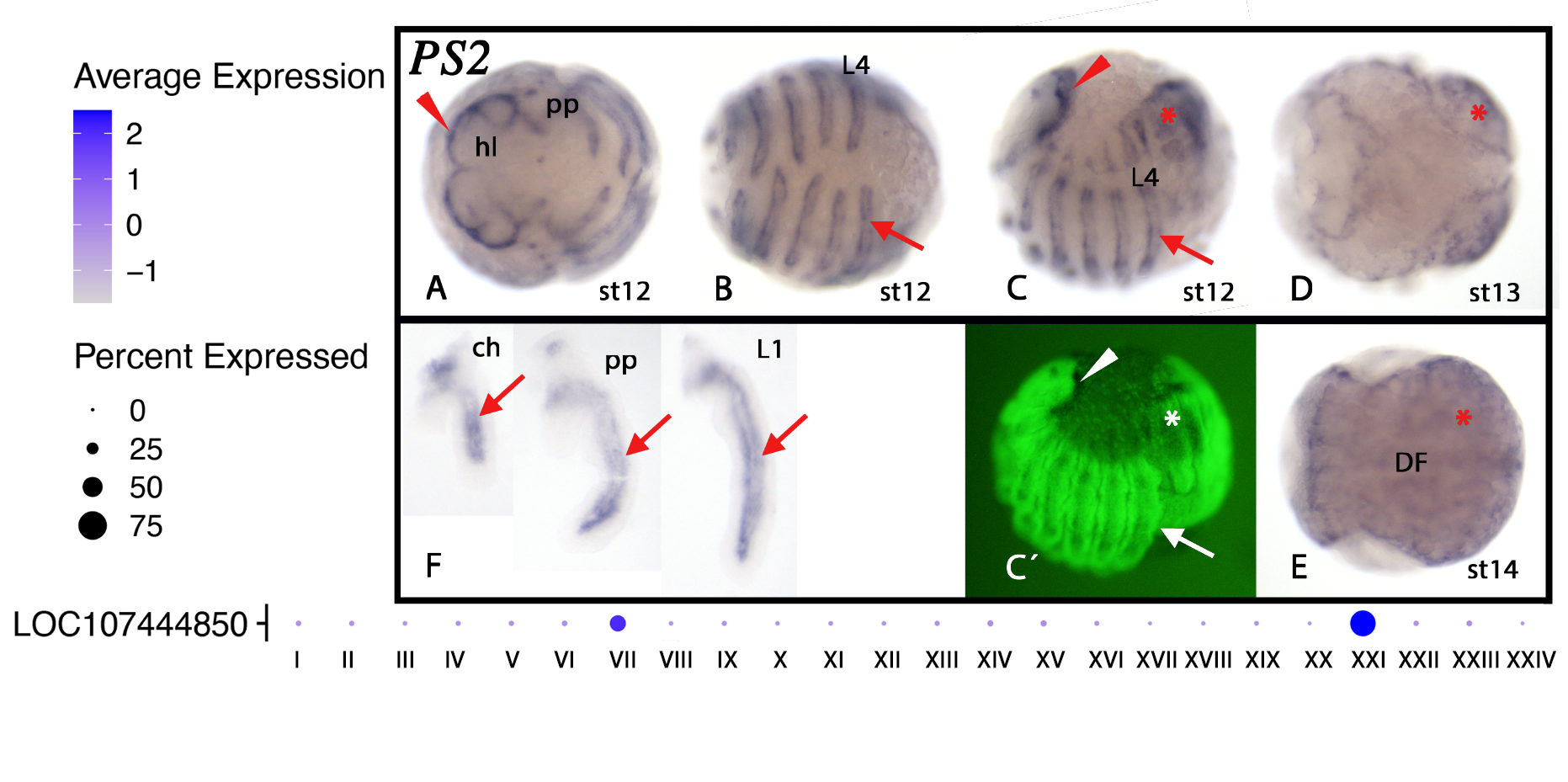

Supplement: Supplementary file 17 — Additional file 17. [file 12864_2023_9898_MOESM17_ESM.tif]

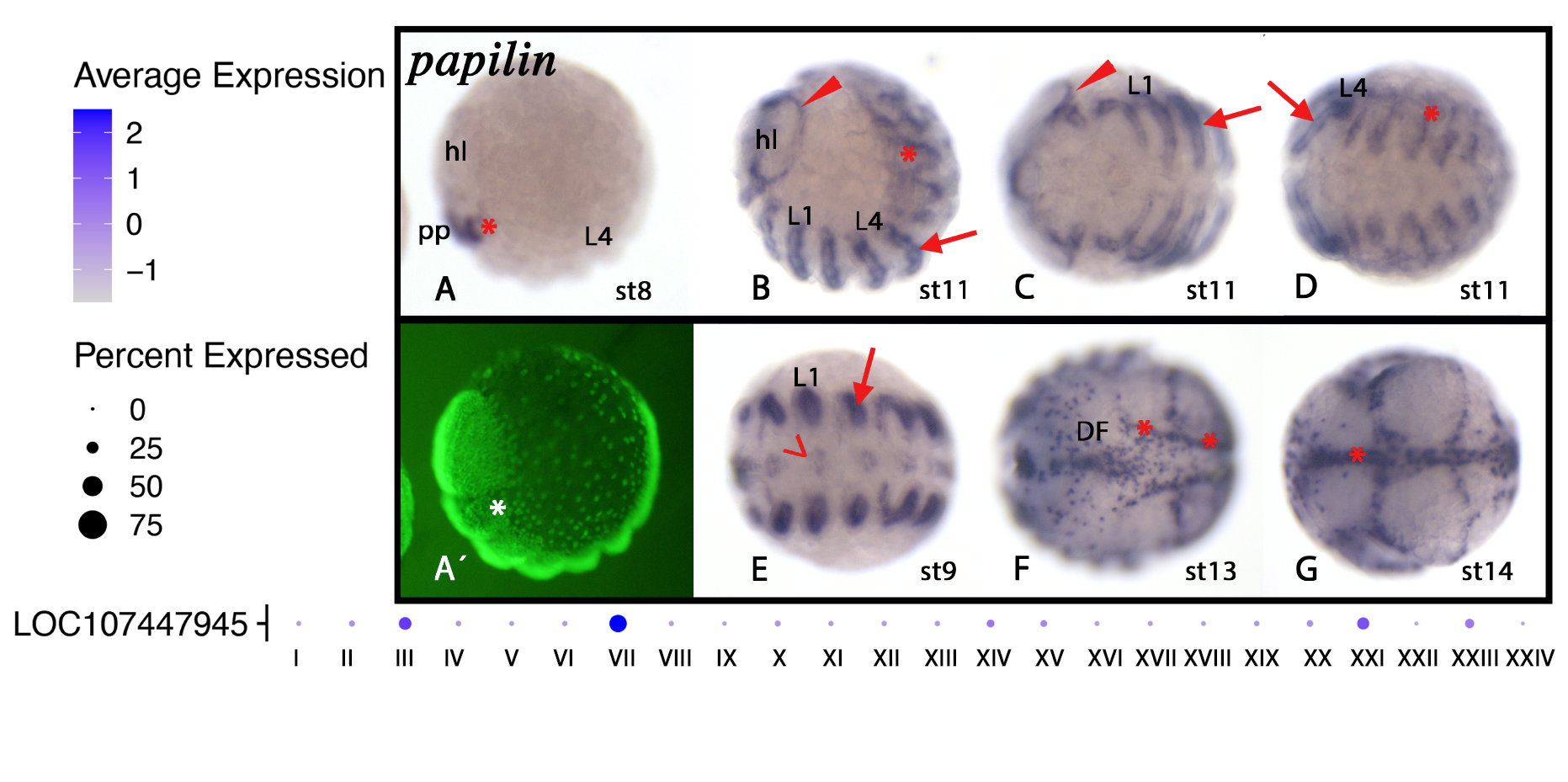

Supplement: Supplementary file 18 — Additional file 18. [file 12864_2023_9898_MOESM18_ESM.tif]

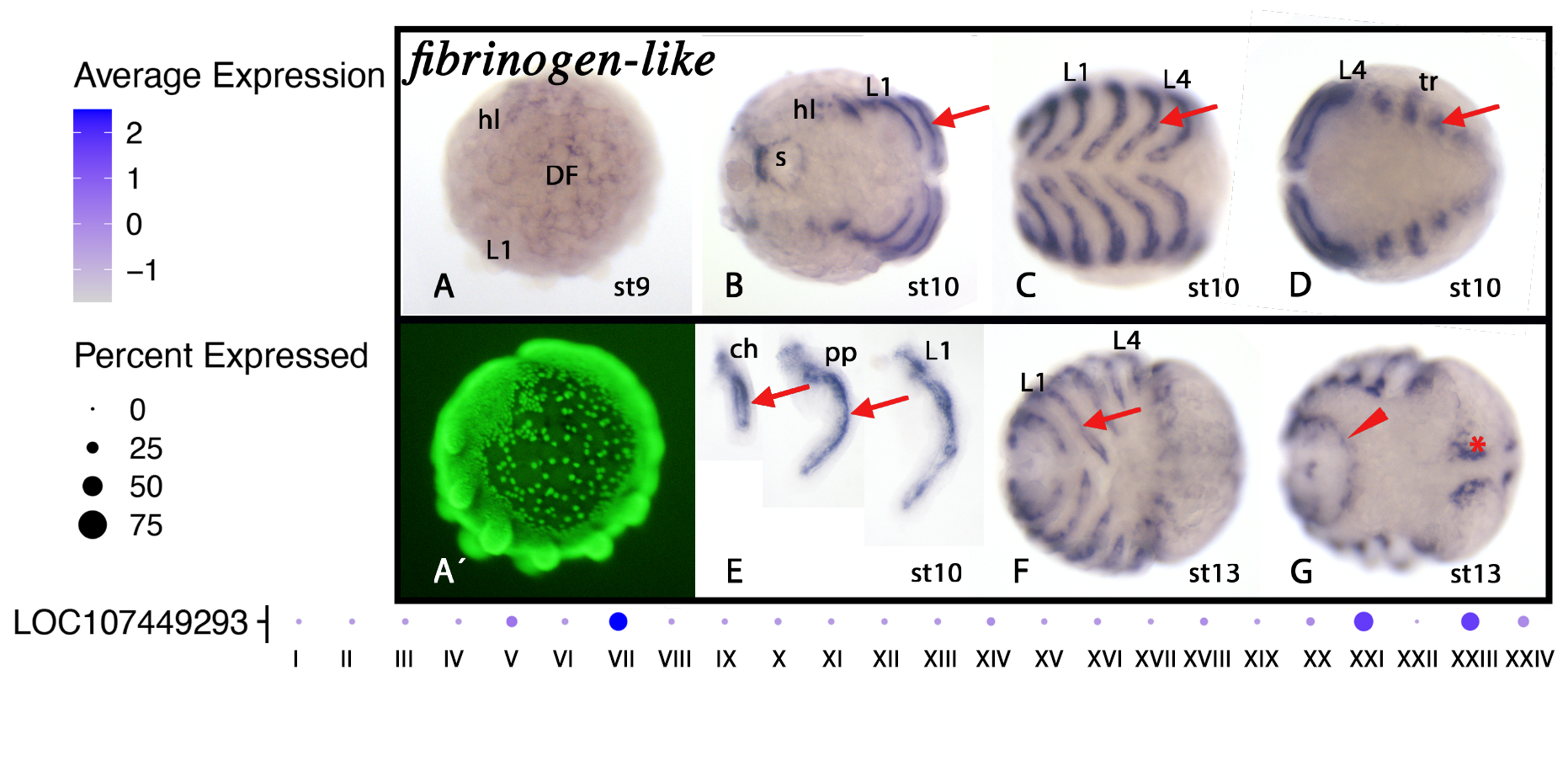

Supplement: Supplementary file 19 — Additional file 19. [file 12864_2023_9898_MOESM19_ESM.tif]

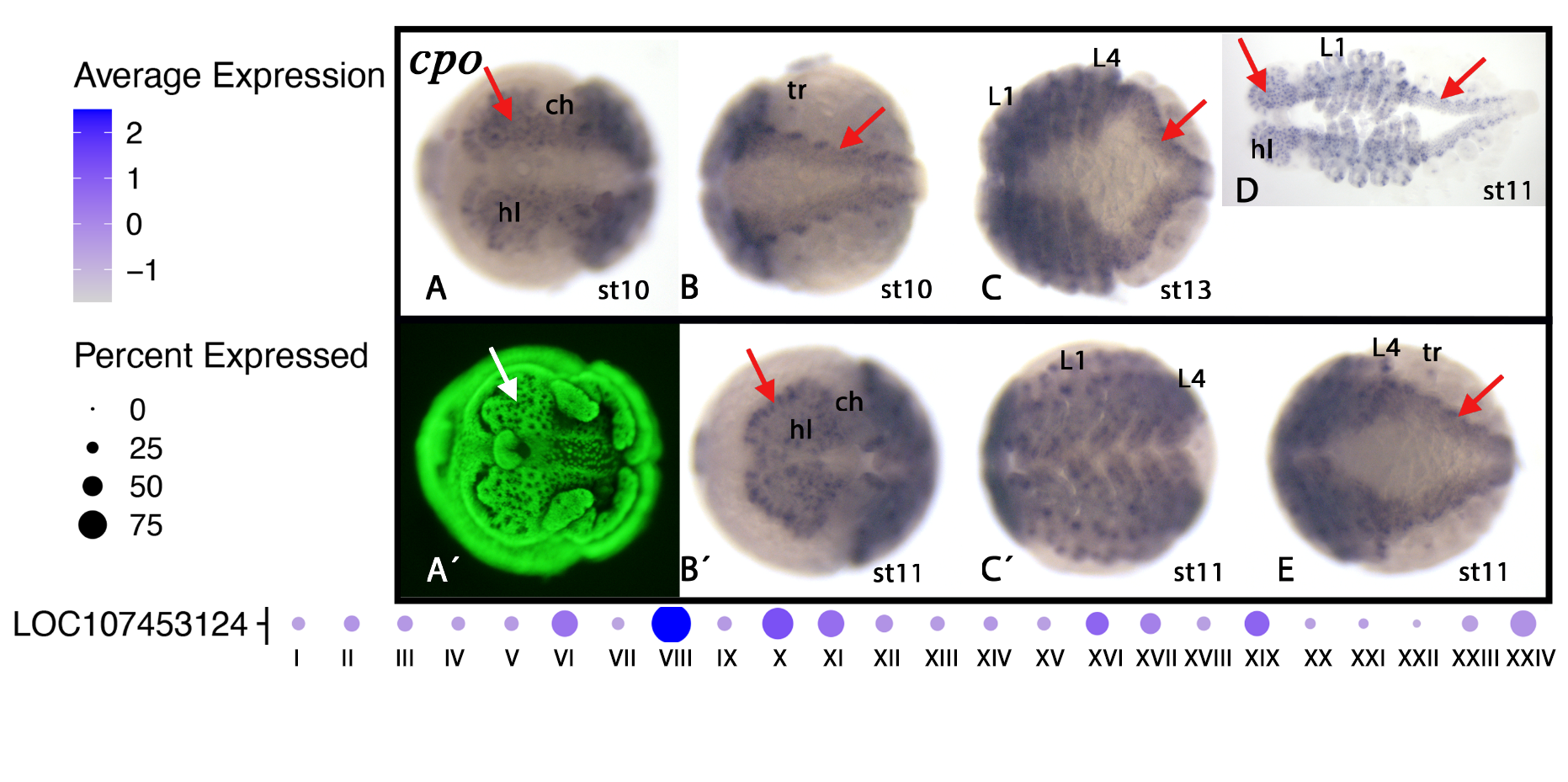

Supplement: Supplementary file 20 — Additional file 20. [file 12864_2023_9898_MOESM20_ESM.tif]

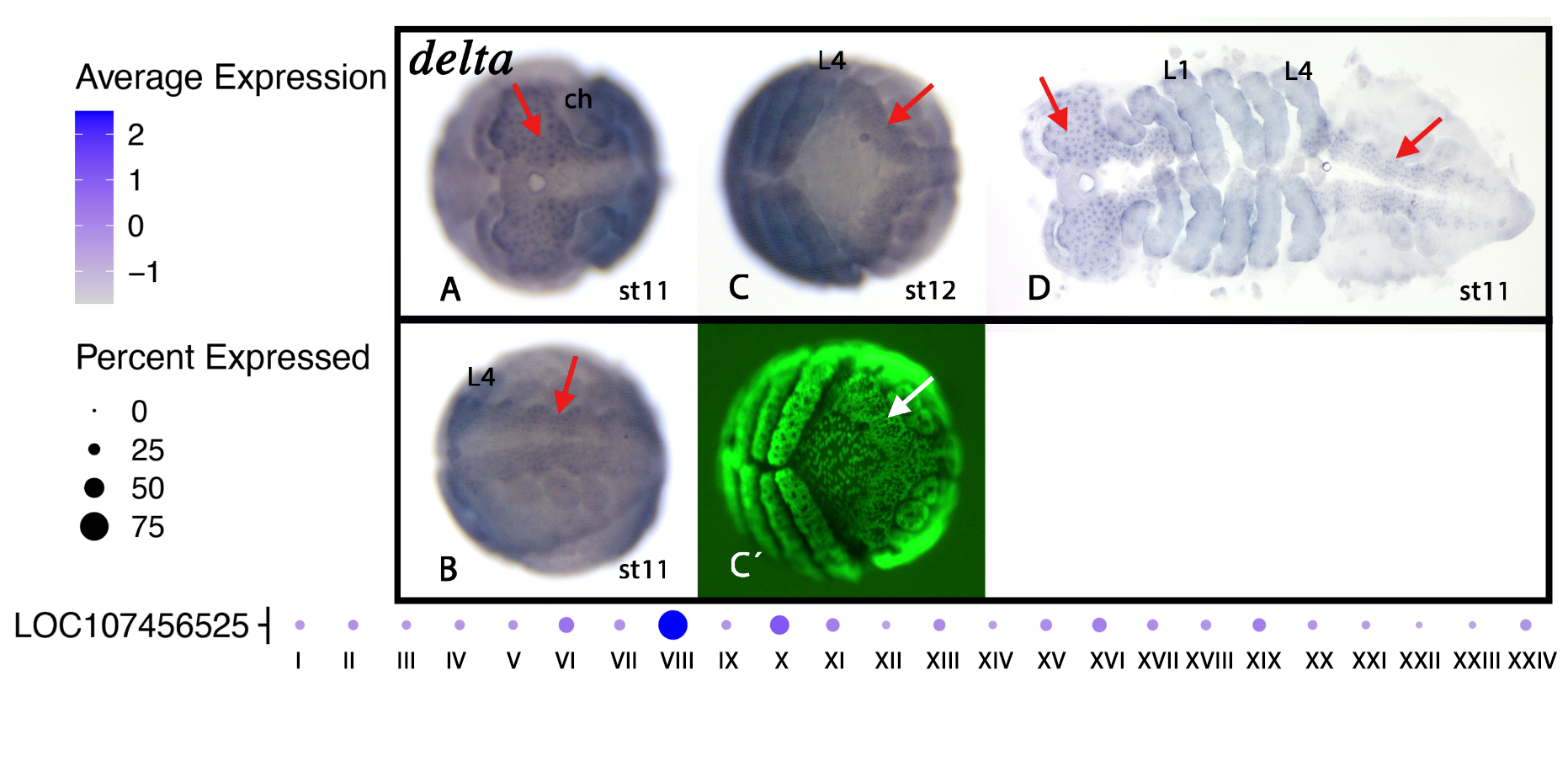

Supplement: Supplementary file 21 — Additional file 21. [file 12864_2023_9898_MOESM21_ESM.tif]

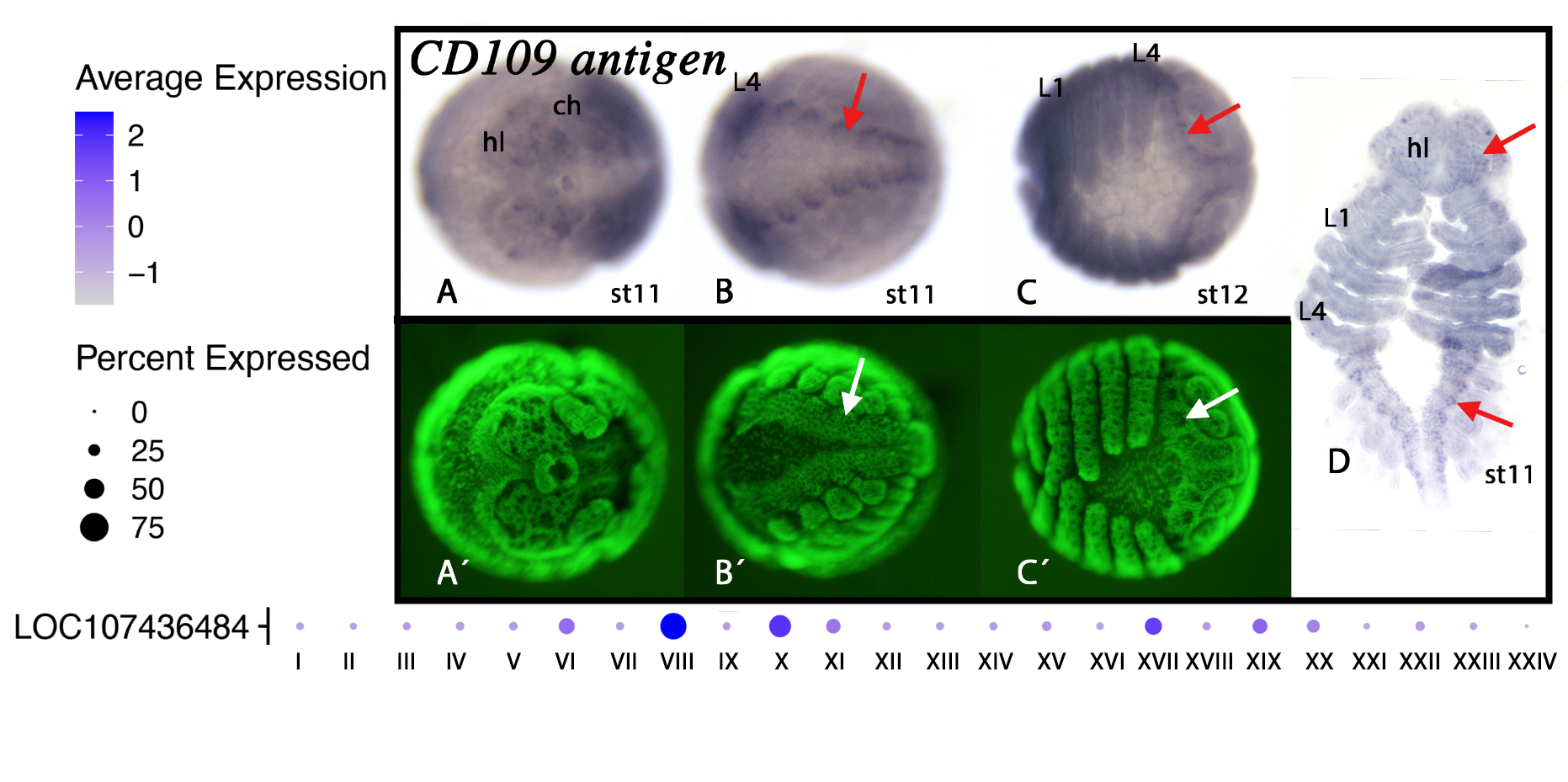

Supplement: Supplementary file 22 — Additional file 22. [file 12864_2023_9898_MOESM22_ESM.tif]

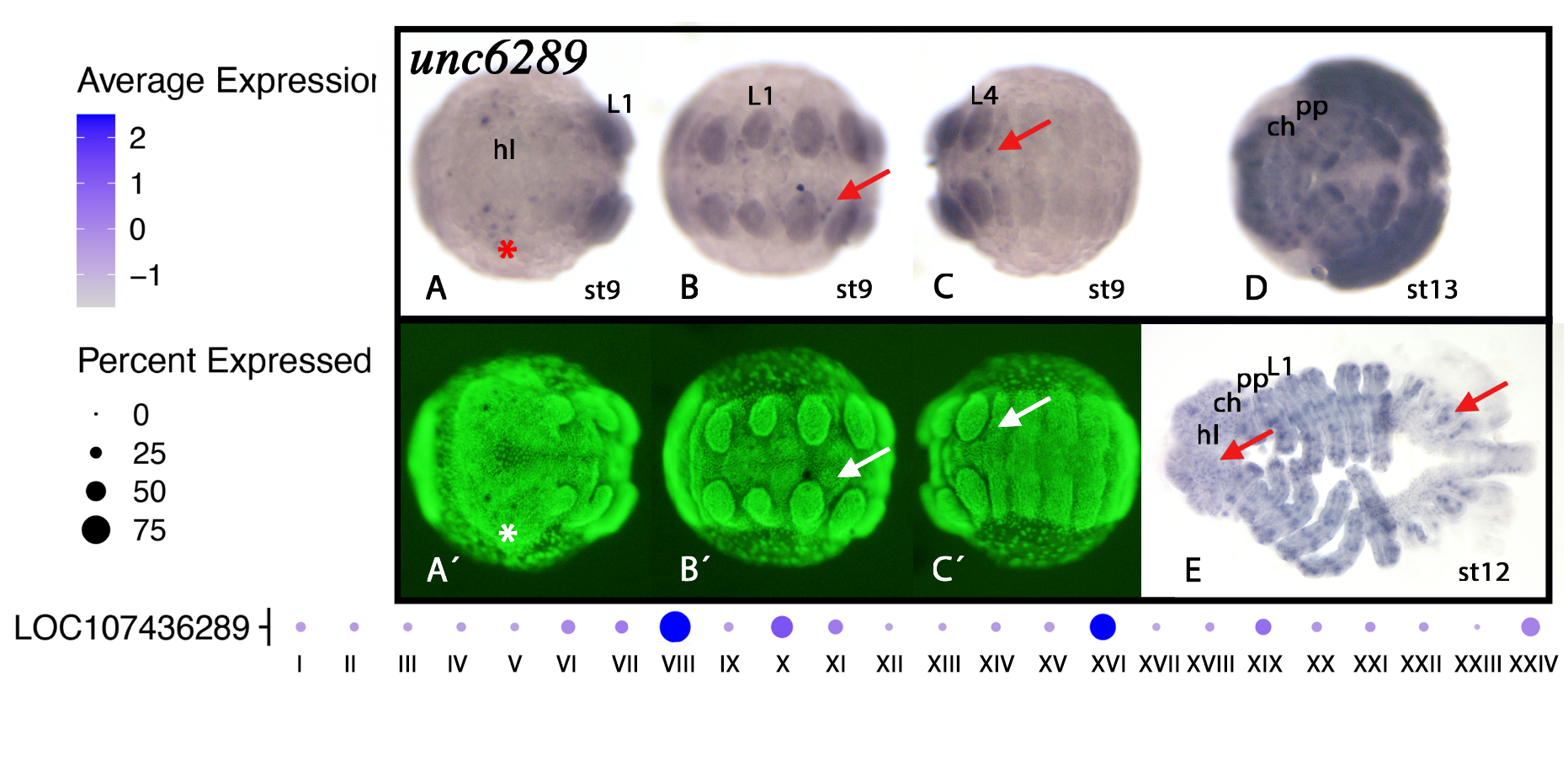

Supplement: Supplementary file 23 — Additional file 23. [file 12864_2023_9898_MOESM23_ESM.tif]

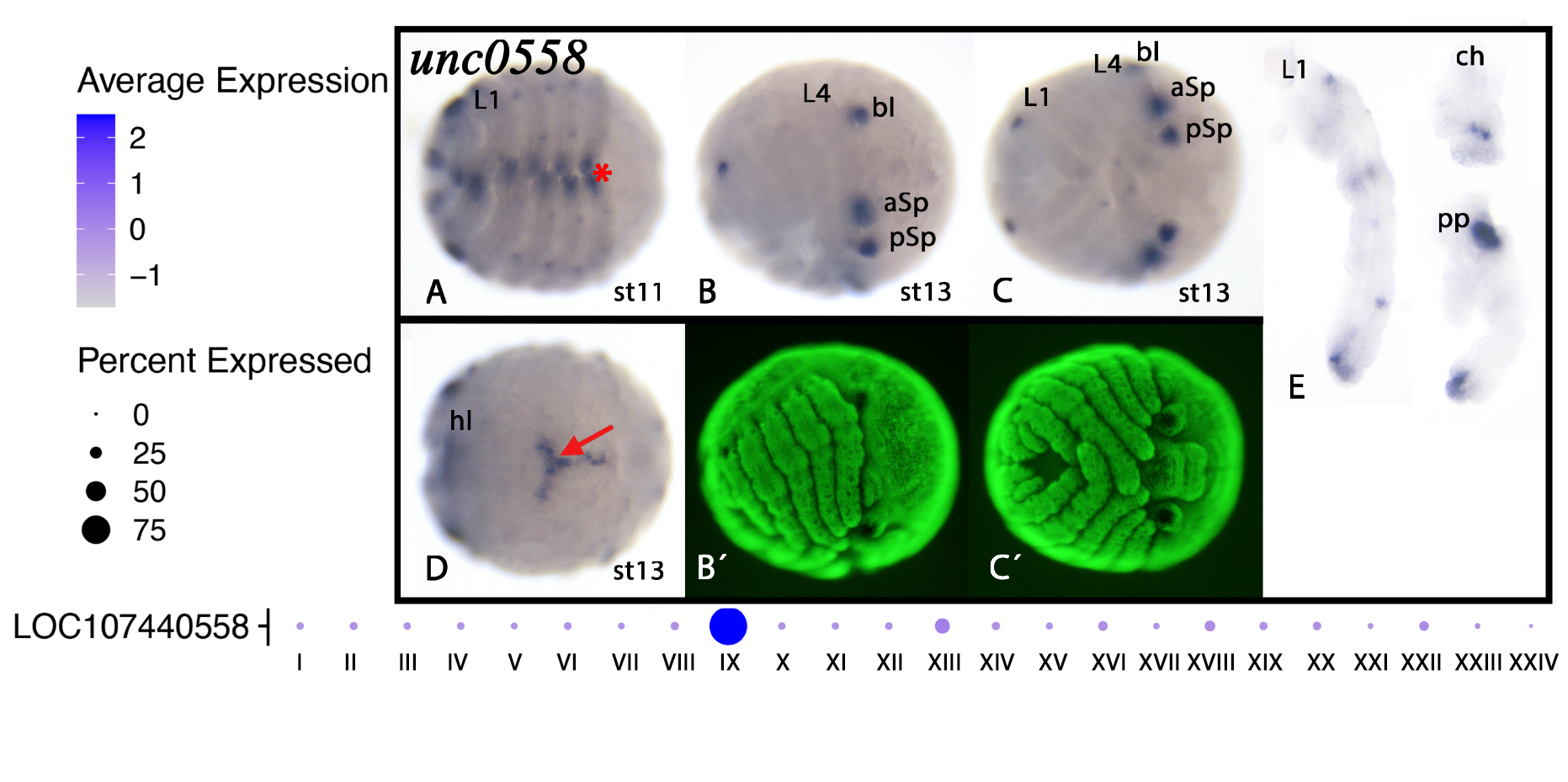

Supplement: Supplementary file 24 — Additional file 24. [file 12864_2023_9898_MOESM24_ESM.tif]

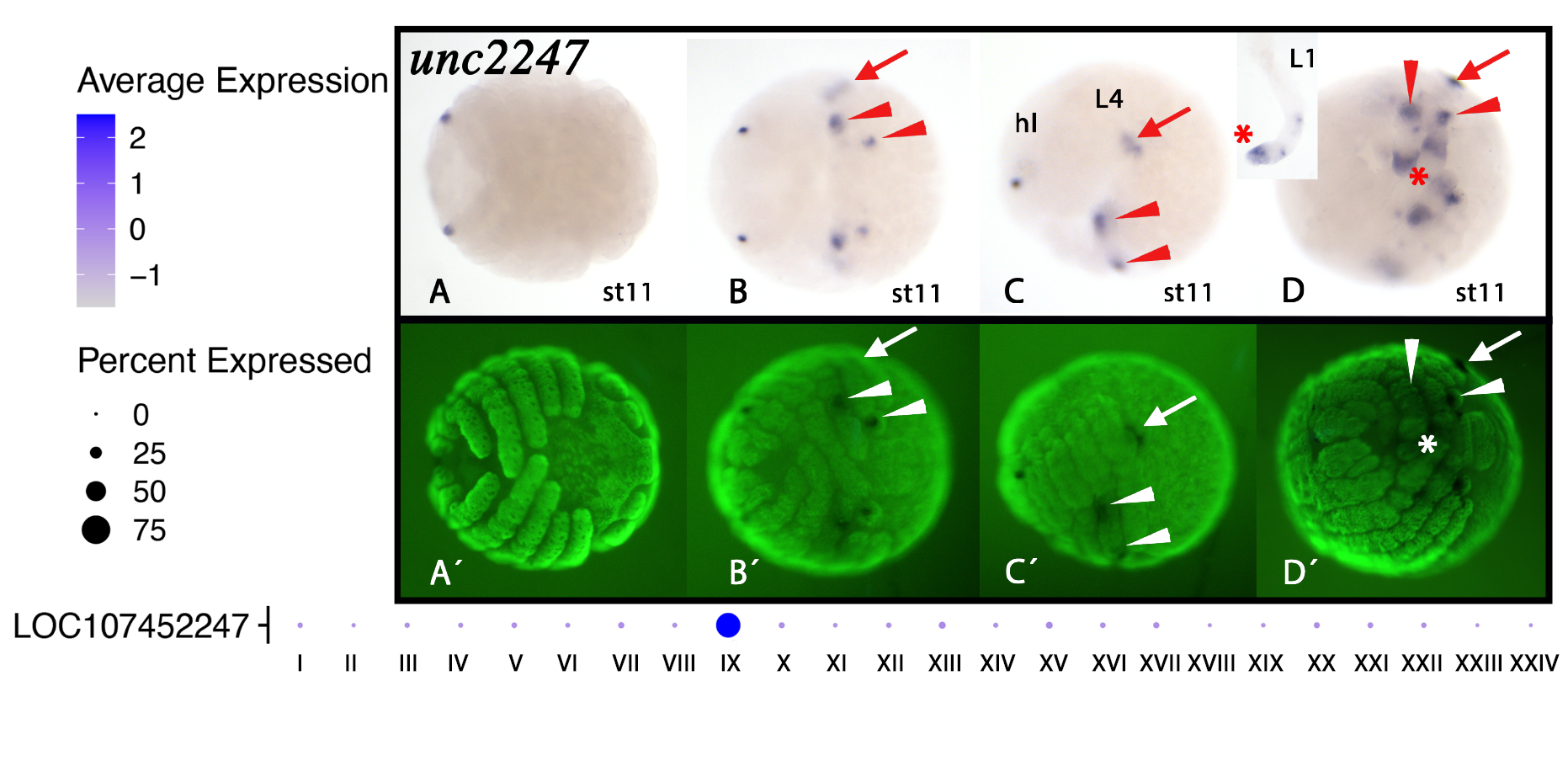

Supplement: Supplementary file 25 — Additional file 25. [file 12864_2023_9898_MOESM25_ESM.tif]

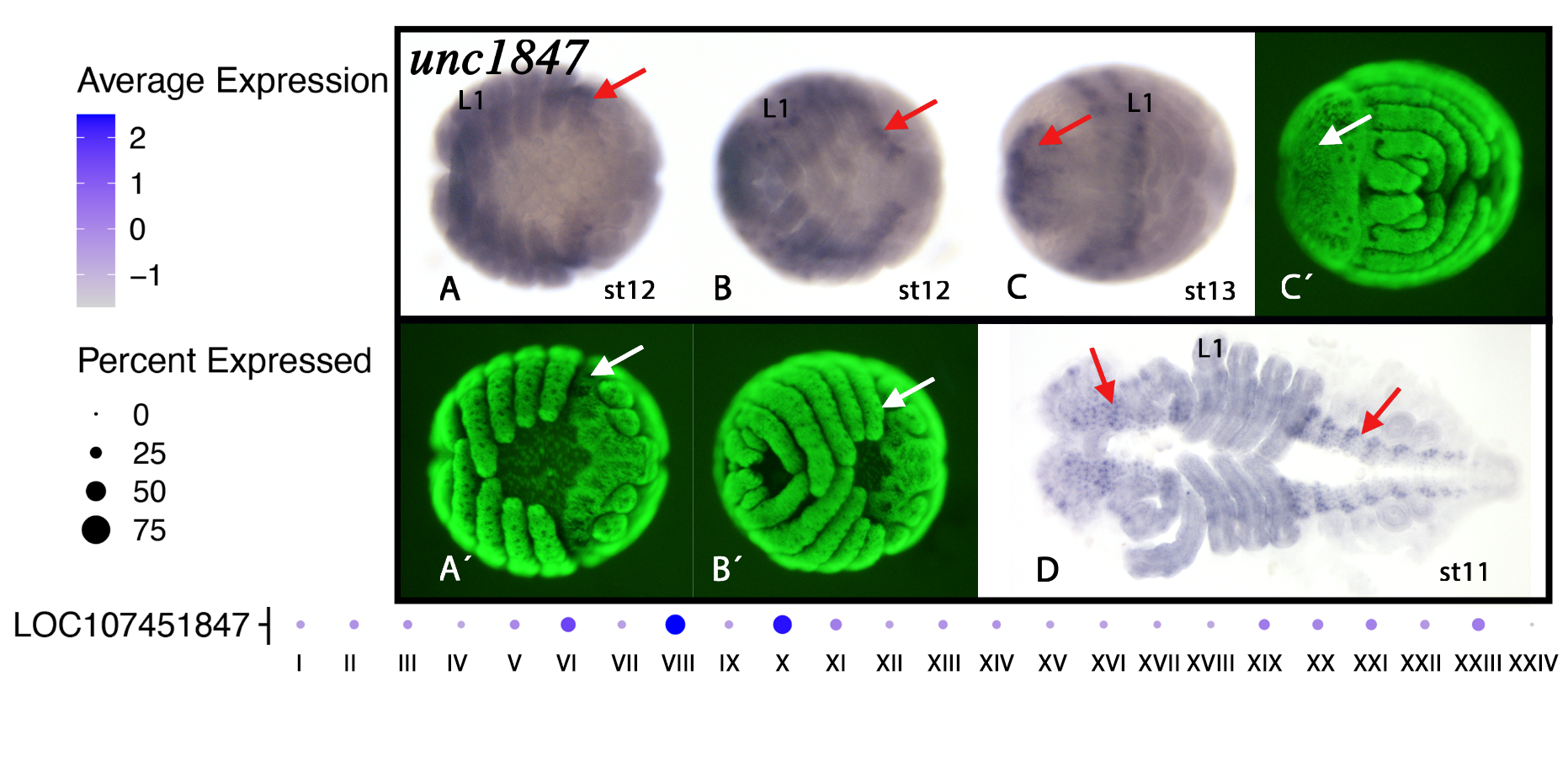

Supplement: Supplementary file 26 — Additional file 26. [file 12864_2023_9898_MOESM26_ESM.tif]

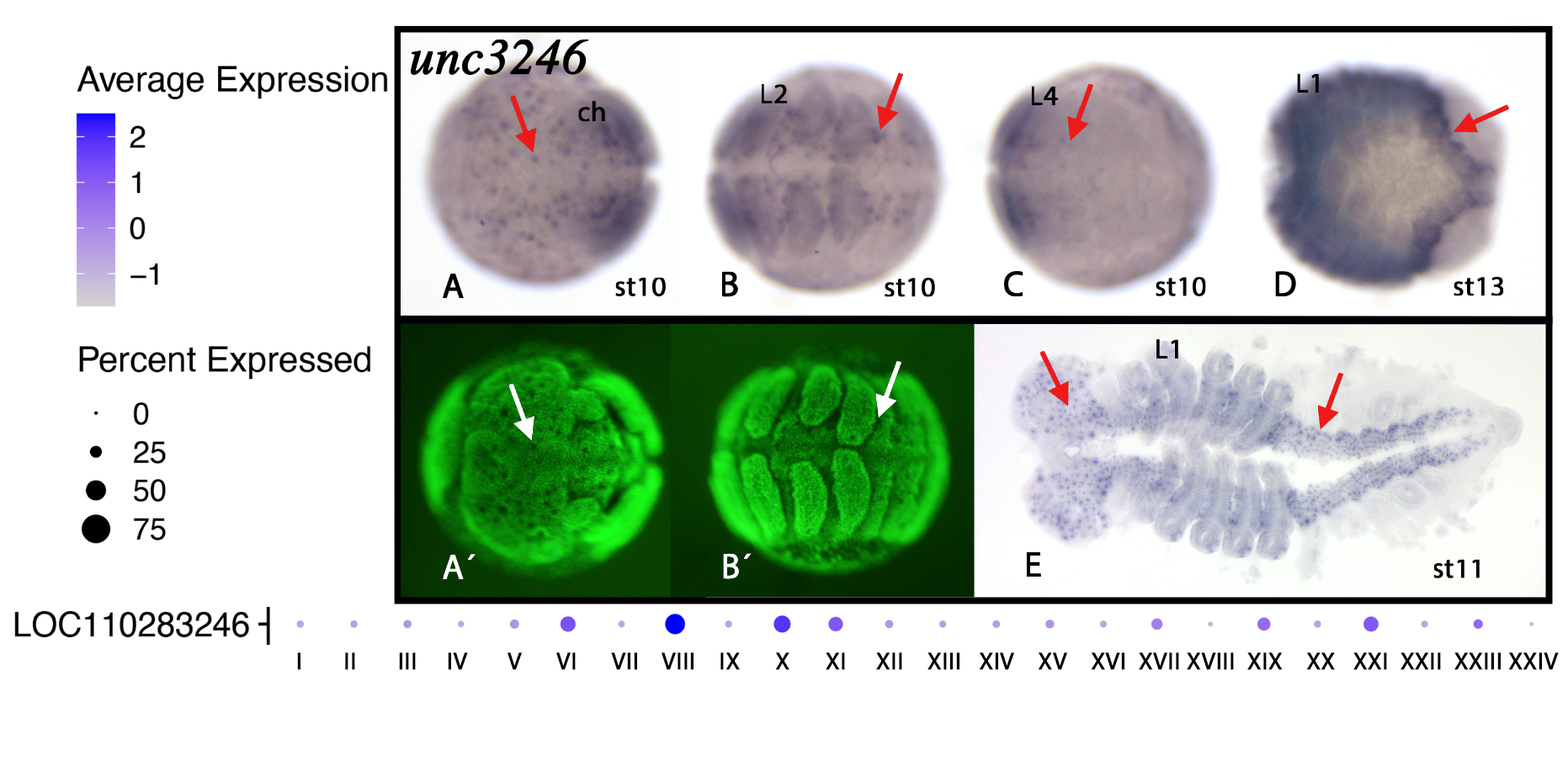

Supplement: Supplementary file 27 — Additional file 27. [file 12864_2023_9898_MOESM27_ESM.tif]

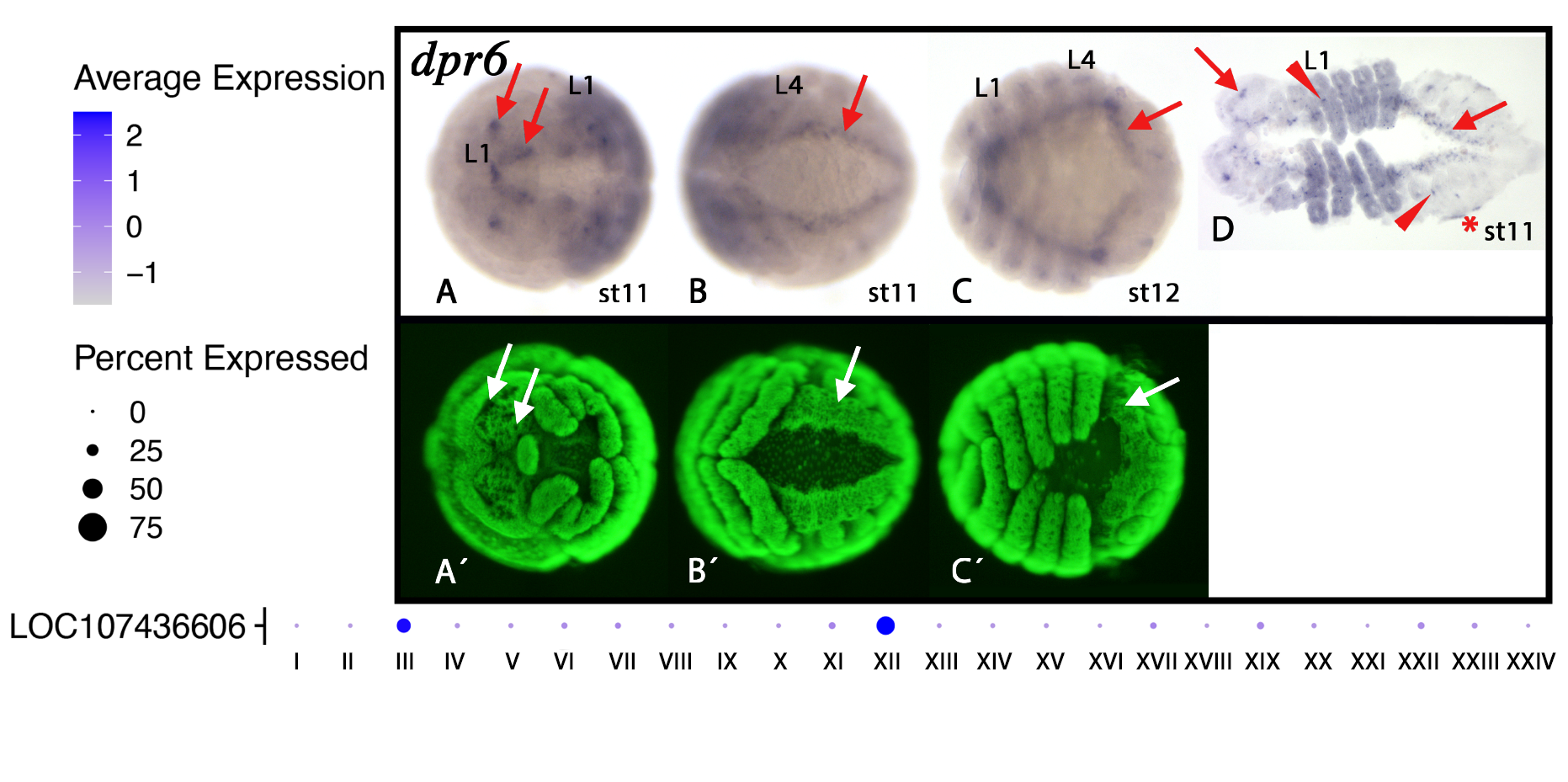

Supplement: Supplementary file 28 — Additional file 28. [file 12864_2023_9898_MOESM28_ESM.tif]

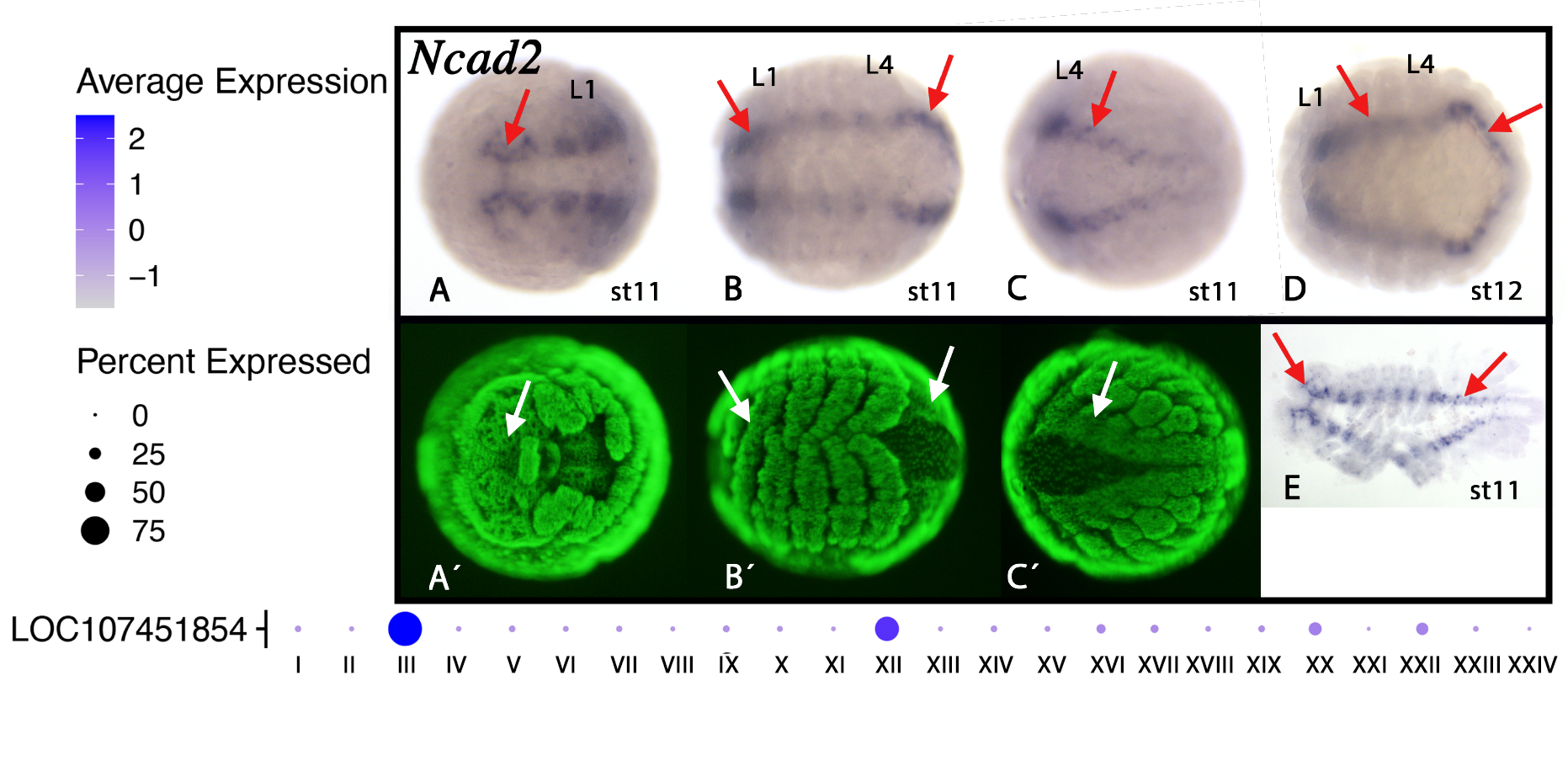

Supplement: Supplementary file 29 — Additional file 29. [file 12864_2023_9898_MOESM29_ESM.tif]

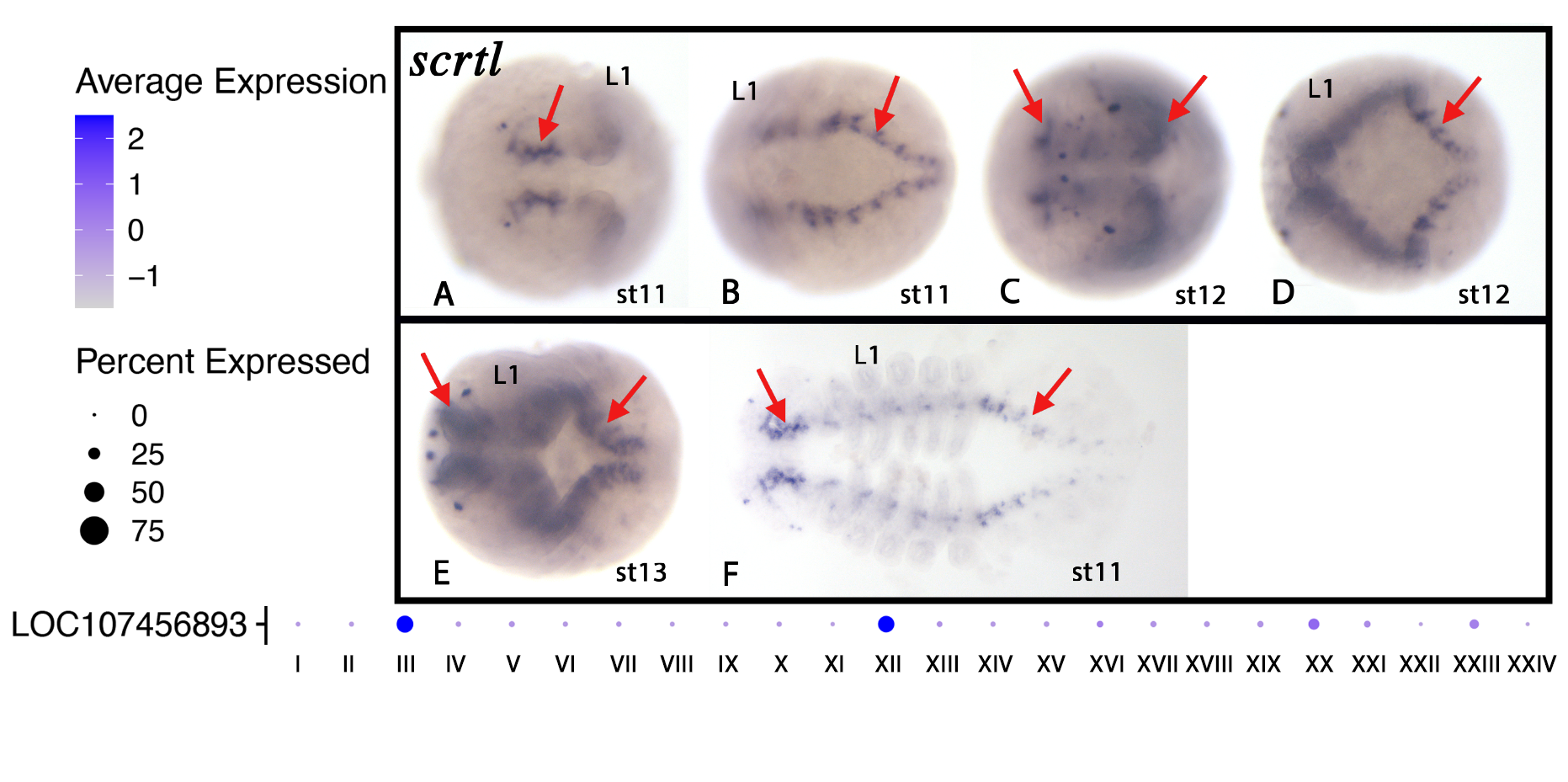

Supplement: Supplementary file 30 — Additional file 30. [file 12864_2023_9898_MOESM30_ESM.tif]

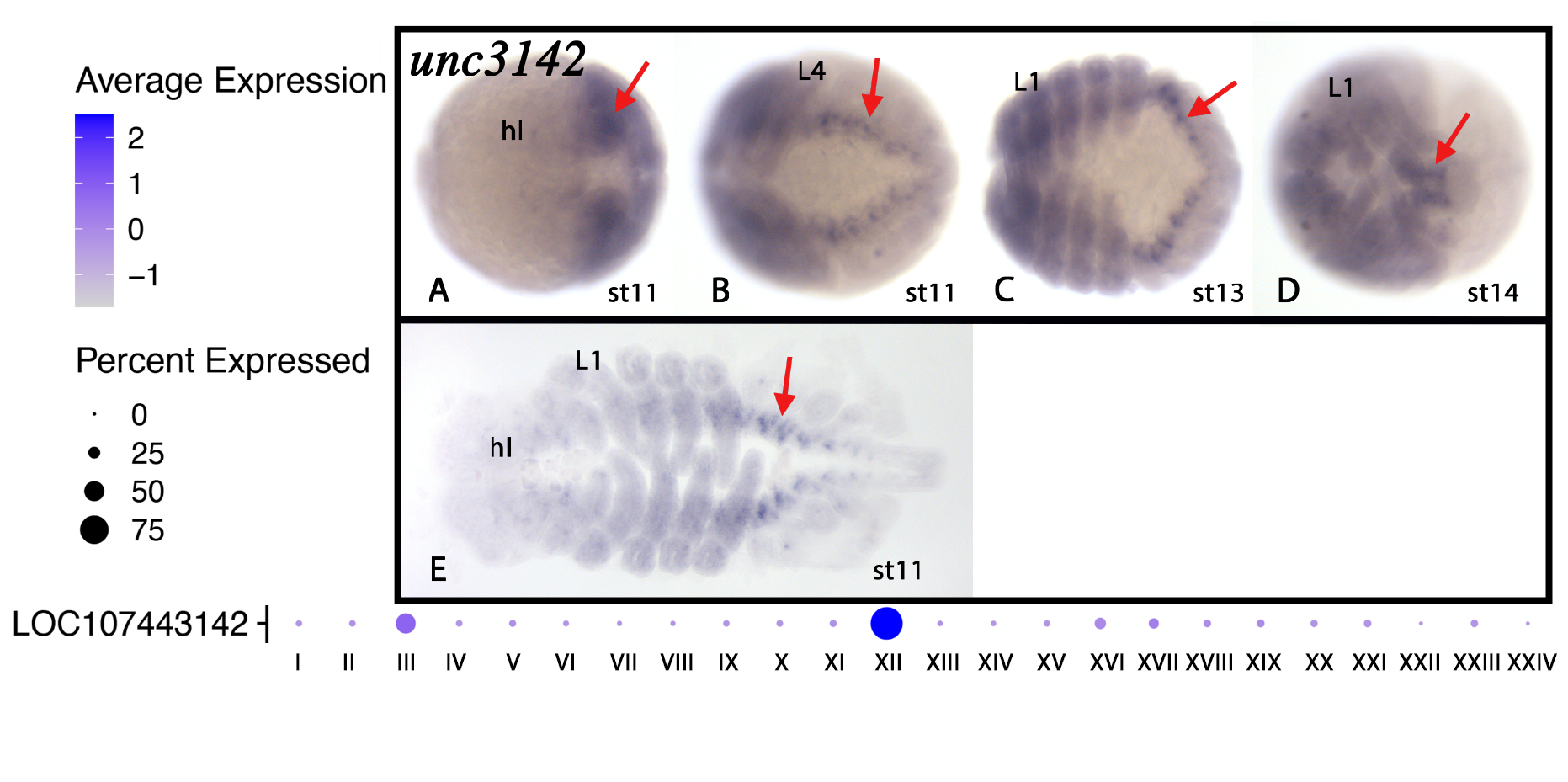

Supplement: Supplementary file 31 — Additional file 31. [file 12864_2023_9898_MOESM31_ESM.tif]

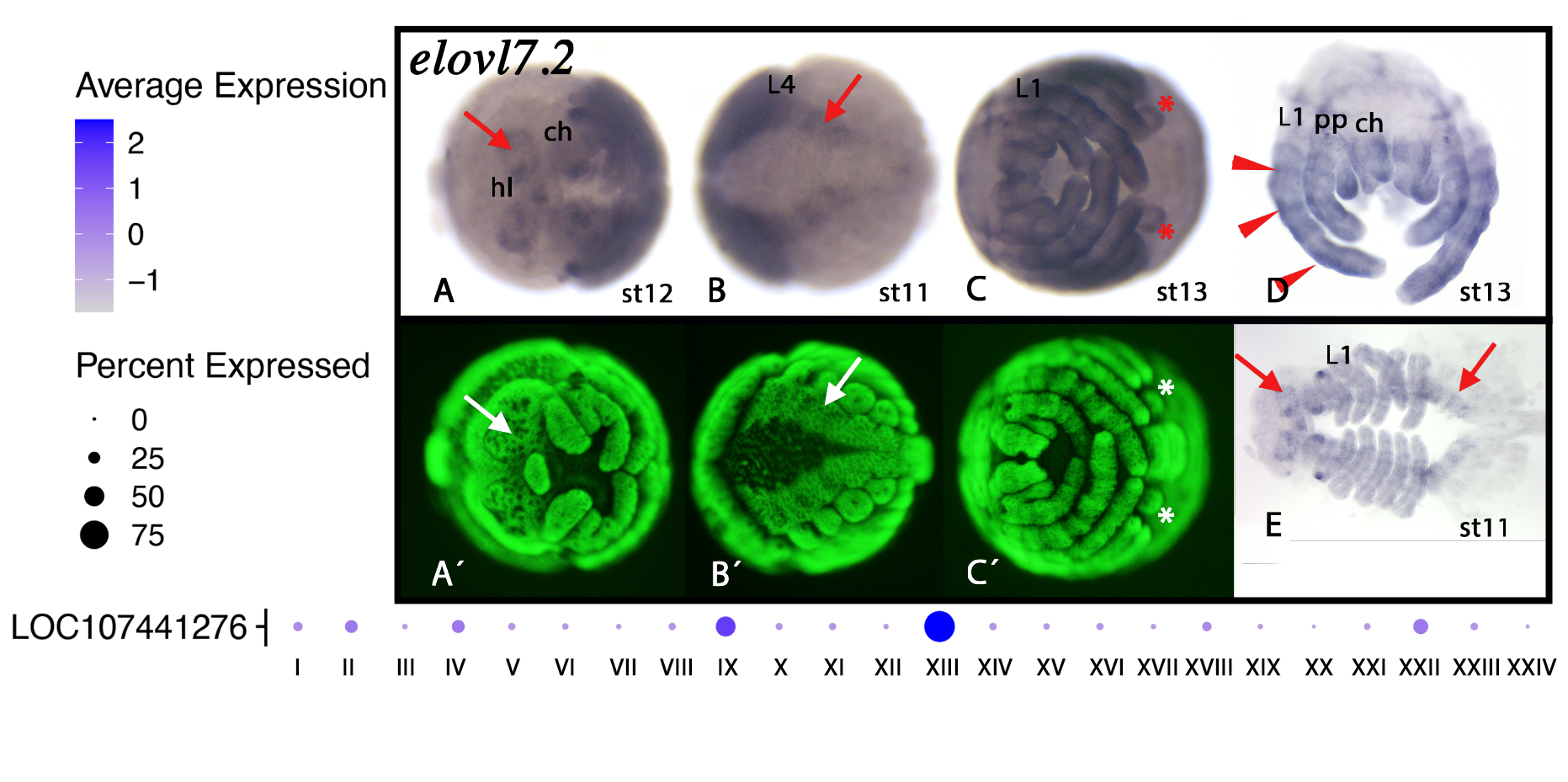

Supplement: Supplementary file 32 — Additional file 32. [file 12864_2023_9898_MOESM32_ESM.tif]

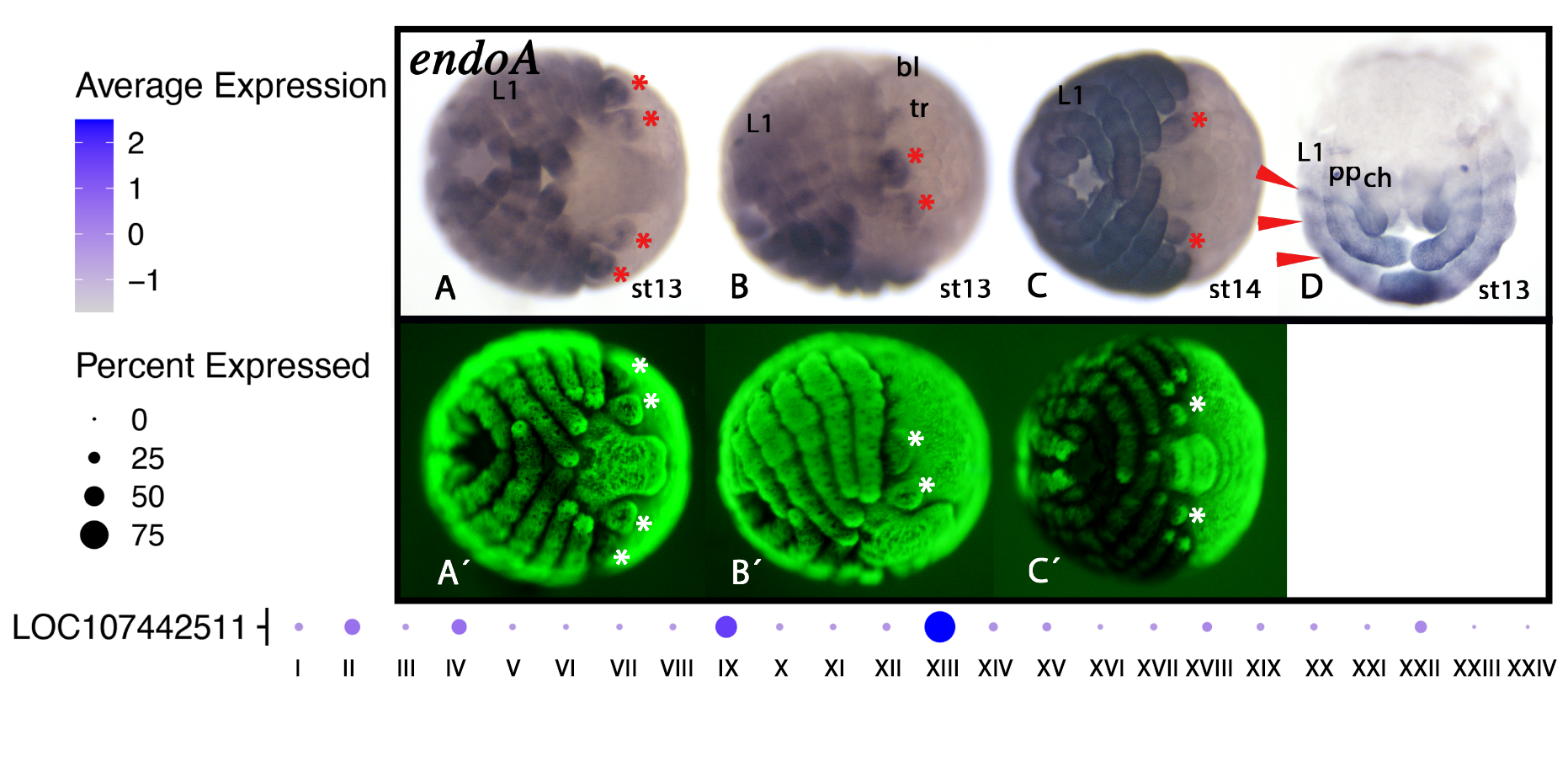

Supplement: Supplementary file 33 — Additional file 33. [file 12864_2023_9898_MOESM33_ESM.tif]

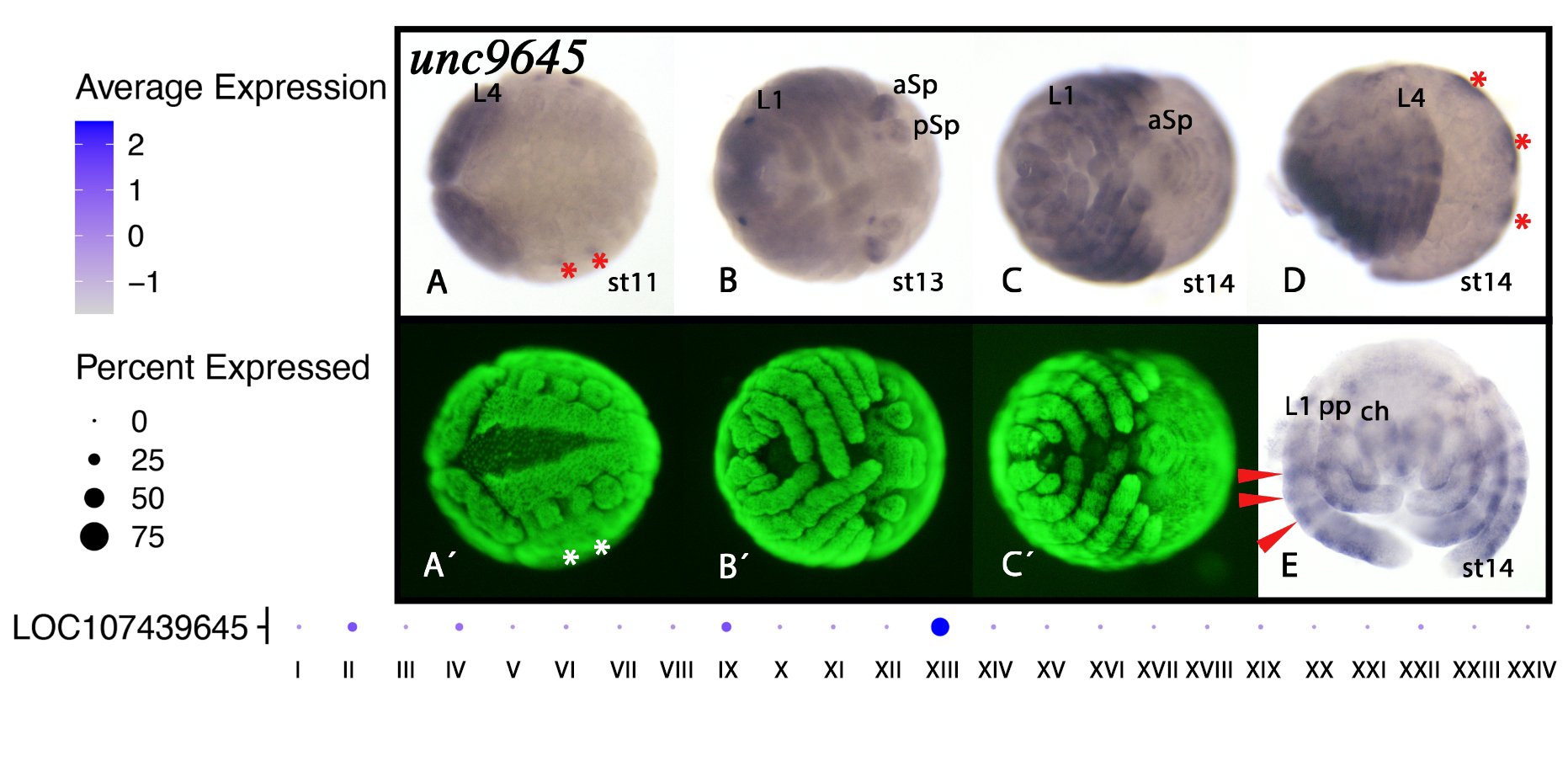

Supplement: Supplementary file 34 — Additional file 34. [file 12864_2023_9898_MOESM34_ESM.tif]

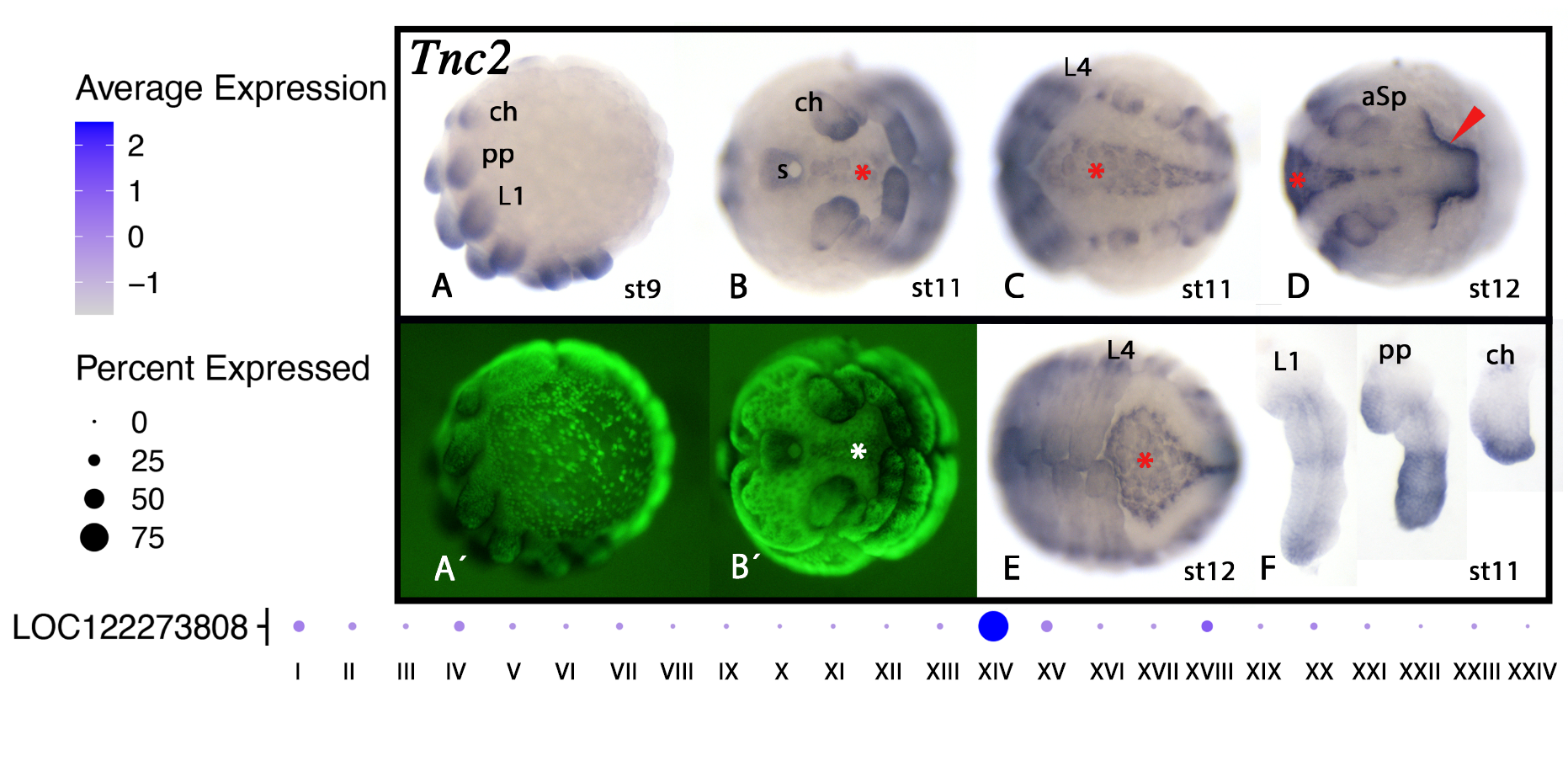

Supplement: Supplementary file 35 — Additional file 35. [file 12864_2023_9898_MOESM35_ESM.tif]

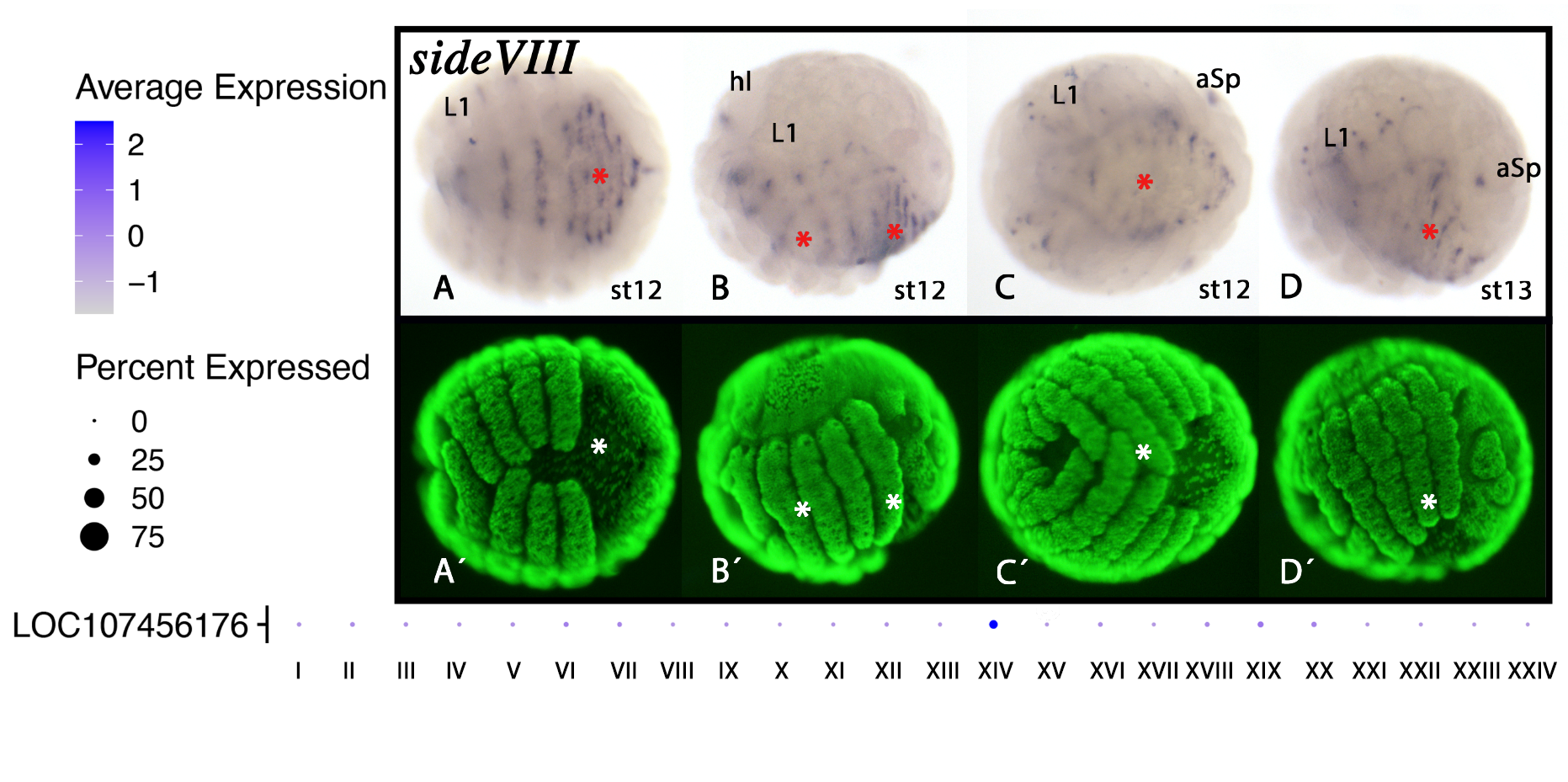

Supplement: Supplementary file 36 — Additional file 36. [file 12864_2023_9898_MOESM36_ESM.tif]

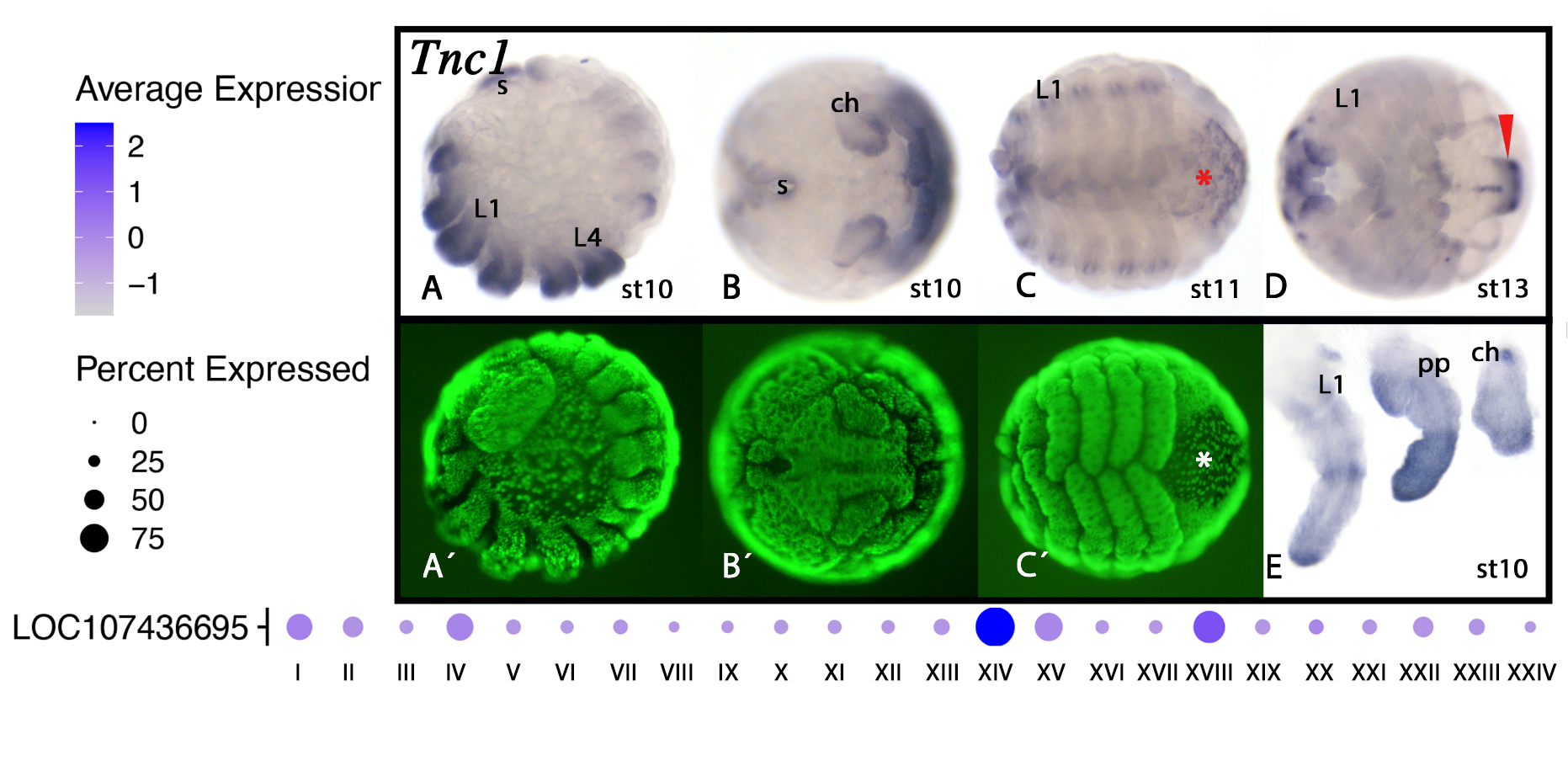

Supplement: Supplementary file 37 — Additional file 37. [file 12864_2023_9898_MOESM37_ESM.tif]

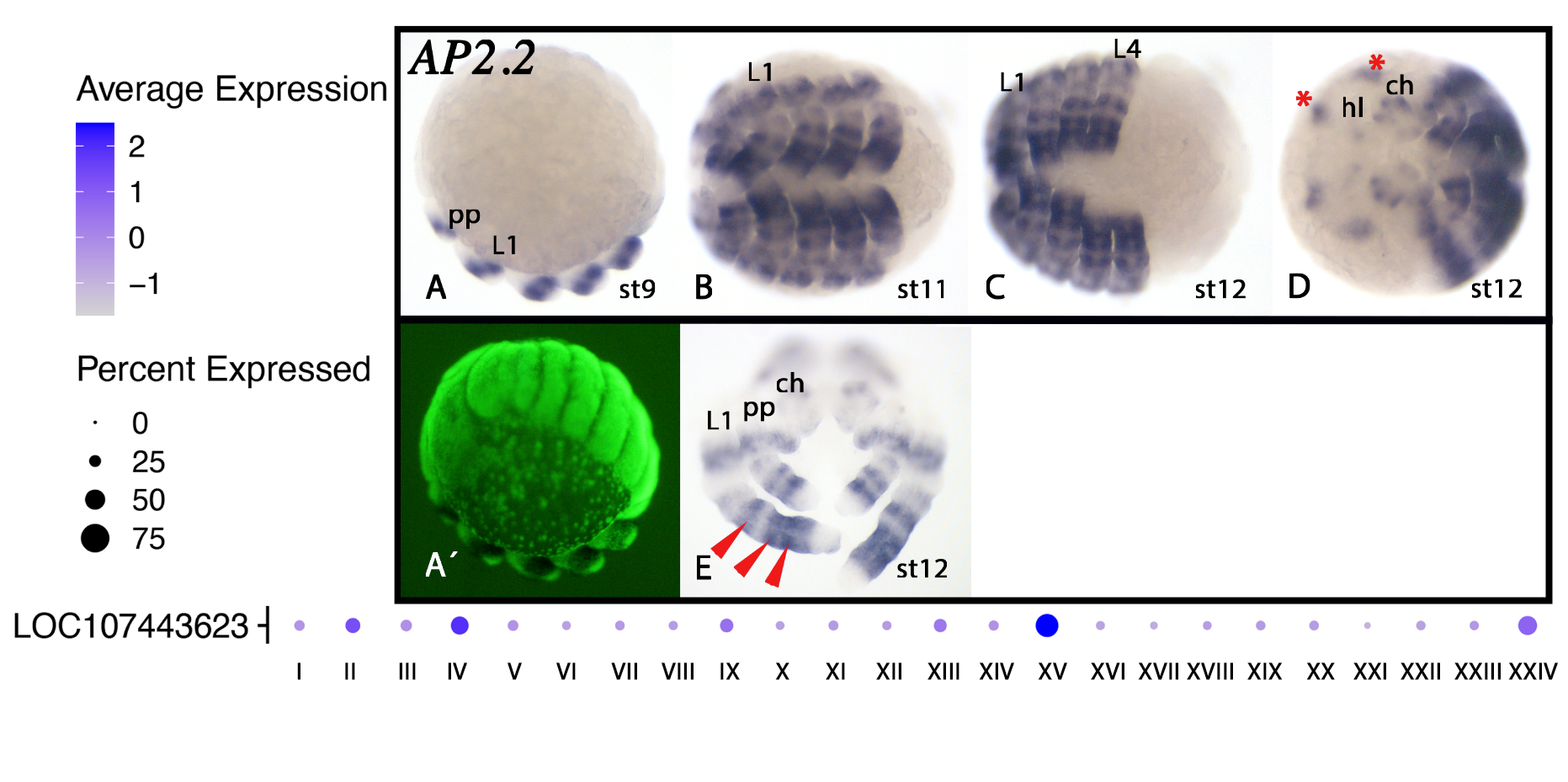

Supplement: Supplementary file 38 — Additional file 38. [file 12864_2023_9898_MOESM38_ESM.tif]

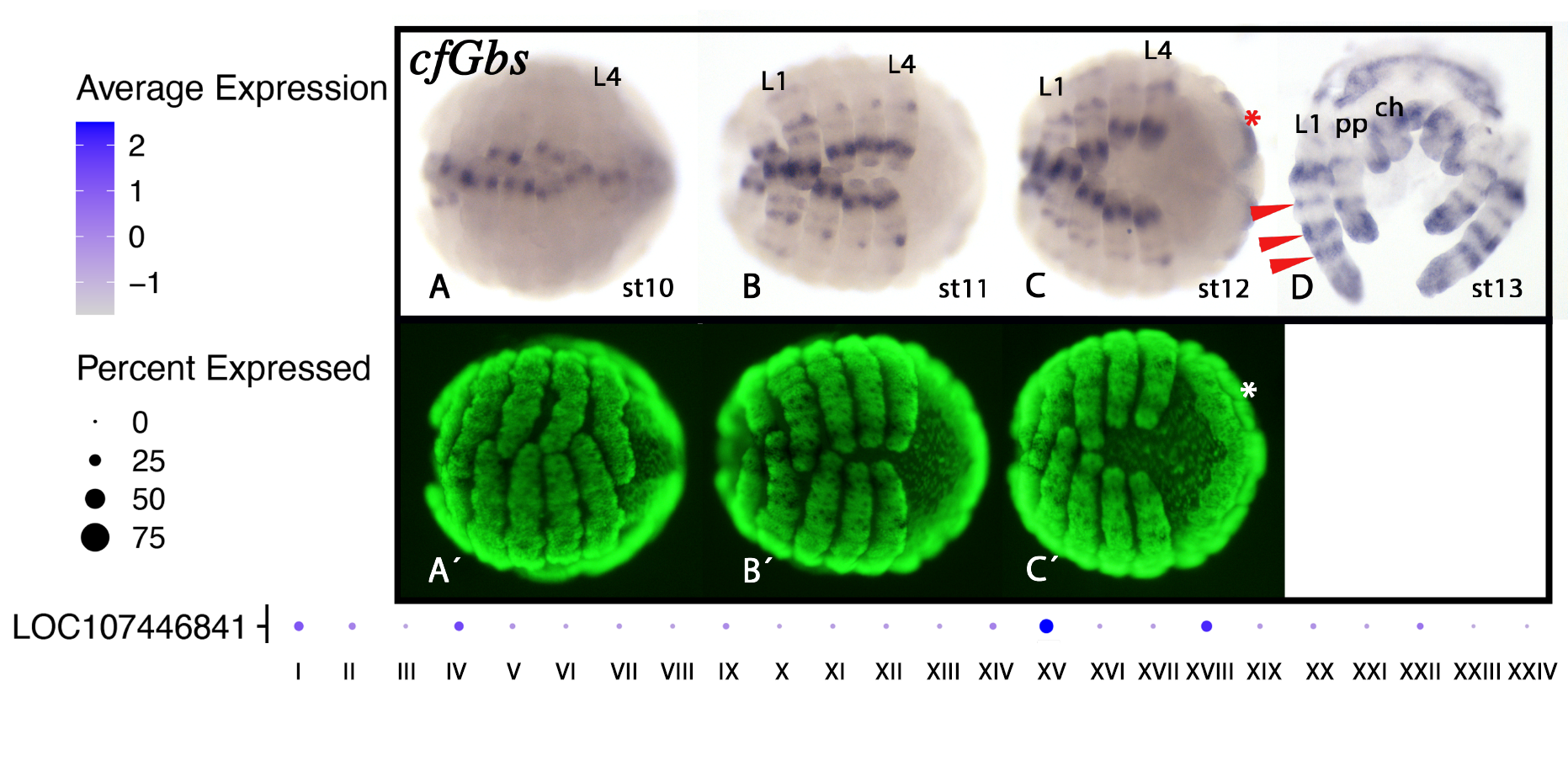

Supplement: Supplementary file 39 — Additional file 39. [file 12864_2023_9898_MOESM39_ESM.tif]

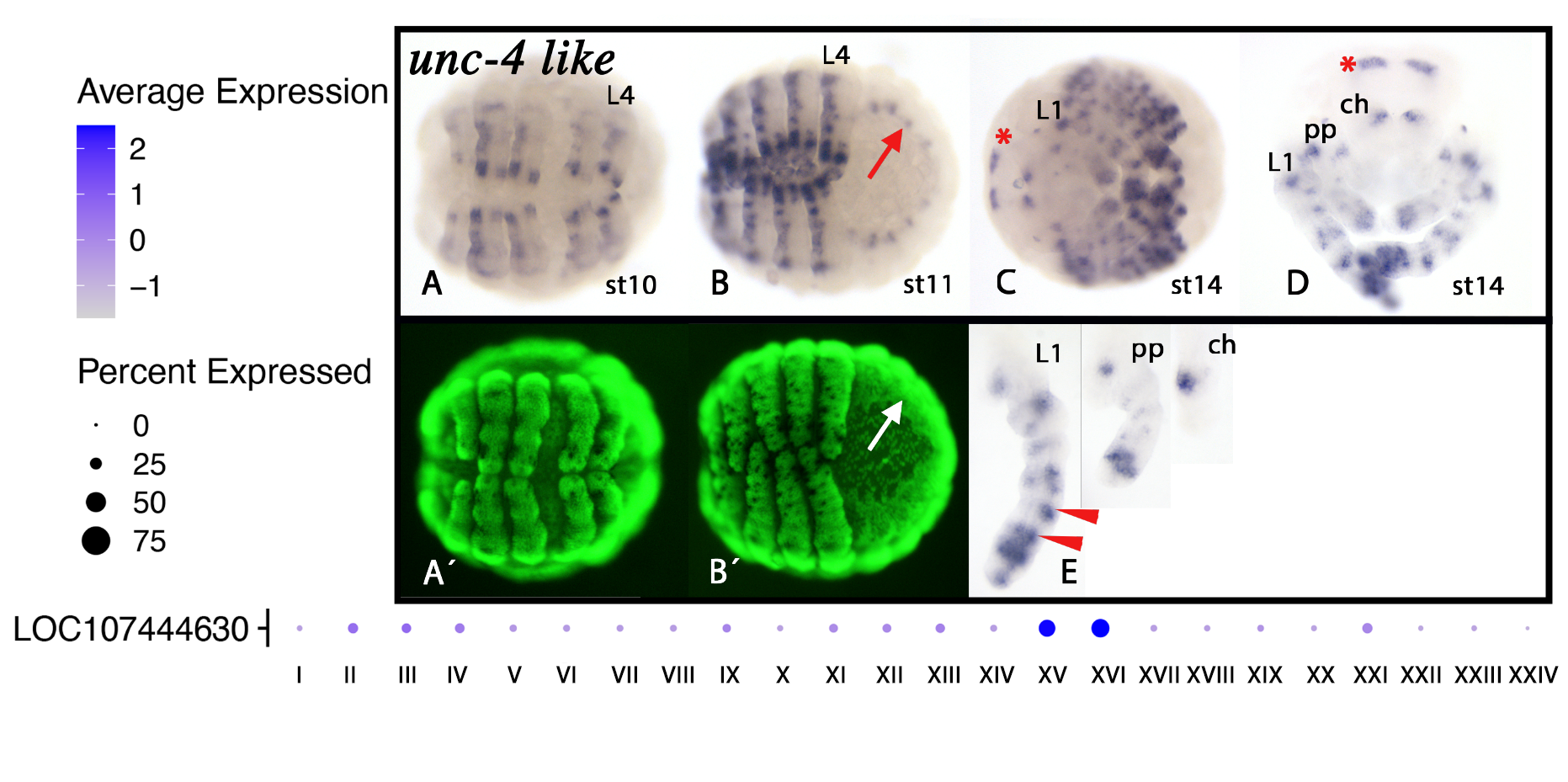

Supplement: Supplementary file 40 — Additional file 40. [file 12864_2023_9898_MOESM40_ESM.tif]

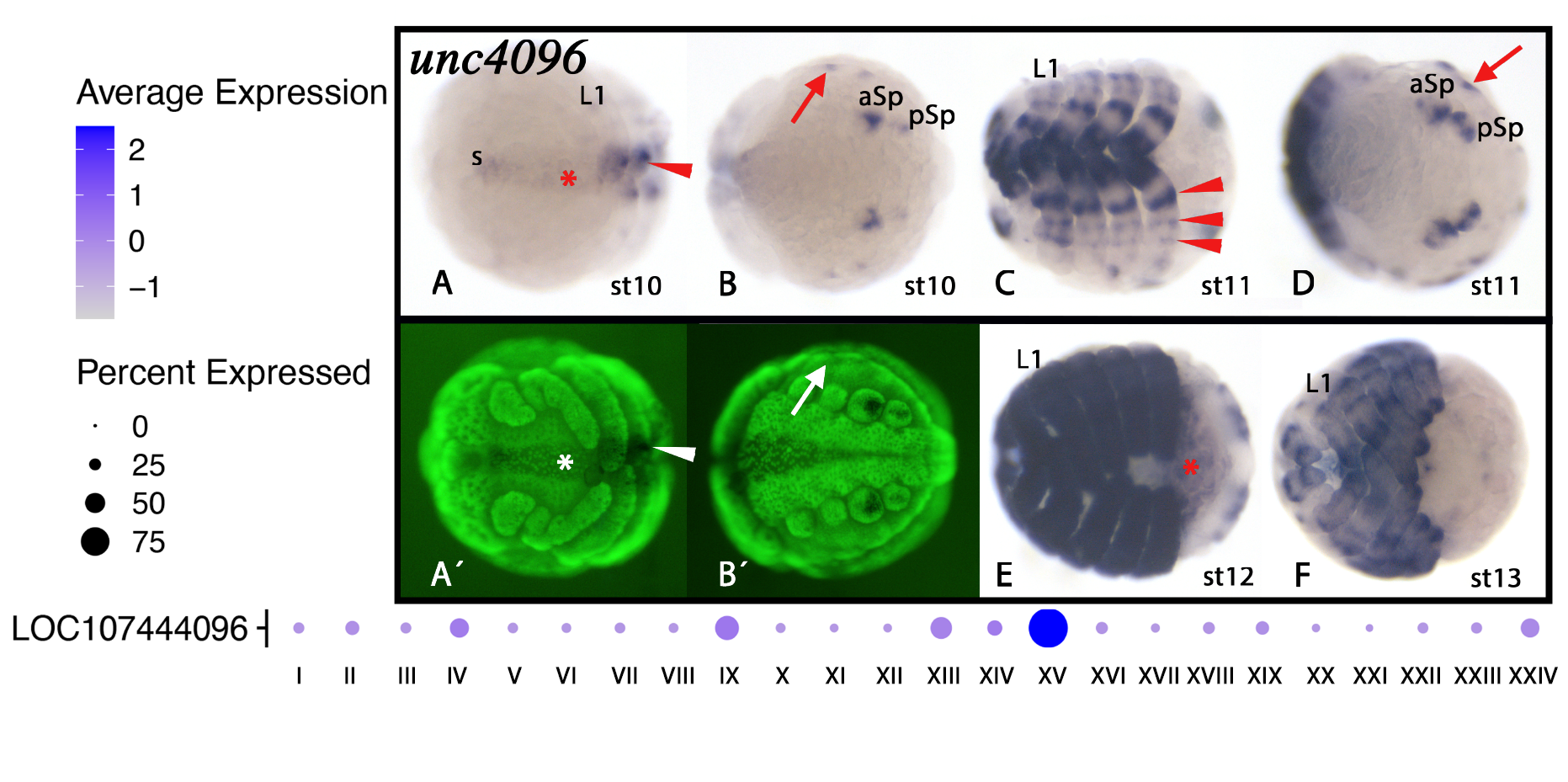

Supplement: Supplementary file 41 — Additional file 41. [file 12864_2023_9898_MOESM41_ESM.tif]

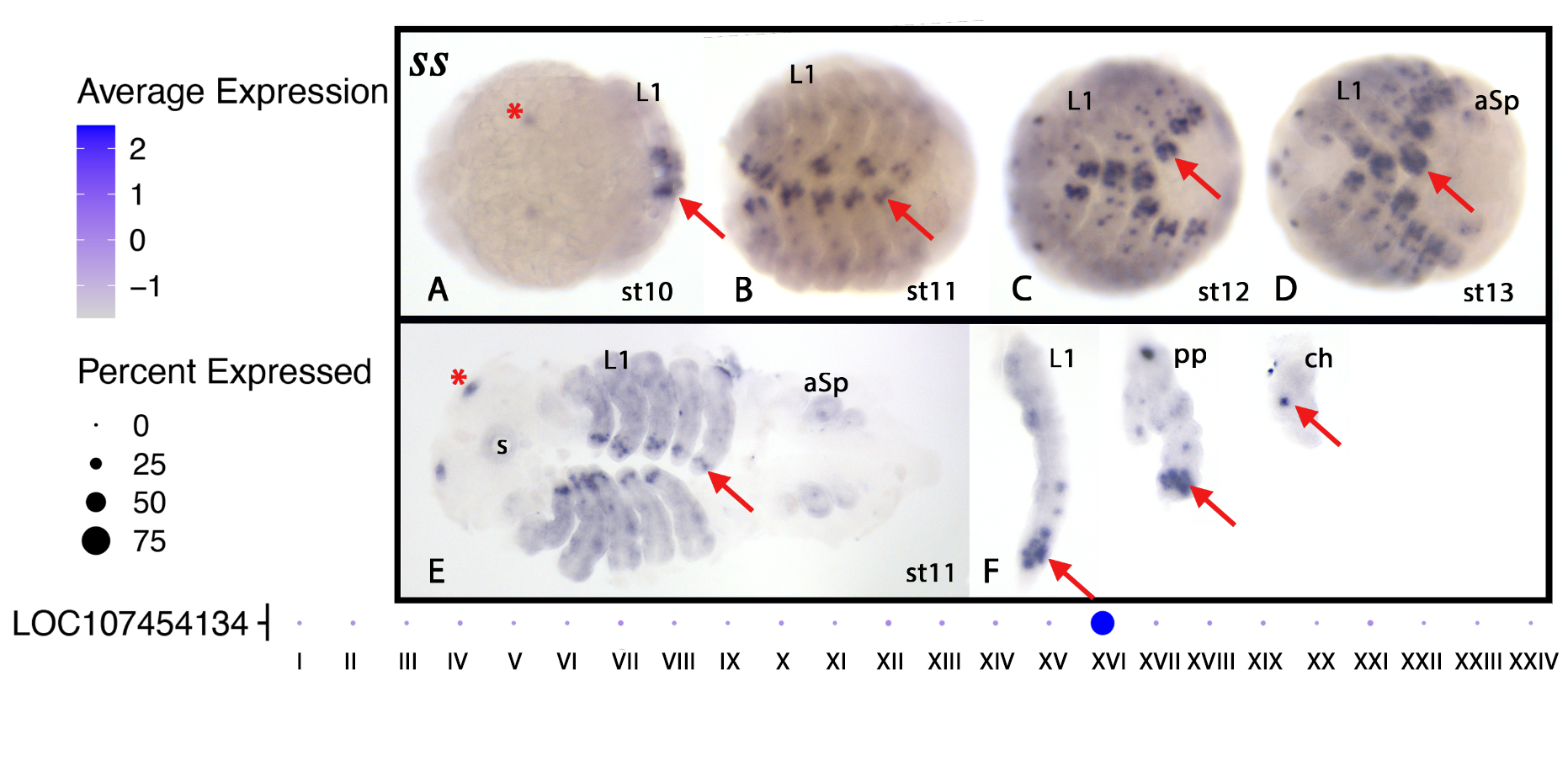

Supplement: Supplementary file 42 — Additional file 42. [file 12864_2023_9898_MOESM42_ESM.tif]

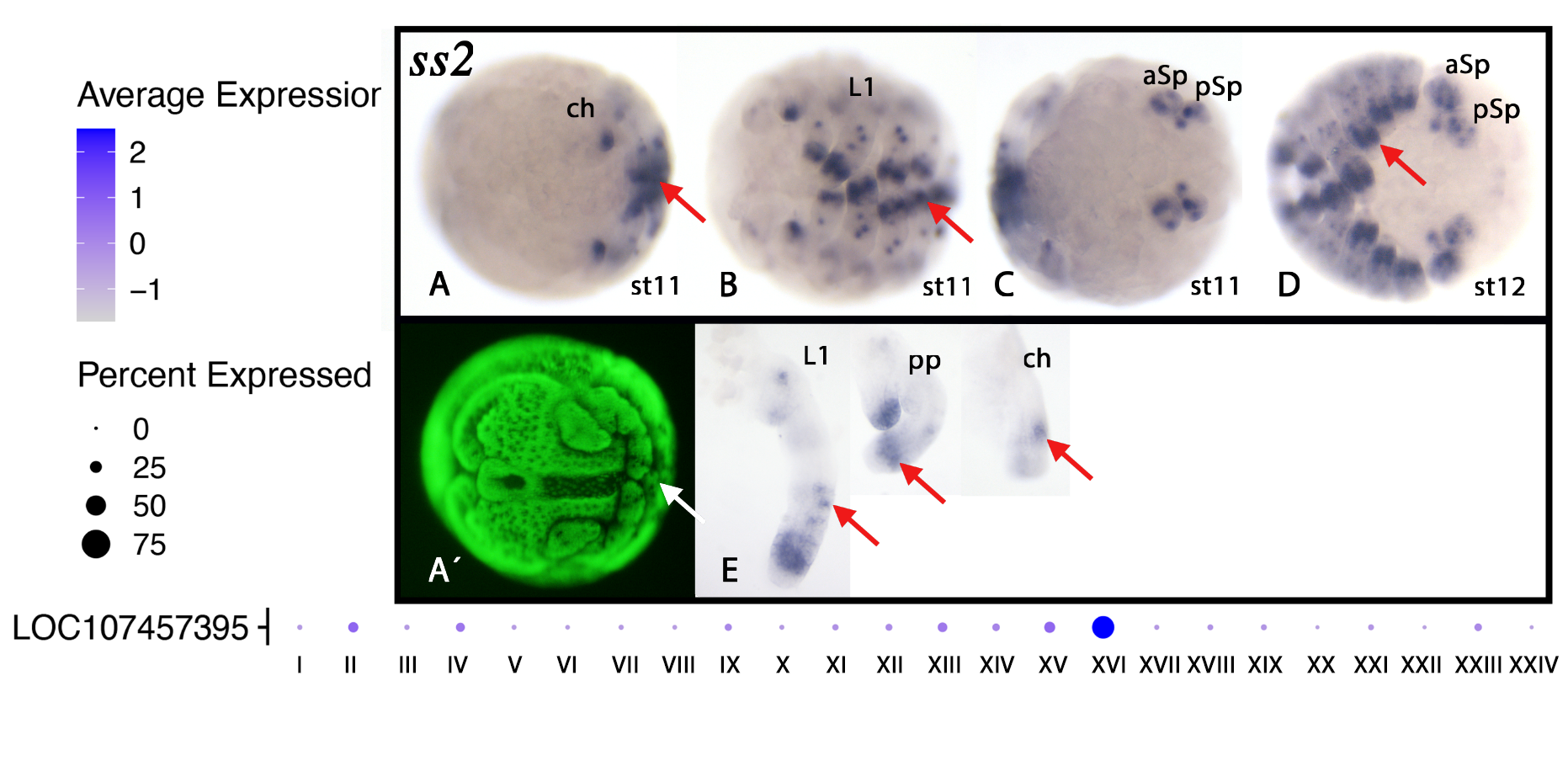

Supplement: Supplementary file 43 — Additional file 43. [file 12864_2023_9898_MOESM43_ESM.tif]

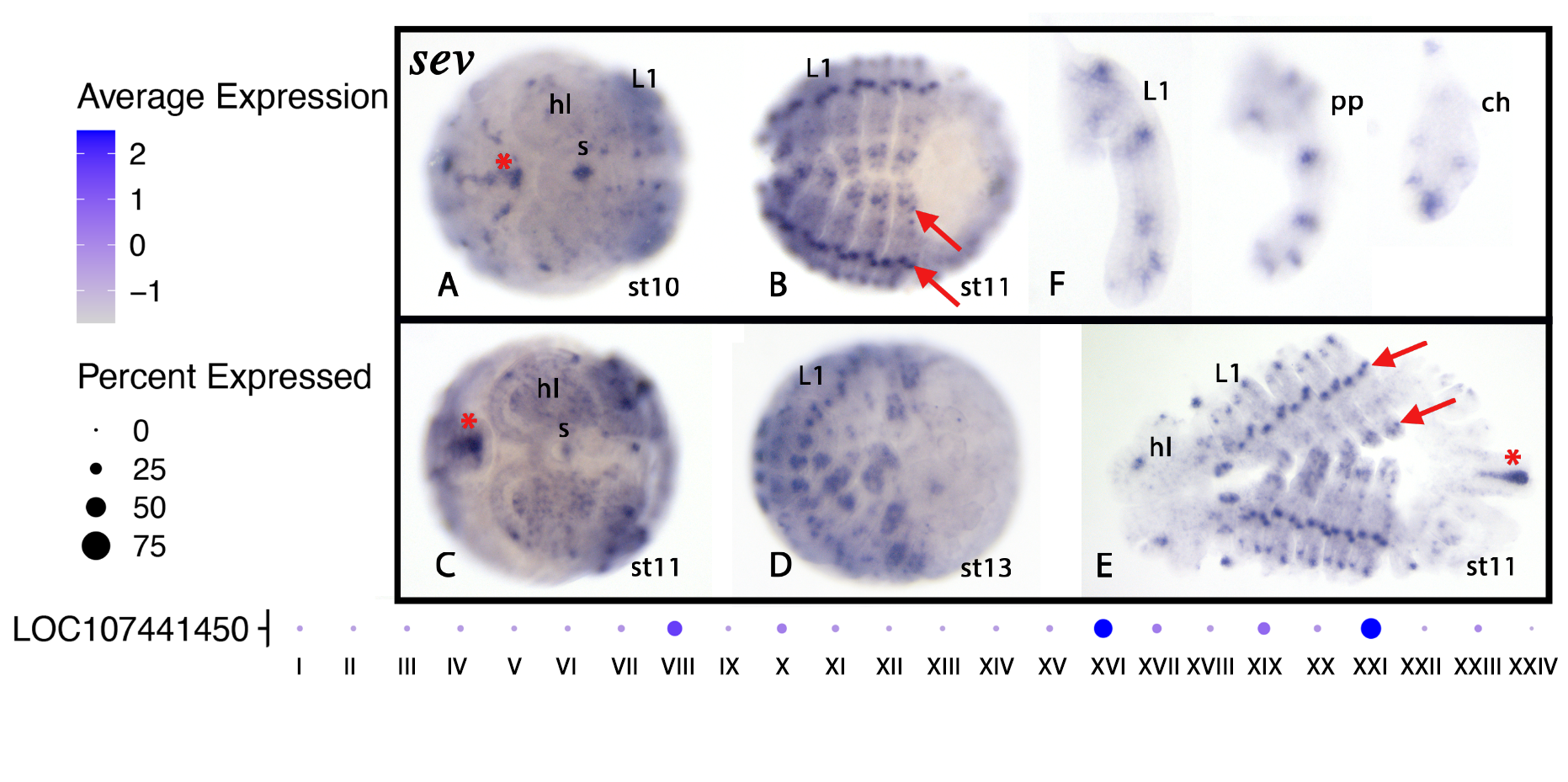

Supplement: Supplementary file 44 — Additional file 44. [file 12864_2023_9898_MOESM44_ESM.tif]

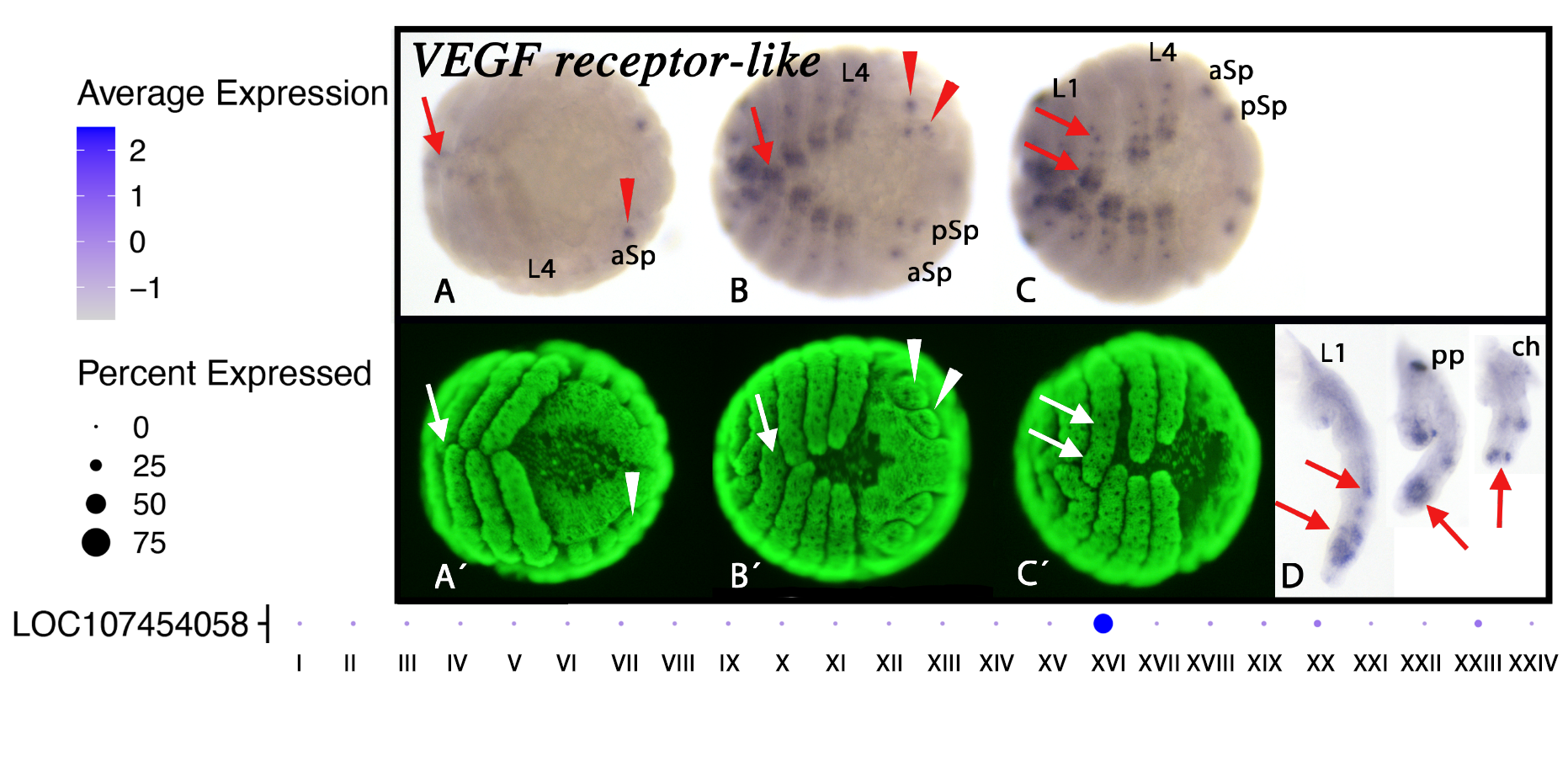

Supplement: Supplementary file 45 — Additional file 45. [file 12864_2023_9898_MOESM45_ESM.tif]

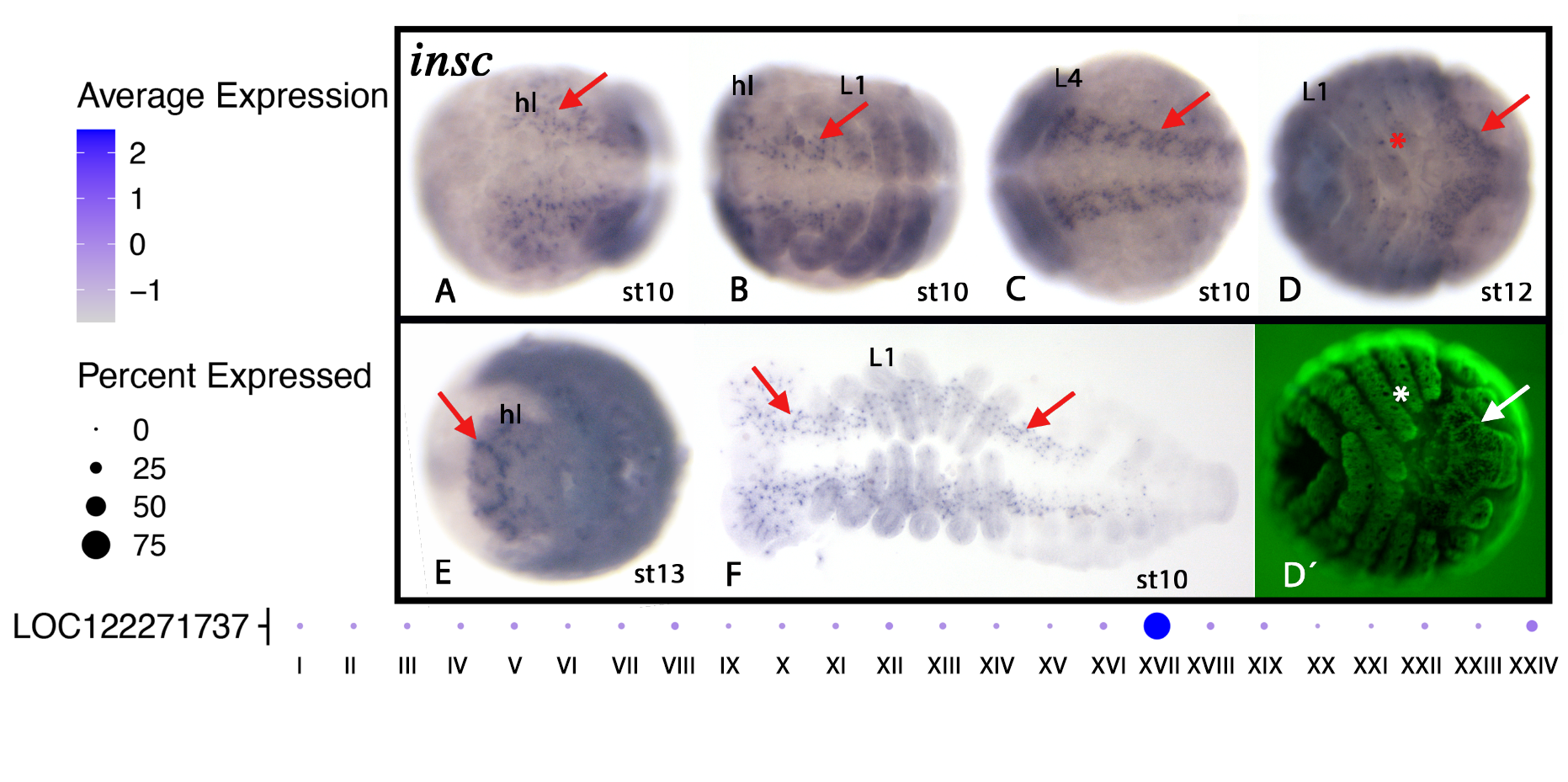

Supplement: Supplementary file 46 — Additional file 46. [file 12864_2023_9898_MOESM46_ESM.tif]

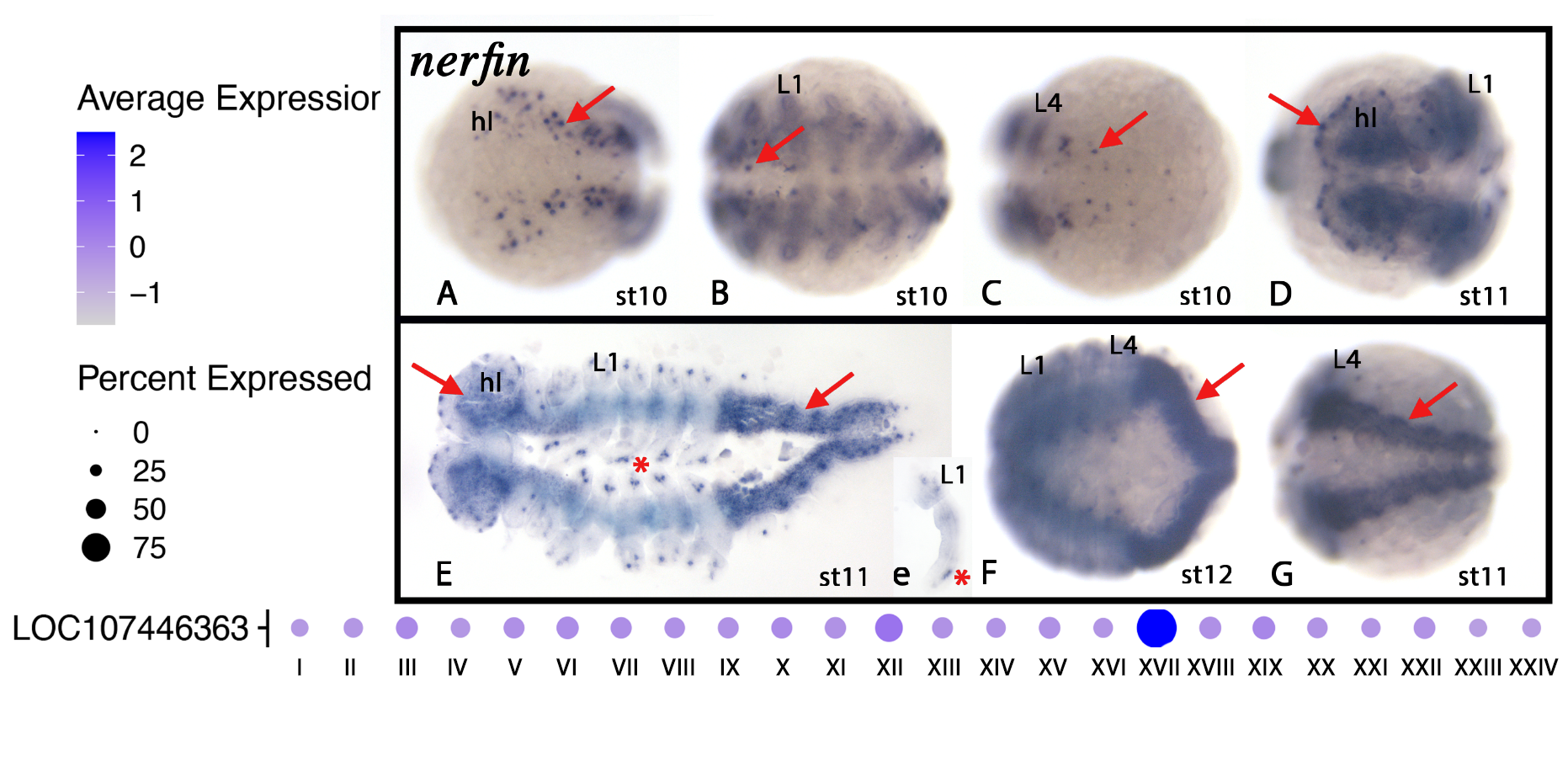

Supplement: Supplementary file 47 — Additional file 47. [file 12864_2023_9898_MOESM47_ESM.tif]

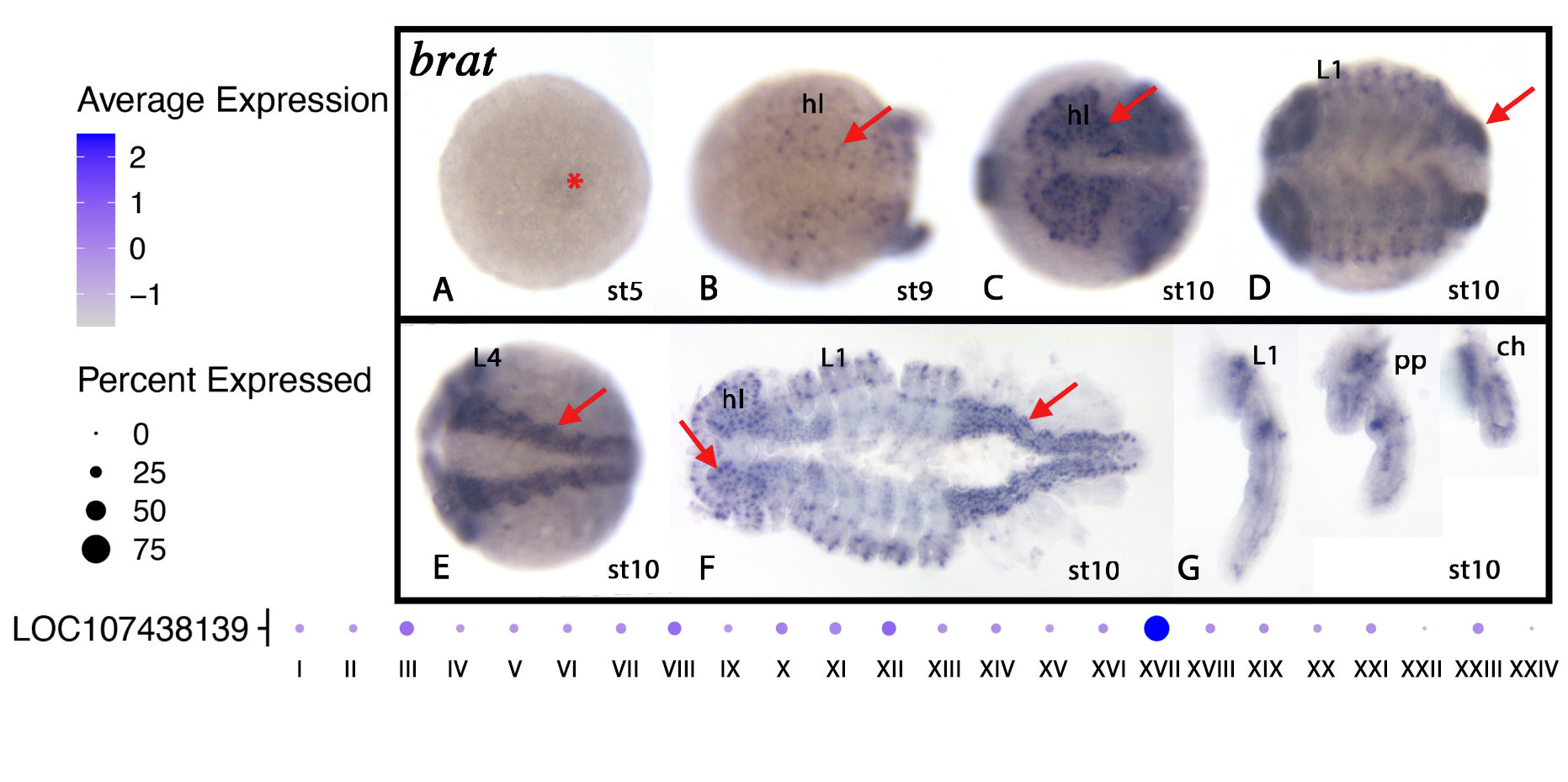

Supplement: Supplementary file 48 — Additional file 48. [file 12864_2023_9898_MOESM48_ESM.tif]

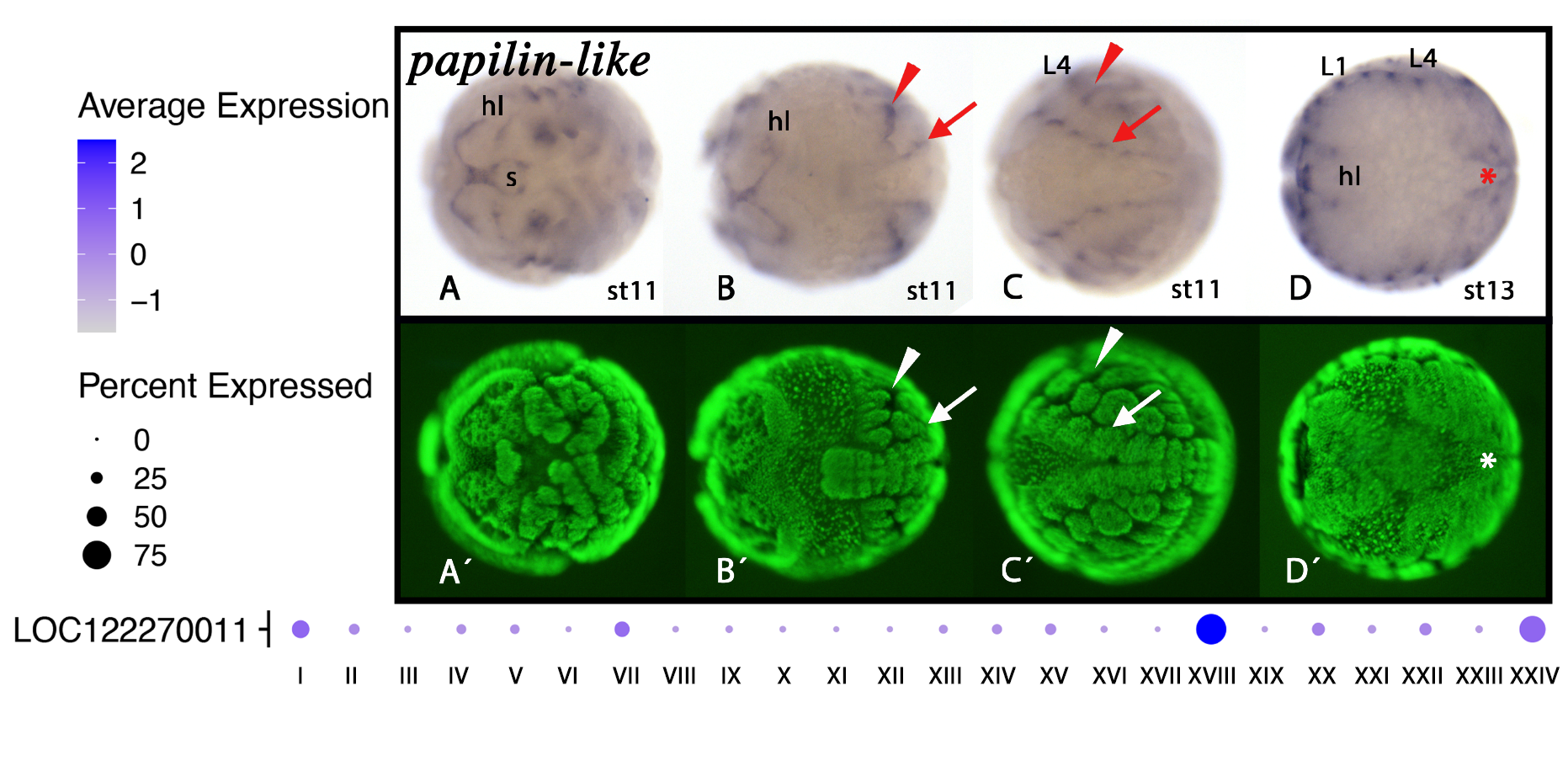

Supplement: Supplementary file 49 — Additional file 49. [file 12864_2023_9898_MOESM49_ESM.tif]

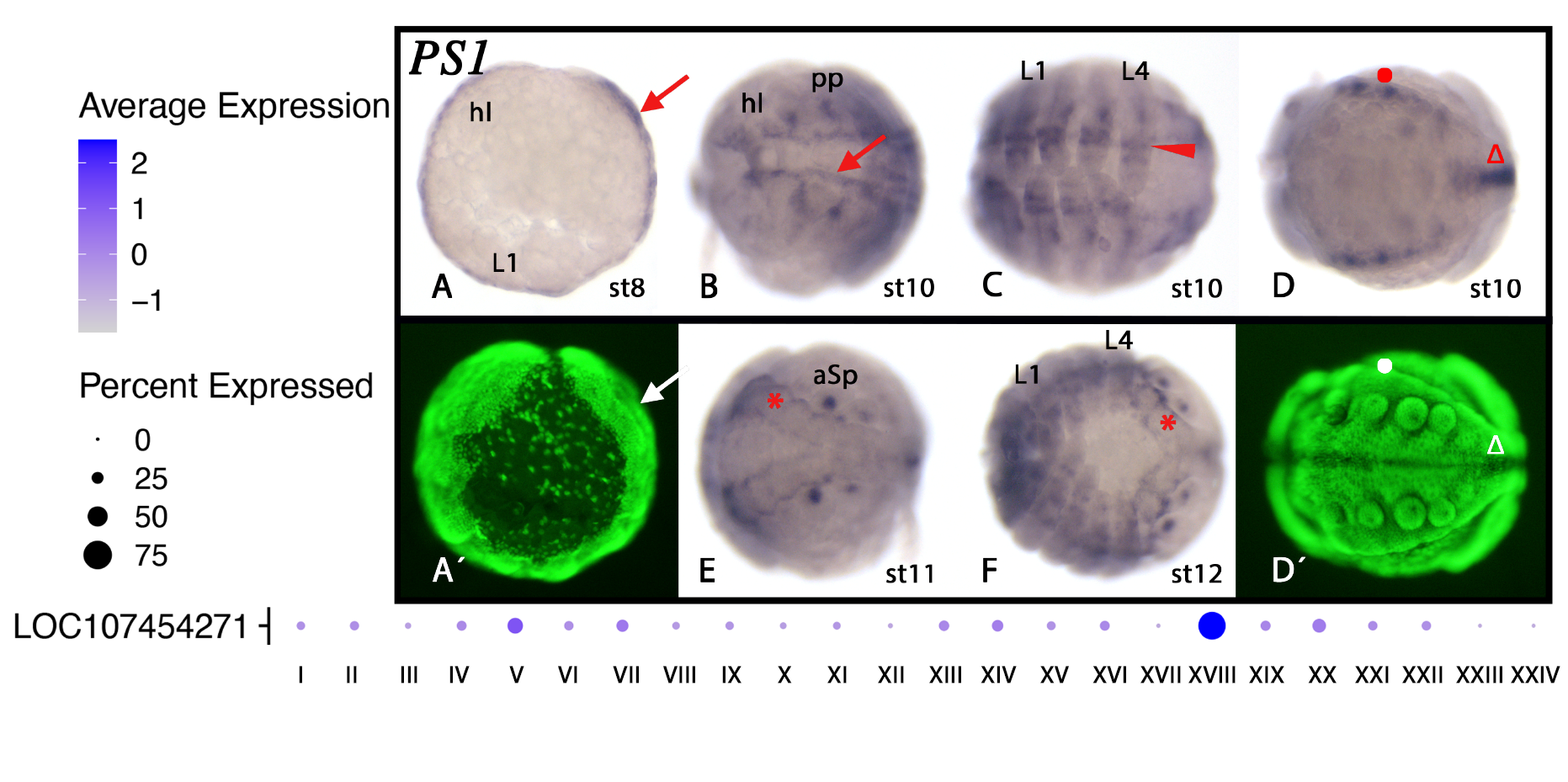

Supplement: Supplementary file 50 — Additional file 50. [file 12864_2023_9898_MOESM50_ESM.tif]

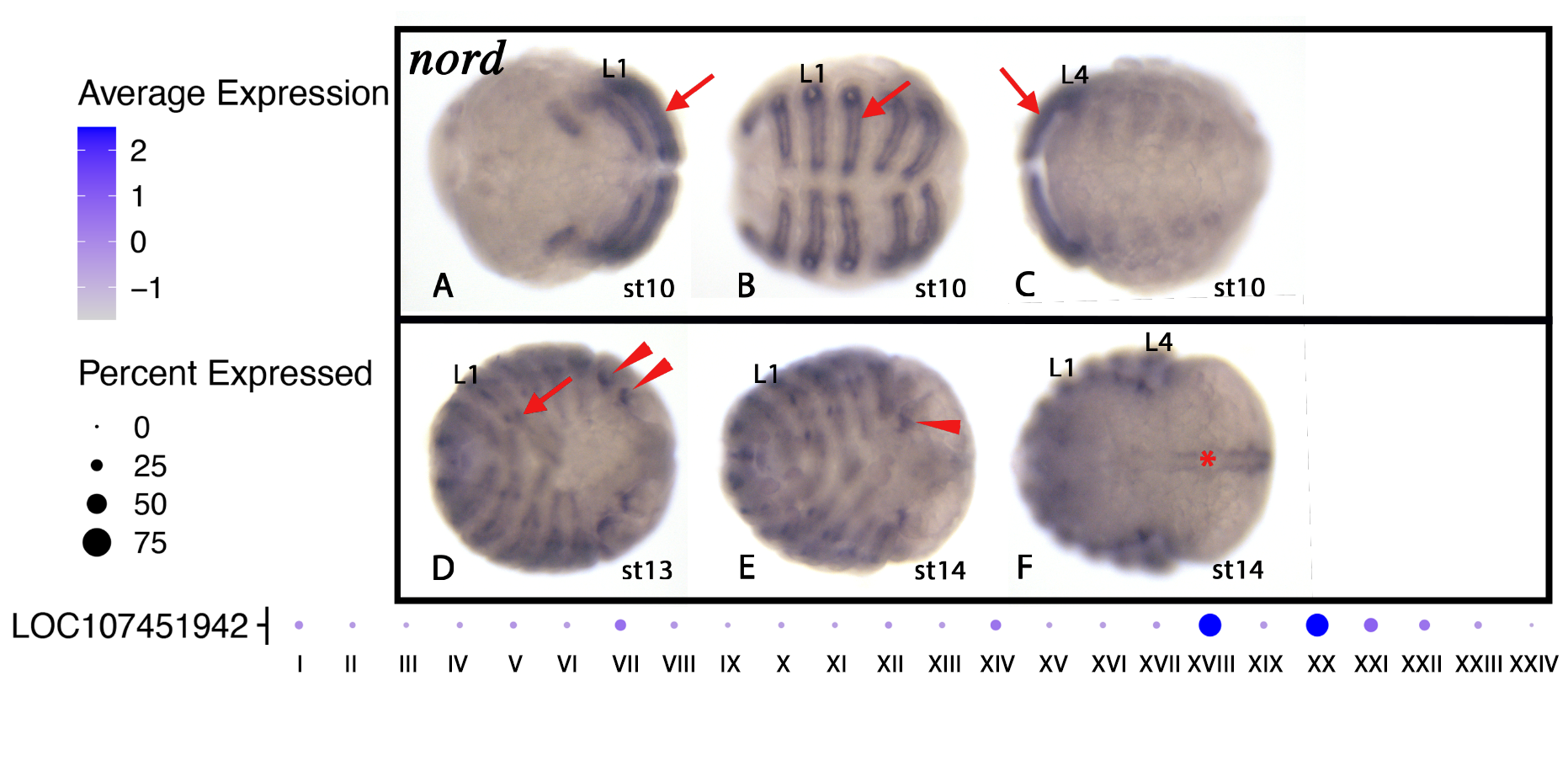

Supplement: Supplementary file 51 — Additional file 51. [file 12864_2023_9898_MOESM51_ESM.tif]

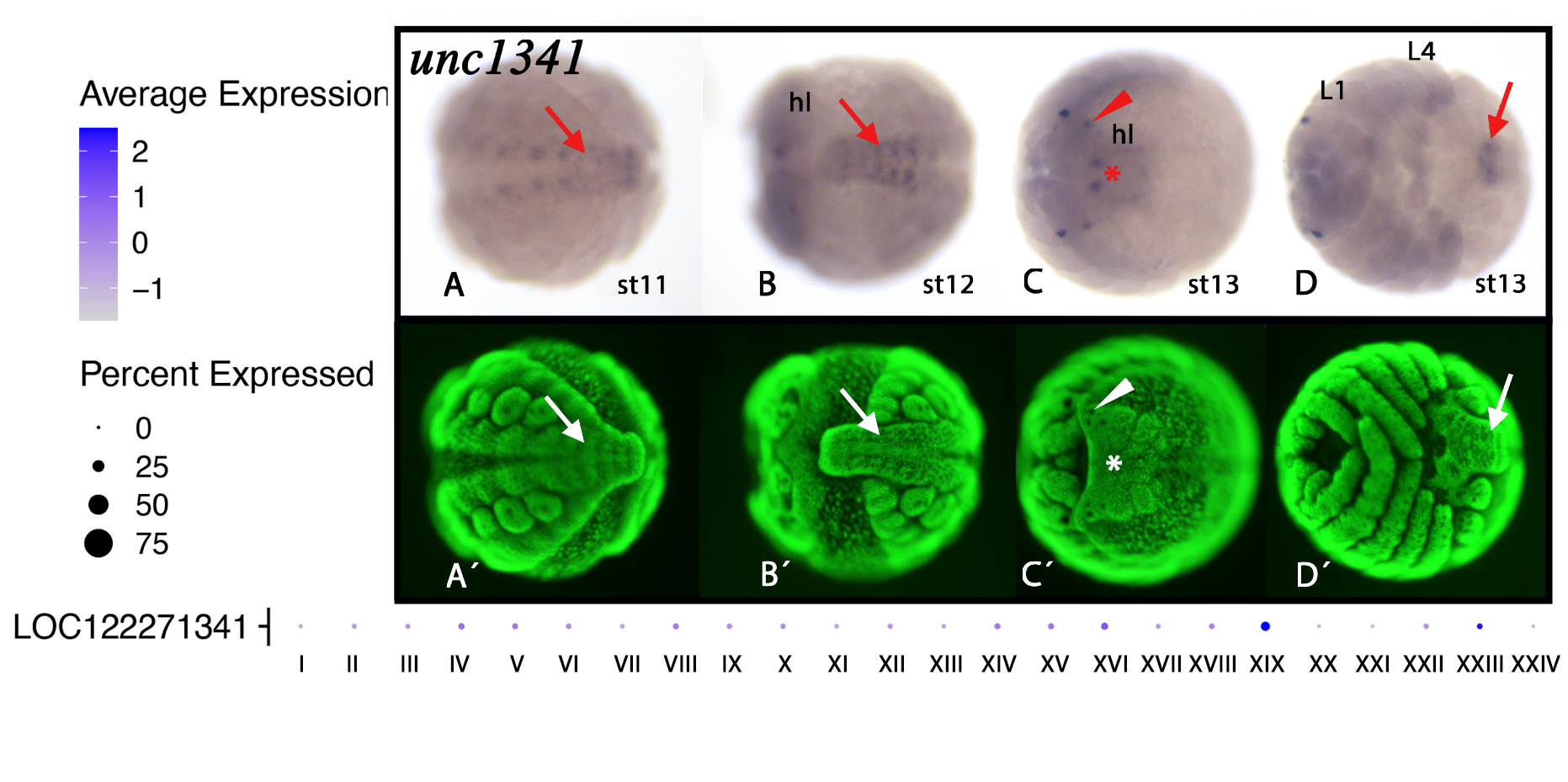

Supplement: Supplementary file 52 — Additional file 52. [file 12864_2023_9898_MOESM52_ESM.tif]

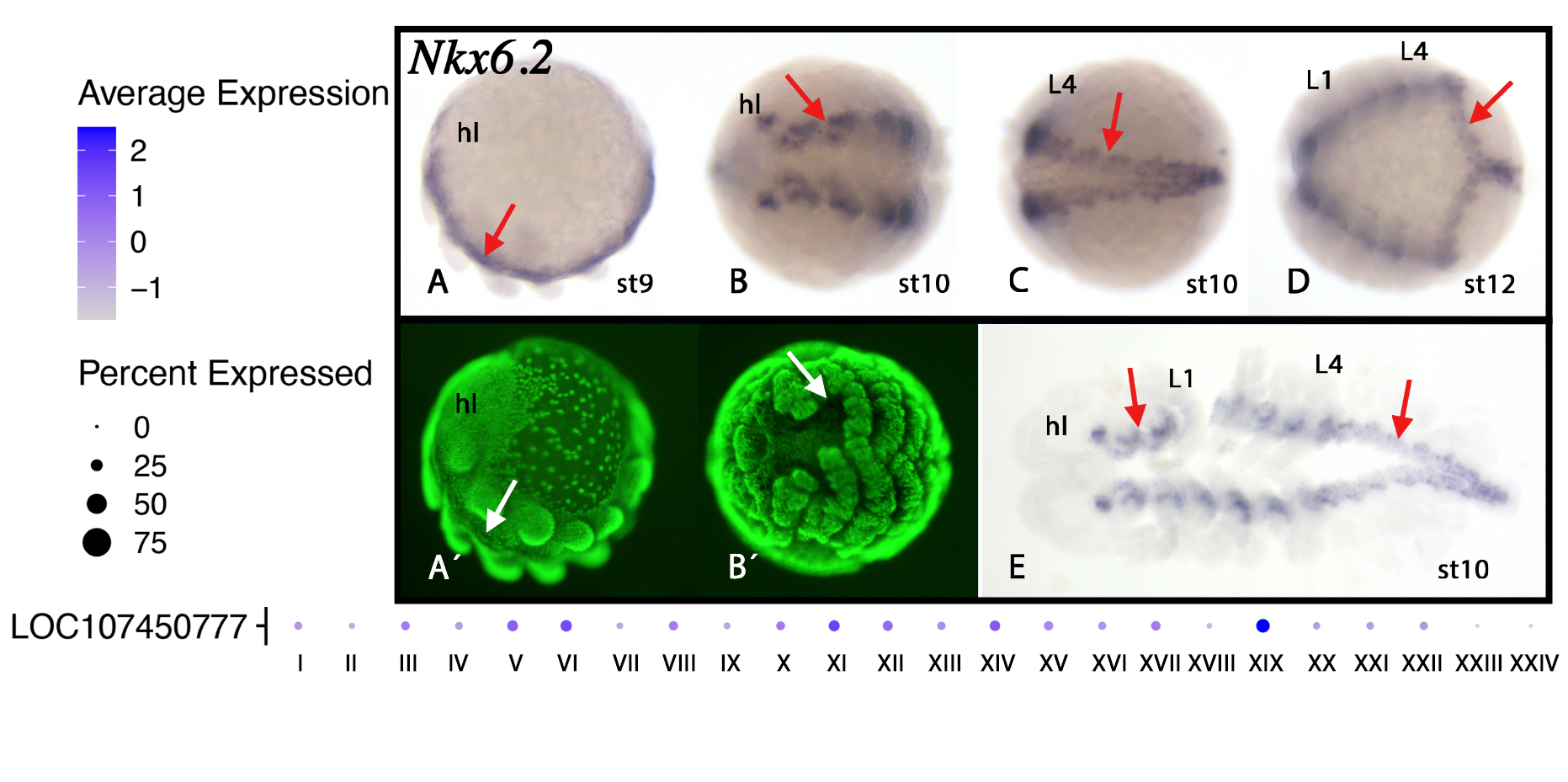

Supplement: Supplementary file 53 — Additional file 53. [file 12864_2023_9898_MOESM53_ESM.tif]

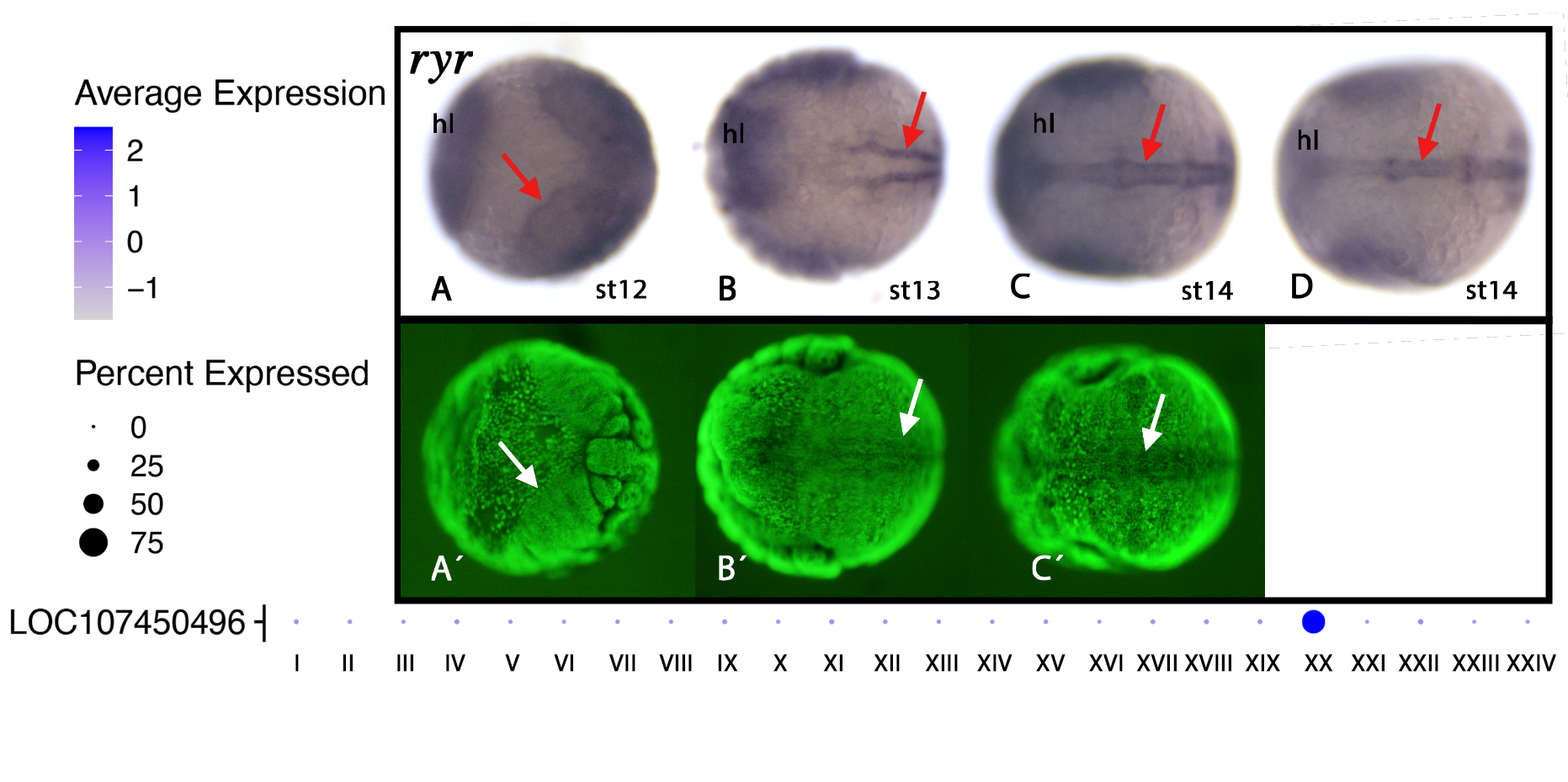

Supplement: Supplementary file 54 — Additional file 54. [file 12864_2023_9898_MOESM54_ESM.tif]

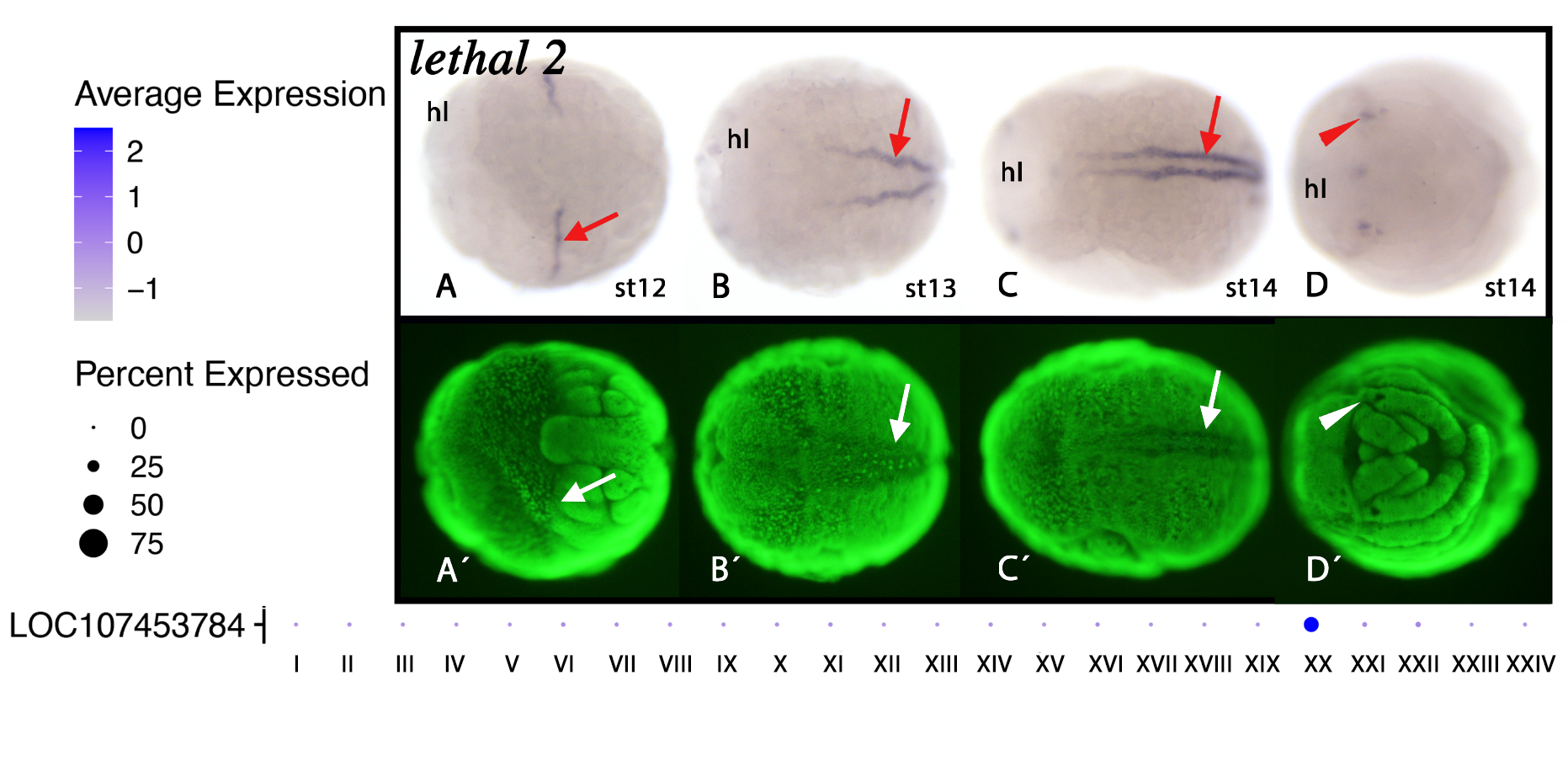

Supplement: Supplementary file 55 — Additional file 55. [file 12864_2023_9898_MOESM55_ESM.tif]

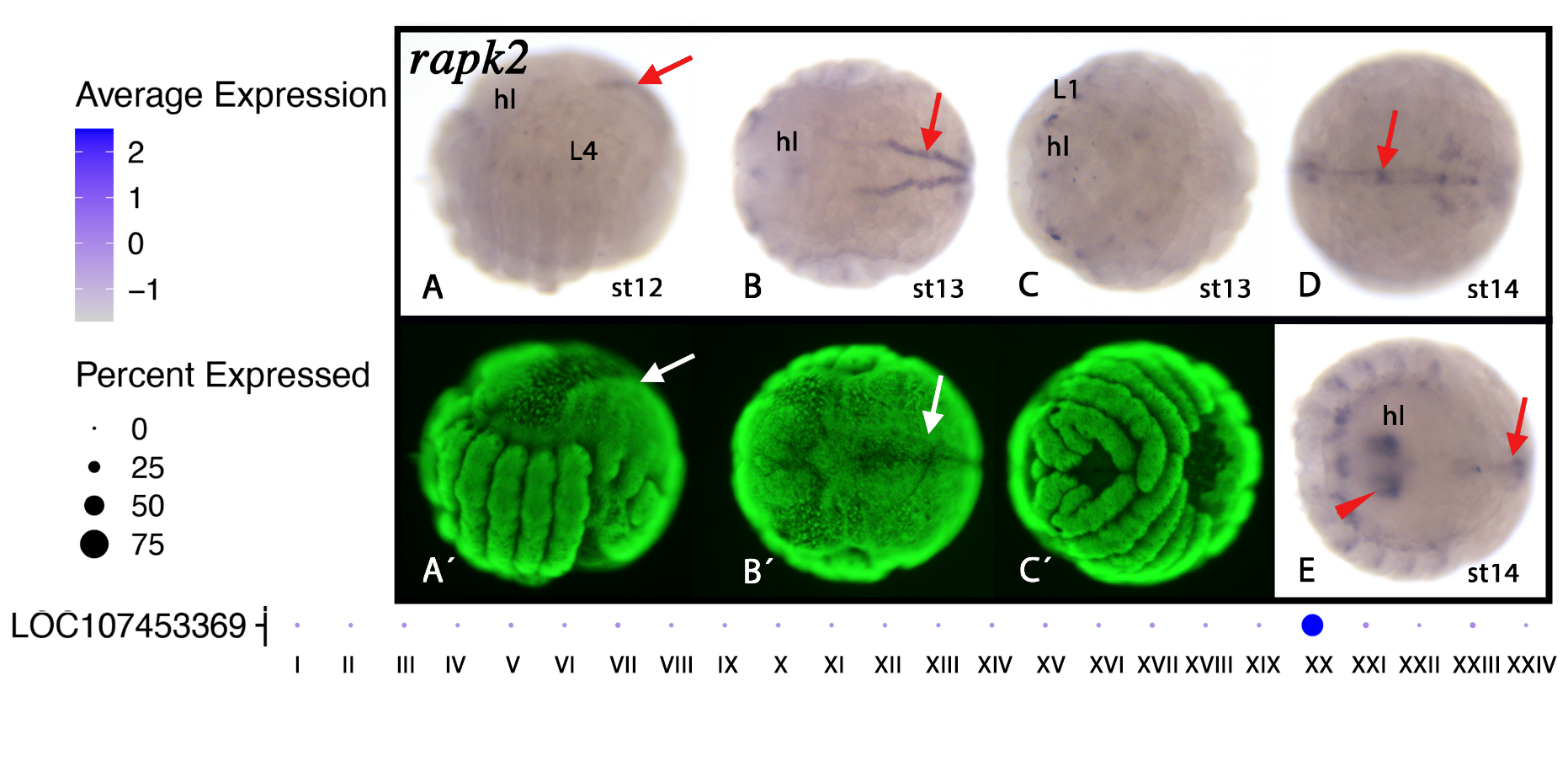

Supplement: Supplementary file 56 — Additional file 56. [file 12864_2023_9898_MOESM56_ESM.tif]

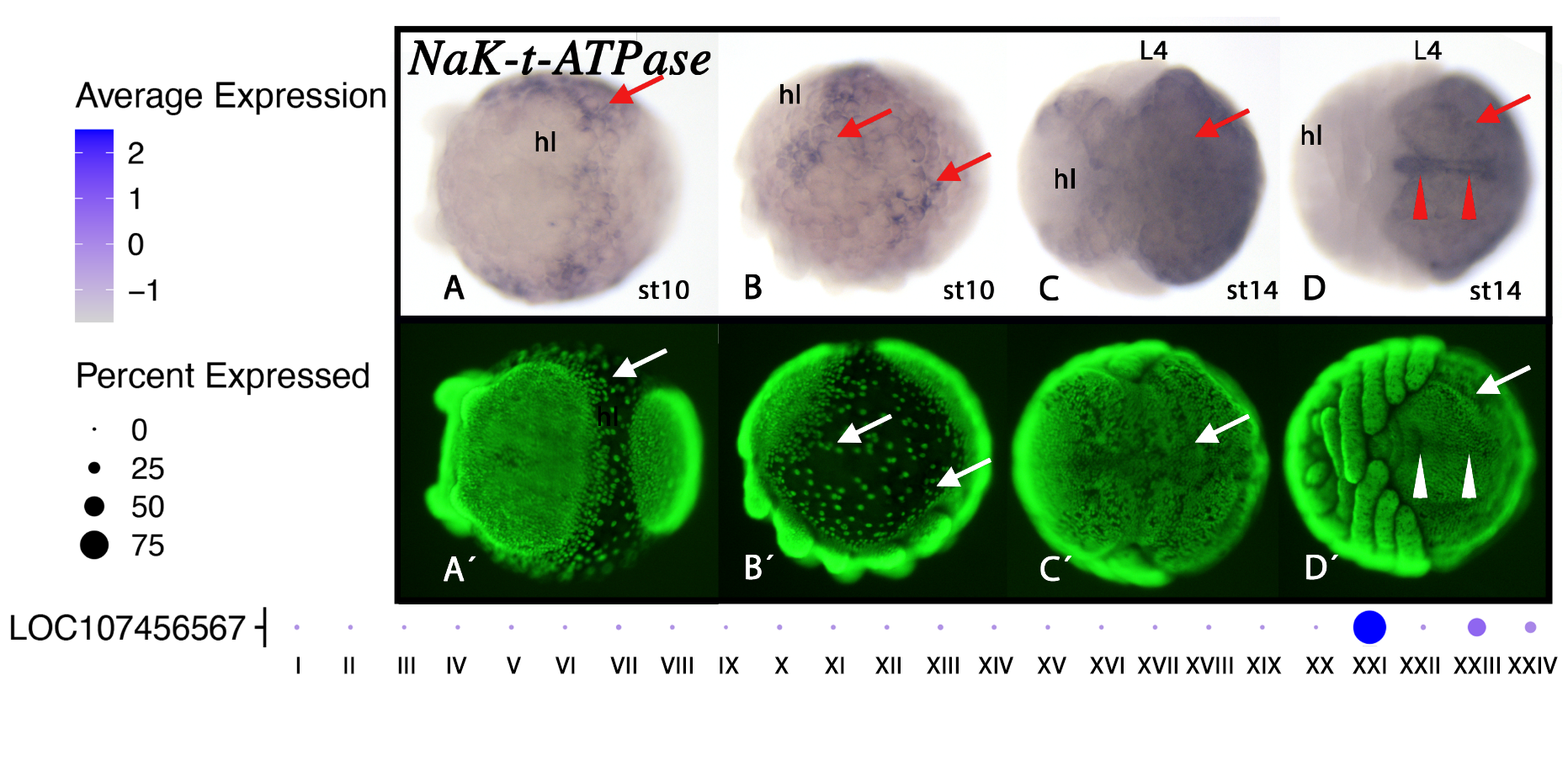

Supplement: Supplementary file 57 — Additional file 57. [file 12864_2023_9898_MOESM57_ESM.tif]

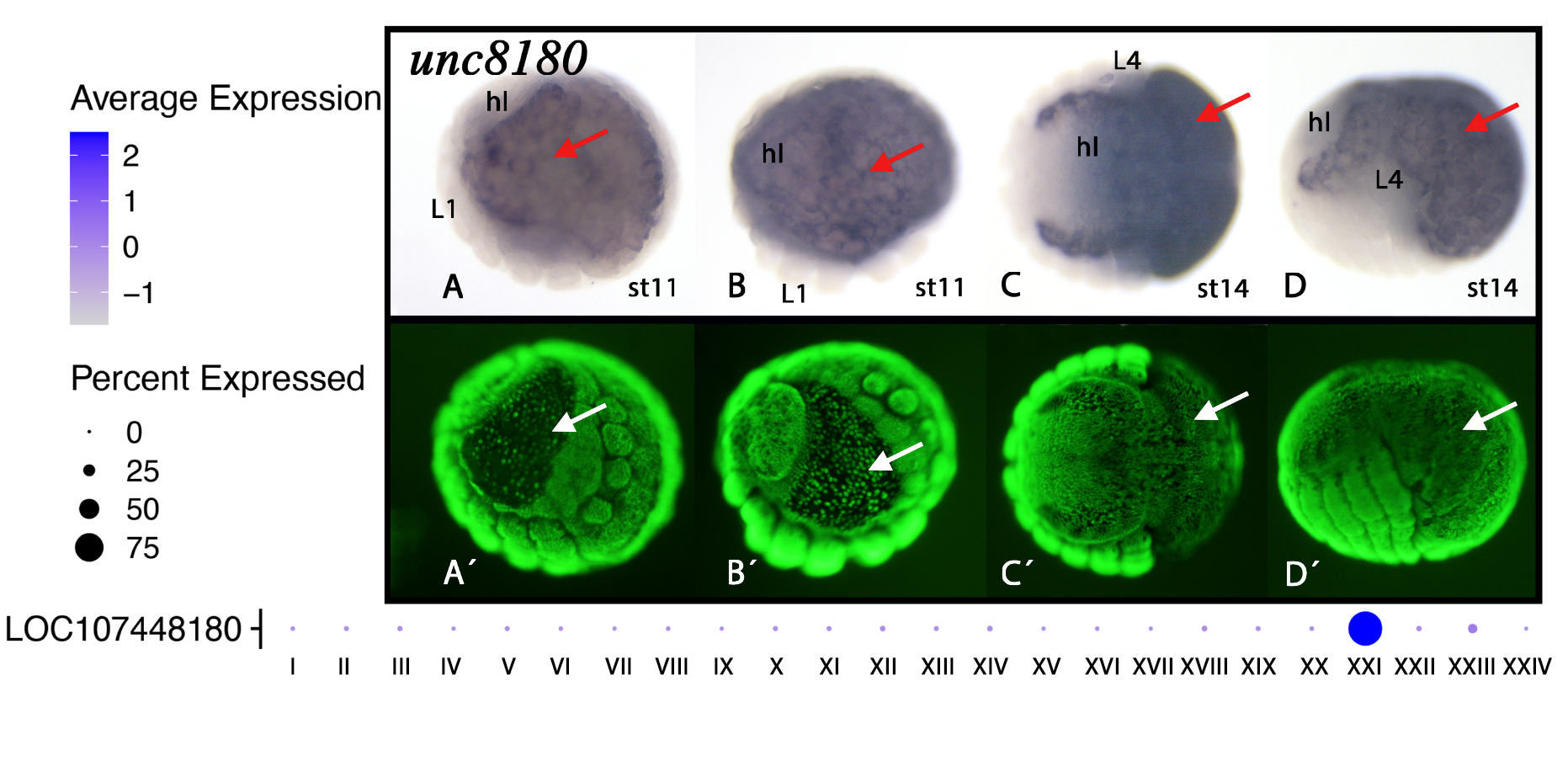

Supplement: Supplementary file 58 — Additional file 58. [file 12864_2023_9898_MOESM58_ESM.tif]

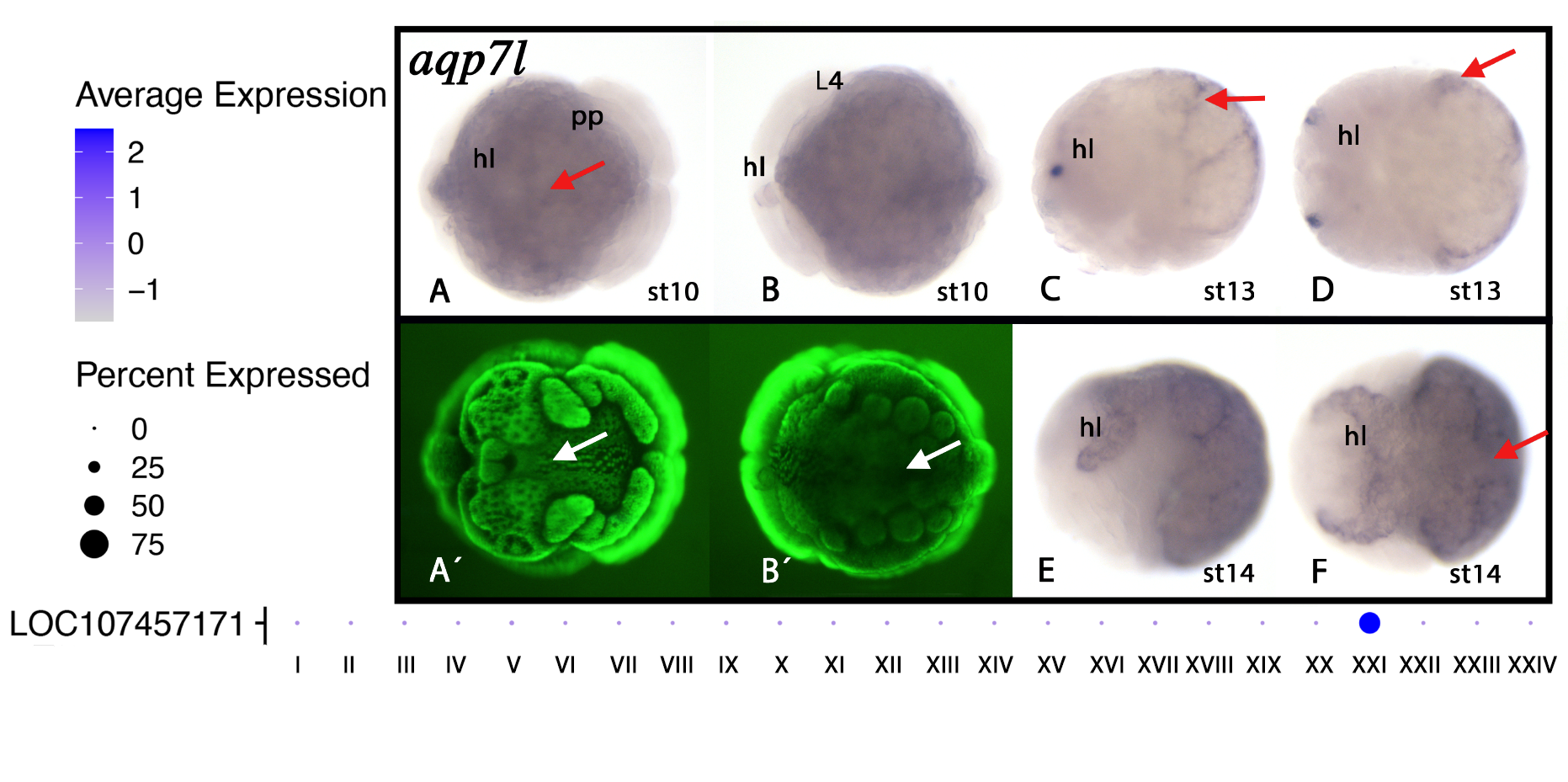

Supplement: Supplementary file 59 — Additional file 59. [file 12864_2023_9898_MOESM59_ESM.tif]

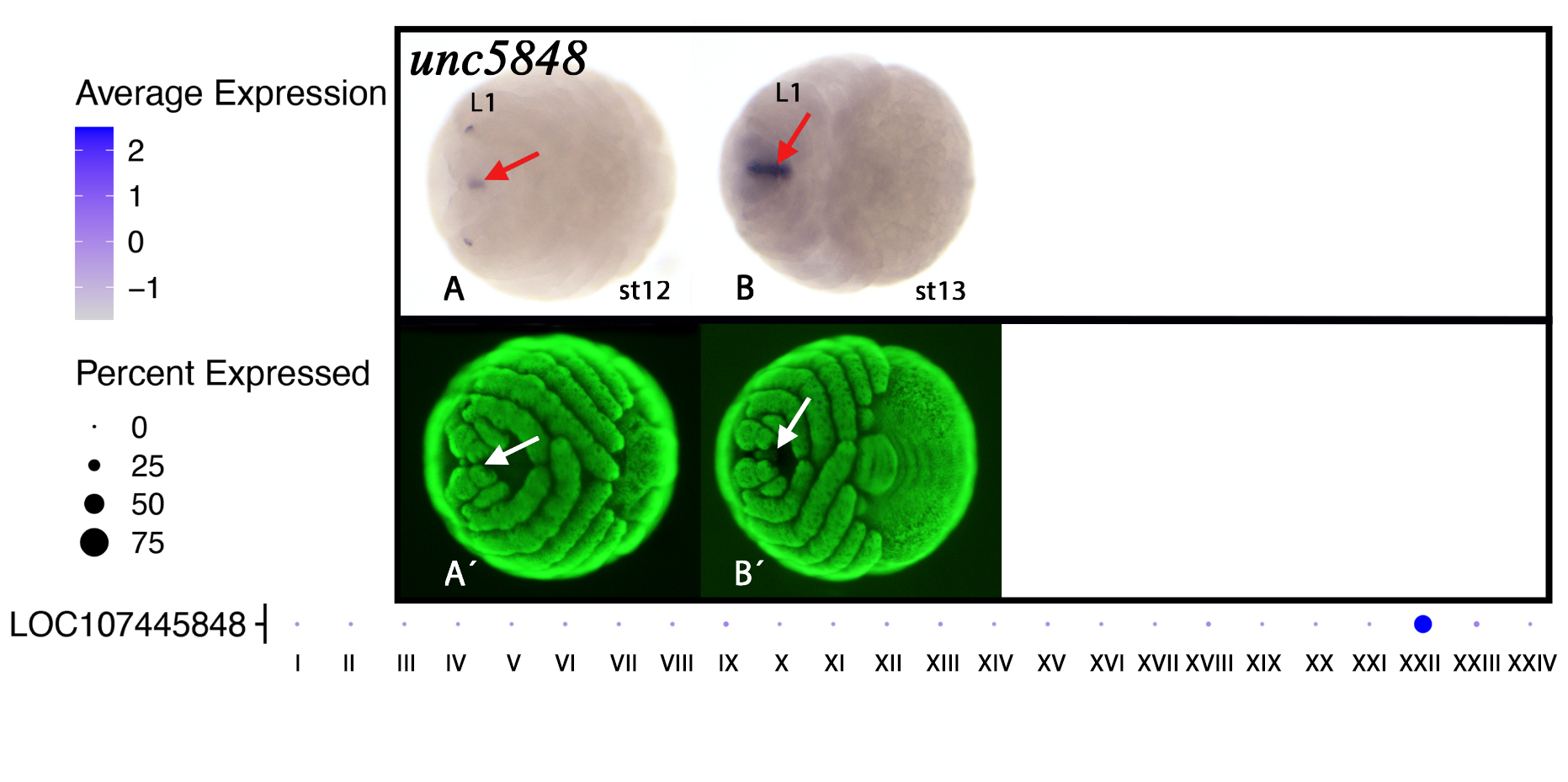

Supplement: Supplementary file 60 — Additional file 60. [file 12864_2023_9898_MOESM60_ESM.tif]

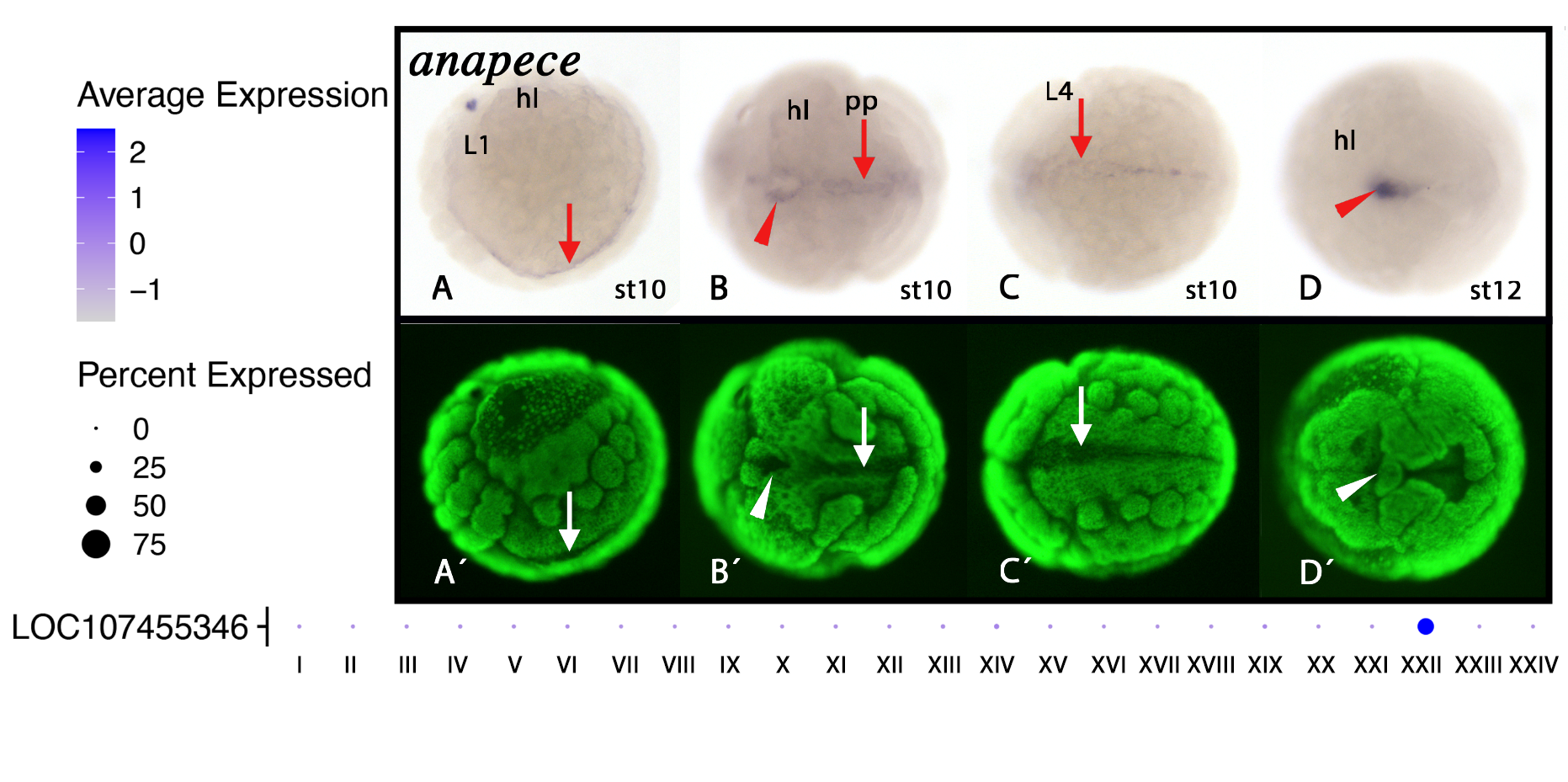

Supplement: Supplementary file 61 — Additional file 61. [file 12864_2023_9898_MOESM61_ESM.tif]

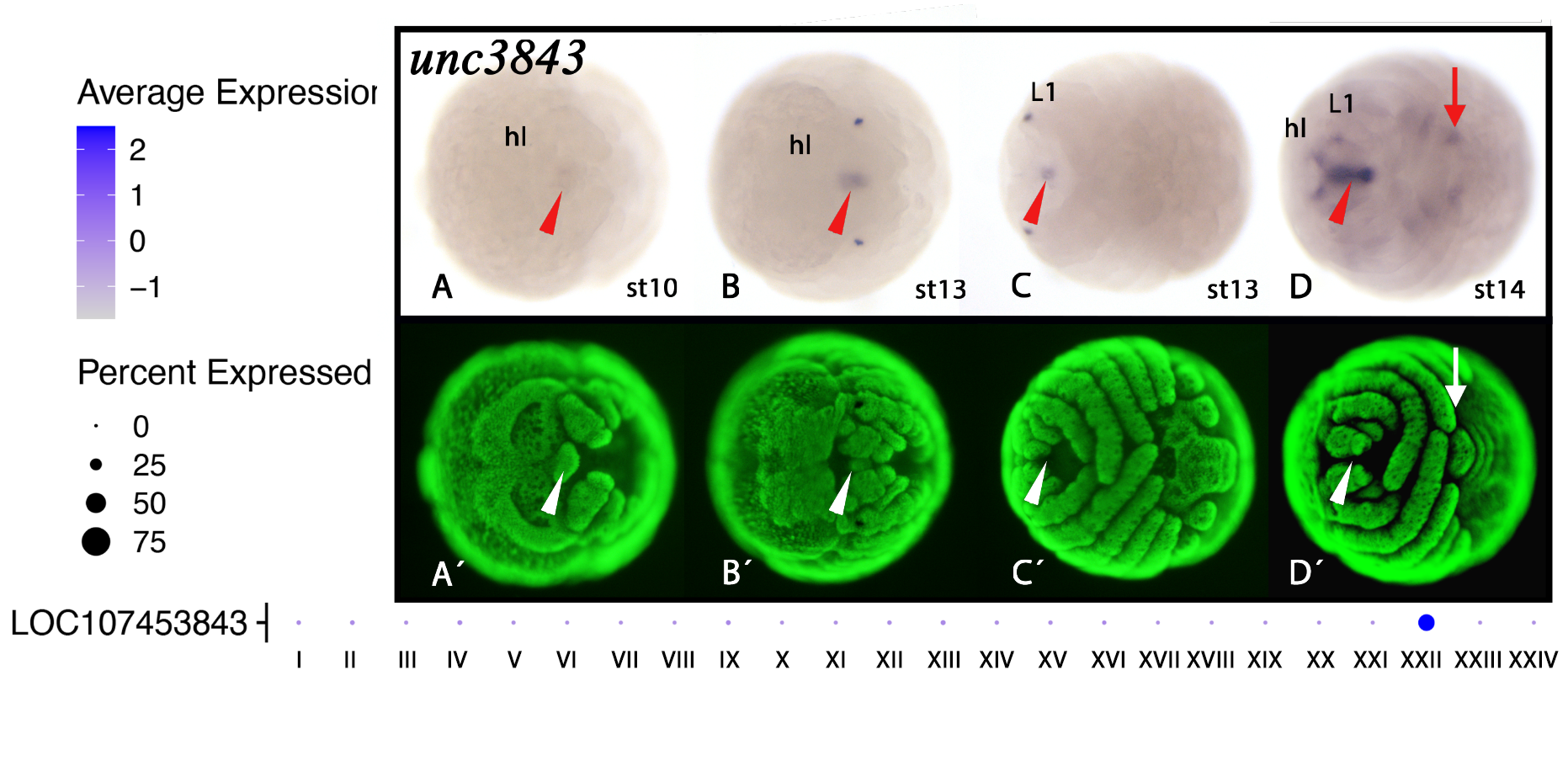

Supplement: Supplementary file 62 — Additional file 62. [file 12864_2023_9898_MOESM62_ESM.tif]

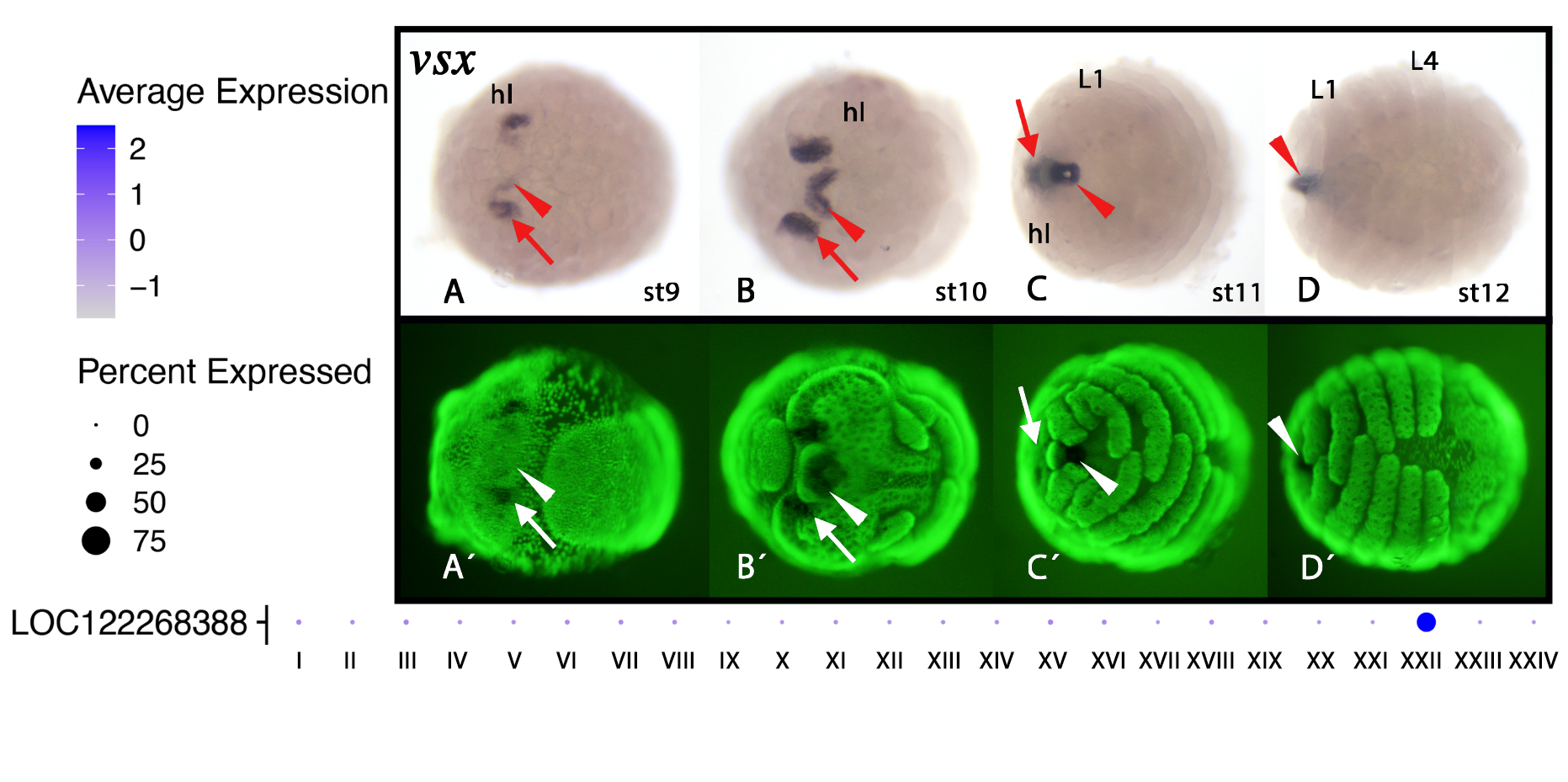

Supplement: Supplementary file 63 — Additional file 63. [file 12864_2023_9898_MOESM63_ESM.tif]

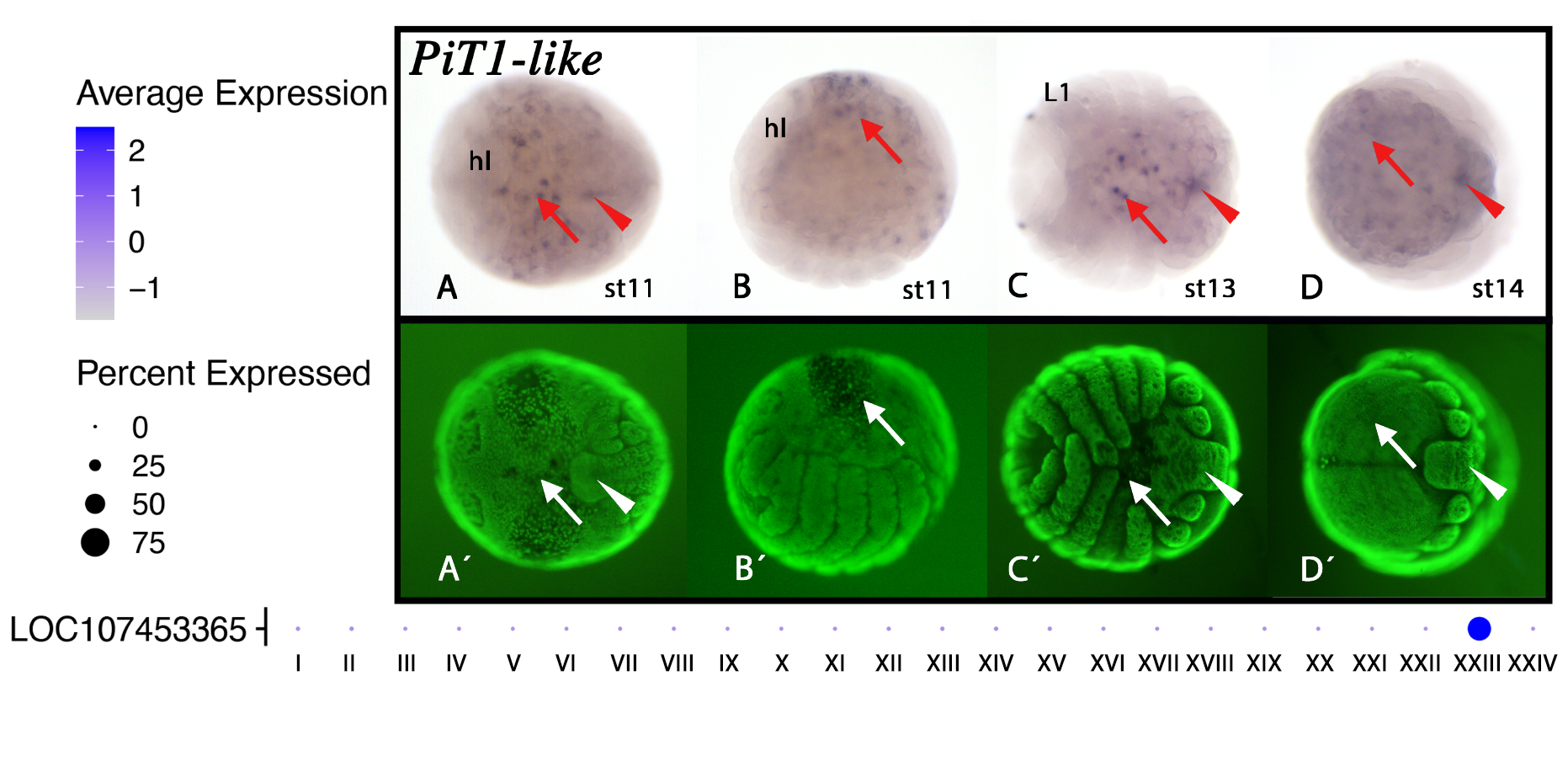

Supplement: Supplementary file 64 — Additional file 64. [file 12864_2023_9898_MOESM64_ESM.tif]

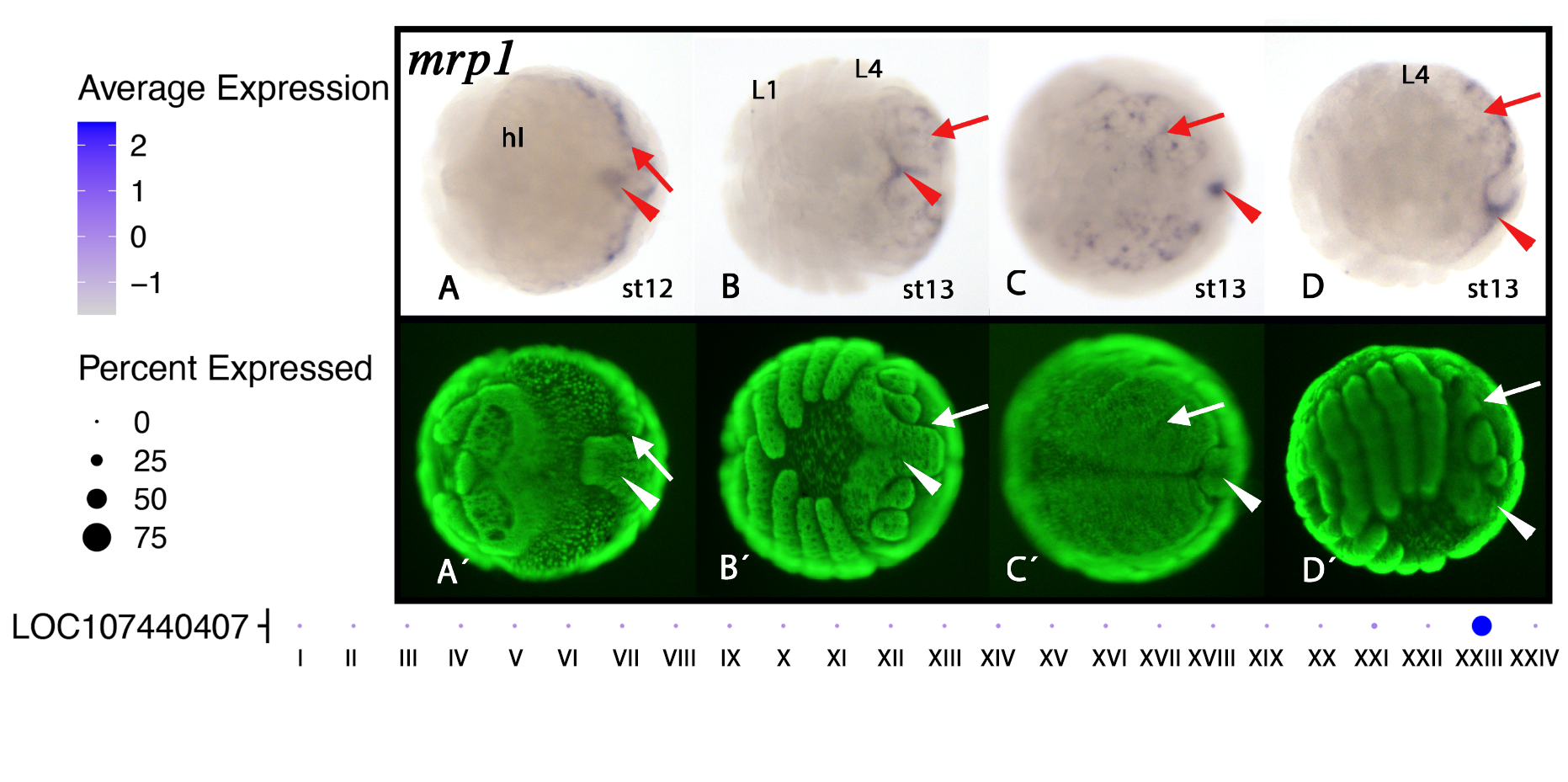

Supplement: Supplementary file 65 — Additional file 65. [file 12864_2023_9898_MOESM65_ESM.tif]

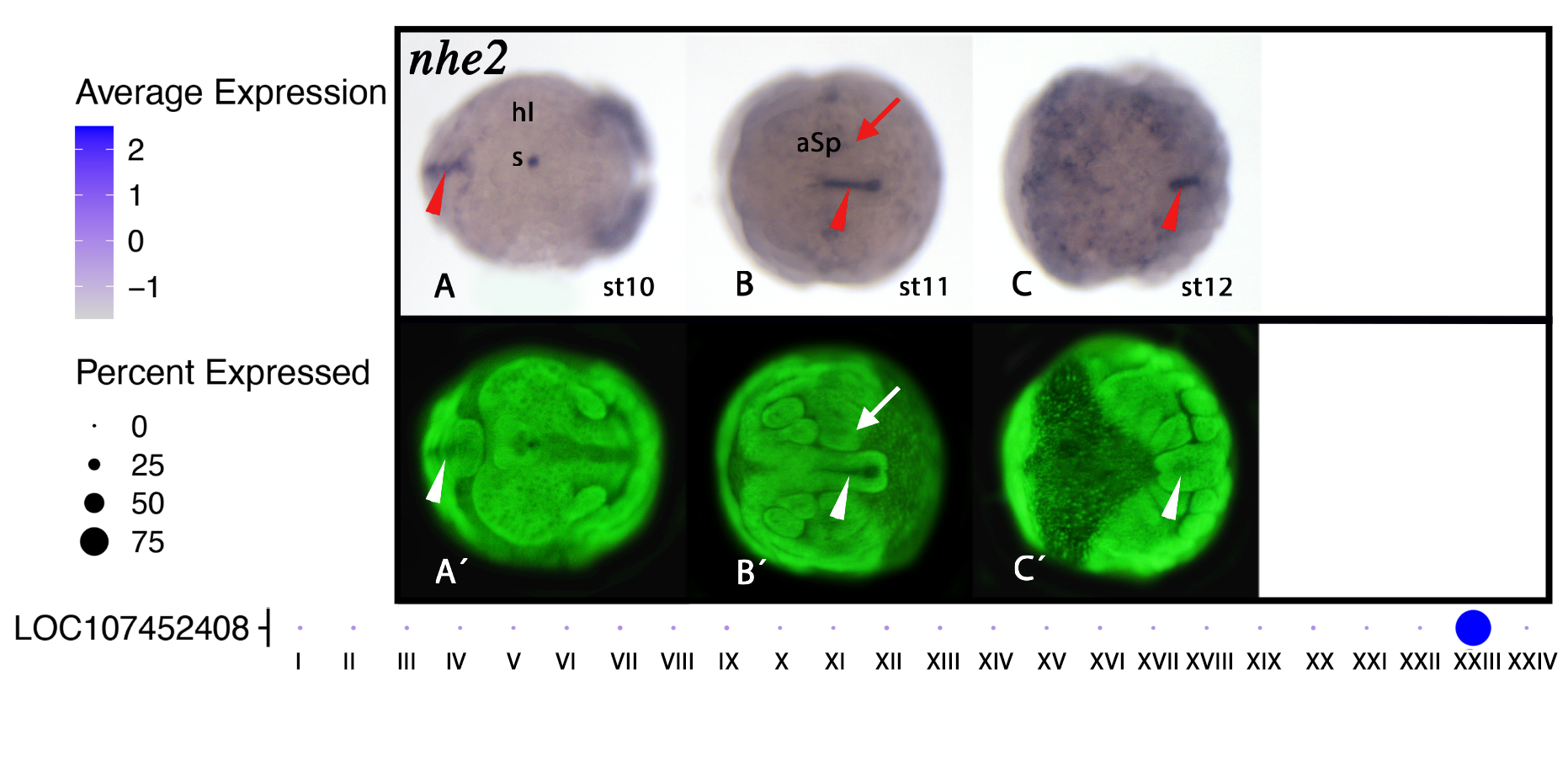

Supplement: Supplementary file 66 — Additional file 66. [file 12864_2023_9898_MOESM66_ESM.tif]

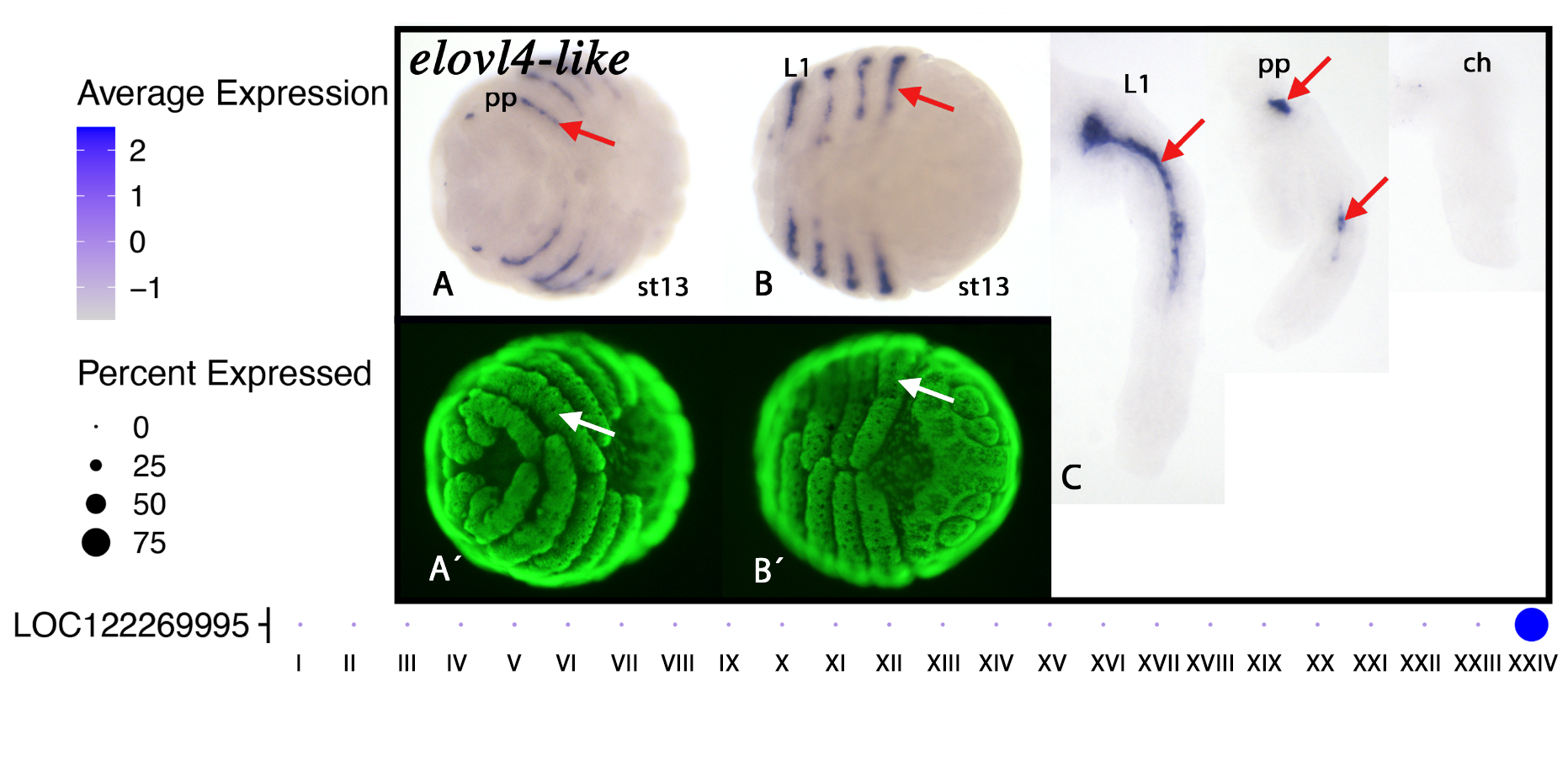

Supplement: Supplementary file 67 — Additional file 67. [file 12864_2023_9898_MOESM67_ESM.tif]

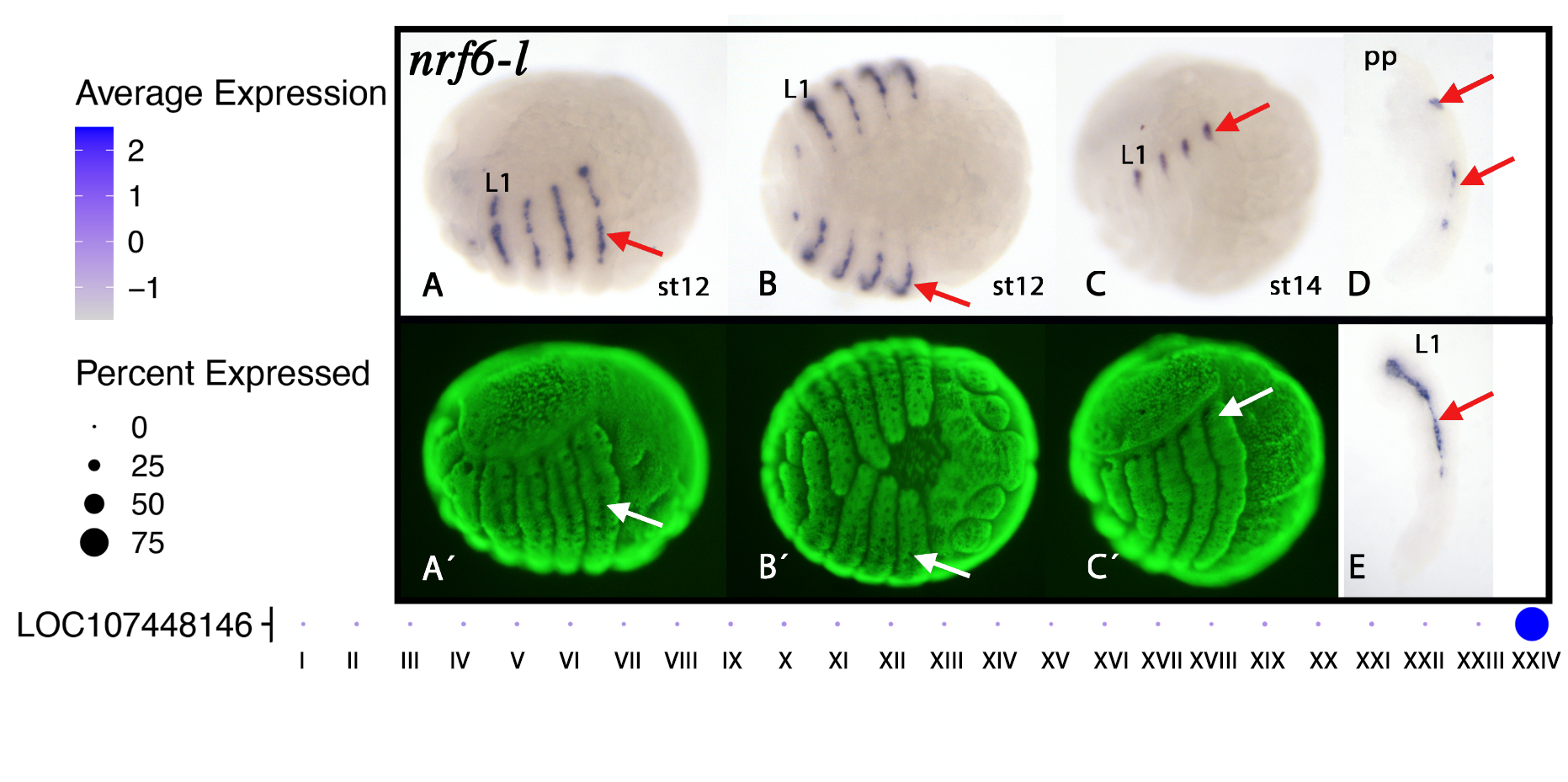

Supplement: Supplementary file 68 — Additional file 68. [file 12864_2023_9898_MOESM68_ESM.tif]

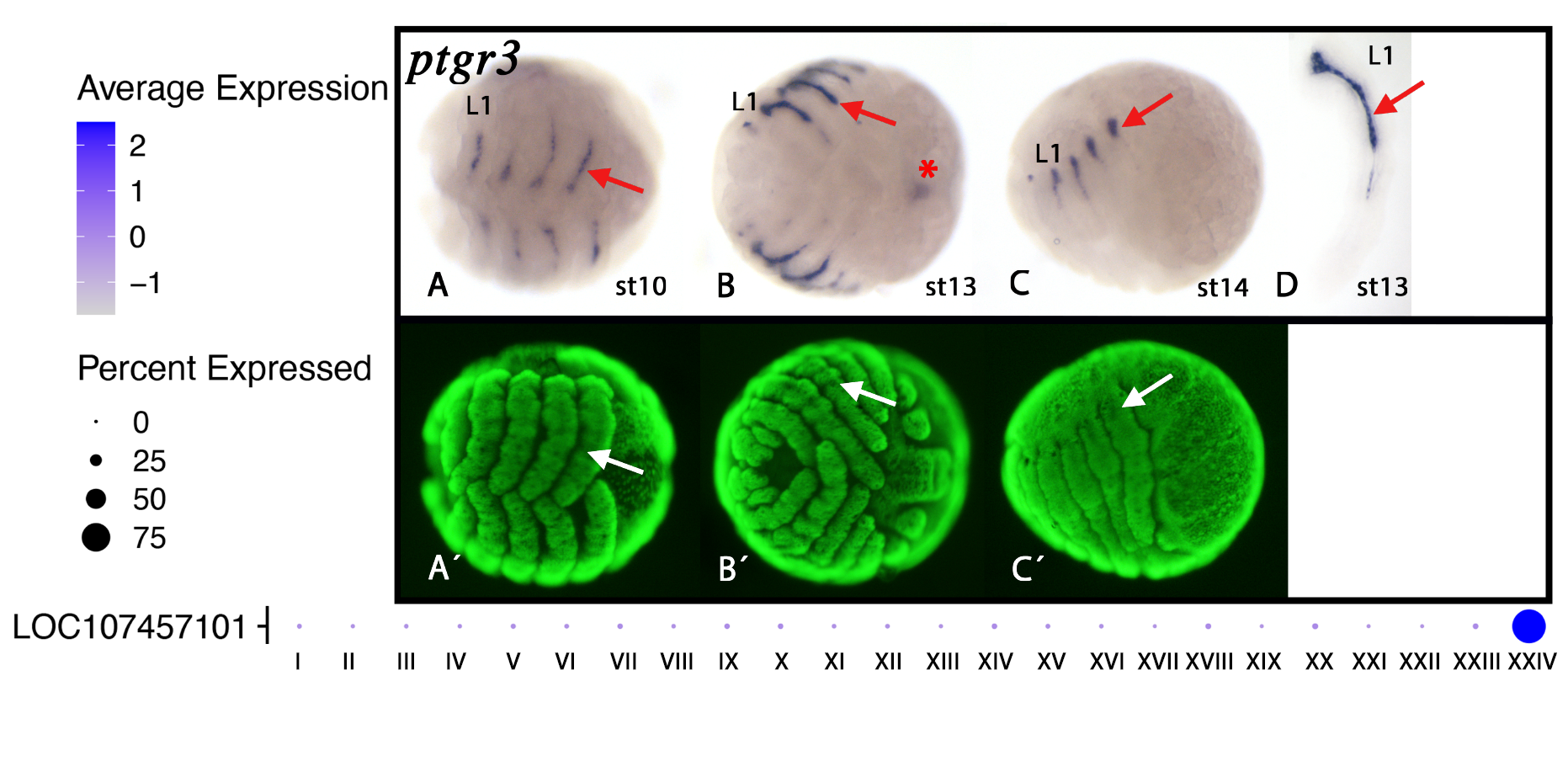

Supplement: Supplementary file 69 — Additional file 69. [file 12864_2023_9898_MOESM69_ESM.tif]
