# Supplementary material for: Single-cell RNA sequencing of mid-to-late stage spider embryos: new insights into spider development
Source: BMC Genomics. 2024 Feb 7;25:150. doi: 10.1186/s12864-023-09898-x (PMC10848406; doi:10.1186/s12864-023-09898-x)
Supplement: Supplementary file 70 — Additional file 70. [file 12864_2023_9898_MOESM70_ESM.docx]

**A simple dissociation protocol for single-cell RNA sequencing of spider embryos**

**Material**

*Reagents*:

90% methanol

Household chlorine bleach (e.g. Klorin™ Original)

Pronase from *Streptomyces griseus* (Roche #10165921001)

Sodium Thioglycolate (Sigma-Aldrich #TC632)

1x Calcium-and-Magnesium-free Phosphate Buffered Saline (D-PBS) (Corning #21-040-CV)

Fluorescein Diacetate (FDA) (Invitrogen® #F1303)

Propidium Iodide (PI) (Sigma-Aldrich #P4170)

*Technical equipment*:

Watchmaker glass or equivalent (e.g. Syracuse Watchmaker Glasses, 6.35 cm diameter)

Shaker (e.g. BioSan Thermo-Shaker TS-100C)

Centrifuge (e.g. Eppendorf™ Centrifuge 5427 R)

Tweezers (e.g. Sigma #T5415)

P1000 pipette and tips

P200 pipette and tips

P20 pipette and tips

P10 pipette and tips

Cell strainer, 40μm mesh size (Corning® cell strainer CLS431750)

20μm nylon mesh (e.g. pluriStrainer® Mini 20μm)

50ml collection tubes (e.g. Falcon 50ml Conical Centrifuge Tubes #10788561)

1.5ml low binding tubes (e.g. Thermo Scientific™ Low Protein Binding Collection Tubes #15342617)

Stereomicroscope (e.g. Leica M125 C)

Fluorescent microscope (e.g. Carl Zeiss Axio Imager.D2m)

Hemocytometer (e.g. SKC, Inc. C-Chip™ Disposable Hemocytometers #22600102)

**Protocol**

Perform all procedures at room temperature (20 – 22°C). Aliquots of the required reagents should be thawed in advance, and stored at room temperature. Prior to starting, check the quality and the stage of the embryos under a stereoscope. Apply a small amount of 90% methanol to a small number (ca. 10) of embryos collected in a watchmaker glass under a stereoscope. The methanol turns the chorion transparent. At least nine out of ten embryos should have developed properly and thus be of the same developmental stage.

1. *For stage 10.1 embryos*:

(Staging of embryos follows Mittmann and Wolff (2012))

*Embryo preparation:*

1. Take out ca. 50 embryos from the high-quality cocoon^1^ and collect them in a watchmaker glass.
2. Add 1ml of bleach to dissolve the outer membrane of the spider eggs (the chorion). This step ensures that no contaminating organisms such as microbes or mites end up in the spider-cell solution. Remove bleach by washing in water for several times (until the odor of bleach has disappeared). At this point, the embryos still have the inner egg-membrane (the vitelline membrane). Double-check that all embryos are of the desired developmental stages and have developed normally (remove embryos that do not fall into these categories). Even in good cocoons, around 10% of the embryos do not develop (are not fertilized), or develop irregularities (often delayed development).
3. Add 1ml 1X D-PBS and remove the vitelline membrane from the embryos using tweezers. This procedure should not take longer than 10 minutes to prevent increased cell-death. During this process, embryos will be damaged, fall apart, and the yolk mass will disintegrate (“embryo suspension”). This will not negatively influence the outcome of the procedure.

^1^Take a second or third cocoon of given female. Check the quality of her offspring by monitoring the development of her previously laid eggs. Only use high-quality eggs.

*Removing of yolk:*

1. Remove as much yolk as possible using tweezers and a 200P pipette.
2. Collect the embryonic tissue in a 1.5ml microcentrifuge tube and put it in a shaker at 1100 rpm for 5 minutes at 22°C – 24°C.
3. Discard 100 μl of the supernatant to reduce the number of small fat-droplets present by density gradient.
4. Add 1x D-PBS up to 1.5ml.
5. Centrifuge at 800 rpm for 4 minutes to decrease the size of any remaining fat-droplet to prevent them from clogging the device for cell capture (e.g. Chromium Controller™). The embryonic tissue will pellet in the bottom of the tube
6. Take out and discard supernatant by pipetting.
7. Suspend pellet in 1.5ml of 1x D-PBS by pipetting up and down.
8. Repeat steps g. and h. one more time.

*Dissociation:*

1. Resuspend pellet in 1ml of dissociation mix^2^ by pipetting up and down.
2. Transfer the suspension to a watchmaker glass.
3. Pipette the suspension up and down using a 1000 μl pipette for a few times. Swirl the watchmaker glass by hand intermittently in between the process of pipetting. The mild shearing forces will help the tissues to dissociate.
4. Check the progress of the dissociation under the microscope after the first 20 minutes.
5. If aggregates of tissue are still present, continue the dissociation for 20 to 30 additional minutes. When all cells are visibly dissociated under the microscope, proceed to step p.

^2^Dissociation mix 10.1: 250μl 2% Pronase + 250μl 1X D-PBS + 500μl 2% Sodium Thioglycolate

*Preparation of single cell suspension for cell capture^3^:*

1. Using a 1000ul pipette, apply and filter the suspension through a 40μm cell strainer attached to the top of a 50ml collection tube. By letting the suspension filter by gravity into the recipient tube without collecting it back with a pipette, we prevent aspirating clumps of tissue that have not dissociated properly.
2. Transfer the filtered suspension to a 1.5ml low binding tube, and add 1x D-PBS up to 1.5ml.
3. Centrifuge at 800 rpm for 4 minutes.
4. Discard supernatant and resuspend the pellet in 1.5ml of 1x D-PBS.
5. Centrifuge at 800 rpm for 4 minutes.
6. Discard supernatant and resuspend pellet in 100μl of 1x D-PBS.
7. Filter the resuspension carefully twice through a 20μm mesh using a 200 μl pipette and collect the suspension with a new pipette tip into a 1.5ml low binding tube.
8. Pipette the suspension gently up and down a couple of times using a 20μl pipette. This will homogenize the suspension for cell counting.
9. Assess cell viability via a live-dead assay with Fluorescein Diacetate (FDA) and Propidium Iodide (PI). Add 1μl of PI (1:50 dilution) and 1μl of FDA (1:10 dilution) to 8μl cell suspension, mix gently to avoid damaging the cells in the suspension, and check viability under the fluorescent microscope (10x and/or 20x augmentation): green-stained cells are alive, and red-stained cells are dead. In the meantime, keep the suspension at room temperature.
10. Pipette 10μl of cell suspension onto a wedge in the hemocytometer, and count the cells^4^ found in its large center square under the light microscope at 10x augmentation, including the cells that lie on the top and right boundaries. Use a clicker counter as aid. Multiply the number of cells by 10 to obtain the number of cells per μl.

**^3^**Cell capture is the process by which dissociated cells are captured into 10x Barcoded Gel Beads-in emulsion (GEMs) for single cell RNA sequencing, following the 10x Genomics Chromium Next GEM Single Cell 3’ v3.1 User Guide.

^4^When counting cells, keep in mind that the percentage of live cells should be higher than 80% in order to proceed to cell capture and sequencing. If it is less, it is better to restart the dissociation.

**Troubleshooting**

1. Before adding the dissociation mix, it is necessary to reduce the amount of yolk in the suspension as much as possible to prevent clogging the device for cell capture. To solve that we manually separated the yolk mass from the embryos with tweezers and pipetting and, then centrifuged the suspension to fraction the fat-droplets into smaller sizes.
2. In initial try-outs, we mixed 500 μl of 2% Pronase with 500 μl of Sodium Thioglycolate. However, this enzyme concentration causes the presence of more debris in the suspension. For that reason, we diminished it to 1%.
3. Tearing forces during tissue dissociation can rupture the nucleus and cause DNA leakage, which is visible as a whitish elastic string that can clog the pipette. We removed this string during the first filtering using a 40μm cell strainer.
4. After adding the dissociation mix to the suspension, if the dissociation is not successful after 40 minutes, terminate the experiment and try again. Extended dissociation time leads to increased cell damage (leaking of mRNAs) and cell death.
5. Ensure that the cell suspension is not too concentrated or too diluted before performing cell capture. Check the optimal range for cell capture recommended by the manufacturer’s user guide beforehand.

This dissociation protocol works for middle-late stage spider embryos. Given the similar embryonic morphology, this protocol might also work for other spider species. This protocol may work for other terrestrial arthropods as well, but thus far has been applied to embryos of *Parasteatoda tepidariorum* only.
