# Supplementary material for: Single-cell RNA sequencing of mid-to-late stage spider embryos: new insights into spider development
Source: BMC Genomics. 2024 Feb 7;25:150. doi: 10.1186/s12864-023-09898-x (PMC10848406; doi:10.1186/s12864-023-09898-x)
Supplement: Supplementary file 71 — Additional file 71. [file 12864_2023_9898_MOESM71_ESM.zip › FastQC report/SC062_S2_L001_I1_001_fastqc.html]

SC062\_S2\_L001\_I1\_001.fastq.gz FastQC Report 

FastQC Report

Mon 9 Aug 2021  
SC062\_S2\_L001\_I1\_001.fastq.gz

## Summary

- Basic Statistics
- Per base sequence quality
- Per tile sequence quality
- Per sequence quality scores
- Per base sequence content
- Per sequence GC content
- Per base N content
- Sequence Length Distribution
- Sequence Duplication Levels
- Overrepresented sequences
- Adapter Content

## Basic Statistics

| Measure | Value |
| --- | --- |
| Filename | SC062\_S2\_L001\_I1\_001.fastq.gz |
| File type | Conventional base calls |
| Encoding | Sanger / Illumina 1.9 |
| Total Sequences | 81885234 |
| Sequences flagged as poor quality | 0 |
| Sequence length | 8 |
| %GC | 50 |

## Per base sequence quality

## Per tile sequence quality

## Per sequence quality scores

## Per base sequence content

## Per sequence GC content

## Per base N content

## Sequence Length Distribution

## Sequence Duplication Levels

## Overrepresented sequences

| Sequence | Count | Percentage | Possible Source |
| --- | --- | --- | --- |
| GGCTGTTG | 21034208 | 25.68742491472883 | No Hit |
| CCGATAGC | 20985538 | 25.62798806925312 | No Hit |
| ATACCCAA | 19784478 | 24.161227896106396 | No Hit |
| TATGAGCT | 18976870 | 23.17495972472888 | No Hit |
| ATACCAAA | 135746 | 0.16577591999065425 | No Hit |
| GGCTGTTA | 88132 | 0.10762868431199696 | No Hit |

## Adapter Content

Can't analyse adapters as read length is too short (12 vs 0)

Produced by FastQC (version 0.11.9)
