# Supplementary material for: Single-cell RNA sequencing of mid-to-late stage spider embryos: new insights into spider development
Source: BMC Genomics. 2024 Feb 7;25:150. doi: 10.1186/s12864-023-09898-x (PMC10848406; doi:10.1186/s12864-023-09898-x)
Supplement: Supplementary file 71 — Additional file 71. [file 12864_2023_9898_MOESM71_ESM.zip › FastQC report/SC062_S2_L001_R2_001_fastqc.html]

SC062\_S2\_L001\_R2\_001.fastq.gz FastQC Report 

FastQC Report

Mon 9 Aug 2021  
SC062\_S2\_L001\_R2\_001.fastq.gz

## Summary

- Basic Statistics
- Per base sequence quality
- Per tile sequence quality
- Per sequence quality scores
- Per base sequence content
- Per sequence GC content
- Per base N content
- Sequence Length Distribution
- Sequence Duplication Levels
- Overrepresented sequences
- Adapter Content

## Basic Statistics

| Measure | Value |
| --- | --- |
| Filename | SC062\_S2\_L001\_R2\_001.fastq.gz |
| File type | Conventional base calls |
| Encoding | Sanger / Illumina 1.9 |
| Total Sequences | 81885234 |
| Sequences flagged as poor quality | 0 |
| Sequence length | 130 |
| %GC | 46 |

## Per base sequence quality

## Per tile sequence quality

## Per sequence quality scores

## Per base sequence content

## Per sequence GC content

## Per base N content

## Sequence Length Distribution

## Sequence Duplication Levels

## Overrepresented sequences

| Sequence | Count | Percentage | Possible Source |
| --- | --- | --- | --- |
| AAGCAGTGGTATCAACGCAGAGTACATGGGGGAGTATCGCGTCAGTCTGT | 508199 | 0.6206234936081394 | Clontech SMARTer II A Oligonucleotide (100% over 25bp) |
| AAGCAGTGGTATCAACGCAGAGTACATGGGGTCGACCTCAGATCAGACGA | 461727 | 0.5638708927668205 | Clontech SMARTer II A Oligonucleotide (100% over 25bp) |
| AAGCAGTGGTATCAACGCAGAGTACATGGGGGTTGACCGGCCCTGGAAGA | 284205 | 0.3470772276232367 | Clontech SMARTer II A Oligonucleotide (100% over 25bp) |
| GGGGGGGGGGGGGGGGGGGGGGGGGGGGGGGGGGGGGGGGGGGGGGGGGG | 220027 | 0.2687016806961802 | No Hit |
| AAGCAGTGGTATCAACGCAGAGTACATGGGCGCCGAAATTGTCCGATGAT | 212719 | 0.25977699471433396 | Clontech SMARTer II A Oligonucleotide (100% over 25bp) |
| GTCCGAAGCGGGTGTGGCACTGCACCGGGACTGGGCGAGACTGGCTGCAG | 210951 | 0.2576178752813969 | No Hit |
| AAGCAGTGGTATCAACGCAGAGTACATGGGAAAAGTTGTTGCGGTTAAAA | 198073 | 0.24189098610867985 | Clontech SMARTer II A Oligonucleotide (100% over 25bp) |
| GGCCCGTCGGGCTGGGGTCCGAAGCGGGTGTGGCACTGCACCGGGACTGG | 164781 | 0.20123408330249137 | No Hit |
| AAGCAGTGGTATCAACGCAGAGTACATGGGGTCCCGCTGCCGACCGAAAG | 158083 | 0.19305434237386437 | Clontech SMARTer II A Oligonucleotide (100% over 25bp) |
| AAGCAGTGGTATCAACGCAGAGTACATGGGATTGGAGGGAAAGTCTGGTG | 153042 | 0.18689816530291653 | Clontech SMARTer II A Oligonucleotide (100% over 25bp) |
| GCTCTGAGGACTGGGCCCGTCGGGCTGGGGTCCGAAGCGGGTGTGGCACT | 148514 | 0.18136847480951204 | No Hit |
| GGCGAGACTGGCTGCAGCGATGCAGTCCGGTCCGGCCCGGACCAGCGTCG | 146762 | 0.1792288949189545 | No Hit |
| GTTCGATCCGTAACTTCGGGATAAGGATTGGCTCTGAGGACTGGGCCCGT | 136960 | 0.1672584827687004 | No Hit |
| GCAGTGGTATCAACGCAGAGTACATGGGGGAGTATCGCGTCAGTCTGTAG | 132308 | 0.16157736082185464 | Clontech SMARTer II A Oligonucleotide (100% over 23bp) |
| GTGGTATCAACGCAGAGTACATGGGGGAGTATCGCGTCAGTCTGTAGAGG | 130164 | 0.15895906214299882 | No Hit |
| CTTCGGGATAAGGATTGGCTCTGAGGACTGGGCCCGTCGGGCTGGGGTCC | 129197 | 0.15777814105043653 | No Hit |
| GGATTGGCTCTGAGGACTGGGCCCGTCGGGCTGGGGTCCGAAGCGGGTGT | 126587 | 0.15459075319977714 | No Hit |
| CCGGGACTGGGCGAGACTGGCTGCAGCGATGCAGTCCGGTCCGGCCCGGA | 124531 | 0.1520799220040087 | No Hit |
| CGAAGCGGGTGTGGCACTGCACCGGGACTGGGCGAGACTGGCTGCAGCGA | 123039 | 0.15025785967711836 | No Hit |
| GAAGCGGGTGTGGCACTGCACCGGGACTGGGCGAGACTGGCTGCAGCGAT | 121439 | 0.14830390543916624 | No Hit |
| GGCTGGGGTCCGAAGCGGGTGTGGCACTGCACCGGGACTGGGCGAGACTG | 120762 | 0.14747713855223277 | No Hit |
| GTAACTTCGGGATAAGGATTGGCTCTGAGGACTGGGCCCGTCGGGCTGGG | 115433 | 0.1409692497184535 | No Hit |
| GTCCGGTCCGGCCCGGACCAGCGTCGGGGCCTTCCCGTGGAATGCCTCAG | 112865 | 0.13783315316654038 | No Hit |
| GGACTGGGCCCGTCGGGCTGGGGTCCGAAGCGGGTGTGGCACTGCACCGG | 111254 | 0.13586576549320234 | No Hit |
| GCCCGGACCAGCGTCGGGGCCTTCCCGTGGAATGCCTCAGCTGCGCGGCG | 111199 | 0.13579859831627275 | No Hit |
| GAACAATGTAGGTAAGGGAAGTCGGCAAGTTCGATCCGTAACTTCGGGAT | 111194 | 0.13579249220927914 | No Hit |
| AAGCAGTGGTATCAACGCAGAGTACATGGGAGGACCTCGGTTCTATTTTG | 108564 | 0.13258067993064537 | Clontech SMARTer II A Oligonucleotide (100% over 25bp) |
| GGGAAGTCGGCAAGTTCGATCCGTAACTTCGGGATAAGGATTGGCTCTGA | 106654 | 0.13024814705909005 | No Hit |
| GCAGTGGTATCAACGCAGAGTACATGGGGTCGACCTCAGATCAGACGAGA | 106515 | 0.13007839728466794 | Clontech SMARTer II A Oligonucleotide (100% over 23bp) |
| CTGAGGACTGGGCCCGTCGGGCTGGGGTCCGAAGCGGGTGTGGCACTGCA | 105986 | 0.12943237116474504 | No Hit |
| GTGGTATCAACGCAGAGTACATGGGGTCGACCTCAGATCAGACGAGACGA | 105954 | 0.129393292079986 | No Hit |
| ATTGGCTCTGAGGACTGGGCCCGTCGGGCTGGGGTCCGAAGCGGGTGTGG | 103544 | 0.12645014850907063 | No Hit |
| CTGGGCGAGACTGGCTGCAGCGATGCAGTCCGGTCCGGCCCGGACCAGCG | 103345 | 0.12620712545072535 | No Hit |
| CTGGGCCCGTCGGGCTGGGGTCCGAAGCGGGTGTGGCACTGCACCGGGAC | 102016 | 0.12458412221182638 | No Hit |
| GGGGCCTTCCCGTGGAATGCCTCAGCTGCGCGGCGGACCGTGCCTCGGTG | 101198 | 0.12358516310767337 | No Hit |
| GTCGGGCTGGGGTCCGAAGCGGGTGTGGCACTGCACCGGGACTGGGCGAG | 100093 | 0.12223571346208768 | No Hit |
| GCACTGCACCGGGACTGGGCGAGACTGGCTGCAGCGATGCAGTCCGGTCC | 97385 | 0.11892864591435373 | No Hit |
| GGCACTGCACCGGGACTGGGCGAGACTGGCTGCAGCGATGCAGTCCGGTC | 95168 | 0.11622119807339136 | No Hit |
| GGCAAGTTCGATCCGTAACTTCGGGATAAGGATTGGCTCTGAGGACTGGG | 94039 | 0.1148424391142364 | No Hit |
| GTGGCACTGCACCGGGACTGGGCGAGACTGGCTGCAGCGATGCAGTCCGG | 93612 | 0.11432097757698292 | No Hit |
| GTCGGCAAGTTCGATCCGTAACTTCGGGATAAGGATTGGCTCTGAGGACT | 93507 | 0.11419274933011732 | No Hit |
| AAGCAGTGGTATCAACGCAGAGTACATGGGCAGAAATCACATTGCGTCAG | 92810 | 0.11334155801520944 | Clontech SMARTer II A Oligonucleotide (100% over 25bp) |
| GGTCCGAAGCGGGTGTGGCACTGCACCGGGACTGGGCGAGACTGGCTGCA | 92584 | 0.11306556197909869 | No Hit |
| AGCGGGTGTGGCACTGCACCGGGACTGGGCGAGACTGGCTGCAGCGATGC | 91920 | 0.11225467097034858 | No Hit |
| GGGCGAGACTGGCTGCAGCGATGCAGTCCGGTCCGGCCCGGACCAGCGTC | 91549 | 0.11180159783142343 | No Hit |
| CGTCGGGCTGGGGTCCGAAGCGGGTGTGGCACTGCACCGGGACTGGGCGA | 91181 | 0.11135218835669443 | No Hit |
| CTCAGCTGCGCGGCGGACCGTGCCTCGGTGCGGACCGACCGTTTCGGCGG | 89843 | 0.109718194125207 | No Hit |
| GCAAGTTCGATCCGTAACTTCGGGATAAGGATTGGCTCTGAGGACTGGGC | 88319 | 0.10785705271355761 | No Hit |
| CGGCAAGTTCGATCCGTAACTTCGGGATAAGGATTGGCTCTGAGGACTGG | 86765 | 0.10595927465994663 | No Hit |
| GCGAGACTGGCTGCAGCGATGCAGTCCGGTCCGGCCCGGACCAGCGTCGG | 86101 | 0.1051483836511965 | No Hit |
| AAGCAGTGGTATCAACGCAGAGTACATGGGAAAAAAAAAAAAAAAAAAAA | 86095 | 0.1051410563228042 | Clontech SMARTer II A Oligonucleotide (100% over 25bp) |
| CCGTGGAATGCCTCAGCTGCGCGGCGGACCGTGCCTCGGTGCGGACCGAC | 85611 | 0.10454998516582366 | No Hit |
| CCGTAACTTCGGGATAAGGATTGGCTCTGAGGACTGGGCCCGTCGGGCTG | 85489 | 0.10440099615517982 | No Hit |
| GTGTGGCACTGCACCGGGACTGGGCGAGACTGGCTGCAGCGATGCAGTCC | 85195 | 0.10404195706395614 | No Hit |
| CCAGCGTCGGGGCCTTCCCGTGGAATGCCTCAGCTGCGCGGCGGACCGTG | 85074 | 0.103894189274711 | No Hit |

## Adapter Content

Produced by FastQC (version 0.11.9)
