# Supplementary material for: Single-cell RNA sequencing of mid-to-late stage spider embryos: new insights into spider development
Source: BMC Genomics. 2024 Feb 7;25:150. doi: 10.1186/s12864-023-09898-x (PMC10848406; doi:10.1186/s12864-023-09898-x)
Supplement: Supplementary file 71 — Additional file 71. [file 12864_2023_9898_MOESM71_ESM.zip › FastQC report/SC062_S2_L002_I1_001_fastqc.html]

SC062\_S2\_L002\_I1\_001.fastq.gz FastQC Report 

FastQC Report

Mon 9 Aug 2021  
SC062\_S2\_L002\_I1\_001.fastq.gz

## Summary

- Basic Statistics
- Per base sequence quality
- Per tile sequence quality
- Per sequence quality scores
- Per base sequence content
- Per sequence GC content
- Per base N content
- Sequence Length Distribution
- Sequence Duplication Levels
- Overrepresented sequences
- Adapter Content

## Basic Statistics

| Measure | Value |
| --- | --- |
| Filename | SC062\_S2\_L002\_I1\_001.fastq.gz |
| File type | Conventional base calls |
| Encoding | Sanger / Illumina 1.9 |
| Total Sequences | 82166680 |
| Sequences flagged as poor quality | 0 |
| Sequence length | 8 |
| %GC | 50 |

## Per base sequence quality

## Per tile sequence quality

## Per sequence quality scores

## Per base sequence content

## Per sequence GC content

## Per base N content

## Sequence Length Distribution

## Sequence Duplication Levels

## Overrepresented sequences

| Sequence | Count | Percentage | Possible Source |
| --- | --- | --- | --- |
| GGCTGTTG | 21108341 | 25.689660334335034 | No Hit |
| CCGATAGC | 21021573 | 25.58406035147094 | No Hit |
| ATACCCAA | 19805890 | 24.104527528677075 | No Hit |
| TATGAGCT | 19065191 | 23.20306844574954 | No Hit |
| ATACCAAA | 137271 | 0.1670640702532949 | No Hit |
| GGCTGTTA | 88436 | 0.10763000281866081 | No Hit |

## Adapter Content

Can't analyse adapters as read length is too short (12 vs 0)

Produced by FastQC (version 0.11.9)
