# Supplementary material for: Single-cell RNA sequencing of mid-to-late stage spider embryos: new insights into spider development
Source: BMC Genomics. 2024 Feb 7;25:150. doi: 10.1186/s12864-023-09898-x (PMC10848406; doi:10.1186/s12864-023-09898-x)
Supplement: Supplementary file 71 — Additional file 71. [file 12864_2023_9898_MOESM71_ESM.zip › FastQC report/SC062_S2_L002_R2_001_fastqc.html]

SC062\_S2\_L002\_R2\_001.fastq.gz FastQC Report 

FastQC Report

Mon 9 Aug 2021  
SC062\_S2\_L002\_R2\_001.fastq.gz

## Summary

- Basic Statistics
- Per base sequence quality
- Per tile sequence quality
- Per sequence quality scores
- Per base sequence content
- Per sequence GC content
- Per base N content
- Sequence Length Distribution
- Sequence Duplication Levels
- Overrepresented sequences
- Adapter Content

## Basic Statistics

| Measure | Value |
| --- | --- |
| Filename | SC062\_S2\_L002\_R2\_001.fastq.gz |
| File type | Conventional base calls |
| Encoding | Sanger / Illumina 1.9 |
| Total Sequences | 82166680 |
| Sequences flagged as poor quality | 0 |
| Sequence length | 130 |
| %GC | 46 |

## Per base sequence quality

## Per tile sequence quality

## Per sequence quality scores

## Per base sequence content

## Per sequence GC content

## Per base N content

## Sequence Length Distribution

## Sequence Duplication Levels

## Overrepresented sequences

| Sequence | Count | Percentage | Possible Source |
| --- | --- | --- | --- |
| AAGCAGTGGTATCAACGCAGAGTACATGGGGGAGTATCGCGTCAGTCTGT | 511199 | 0.6221487834241325 | Clontech SMARTer II A Oligonucleotide (100% over 25bp) |
| AAGCAGTGGTATCAACGCAGAGTACATGGGGTCGACCTCAGATCAGACGA | 461609 | 0.561795852041241 | Clontech SMARTer II A Oligonucleotide (100% over 25bp) |
| AAGCAGTGGTATCAACGCAGAGTACATGGGGGTTGACCGGCCCTGGAAGA | 286464 | 0.34863767162066184 | Clontech SMARTer II A Oligonucleotide (100% over 25bp) |
| GGGGGGGGGGGGGGGGGGGGGGGGGGGGGGGGGGGGGGGGGGGGGGGGGG | 213578 | 0.2599326150210767 | No Hit |
| AAGCAGTGGTATCAACGCAGAGTACATGGGCGCCGAAATTGTCCGATGAT | 212472 | 0.25858657061475526 | Clontech SMARTer II A Oligonucleotide (100% over 25bp) |
| GTCCGAAGCGGGTGTGGCACTGCACCGGGACTGGGCGAGACTGGCTGCAG | 211093 | 0.25690827474105077 | No Hit |
| AAGCAGTGGTATCAACGCAGAGTACATGGGAAAAGTTGTTGCGGTTAAAA | 198323 | 0.24136669511290954 | Clontech SMARTer II A Oligonucleotide (100% over 25bp) |
| GGCCCGTCGGGCTGGGGTCCGAAGCGGGTGTGGCACTGCACCGGGACTGG | 165755 | 0.2017301903886101 | No Hit |
| AAGCAGTGGTATCAACGCAGAGTACATGGGGTCCCGCTGCCGACCGAAAG | 156755 | 0.19077684531004538 | Clontech SMARTer II A Oligonucleotide (100% over 25bp) |
| AAGCAGTGGTATCAACGCAGAGTACATGGGATTGGAGGGAAAGTCTGGTG | 154435 | 0.18795331635645982 | Clontech SMARTer II A Oligonucleotide (100% over 25bp) |
| GCTCTGAGGACTGGGCCCGTCGGGCTGGGGTCCGAAGCGGGTGTGGCACT | 149707 | 0.18219915907518716 | No Hit |
| GGCGAGACTGGCTGCAGCGATGCAGTCCGGTCCGGCCCGGACCAGCGTCG | 147239 | 0.17919550844697632 | No Hit |
| GTTCGATCCGTAACTTCGGGATAAGGATTGGCTCTGAGGACTGGGCCCGT | 137113 | 0.16687177819524895 | No Hit |
| GCAGTGGTATCAACGCAGAGTACATGGGGGAGTATCGCGTCAGTCTGTAG | 133191 | 0.16209855381767888 | Clontech SMARTer II A Oligonucleotide (100% over 23bp) |
| GTGGTATCAACGCAGAGTACATGGGGGAGTATCGCGTCAGTCTGTAGAGG | 130716 | 0.1590863839210736 | No Hit |
| CTTCGGGATAAGGATTGGCTCTGAGGACTGGGCCCGTCGGGCTGGGGTCC | 129656 | 0.15779632327848708 | No Hit |
| GGATTGGCTCTGAGGACTGGGCCCGTCGGGCTGGGGTCCGAAGCGGGTGT | 125594 | 0.15285271353302823 | No Hit |
| CGAAGCGGGTGTGGCACTGCACCGGGACTGGGCGAGACTGGCTGCAGCGA | 123351 | 0.15012289653178149 | No Hit |
| GAAGCGGGTGTGGCACTGCACCGGGACTGGGCGAGACTGGCTGCAGCGAT | 122233 | 0.14876224766535534 | No Hit |
| CCGGGACTGGGCGAGACTGGCTGCAGCGATGCAGTCCGGTCCGGCCCGGA | 121745 | 0.14816833295442874 | No Hit |
| GGCTGGGGTCCGAAGCGGGTGTGGCACTGCACCGGGACTGGGCGAGACTG | 121217 | 0.1475257367098196 | No Hit |
| GTAACTTCGGGATAAGGATTGGCTCTGAGGACTGGGCCCGTCGGGCTGGG | 115358 | 0.140395109063674 | No Hit |
| GGACTGGGCCCGTCGGGCTGGGGTCCGAAGCGGGTGTGGCACTGCACCGG | 111579 | 0.13579592116901887 | No Hit |
| GTCCGGTCCGGCCCGGACCAGCGTCGGGGCCTTCCCGTGGAATGCCTCAG | 111095 | 0.1352068746114605 | No Hit |
| GAACAATGTAGGTAAGGGAAGTCGGCAAGTTCGATCCGTAACTTCGGGAT | 110992 | 0.13508151966222806 | No Hit |
| AAGCAGTGGTATCAACGCAGAGTACATGGGAGGACCTCGGTTCTATTTTG | 108444 | 0.13198050596665217 | Clontech SMARTer II A Oligonucleotide (100% over 25bp) |
| GGGAAGTCGGCAAGTTCGATCCGTAACTTCGGGATAAGGATTGGCTCTGA | 107448 | 0.13076833577795768 | No Hit |
| GCAGTGGTATCAACGCAGAGTACATGGGGTCGACCTCAGATCAGACGAGA | 106345 | 0.12942594248666248 | Clontech SMARTer II A Oligonucleotide (100% over 23bp) |
| CTGAGGACTGGGCCCGTCGGGCTGGGGTCCGAAGCGGGTGTGGCACTGCA | 105841 | 0.12881255516226287 | No Hit |
| GTGGTATCAACGCAGAGTACATGGGGTCGACCTCAGATCAGACGAGACGA | 105806 | 0.12876995882029066 | No Hit |
| GCCCGGACCAGCGTCGGGGCCTTCCCGTGGAATGCCTCAGCTGCGCGGCG | 104632 | 0.12734115580670902 | No Hit |
| ATTGGCTCTGAGGACTGGGCCCGTCGGGCTGGGGTCCGAAGCGGGTGTGG | 103892 | 0.12644054743358257 | No Hit |
| CTGGGCCCGTCGGGCTGGGGTCCGAAGCGGGTGTGGCACTGCACCGGGAC | 102024 | 0.12416711981060935 | No Hit |
| GGGGCCTTCCCGTGGAATGCCTCAGCTGCGCGGCGGACCGTGCCTCGGTG | 101592 | 0.12364135924683825 | No Hit |
| GTCGGGCTGGGGTCCGAAGCGGGTGTGGCACTGCACCGGGACTGGGCGAG | 101557 | 0.12359876290486606 | No Hit |
| CTGGGCGAGACTGGCTGCAGCGATGCAGTCCGGTCCGGCCCGGACCAGCG | 100034 | 0.1217452135099045 | No Hit |
| GCACTGCACCGGGACTGGGCGAGACTGGCTGCAGCGATGCAGTCCGGTCC | 97733 | 0.11894480828481813 | No Hit |
| GGCACTGCACCGGGACTGGGCGAGACTGGCTGCAGCGATGCAGTCCGGTC | 94875 | 0.11546651270320282 | No Hit |
| GGCAAGTTCGATCCGTAACTTCGGGATAAGGATTGGCTCTGAGGACTGGG | 94480 | 0.11498578255808801 | No Hit |
| AAGCAGTGGTATCAACGCAGAGTACATGGGCAGAAATCACATTGCGTCAG | 93777 | 0.1141302046036179 | Clontech SMARTer II A Oligonucleotide (100% over 25bp) |
| GTCGGCAAGTTCGATCCGTAACTTCGGGATAAGGATTGGCTCTGAGGACT | 93775 | 0.11412777052693379 | No Hit |
| GGTCCGAAGCGGGTGTGGCACTGCACCGGGACTGGGCGAGACTGGCTGCA | 93309 | 0.11356063065953255 | No Hit |
| GTGGCACTGCACCGGGACTGGGCGAGACTGGCTGCAGCGATGCAGTCCGG | 93178 | 0.11340119863672232 | No Hit |
| AGCGGGTGTGGCACTGCACCGGGACTGGGCGAGACTGGCTGCAGCGATGC | 92253 | 0.1122754381703143 | No Hit |
| CCAGCGTCGGGGCCTTCCCGTGGAATGCCTCAGCTGCGCGGCGGACCGTG | 92004 | 0.11197239562314068 | No Hit |
| CGTCGGGCTGGGGTCCGAAGCGGGTGTGGCACTGCACCGGGACTGGGCGA | 91362 | 0.11119105700753638 | No Hit |
| CTCAGCTGCGCGGCGGACCGTGCCTCGGTGCGGACCGACCGTTTCGGCGG | 90271 | 0.10986326817634592 | No Hit |
| GGGCGAGACTGGCTGCAGCGATGCAGTCCGGTCCGGCCCGGACCAGCGTC | 89245 | 0.10861458683738956 | No Hit |
| GCAAGTTCGATCCGTAACTTCGGGATAAGGATTGGCTCTGAGGACTGGGC | 87830 | 0.10689247758337078 | No Hit |
| CGGCAAGTTCGATCCGTAACTTCGGGATAAGGATTGGCTCTGAGGACTGG | 87256 | 0.10619389757502676 | No Hit |
| CCGTAACTTCGGGATAAGGATTGGCTCTGAGGACTGGGCCCGTCGGGCTG | 85589 | 0.10416509465880816 | No Hit |
| CCGTGGAATGCCTCAGCTGCGCGGCGGACCGTGCCTCGGTGCGGACCGAC | 85571 | 0.10414318796865105 | No Hit |
| GTGTGGCACTGCACCGGGACTGGGCGAGACTGGCTGCAGCGATGCAGTCC | 85486 | 0.10403973970957571 | No Hit |
| GCGAGACTGGCTGCAGCGATGCAGTCCGGTCCGGCCCGGACCAGCGTCGG | 85027 | 0.10348111911056891 | No Hit |
| AAGCAGTGGTATCAACGCAGAGTACATGGGAAAAAAAAAAAAAAAAAAAA | 85024 | 0.10347746799554272 | Clontech SMARTer II A Oligonucleotide (100% over 25bp) |

## Adapter Content

Produced by FastQC (version 0.11.9)
