# Supplementary material for: Single-cell RNA sequencing of mid-to-late stage spider embryos: new insights into spider development
Source: BMC Genomics. 2024 Feb 7;25:150. doi: 10.1186/s12864-023-09898-x (PMC10848406; doi:10.1186/s12864-023-09898-x)
Supplement: Supplementary file 71 — Additional file 71. [file 12864_2023_9898_MOESM71_ESM.zip › FastQC report/SC062_S2_L003_I1_001_fastqc.html]

SC062\_S2\_L003\_I1\_001.fastq.gz FastQC Report 

FastQC Report

Mon 9 Aug 2021  
SC062\_S2\_L003\_I1\_001.fastq.gz

## Summary

- Basic Statistics
- Per base sequence quality
- Per tile sequence quality
- Per sequence quality scores
- Per base sequence content
- Per sequence GC content
- Per base N content
- Sequence Length Distribution
- Sequence Duplication Levels
- Overrepresented sequences
- Adapter Content

## Basic Statistics

| Measure | Value |
| --- | --- |
| Filename | SC062\_S2\_L003\_I1\_001.fastq.gz |
| File type | Conventional base calls |
| Encoding | Sanger / Illumina 1.9 |
| Total Sequences | 83100049 |
| Sequences flagged as poor quality | 0 |
| Sequence length | 8 |
| %GC | 50 |

## Per base sequence quality

## Per tile sequence quality

## Per sequence quality scores

## Per base sequence content

## Per sequence GC content

## Per base N content

## Sequence Length Distribution

## Sequence Duplication Levels

## Overrepresented sequences

| Sequence | Count | Percentage | Possible Source |
| --- | --- | --- | --- |
| GGCTGTTG | 20968144 | 25.232408707725313 | No Hit |
| CCGATAGC | 20796339 | 25.02566394395267 | No Hit |
| ATACCCAA | 19534727 | 23.507479520258766 | No Hit |
| TATGAGCT | 18893699 | 22.73608647330641 | No Hit |
| ATACCAAA | 144777 | 0.17422011387742983 | No Hit |
| GGCGGTTG | 127741 | 0.15371952428090627 | No Hit |
| GGCTGGTG | 93840 | 0.11292412113980824 | No Hit |
| GGCTGTTA | 92818 | 0.1116942783030128 | No Hit |
| AGACCCAA | 90542 | 0.108955411085257 | No Hit |
| CCGGTAGC | 87861 | 0.10572917953393746 | No Hit |

## Adapter Content

Can't analyse adapters as read length is too short (12 vs 0)

Produced by FastQC (version 0.11.9)
