# Supplementary material for: Single-cell RNA sequencing of mid-to-late stage spider embryos: new insights into spider development
Source: BMC Genomics. 2024 Feb 7;25:150. doi: 10.1186/s12864-023-09898-x (PMC10848406; doi:10.1186/s12864-023-09898-x)
Supplement: Supplementary file 71 — Additional file 71. [file 12864_2023_9898_MOESM71_ESM.zip › FastQC report/SC062_S2_L003_R2_001_fastqc.html]

SC062\_S2\_L003\_R2\_001.fastq.gz FastQC Report 

FastQC Report

Mon 9 Aug 2021  
SC062\_S2\_L003\_R2\_001.fastq.gz

## Summary

- Basic Statistics
- Per base sequence quality
- Per tile sequence quality
- Per sequence quality scores
- Per base sequence content
- Per sequence GC content
- Per base N content
- Sequence Length Distribution
- Sequence Duplication Levels
- Overrepresented sequences
- Adapter Content

## Basic Statistics

| Measure | Value |
| --- | --- |
| Filename | SC062\_S2\_L003\_R2\_001.fastq.gz |
| File type | Conventional base calls |
| Encoding | Sanger / Illumina 1.9 |
| Total Sequences | 83100049 |
| Sequences flagged as poor quality | 0 |
| Sequence length | 130 |
| %GC | 46 |

## Per base sequence quality

## Per tile sequence quality

## Per sequence quality scores

## Per base sequence content

## Per sequence GC content

## Per base N content

## Sequence Length Distribution

## Sequence Duplication Levels

## Overrepresented sequences

| Sequence | Count | Percentage | Possible Source |
| --- | --- | --- | --- |
| AAGCAGTGGTATCAACGCAGAGTACATGGGGGAGTATCGCGTCAGTCTGT | 513815 | 0.6183089013581688 | Clontech SMARTer II A Oligonucleotide (100% over 25bp) |
| AAGCAGTGGTATCAACGCAGAGTACATGGGGTCGACCTCAGATCAGACGA | 467565 | 0.5626530978339134 | Clontech SMARTer II A Oligonucleotide (100% over 25bp) |
| AAGCAGTGGTATCAACGCAGAGTACATGGGGGTTGACCGGCCCTGGAAGA | 288253 | 0.3468746450438314 | Clontech SMARTer II A Oligonucleotide (100% over 25bp) |
| GGGGGGGGGGGGGGGGGGGGGGGGGGGGGGGGGGGGGGGGGGGGGGGGGG | 219586 | 0.26424292481464123 | No Hit |
| GTCCGAAGCGGGTGTGGCACTGCACCGGGACTGGGCGAGACTGGCTGCAG | 214448 | 0.25806001630636827 | No Hit |
| AAGCAGTGGTATCAACGCAGAGTACATGGGCGCCGAAATTGTCCGATGAT | 213187 | 0.2565425683443339 | Clontech SMARTer II A Oligonucleotide (100% over 25bp) |
| AAGCAGTGGTATCAACGCAGAGTACATGGGAAAAGTTGTTGCGGTTAAAA | 200180 | 0.24089035134022604 | Clontech SMARTer II A Oligonucleotide (100% over 25bp) |
| GGCCCGTCGGGCTGGGGTCCGAAGCGGGTGTGGCACTGCACCGGGACTGG | 167682 | 0.20178327452009087 | No Hit |
| AAGCAGTGGTATCAACGCAGAGTACATGGGGTCCCGCTGCCGACCGAAAG | 159884 | 0.19239940520372018 | Clontech SMARTer II A Oligonucleotide (100% over 25bp) |
| AAGCAGTGGTATCAACGCAGAGTACATGGGATTGGAGGGAAAGTCTGGTG | 154914 | 0.1864186626412218 | Clontech SMARTer II A Oligonucleotide (100% over 25bp) |
| GCTCTGAGGACTGGGCCCGTCGGGCTGGGGTCCGAAGCGGGTGTGGCACT | 151737 | 0.18259556020237727 | No Hit |
| GGCGAGACTGGCTGCAGCGATGCAGTCCGGTCCGGCCCGGACCAGCGTCG | 147502 | 0.17749929365264275 | No Hit |
| GTTCGATCCGTAACTTCGGGATAAGGATTGGCTCTGAGGACTGGGCCCGT | 138777 | 0.16699990152833724 | No Hit |
| GCAGTGGTATCAACGCAGAGTACATGGGGGAGTATCGCGTCAGTCTGTAG | 134647 | 0.16202998869471183 | Clontech SMARTer II A Oligonucleotide (100% over 23bp) |
| CTTCGGGATAAGGATTGGCTCTGAGGACTGGGCCCGTCGGGCTGGGGTCC | 131805 | 0.15861001477869163 | No Hit |
| GTGGTATCAACGCAGAGTACATGGGGGAGTATCGCGTCAGTCTGTAGAGG | 131243 | 0.15793372155532664 | No Hit |
| GGATTGGCTCTGAGGACTGGGCCCGTCGGGCTGGGGTCCGAAGCGGGTGT | 128189 | 0.1542586334696385 | No Hit |
| CGAAGCGGGTGTGGCACTGCACCGGGACTGGGCGAGACTGGCTGCAGCGA | 125914 | 0.15152096962060757 | No Hit |
| GAAGCGGGTGTGGCACTGCACCGGGACTGGGCGAGACTGGCTGCAGCGAT | 123635 | 0.14877849229667722 | No Hit |
| GGCTGGGGTCCGAAGCGGGTGTGGCACTGCACCGGGACTGGGCGAGACTG | 123024 | 0.14804323400579464 | No Hit |
| CCGGGACTGGGCGAGACTGGCTGCAGCGATGCAGTCCGGTCCGGCCCGGA | 122652 | 0.1475955808401509 | No Hit |
| GTAACTTCGGGATAAGGATTGGCTCTGAGGACTGGGCCCGTCGGGCTGGG | 117260 | 0.14110701667576633 | No Hit |
| GTCCGGTCCGGCCCGGACCAGCGTCGGGGCCTTCCCGTGGAATGCCTCAG | 116186 | 0.13981459866527876 | No Hit |
| GGACTGGGCCCGTCGGGCTGGGGTCCGAAGCGGGTGTGGCACTGCACCGG | 113498 | 0.13657994353288527 | No Hit |
| GAACAATGTAGGTAAGGGAAGTCGGCAAGTTCGATCCGTAACTTCGGGAT | 112476 | 0.13535010069608985 | No Hit |
| AAGCAGTGGTATCAACGCAGAGTACATGGGAGGACCTCGGTTCTATTTTG | 109318 | 0.13154986226301743 | Clontech SMARTer II A Oligonucleotide (100% over 25bp) |
| GGGAAGTCGGCAAGTTCGATCCGTAACTTCGGGATAAGGATTGGCTCTGA | 108685 | 0.13078812986018817 | No Hit |
| CTGAGGACTGGGCCCGTCGGGCTGGGGTCCGAAGCGGGTGTGGCACTGCA | 107997 | 0.1299602121774922 | No Hit |
| GCAGTGGTATCAACGCAGAGTACATGGGGTCGACCTCAGATCAGACGAGA | 107989 | 0.12995058522769343 | Clontech SMARTer II A Oligonucleotide (100% over 23bp) |
| GTGGTATCAACGCAGAGTACATGGGGTCGACCTCAGATCAGACGAGACGA | 107062 | 0.12883506241975862 | No Hit |
| ATTGGCTCTGAGGACTGGGCCCGTCGGGCTGGGGTCCGAAGCGGGTGTGG | 105624 | 0.12710461819342608 | No Hit |
| CTGGGCCCGTCGGGCTGGGGTCCGAAGCGGGTGTGGCACTGCACCGGGAC | 103179 | 0.12416238166117087 | No Hit |
| GCCCGGACCAGCGTCGGGGCCTTCCCGTGGAATGCCTCAGCTGCGCGGCG | 103153 | 0.1241310940743248 | No Hit |
| GGGGCCTTCCCGTGGAATGCCTCAGCTGCGCGGCGGACCGTGCCTCGGTG | 102621 | 0.12349090191270526 | No Hit |
| GTCGGGCTGGGGTCCGAAGCGGGTGTGGCACTGCACCGGGACTGGGCGAG | 102525 | 0.12337537851511977 | No Hit |
| CCAGCGTCGGGGCCTTCCCGTGGAATGCCTCAGCTGCGCGGCGGACCGTG | 101153 | 0.12172435662462726 | No Hit |
| CTGGGCGAGACTGGCTGCAGCGATGCAGTCCGGTCCGGCCCGGACCAGCG | 100387 | 0.12080257618139312 | No Hit |
| GCACTGCACCGGGACTGGGCGAGACTGGCTGCAGCGATGCAGTCCGGTCC | 99357 | 0.11956310639479888 | No Hit |
| GGCAAGTTCGATCCGTAACTTCGGGATAAGGATTGGCTCTGAGGACTGGG | 96532 | 0.11616358974710111 | No Hit |
| GGCACTGCACCGGGACTGGGCGAGACTGGCTGCAGCGATGCAGTCCGGTC | 96077 | 0.1156160569772949 | No Hit |
| GTGGCACTGCACCGGGACTGGGCGAGACTGGCTGCAGCGATGCAGTCCGG | 94857 | 0.11414794713297943 | No Hit |
| GGTCCGAAGCGGGTGTGGCACTGCACCGGGACTGGGCGAGACTGGCTGCA | 94466 | 0.11367742996156356 | No Hit |
| GTCGGCAAGTTCGATCCGTAACTTCGGGATAAGGATTGGCTCTGAGGACT | 94387 | 0.11358236383230051 | No Hit |
| AAGCAGTGGTATCAACGCAGAGTACATGGGCAGAAATCACATTGCGTCAG | 93882 | 0.11297466262625189 | Clontech SMARTer II A Oligonucleotide (100% over 25bp) |
| AGCGGGTGTGGCACTGCACCGGGACTGGGCGAGACTGGCTGCAGCGATGC | 93471 | 0.11248007808033902 | No Hit |
| CGTCGGGCTGGGGTCCGAAGCGGGTGTGGCACTGCACCGGGACTGGGCGA | 92527 | 0.1113440980040818 | No Hit |
| CTCAGCTGCGCGGCGGACCGTGCCTCGGTGCGGACCGACCGTTTCGGCGG | 90867 | 0.10934650592083284 | No Hit |
| GCAAGTTCGATCCGTAACTTCGGGATAAGGATTGGCTCTGAGGACTGGGC | 89546 | 0.10775685583530763 | No Hit |
| GGGCGAGACTGGCTGCAGCGATGCAGTCCGGTCCGGCCCGGACCAGCGTC | 88659 | 0.10668946777636677 | No Hit |
| CGGCAAGTTCGATCCGTAACTTCGGGATAAGGATTGGCTCTGAGGACTGG | 88225 | 0.1061672057497824 | No Hit |
| CCGTGGAATGCCTCAGCTGCGCGGCGGACCGTGCCTCGGTGCGGACCGAC | 87555 | 0.10536094870413375 | No Hit |
| CCGTAACTTCGGGATAAGGATTGGCTCTGAGGACTGGGCCCGTCGGGCTG | 87153 | 0.10487719447674453 | No Hit |
| GTGTGGCACTGCACCGGGACTGGGCGAGACTGGCTGCAGCGATGCAGTCC | 86526 | 0.10412268228626437 | No Hit |
| AAGCAGTGGTATCAACGCAGAGTACATGGGAAAAAAAAAAAAAAAAAAAA | 86491 | 0.10408056438089466 | Clontech SMARTer II A Oligonucleotide (100% over 25bp) |
| GCGAGACTGGCTGCAGCGATGCAGTCCGGTCCGGCCCGGACCAGCGTCGG | 86291 | 0.1038398906359249 | No Hit |
| GTCGGGGCCTTCCCGTGGAATGCCTCAGCTGCGCGGCGGACCGTGCCTCG | 83319 | 0.10026347878567436 | No Hit |

## Adapter Content

Produced by FastQC (version 0.11.9)
