# Supplementary material for: Single-cell RNA sequencing of mid-to-late stage spider embryos: new insights into spider development
Source: BMC Genomics. 2024 Feb 7;25:150. doi: 10.1186/s12864-023-09898-x (PMC10848406; doi:10.1186/s12864-023-09898-x)
Supplement: Supplementary file 71 — Additional file 71. [file 12864_2023_9898_MOESM71_ESM.zip › FastQC report/SC062_S2_L004_I1_001_fastqc.html]

SC062\_S2\_L004\_I1\_001.fastq.gz FastQC Report 

FastQC Report

Mon 9 Aug 2021  
SC062\_S2\_L004\_I1\_001.fastq.gz

## Summary

- Basic Statistics
- Per base sequence quality
- Per tile sequence quality
- Per sequence quality scores
- Per base sequence content
- Per sequence GC content
- Per base N content
- Sequence Length Distribution
- Sequence Duplication Levels
- Overrepresented sequences
- Adapter Content

## Basic Statistics

| Measure | Value |
| --- | --- |
| Filename | SC062\_S2\_L004\_I1\_001.fastq.gz |
| File type | Conventional base calls |
| Encoding | Sanger / Illumina 1.9 |
| Total Sequences | 82895647 |
| Sequences flagged as poor quality | 0 |
| Sequence length | 8 |
| %GC | 50 |

## Per base sequence quality

## Per tile sequence quality

## Per sequence quality scores

## Per base sequence content

## Per sequence GC content

## Per base N content

## Sequence Length Distribution

## Sequence Duplication Levels

## Overrepresented sequences

| Sequence | Count | Percentage | Possible Source |
| --- | --- | --- | --- |
| GGCTGTTG | 21290910 | 25.683990378891668 | No Hit |
| CCGATAGC | 21197020 | 25.570727495498048 | No Hit |
| ATACCCAA | 19988017 | 24.112263699443712 | No Hit |
| TATGAGCT | 19251035 | 23.223215810113647 | No Hit |
| ATACCAAA | 138718 | 0.16734051186065296 | No Hit |
| GGCTGTTA | 89195 | 0.10759913605596202 | No Hit |

## Adapter Content

Can't analyse adapters as read length is too short (12 vs 0)

Produced by FastQC (version 0.11.9)
