# Supplementary material for: Single-cell RNA sequencing of mid-to-late stage spider embryos: new insights into spider development
Source: BMC Genomics. 2024 Feb 7;25:150. doi: 10.1186/s12864-023-09898-x (PMC10848406; doi:10.1186/s12864-023-09898-x)
Supplement: Supplementary file 71 — Additional file 71. [file 12864_2023_9898_MOESM71_ESM.zip › FastQC report/SC062_S2_L004_R2_001_fastqc.html]

SC062\_S2\_L004\_R2\_001.fastq.gz FastQC Report 

FastQC Report

Mon 9 Aug 2021  
SC062\_S2\_L004\_R2\_001.fastq.gz

## Summary

- Basic Statistics
- Per base sequence quality
- Per tile sequence quality
- Per sequence quality scores
- Per base sequence content
- Per sequence GC content
- Per base N content
- Sequence Length Distribution
- Sequence Duplication Levels
- Overrepresented sequences
- Adapter Content

## Basic Statistics

| Measure | Value |
| --- | --- |
| Filename | SC062\_S2\_L004\_R2\_001.fastq.gz |
| File type | Conventional base calls |
| Encoding | Sanger / Illumina 1.9 |
| Total Sequences | 82895647 |
| Sequences flagged as poor quality | 0 |
| Sequence length | 130 |
| %GC | 46 |

## Per base sequence quality

## Per tile sequence quality

## Per sequence quality scores

## Per base sequence content

## Per sequence GC content

## Per base N content

## Sequence Length Distribution

## Sequence Duplication Levels

## Overrepresented sequences

| Sequence | Count | Percentage | Possible Source |
| --- | --- | --- | --- |
| AAGCAGTGGTATCAACGCAGAGTACATGGGGGAGTATCGCGTCAGTCTGT | 514687 | 0.6208854368432639 | Clontech SMARTer II A Oligonucleotide (100% over 25bp) |
| AAGCAGTGGTATCAACGCAGAGTACATGGGGTCGACCTCAGATCAGACGA | 467884 | 0.5644252948529372 | Clontech SMARTer II A Oligonucleotide (100% over 25bp) |
| AAGCAGTGGTATCAACGCAGAGTACATGGGGGTTGACCGGCCCTGGAAGA | 288578 | 0.3481220190005876 | Clontech SMARTer II A Oligonucleotide (100% over 25bp) |
| GGGGGGGGGGGGGGGGGGGGGGGGGGGGGGGGGGGGGGGGGGGGGGGGGG | 216169 | 0.26077243838871295 | No Hit |
| GTCCGAAGCGGGTGTGGCACTGCACCGGGACTGGGCGAGACTGGCTGCAG | 213696 | 0.2577891695567561 | No Hit |
| AAGCAGTGGTATCAACGCAGAGTACATGGGCGCCGAAATTGTCCGATGAT | 213628 | 0.257707138711397 | Clontech SMARTer II A Oligonucleotide (100% over 25bp) |
| AAGCAGTGGTATCAACGCAGAGTACATGGGAAAAGTTGTTGCGGTTAAAA | 200105 | 0.2413938575085855 | Clontech SMARTer II A Oligonucleotide (100% over 25bp) |
| GGCCCGTCGGGCTGGGGTCCGAAGCGGGTGTGGCACTGCACCGGGACTGG | 167692 | 0.20229288999939887 | No Hit |
| AAGCAGTGGTATCAACGCAGAGTACATGGGGTCCCGCTGCCGACCGAAAG | 159405 | 0.19229598388923846 | Clontech SMARTer II A Oligonucleotide (100% over 25bp) |
| AAGCAGTGGTATCAACGCAGAGTACATGGGATTGGAGGGAAAGTCTGGTG | 156410 | 0.18868300768555432 | Clontech SMARTer II A Oligonucleotide (100% over 25bp) |
| GCTCTGAGGACTGGGCCCGTCGGGCTGGGGTCCGAAGCGGGTGTGGCACT | 151733 | 0.1830409743951935 | No Hit |
| GTTCGATCCGTAACTTCGGGATAAGGATTGGCTCTGAGGACTGGGCCCGT | 137742 | 0.16616312796255756 | No Hit |
| GGCGAGACTGGCTGCAGCGATGCAGTCCGGTCCGGCCCGGACCAGCGTCG | 137086 | 0.16537177157203442 | No Hit |
| GCAGTGGTATCAACGCAGAGTACATGGGGGAGTATCGCGTCAGTCTGTAG | 135394 | 0.16333065112574607 | Clontech SMARTer II A Oligonucleotide (100% over 23bp) |
| GTGGTATCAACGCAGAGTACATGGGGGAGTATCGCGTCAGTCTGTAGAGG | 131072 | 0.15811686710159822 | No Hit |
| CTTCGGGATAAGGATTGGCTCTGAGGACTGGGCCCGTCGGGCTGGGGTCC | 130743 | 0.1577199825703755 | No Hit |
| GGATTGGCTCTGAGGACTGGGCCCGTCGGGCTGGGGTCCGAAGCGGGTGT | 127722 | 0.15407564163170112 | No Hit |
| CGAAGCGGGTGTGGCACTGCACCGGGACTGGGCGAGACTGGCTGCAGCGA | 124689 | 0.15041682466149278 | No Hit |
| GAAGCGGGTGTGGCACTGCACCGGGACTGGGCGAGACTGGCTGCAGCGAT | 123415 | 0.1488799526469707 | No Hit |
| GGCTGGGGTCCGAAGCGGGTGTGGCACTGCACCGGGACTGGGCGAGACTG | 122710 | 0.14802948579435057 | No Hit |
| CCGGGACTGGGCGAGACTGGCTGCAGCGATGCAGTCCGGTCCGGCCCGGA | 121501 | 0.14657102561730412 | No Hit |
| GTAACTTCGGGATAAGGATTGGCTCTGAGGACTGGGCCCGTCGGGCTGGG | 116345 | 0.14035115740154608 | No Hit |
| GAACAATGTAGGTAAGGGAAGTCGGCAAGTTCGATCCGTAACTTCGGGAT | 113001 | 0.13631716994741594 | No Hit |
| GGACTGGGCCCGTCGGGCTGGGGTCCGAAGCGGGTGTGGCACTGCACCGG | 112311 | 0.13548479813421324 | No Hit |
| AAGCAGTGGTATCAACGCAGAGTACATGGGAGGACCTCGGTTCTATTTTG | 109684 | 0.13231575356423722 | Clontech SMARTer II A Oligonucleotide (100% over 25bp) |
| GCAGTGGTATCAACGCAGAGTACATGGGGTCGACCTCAGATCAGACGAGA | 108456 | 0.13083437300392867 | Clontech SMARTer II A Oligonucleotide (100% over 23bp) |
| GGGAAGTCGGCAAGTTCGATCCGTAACTTCGGGATAAGGATTGGCTCTGA | 108323 | 0.13067393032109392 | No Hit |
| CTGAGGACTGGGCCCGTCGGGCTGGGGTCCGAAGCGGGTGTGGCACTGCA | 107376 | 0.12953153016587227 | No Hit |
| GTGGTATCAACGCAGAGTACATGGGGTCGACCTCAGATCAGACGAGACGA | 106962 | 0.12903210707795068 | No Hit |
| GTCCGGTCCGGCCCGGACCAGCGTCGGGGCCTTCCCGTGGAATGCCTCAG | 106473 | 0.1284422087929418 | No Hit |
| GCCCGGACCAGCGTCGGGGCCTTCCCGTGGAATGCCTCAGCTGCGCGGCG | 104961 | 0.12661822881966286 | No Hit |
| ATTGGCTCTGAGGACTGGGCCCGTCGGGCTGGGGTCCGAAGCGGGTGTGG | 104573 | 0.12615017046673152 | No Hit |
| CTGGGCCCGTCGGGCTGGGGTCCGAAGCGGGTGTGGCACTGCACCGGGAC | 102875 | 0.1241018120046762 | No Hit |
| GGGGCCTTCCCGTGGAATGCCTCAGCTGCGCGGCGGACCGTGCCTCGGTG | 102214 | 0.12330442393434723 | No Hit |
| GTCGGGCTGGGGTCCGAAGCGGGTGTGGCACTGCACCGGGACTGGGCGAG | 102014 | 0.12306315674211457 | No Hit |
| GCACTGCACCGGGACTGGGCGAGACTGGCTGCAGCGATGCAGTCCGGTCC | 98967 | 0.11938745106844996 | No Hit |
| CTGGGCGAGACTGGCTGCAGCGATGCAGTCCGGTCCGGCCCGGACCAGCG | 98474 | 0.11879272743959643 | No Hit |
| GGCACTGCACCGGGACTGGGCGAGACTGGCTGCAGCGATGCAGTCCGGTC | 96453 | 0.11635472246208538 | No Hit |
| GGCAAGTTCGATCCGTAACTTCGGGATAAGGATTGGCTCTGAGGACTGGG | 95412 | 0.11509892672651437 | No Hit |
| GTGGCACTGCACCGGGACTGGGCGAGACTGGCTGCAGCGATGCAGTCCGG | 94580 | 0.11409525520682648 | No Hit |
| GTCGGCAAGTTCGATCCGTAACTTCGGGATAAGGATTGGCTCTGAGGACT | 94508 | 0.11400839901762273 | No Hit |
| AAGCAGTGGTATCAACGCAGAGTACATGGGCAGAAATCACATTGCGTCAG | 93710 | 0.1130457429206144 | Clontech SMARTer II A Oligonucleotide (100% over 25bp) |
| GGTCCGAAGCGGGTGTGGCACTGCACCGGGACTGGGCGAGACTGGCTGCA | 93565 | 0.11287082420624571 | No Hit |
| CCAGCGTCGGGGCCTTCCCGTGGAATGCCTCAGCTGCGCGGCGGACCGTG | 93030 | 0.11222543446702334 | No Hit |
| AGCGGGTGTGGCACTGCACCGGGACTGGGCGAGACTGGCTGCAGCGATGC | 92697 | 0.11182372459195594 | No Hit |
| CGTCGGGCTGGGGTCCGAAGCGGGTGTGGCACTGCACCGGGACTGGGCGA | 92150 | 0.11116385882119961 | No Hit |
| CTCAGCTGCGCGGCGGACCGTGCCTCGGTGCGGACCGACCGTTTCGGCGG | 90341 | 0.10898159706745518 | No Hit |
| GCAAGTTCGATCCGTAACTTCGGGATAAGGATTGGCTCTGAGGACTGGGC | 89324 | 0.1077547533949521 | No Hit |
| CGGCAAGTTCGATCCGTAACTTCGGGATAAGGATTGGCTCTGAGGACTGG | 87703 | 0.10579928280190634 | No Hit |
| CCGTGGAATGCCTCAGCTGCGCGGCGGACCGTGCCTCGGTGCGGACCGAC | 86955 | 0.1048969435029562 | No Hit |
| GTGTGGCACTGCACCGGGACTGGGCGAGACTGGCTGCAGCGATGCAGTCC | 86560 | 0.10442044079829668 | No Hit |
| CCGTAACTTCGGGATAAGGATTGGCTCTGAGGACTGGGCCCGTCGGGCTG | 86480 | 0.10432393392140363 | No Hit |
| GGGCGAGACTGGCTGCAGCGATGCAGTCCGGTCCGGCCCGGACCAGCGTC | 86464 | 0.10430463254602501 | No Hit |
| AAGCAGTGGTATCAACGCAGAGTACATGGGAAAAAAAAAAAAAAAAAAAA | 86189 | 0.1039728901567051 | Clontech SMARTer II A Oligonucleotide (100% over 25bp) |
| GTCGGGGCCTTCCCGTGGAATGCCTCAGCTGCGCGGCGGACCGTGCCTCG | 82990 | 0.10011382141694365 | No Hit |

## Adapter Content

Produced by FastQC (version 0.11.9)
