# Supplementary material for: Single-cell RNA sequencing of mid-to-late stage spider embryos: new insights into spider development
Source: BMC Genomics. 2024 Feb 7;25:150. doi: 10.1186/s12864-023-09898-x (PMC10848406; doi:10.1186/s12864-023-09898-x)
Supplement: Supplementary file 75 — Additional file 75. [file 12864_2023_9898_MOESM75_ESM.docx]

**Supplementary File 6 – Description of gene expression**

*hex1* (Supplementary Figure 2)

*hex1* is expressed in dorsal tissue lining the germ band, including the head region, the leg-bearing part of the prosoma, and the opisthosoma. At earlier stages, *hex1* is expressed in the proximal part of the legs and the pedipalps, and the distal part of the chelicerae. Later during development, *hex1* is expressed in the form of rings along the proximal to distal axis (PD) of the legs and the pedipalps.

*elovl7* (Supplementary Figure 3)

*elovl7* is expressed in dorsal tissue lining the germ band, including the head region the leg-bearing part of the prosoma and the opisthosoma. In the legs and pedipalps, *elovl7* is first expressed in the distal half, but later there is expression all along the PD axis of these appendages in the form of rings.

*disco* (Supplementary Figure 4)

As soon as the appendages start to form, they express *disco*. Later, *disco* is expressed in the form of rings along the PD axis of the pedipalps the walking legs, and even the chelicerae. Additional expression is seen in the highly modified opisthosomal appendages, the spinnerets, the tracheae, and especially the developing book lungs. Weak expression is in the most posterior of the embryo.

*sax3* (Supplementary Figure 5)

*sax3* is exclusively expressed in the central nervous system (CNS), i.e. the brain and the ventral nerve cord.

*Ncad1* (Supplementary Figure 6)

*Ncad1* is expressed in the developing (CNS) and in a somewhat diffuse pattern in the appendages. At later developmental stages, expression in the appendages is in the form of separate spots, likely associated with the developing peripheral nervous system (PNS). A single spot of expression is located in the developing book lungs, but not the other opisthosomal appendages.

*pika* (Supplementary Figure 7)

*pika* is almost exclusively expressed in the CNS, but a few spots of expression in the developing appendages of both, the pro- and opisthosoma suggest an additional function in the development of the PNS.

*fstl5* (Supplementary Figure 8)

*fstl5* is expressed in the CNS and the PNS. Prominent expression is also seen in the developing spinnerets. At later developmental stages, there is also faint dorsal expression, possibly associated with the developing heart.

*cp47Ef-like* (Supplementary Figure 9)

Expression of this gene is in the form of rings in the developing appendages. There is also expression associated with the trachea, and faintly also the spinnerets.

*cht7* (Supplementary Figure 10)

*cht7* is exclusively expressed in the form of broad rings in the developing prosomal appendages, but is lacking from the opisthosomal appendages.

*np* (Supplementary Figure 11)

*np* is expressed in dorsal tissue lining the periphery of the complete germ band. Additionally, *np* is expressed in the form of rings along the PD axis of all appendages (including the rudimentary opisthosomal appendages).

*magu* (Supplementary Figure 12)

*magu* is expressed in the CNS and in single cells (or cell clusters) in the developing appendages, suggesting a function in PNS development. At later developmental stages, *magu* is also expressed the spinnerets, and in the form of a salt-and-pepper pattern in the developing heart.

*zfp395-like* (Supplementary Figure 13)

First expression of this gene is seen in the dorsal field (DF) (i.e. outside the embryo proper (= the germ band)). Later, this expression disappears and instead expression appears in the developing CNS.

*otop2* (Supplementary Figure 14)

*otop2* is first expressed in the DF, but this expression disappears later. Instead expression appears in the CNS and in the dorsal tissue of the opisthosoma. At later stages this expression is especially prominent in the developing heart (the dorsal tube).

*nog* (Supplementary Figure 15)

Very early during germ band formation (stage 6), *nog* is expressed in the dorsal margin of the developing embryo. This remains the only expression until stage 9 when additional expression appears in the CNS and the developing appendages.

*delta-2* (Supplementary Figure 16)

*delta-2* is exclusively expressed in the CNS and the PNS.

*PS2* (Supplementary Figure 17)

*PS2* is expressed in dorsal tissue around the head lobe, in the form of patches dorsal to the base of the pedipalps and legs, and in dorsal tissue of the opisthosoma. *PS2* is expressed also inside the developing appendages, likely representing mesodermal tissue.

*papilin* (Supplementary Figure 18)

First, *papilin* is exclusively expressed in the pedipalpal segment. Later, it is expressed in the dorsal region of the head lobe, dorsal tissue of the opisthosoma, and inside the developing appendages of both the pro- and opisthosoma. At least this latter expression is likely representing mesodermal tissue. Additional expression is in the form of segmental pages in ventral midline region. At later developmental stages, *papilin* is also expressed in the form of single dots (likely representing single cells) in the dorsal field and the developing heart (and associated structures).

*fibrinogen-like* (Supplementary Figure 19)

*fibrinogen-like* is first expressed in the dorsal field. Later, this expression disappears, and instead expression appears around the stomodaeum (mouth) and inside all developing appendages. ­This latter expression likely represents mesodermal tissue. At late developmental stages, fibrinogen-like is also expressed in the developing heart.

*cpo* (Supplementary Figure 20)

*cpo* is exclusively expressed in the developing CNS and PNS.

*delta* (Supplementary Figure 21)

First, *delta* is exclusively expressed in the CNS. Later, faint expression is also detectable in the developing heart.

*CD109 antigen* (Supplementary Figure 22)

This gene is exclusively expressed in the developing CNS.

*unc6289* (Supplementary Figure 23)

This previously uncharacterized gene is expressed in the CNS and the PNS. Dots of stronger expression in the head could be associated with the developing eyes.

*unc0558* (Supplementary Figure 24)

At around stage 11, *unc0558* is expressed in the tips of the developing appendages and in a few dot-like domains in the legs and the chelicerae. Later, strong expression appears in all opisthosomal appendages, except for the tracheae. At late developmental stages, this is almost the only remaining expression of *unc0558*. Additional expression, however, is in the suture of the dorsal tissue after (or at the very end of) dorsal closure in the form of the letter upsilon (Y).

*unc2247* (Supplementary Figure 25)

Expression of this gene is restricted to later developmental stages. At stage 11, strong expression appears in the spinnerets and the book lungs. Later, also the tips of the walking legs and the pedipalps express *unc2247*.

*unc1847* (Supplementary Figure 26)

This gene appears to be exclusively expressed in the developing CNS, although signal is detectable in the appendages. It is unclear if this latter signal is background or faint ubiquitous expression in these tissues.

*unc3246* (Supplementary Figure 27)

This gene is exclusively expressed in the developing CNS.

*dpr6* (Supplementary Figure 28)

*dpr6* is expressed in the developing CNS and the PNS. The latter expression is visible in the form of single cells or groups of cells in the dorsal of the opisthosoma, including their limb buds, and in the developing heart.

*Ncad2* (Supplementary Figure 29)

*Ncad2* is almost exclusively expressed in the CNS (brain and ventral nerve cord). Only a few single cells or groups of cells outside these structures express *Ncad2* implying a function in some cells/structures of the PNS.

*scrtl* (Supplementary Figure 30)

*scrtl* is exclusively expressed in the CNS.

*unc3142* (Supplementary Figure 31)

This previously undescribed gene is exclusively expressed in cells of the developing CNS and in the form of a single dot in the developing book lungs.

*elovl7.2* (Supplementary Figure 32)

First, at around stage 12, *elovl7.2* is expressed in the brain, the ventral nerve cord (together forming the CNS), and in the form of rings along the PD axis in the developing appendages.

*endoA* (Supplementary Figure 33)

*endoA* exclusively is expressed in the form of rings along the PD axis of all appendages.

*unc9645* (Supplementary Figure 34)

This previously uncharacterized gene is first expressed in the form of two patches dorsal to the base of the book lungs and the tracheae. Later, expression appears in the spinnerets and in the form of rings along the PD axis of the prosomal appendages. At stage 14, additional expression is dorsally in the opisthosoma.

*Tnc2* (Supplementary Figure 35)

*Tnc2* is first expressed in the distal region of the developing appendages. Later, this expression transforms into a distal spot and a median ring in the legs. Additional expression is around the stomodaeum and, at later stages, also in the very posterior of the embryo surrounding the posterior and lateral part of the segment addition zone (SAZ). The complete ventral sulcus (VS) also strongly expresses *Tnc2*.

*sideVIII* (Supplementary Figure 36)

Expression of *sideVIII* is limited to late developmental stages. At stage 12, expression appears along the split ventral midline and strongly in the form of transverse stripes inside the VS. Additional expression is inside and at the base of the developing pedipalps and legs, and in the form of a single spot in the anterior spinnerets.

*Tnc1* (Supplementary Figure 37)

First, *Tnc1* is expressed in all developing appendages, including the opisthosomal limb buds, the stomodaeum, and faintly at the posterior pole of the embryo. Later, expression in the legs is restricted to the tip and a median ring. In the pedipalps and the chelicerae, expression is strongest in the distal half. At stage 11, *Tnc1* is strongly expressed in the VS.

*AP2.2* (Supplementary Figure 38)

First, *AP2.2* is expressed in the form of a single ring in the developing pedipalps and legs. Later, expression in the legs and the pedipalps transforms into a series of rings along the PD axis. Additional expression is at the base of the chelicerae, and in the form of several patch-like domains in the head lobes.

*cfGbs* (Supplementary Figure 39)

This gene is first expressed in the form of one or two rings in the pedipalps and the legs. Later, additional ring-like domains form along the PD axis of these appendages. The labrum and the chelicerae also express *cfGbs*. At later developmental stages, expression appears also in the spinnerets.

*unc4* (Supplementary Figure 40)

*unc4* is expressed in the form of several patch-like domains in the developing opisthosomal appendages, in the form of two broad domains in the anterior of the head lobes, and in a patched segmental pattern in the ventral nerve cord.

*unc4096* (Supplementary Figure 41)

This gene is first expressed in the VS and the tips of the pedipalps, the legs, and the spinnerets. Additionally, there are two dots of expression in dorsal tissue lined with the second and third opisthosomal segments. Later, expression in the appendages transforms into a series of rings along the PD axis. At late developmental stages, expression in the spinnerets disappears.

*ss* (Supplementary Figure 42)

*ss* is expressed in the form of two dots anterior in the head lobes, the stomodaeum, and in the form of several dots in the developing opisthosomal appendages, suggesting a function in PNS development. Later, comparable expression appears is in the spinnerets.

*ss2* (Supplementary Figure 43)

*ss2* is expressed in the form of single dots in the prosomal appendages and in the spinnerets.

*sev* (Supplementary Figure 44)

*sev* is expressed in the form of multiple small dots in the brain and the ventral nerve cord (together forming the CNS), in the tail region, and in the form of dot-like domains in the appendages.

*VEGF receptor-like* (Supplementary Figure 45)

This gene is expressed exclusively in the form of several scattered small domain within the developing opisthosomal appendages and the spinnerets.

*insc* (Supplementary Figure 46)

At early developmental stages, *insc* is exclusively expressed in the developing CNS. Later, dots of expression appear in the appendages, suggesting a later function in PNS development.

*nerfin* (Supplementary Figure 47)

At early developmental stages, *nerfin* is exclusively expressed in the developing CNS. Later, dots appear in the appendages, suggesting a later function in PNS development.

*brat* (Supplementary Figure 48)

At very early stages, the germ disc, *brat* is faintly expressed in the cumulus. Later, expression is in the developing CNS and PNS.

*papilin-like* (Supplementary Figure 49)

This gene is expressed anterior in the head lobes in the interface between neurogenic and non-neurogenic tissue. Additional expression is dorsal to the base of the opisthosomal appendages, and in the form of a line between the ventral base of the opisthosomal appendages and the ventral nerve cord. Later, expression is also in the form of dots dorsal to the base of the prosomal appendages, and in the developing heart.

*PS1* (Supplementary Figure 50)

*PS1* is first expressed in the ventral midline. Later, a complex pattern of expression develops including expression around the stomodaeum, along the (then) split ventral midline, a series of rings in the prosomal appendages, and the ventral nerve cord. In the latter, strongest expression is in the interface between the ventral nerve cord and the base of the opisthosomal appendages. Additionally, *PS1* is strongly expressed in the spinnerets, and the tail region of the embryo.

*nord* (Supplementary Figure 51)

*nord* is first expressed inside the prosomal and opisthosomal appendages. Later, expression is also in the developing heart.

*unc1341* (Supplementary Figure 52)

This gene is expressed in the ventral nerve cord and in distinct domains in the head lobes. The latter are likely associated with the developing eyes.

*Nkx6.2* (Supplementary Figure 53)

*Nkx6.2* is exclusively expressed in the CNS.

*ryr* (Supplementary Figure 54)

*ryr* is expressed strongly in the developing heart and possibly also ubiquitously in all other tissues. It is, however, not possible to distinguish between faint ubiquitous expression and background.

*lethal 2* (Supplementary Figure 55)

First, this gene is exclusively expressed in the developing heart. Later faint expression appears in the stomodaeum and in a small domain proximally in the pedipalps (not to be mistaken with the artificial staining of the developing egg teeth).

*rapk2* (Supplementary Figure 56)

This gene is expressed in the developing heart, the stomodaeum, internally and proximally-restricted in the legs and pedipalps and, at later stages, also two broad domains in the developing brain.

*NaK-t-ATPase* (Supplementary Figure 57)

This gene first is expressed in the DF. Later, when the DF has been overgrown by the germ band proper, expression is in cells at the interface between the embryo proper and the yolk. Strong expression is also in the tail region that may connect the yolk with the embryo proper.

*unc8180* (Supplementary Figure 58)

*unc8180* is expressed in the DF and tissue/cells that form the interface between the yolk and the embryo proper.

*aqp7l* (Supplementary Figure 59)

*aqp7l* is expressed in the DF and tissue/cells that form the interface between the yolk and the embryo proper.

*unc5848* (Supplementary Figure 60)

This gene is exclusively expressed in the stomodaeum at late developmental stages.

*anapace* (Supplementary Figure 61)

*anapace* is first expressed along the ventral midline including the stomodaeum and the VS.

*unc3843* (Supplementary Figure 62)

This gene is expressed in the stomodaeum and, at later developmental stages in some cells of the CNS.

*vsx* (Supplementary Figure 63)

First, *vsx* is expressed in the form of two domains in the head lobes, and a faint domain surrounding the dorsal anlagen of the stomodaeum. Later, expression is dorsal to the stomodaeum in the ventral part of the labrum, and two separate domains in the head lobes. At stage 11 (and later stages), the two domains in the head lobes fuse ventrally and form one domain. The stomodaeal expression now surrounds the complete mouth opening. Very faint expression may be associated with the split ventral midline.

*PiT1-like* (Supplementary Figure 64)

This gene is expressed in several small domains (representing single cells, or small cell clusters) in the DF. Later, when the germ band overgrows the DF, expression is in these cells that are now located at the interface between the yolk and the embryo proper. Additional expression is internally in the tail region.

*mrp1* (Supplementary Figure 65)

Expression of *mrp1* is associated with cells underlying the opisthosoma and at the edge between opisthosomal tissue and the DF. Later, when the germ band overgrows the DF, expression is in these cells that are now located at the interface between the yolk and the embryo proper. Additional expression is internally in the tail region.

*nhe2* (Supplementary Figure 66)

*nhe2* is expressed in the form of a small dot-like domain ventral to the anlagen of the stomodaeum, internally in the tail region, and in cells of the DF and cells of the interface between the yolk and the embryo proper.

*elovl4* (Supplementary Figure 67)

This gene is exclusively expressed inside the developing legs and pedipalps.

*nrf6-l* (Supplementary Figure 68)

This gene is exclusively expressed inside the developing legs and pedipalps.

*ptgr3* (Supplementary Figure 69)

This gene is exclusively expressed inside the developing legs and pedipalps.

**Figure Legends of Supplementary Figures**

Supplementary Figure 1: Quality assay for cDNA

Electrophoresis using a 2% agarose E-Gel precast system. It shows a smeared column indicating the presence of amplified cDNA.

Supplementary Figure 2: Expression of *hex1*

In all panels, anterior is to the left, except for panel H (anterior view, dorsal up). Panels A and E represent dorsal views. Panels B, C, F and G represent ventral views. Panel D represents lateral view. Arrows mark expression in dorsal tissue and arrowheads mark expression in the form of rings in the developing appendages. Asterisks mark proximal expression of *hex1* in the appendages of earlier stage embryos. Abbreviations: aSp, anterior spinneret; bl, book lung; ch, chelicera; DF, dorsal field; hl, head lobe; L1, first leg; pp, pedipalp; pSp, posterior spinneret; s, stomodaeum; tr, trachea.

Supplementary Figure 3: Expression of *elovl7*

In all panels, anterior is to the left, except panel E (anterior view, dorsal up). Panel A represents dorsal view. Panels A and D represent anterior/dorsal views. Panels B and C represent ventral views. Arrows mark expression in dorsal tissue and arrowheads mark expression in the form of rings in the developing appendages. Asterisks mark distal expression of *elovl7* in the appendages of earlier stage embryos. Panels B´-D´ represent SYBR green staining of the embryos shown in panels B-D. Abbreviations as in Supplementary Figure 2.

Supplementary Figure 4: Expression of *disco*

In all panels, anterior is to the left, except panel F (anterior view, dorsal up). Panels A and C represent ventral views. Panels B and D represent lateral views. Arrowheads mark expression in the form of rings in the developing appendages. Asterisks mark expression at the posterior pole of the embryo. Panels C´ and D´ represent SYBR green staining of the embryos shown in panels C and D. Abbreviations as in Supplementary Figure 2.

Supplementary Figure 5: Expression of *sax3*

In all panels, anterior is to the left. All panels represent ventral views. Panel E represents a flat-mounted germ band. Arrows point to expression in the CNS. Abbreviations as in Supplementary Figure 2.

Supplementary Figure 6: Expression of *Ncad1*

In all panels, anterior is to the left. All panels represent ventral views, except panel E (lateral view on dissected appendages). Panel D represents a flat-mounted germ band. Arrows point to expression in the CNS. Asterisks mark expression in the book lungs. Abbreviations as in Supplementary Figure 2.

Supplementary Figure 7: Expression of *pika*

In all panels, anterior is to the left. All panels represent ventral views. Panel E represents a flat-mounted germ band. Arrows point to expression in the CNS. Abbreviations as in Supplementary Figure 2.

Supplementary Figure 8: Expression of *fstl5*

In all panels, anterior is to the left. Panels A-C represent ventral views. Panel D represents dorsal view, and panel E shows lateral view on dissected appendages. Arrows point to expression in the CNS. Asterisks mark expression associated with the developing heart. Panels B´-D´ represent SYBR green staining of the embryo shown in panels B-D. Abbreviations as in Supplementary Figure 2.

Supplementary Figure 9: Expression of *cp47Ef-like*

In all panels, anterior is to the left, except panel C (dorsal to the left) and panel E (anterior view, dorsal up). Panels A and D represent ventral views. Panel B represents ventral view, and panel C represents an anterior view. Arrowheads mark expression in the form of rings in the developing appendages. Asterisks mark expression in the trachea. Panels A´, B´ and D´ represent SYBR green staining of the embryos shown in panels A, B and D. Abbreviations as in Supplementary Figure 2.

Supplementary Figure 10: Expression of *cht7*

In all panels, anterior is to the left, except panel D (anterior view, dorsal up). Panels A and B represent ventral views. Panel B represents a lateral view. Arrowheads mark expression in the form of rings in the developing appendages. Abbreviations as in Supplementary Figure 2.

Supplementary Figure 11: Expression of *np*

In all panels, anterior is to the left, except panel G (dorsal up, anterior view). Panel A represents an anterior view. Panels B, C, E and F represent ventral views. Panel D represents lateral view. Arrowheads mark expression in the form of rings in the developing appendages. Arrows point to dorsal expression. Panel C´ represents SYBR green staining of the embryo shown in panel C. Abbreviations as in Supplementary Figure 2.

Supplementary Figure 12: Expression of *magu*

In all panels, anterior is to the left, ventral views (except panel E, dorsal view). Panel F represents a flat-mounted germ band. Arrows mark expression in the CNS. The asterisk in panel E marks expression in the heart. Panel A´ represents SYBR green staining of the embryo shown in panel A. Abbreviations as in Supplementary Figure 2.

Supplementary Figure 13: Expression of *zfp395*

­In all panels, anterior is to the left. All panels (except panel A, lateral view) represent ventral views. Arrows point to expression in the CNS. The asterisk in panel A marks expression in the dorsal field. Panel A´ represents SYBR green staining of the embryo shown in panel A. Abbreviations as in Supplementary Figure 2.

Supplementary Figure 14: Expression of *otop2*

In all panels, anterior is to the left. Panel A represents a lateral view. Panels B-E represent ventral views. Panel F represents a view on the posterior of the embryo, dorsal to the right. Panel G represents a dorsal view. Arrows point to expression in the CNS. The asterisks in panel A marks expression in the dorsal field. The asterisk in panels C, F and G mark expression in the heart. Arrowheads point to expression dorsal in the opisthosoma.­ Panel A´ represents SYBR green staining of the embryo shown in panel A. Abbreviations as in Supplementary Figure 2.

Supplementary Figure 15: Expression of *nog*

In all panels, anterior is to the left. Ventral views, except panel C (lateral view). Panels D and E represent flat-mounted germ bands. Arrowheads point to expression around the margin of the early germ band. Arrows point to expression in the CNS. Panel A´ represents SYBR green staining of the embryo shown in panel A. Abbreviations as in Supplementary Figure 2.

Supplementary Figure 16: Expression of *delta-2*

In all panels, anterior is to the left, except panel F (anterior up). All panels represent ventral views. Panel G represents a flat-mounted germ band, and panel F represents a flat-mounted head with a pair of chelicerae and pedipalps. Arrows point to expression in the CNS. Arrowheads point to expression in the PNS of the developing appendages. Abbreviations as in Supplementary Figure 2.

Supplementary Figure 17: Expression of *PS2*

In all panels, anterior is to the left, except panel F (lateral view on dissected appendages). Panels A and B represent ventral views. Panel C represents a lateral view. Panels D and E represent dorsal views. Arrows point to expression inside the appendages. Asterisks mark dorsal expression in the opisthosoma. Arrowheads point to expression surrounding the head lobe. Panel C´ represents a SYBR green staining of the embryo shown in panel C. Abbreviations as in Supplementary Figure 2.

Supplementary Figure 18: Expression of *papilin*

In all panels, anterior is to the left. Panels A and B represent lateral views. Panels C-E represent ventral views. Panels F and G represent dorsal views. The asterisk in panel A marks the pedipalpal segment. The asterisks in panels B and D mark expression dorsal in the opisthosoma. Asterisks in panels F and G mark the developing heart. Arrowheads in panels B and C point to expression surrounding the head lobes. Arrows point to expression inside the appendages. The open arrowhead in panel E points to patch-like expression in the ventral midline. Panel A´ represents a SYBR green staining of the embryo shown in panel A. Abbreviations as in Supplementary Figure 2.

Supplementary Figure 19: Expression of *fibrinogen-like*

In all panels, anterior is to the left (except panel E, lateral view on dissected appendages). Panel A represents a lateral view. Panels B-D, and F represent ventral views. Panel G represents a dorsal view. Arrows point to expression inside the appendages. The asterisk in panel G marks expression in the developing heart, and the arrowhead in the same panel points to expression surrounding the head lobe. Panel A´ represents a SYBR green staining of the embryo shown in panel A. Abbreviations as in Supplementary Figure 2.

Supplementary Figure 20: Expression of *cpo*

In all panels, anterior is to the left, ventral views. Panel D represents a flat-mounted germ band. Arrows point to expression in the CNS. Panel A´ represents a SYBR green staining of the embryo shown in panel A. Abbreviations as in Supplementary Figure 2.

Supplementary Figure 21: Expression of *delta*

In all panels, anterior is to the left, ventral views. Arrows point to expression in the CNS. Panel C´ represents a SYBR green staining of the embryo shown in panel C. Abbreviations as in Supplementary Figure 2.

Supplementary Figure 22: Expression of *CD109 antigen*

In all panels, anterior is to the left, except panel D (anterior up). All panels represent ventral views. Arrows point to expression in the CNS. Panels A´ - C´ represent SYBR green staining of the embryos shown in panels A-C. Abbreviations as in Supplementary Figure 2.

Supplementary Figure 23: Expression of *unc6289*

In all panels, anterior is to the left, ventral views. Panel E represents a flat-mounted embryo. Arrows point to expression in the CNS. The asterisk in panel A marks expression in the developing eyes. Panels A´-C´ represent SYBR green staining of the embryos shown in panels A-C. Abbreviations as in Supplementary Figure 2.

Supplementary Figure 24: Expression of *unc0558*

In all panels, anterior is to the left. Panels A and C represent ventral views. Panel B represents a lateral view. Panel D represents a dorsal view. Panel E shows dissected appendages (lateral view). Asterisks in panel A mark expression in the tips of the limbs. The arrow in panel D points to dorsal expression. Panels B´ and C´ represent SYBR green staining of the embryos shown in panels B and C. Abbreviations as in Supplementary Figure 2.

Supplementary Figure 25: Expression of *unc2247*

In all panels, anterior is to the left. Ventral views, except panel B (lateral view). Arrows point to expression in the book lungs, and arrowheads point to expression in the spinnerets. The asterisk in panel D mark expression in the legs. The inlay in panel B shows a dissected L1 (lateral view). Panels A´-D´ represent SYBR green staining of the embryos shown in panels A-D. Abbreviations as in Supplementary Figure 2.

Supplementary Figure 26: Expression of *unc1847*

In all panels, anterior is to the left, ventral views. Panel D represents a dissected germ band. Arrows point to expression in the CNS. Panels A´ - C´ represent SYBR green staining of the embryos shown in panels A - C. Abbreviations as in Supplementary Figure 2.

Supplementary Figure 27: Expression of *unc3246*

In all panels, anterior is to the left, ventral views. Panel E represents a dissected germ band. Arrows point to expression in the CNS. Panels A´ and B´ represent SYBR green staining of the embryos shown in panels A and B. Abbreviations as in Supplementary Figure 2.

Supplementary Figure 28: Expression of *dpr6*

In all panels, anterior is to the left, ventral views. Panel D represents a dissected germ band. Arrows point to expression in the CNS. Arrowheads point to expression in the PNS. The asterisk marks expression in the heart. Panels A´ - C´ represent SYBR green staining of the embryos shown in panels A - C. Abbreviations as in Supplementary Figure 2.

Supplementary Figure 29: Expression of *Ncad2*

In all panels, anterior is to the left, ventral views. Panel E represents a dissected germ band. Arrows point to expression in the CNS. Panels A´ - C´ represent SYBR green staining of the embryos shown in panels A - C. Abbreviations as in Supplementary Figure 2.

Supplementary Figure 30: Expression of *scrtl*

In all panels, anterior is to the left, ventral views. Panel F represents a dissected germ band. Arrows point to expression in the CNS. Abbreviations as in Supplementary Figure 2.

Supplementary Figure 31: Expression of *unc3142*

In all panels, anterior is to the left, ventral views. Panel E represents a dissected germ band. Arrows point to expression in the CNS. Abbreviations as in Supplementary Figure 2.

Supplementary Figure 32: Expression of *elovl7.2*

In all panels, anterior is to the left, ventral views; except panel D, anterior view, dorsal up. Panel D represents a dissected head and panel E represents a dissected germ band. Arrows point to expression in the CNS. Arrowheads point to expression in form of rings in the appendages. The asterisk marks expression in the spinnerets. Panels A´ - C´ represent SYBR green staining of the embryos shown in panels A - C. Abbreviations as in Supplementary Figure 2.

Supplementary Figure 33: Expression of *endoA*

In all panels, anterior is to the left, ventral views, except panel D, anterior view, dorsal up. Arrowheads point to expression in form of rings in the appendages. The asterisks mark expression in the spinnerets. Panels A´ - C´ represent SYBR green staining of the embryos shown in panels A - C. Abbreviations as in Supplementary Figure 2.

Supplementary Figure 34: Expression of *unc9645*

In all panels, anterior is to the left, ventral view, except panels D (lateral view) and E (anterior view on dissected head). Asterisks in panel A mark dorsal expression in the opisthosoma. Asterisk in panel D mark late dorsal expression. Arrowheads in panel E point to rings of expression in the appendages. Panels A´ - C´ represent SYBR green staining of the embryos shown in panels A-C. Abbreviations as in Supplementary Figure 2.

Supplementary Figure 35: Expression of *tnc2*

In all panels, anterior is to the left, except panel E (dissected appendages, lateral view). Panel A represents a lateral view, panels B-E represent ventral views. Asterisks marks the ventral sulcus. The arrowhead in panel D marks posterior expression surrounding the segment addition zone. Panels A´ and B´ represent SYBR green staining of the embryos shown in panels A and B. Abbreviations as in Supplementary Figure 2.

Supplementary Figure 36: Expression of *sideVIII*

In all panels, anterior is to the left. Panels A and C represent ventral views. Panels B and D represent lateral vies. Asterisks mark expression in the ventral sulcus. Panels A´-D´ represent SYBR green staining of the embryos shown in panels A-D. Abbreviations as in Supplementary Figure 2.

Supplementary Figure 37: Expression of *tnc1*

In all panels, anterior is to the left, except panel E (dissected appendages, lateral view). Panel A represents a lateral view. Panels B-D represent ventral views. The asterisk marks expression in the ventral sulcus. The arrowhead marks expression at the posterior pole of the embryo. Panels A´-C´ represent SYBR green staining of the embryos shown in panels A-C. Abbreviations as in Supplementary Figure 2.

Supplementary Figure 38: Expression of *AP2.2*

In all panels, anterior is to the left, except panel E (anterior view on dissected head, dorsal up). Asterisks in panel D mark expression in the brain/head lobes. Arrowheads in panel E mark expression in the form of rings along the proximal distal axis of the appendages. Panel A´ represents SYBR green staining of the embryo shown in panel A. Abbreviations as in Supplementary Figure 2.

Supplementary Figure 39: Expression of *cfGbs*

In all panels, anterior is to the left, except panel D (dissected head, anterior view, dorsal up). Arrowheads in panel D point to expression in the form of rings in the developing appendages. The asterisk in panel C marks expression in the spinnerets. Panels A´-C´ represent SYBR green staining of the embryos shown in panels A-C. Abbreviations as in Supplementary Figure 2.

Supplementary Figure 40: Expression of *unc-4 like*

In panels A-C, anterior is to the left. Panel D shows a dissected head, anterior view, dorsal up. Panel E represents dissected appendages, lateral view. The arrow in panel B points to expression in the CNS. The asterisk in panels C and D mark expression in the brain/head lobes. The arrowheads in panel E point to expression in the appendages. Panels A´ and B´ represents SYBR green staining of the embryos shown in panels A and B. Abbreviations as in Supplementary Figure 2.

Supplementary Figure 41: Expression of *unc4096*

In all panels, anterior is to the left, ventral views. Asterisks mark expression in the ventral sulcus. Arrows point to expression dorsal in the opisthosoma, and arrowheads point to expression in the form of rings in the appendages. Panels A´ and B´ represents SYBR green staining of the embryos shown in panels A and B. Abbreviations as in Supplementary Figure 2.

Supplementary Figure 42: Expression of *ss*

In all panels, anterior is to the left and ventral views, except panel F (dissected appendages, lateral view). Panel E represents a flat-mounted germ band. Asterisks mark expression in the head lobes, arrows mark expression in the form of single dots in the developing appendages. Abbreviations as in Supplementary Figure 2.

Supplementary Figure 43: Expression of *ss2*

In all panels, anterior is to the left, ventral views, except panel E (dissected appendages, lateral view). Arrows point to expression in the appendages. Panel A´ represents SYBR green staining of the embryo shown in A. Abbreviations as in Supplementary Figure 2.

Supplementary Figure 44: Expression of *sev*

In all panels, anterior is to the left and ventral views, except panel F (dissected appendages, lateral view). Asterisks mark expression in the tail region. Arrows point to dot-like expression in the appendages. Abbreviations as in Supplementary Figure 2.

Supplementary Figure 45: Expression of *VEGF receptor-like*

In all panels, anterior is to the left, except panel D (dissected appendages, lateral view). Arrows point to expression in the prosomal appendages, and arrowheads point to expression in the spinnerets. Panels A´-C´ represent SYBR green staining of the embryos shown in panels A-C. Abbreviations as in Supplementary Figure 2.

Supplementary Figure 46: Expression of *insc*

In all panels, anterior is to the left, ventral views. Arrows point to expression in the CNS. The asterisk marks dot-like expression in the appendages of late-stage embryos. Panel F represents a flat-mounted germ band. Panel D´ represents SYBR green staining of the embryo shown in panel D. Abbreviations as in Supplementary Figure 2.

Supplementary Figure 47: Expression of *nerfin*

In all panels, anterior is to the left, ventral views, except panel e (dissected L1, lateral view). Arrows point to expression in the CNS. The asterisks in panels E and e mark dot-like expression in the appendages. Abbreviations as in Supplementary Figure 2.

Supplementary Figure 48: Expression of *brat*

In all panels, anterior is to the left, ventral views, except panel G (dissected appendages, lateral views). The asterisk in panel A marks expression in the cumulus. Arrows point to expression in the CNS. Abbreviations as in Supplementary Figure 2.

Supplementary Figure 49: Expression of *papilin-like*

In all panels, anterior is to the left. Panel A represents view onto anterior of embryo. Panels B and D represent dorsal views. Panel C represents a ventral view. Arrowheads point to expression at the interface between the opisthosomal appendages and the dorsal tissue. Arrows point to expression at the interface between the CNS and the opisthosomal appendages. The asterisk in panel D marks expression in the developing heart. Panels A´-D´ represents SYBR green staining of the embryos shown in panels A-D. Abbreviations as in Supplementary Figure 2.

Supplementary Figure 50: Expression of *PS1*

In all panels, anterior is to the left, ventral views, except panel A (lateral view) Arrows point to expression in the ventral midline. The arrowhead points to expression in the form of rings in the appendages. The closed circle in panel D marks dorsal expression, and the triangular symbol marks expression in the tail region. Asterisks in panels E and F mark expression in the CNS. Panels A´ and B´ represent SYBR green staining of the embryos shown in panels A and D. Abbreviations as in Supplementary Figure 2.

Supplementary Figure 51: Expression of *nord*

In all panels, anterior is to the left, ventral views (except panel F, dorsal view). Arrows point to expression inside the prosomal appendages. Arrowheads point to expression inside the spinnerets. The asterisk in panel F marks expression in the heart. Abbreviations as in Supplementary Figure 2.

Supplementary Figure 52: Expression of *unc1341*

In all panels, anterior is to the left. Panels A and D represent ventral views. Panels B and C represent dorsal views. Arrows point to expression in the CNS. The arrowhead in panel C point to expression in a lateral eye. The asterisk marks expression in the brain/head lobe. Panels A´-D´ represent SYBR green staining of the embryos shown in A-D. Abbreviations as in Supplementary Figure 2.

Supplementary Figure 53: Expression of *Nkx6.2*

In all panels, anterior is to the left, ventral views (except panel A, lateral view). Arrows point to expression in the CNS. Panels A´ and B´ represent SYBR green staining of the embryos shown in panels A and B. Panel E represents a flat-mounted germ band. Abbreviations as in Supplementary Figure 2.

Supplementary Figure 54: Expression of *ryr*

In all panels, anterior is to the left, dorsal views. Arrows point to expression in the developing heart. Panels A´-C´ represent SYBR green staining of the embryos shown in panels A-C. Abbreviations as in Supplementary Figure 2.

Supplementary Figure 55: Expression of *lethal 2*

In all panels, anterior is to the left, dorsal views (except panel D, view on anterior of embryo). Arrows point to expression in the heart. The arrowhead in panel D points to expression at the base of the pedipalp (not to be mistaken with the artificial staining of the egg teeth at the base of the pedipalps. Panels A´-D´ represent SYBR green staining of the embryos shown in panels A-D. Abbreviations as in Supplementary Figure 2.

Supplementary Figure 56: Expression of *rapk2*

In all panels, anterior is to the left. Panel A represents a lateral view. Panels B, D and E represent dorsal views. Panel C represents a lateral view. Arrows point to expression in the developing heart. The arrowhead in panel E points to expression in the brain. Panels A´-C´ represent SYBR green staining of the embryos shown in panels A-C. Abbreviations as in Supplementary Figure 2.

Supplementary Figure 57: Expression of *NaK-t-ATPase*

In all panels, anterior is to the left. Panel A represents an anterior view. Panel B represents a lateral view. Panel C represents a dorsal view. Panel D represents a ventral view. Arrows point to expression in the dorsal field. Arrowheads in panel D point to expression in the tail region. Panels A´-D´ represents SYBR green staining of the embryos shown in panels A-D. Abbreviations as in Supplementary Figure 2.

Supplementary Figure 58: Expression of *unc8180*

In all panels, anterior is to the left. Panels A, B and D represent lateral views. Panel C represents a dorsal view. Arrows point to expression in the DF and on the interface between the embryo proper and the yolk. A´-D´ represents SYBR green staining of the embryos shown in panels A-D. Abbreviations as in Supplementary Figure 2.

Supplementary Figure 59: Expression of *aqp7l*

In all panels, anterior is to the left. Panels A, B and D represent ventral views. Panels D and E represent lateral views, and panel F represents a dorsal view. Arrows point to expression in tissue surrounding the yolk at the interface between embryo proper and yolk. A´ and B´ represents SYBR green staining of the embryos shown in panels A and B. Abbreviations as in Supplementary Figure 2.

Supplementary Figure 60: Expression of *unc5848*

In all panels, anterior is to the left, ventral views. Arrows point to expression in the stomodaeum. A´ and B´ represents SYBR green staining of the embryos shown in panels A and B. Abbreviations as in Supplementary Figure 2.

Supplementary Figure 61: Expression of *anapece*

In all panels, anterior is to the left. Panel A represents a lateral view. Panels B and C represent ventral views, and panel D represents a view on the anterior of the embryo. Arrows point to expression in the ventral midline and the ventral sulcus. Arrowheads point to expression in the stomodaeum. A´-D´ represents SYBR green staining of the embryos shown in panels A-D. Abbreviations as in Supplementary Figure 2.

Supplementary Figure 62: Expression of *unc3843*

In all panels, anterior is to the left. Panels A and B represent anterior views, panels C and D represent ventral views. Arrowheads point to expression in the stomodaeum and the arrow in panel D points to expression in the CNS. A´-D´ represents SYBR green staining of the embryos shown in panels A-D. Abbreviations as in Supplementary Figure 2.

Supplementary Figure 63: Expression of *vsx*

­In all panels, anterior is to the left. Panel A represents a dorsal view. Panels B-D represent ventral views. Arrows point to expression in the brain/head lobes. Arrowheads point to expression dorsal to the stomodaeum and, at later stages, in the stomodaeum. A´-D´ represents SYBR green staining of the embryos shown in panels A-D. Abbreviations as in Supplementary Figure 2.

Supplementary Figure 64: Expression of *PiT1-like*

In all panels, anterior is to the left. Panel A represents a dorsal view, panel B a lateral view, panel C a ventral view, and panel D a view on the posterior of the embryo. In all panels, arrows point to dot-like expression at the interface between the embryo proper and the yolk. The arrowheads point to expression in the tail region. A´-D´ represents SYBR green staining of the embryos shown in panels A-D. Abbreviations as in Supplementary Figure 2.

Supplementary Figure 65: Expression of *mrp1*

In all panels, anterior is to the left. Panel A represents a dorsal view, panel B a ventral view, panel C a dorsal view, and panel D a lateral view. Arrows point to expression at the interface between the embryo proper and the yolk. The arrowheads point to expression in the tail region. A´-D´ represents SYBR green staining of the embryos shown in panels A-D. Abbreviations as in Supplementary Figure 2.

Supplementary Figure 66: Expression of *nhe2*

In all panels, anterior is to the left. Panel A represents a ventral view. Panel B is a view on the posterior of an embryo, and panel C represents a dorsal view. Arrows point to expression at the interface between the embryo proper and the yolk. The arrowheads point to expression in the tail region. A´-C´ represents SYBR green staining of the embryos shown in panels A-C. Abbreviations as in Supplementary Figure 2.

Supplementary Figure 67: Expression of *elovl4-like*

In all panels, anterior is to the left, except panel C (dissected appendages, lateral view). Arrows point to expression inside the appendages. Panels A´ and B´ represent SYBR green staining of the embryos shown in panels A and B. Abbreviations as in Supplementary Figure 2.

Supplementary Figure 68: Expression of *nrf6-l*

In all panels, anterior is to the left, except panels D and E (dissected appendages, lateral views). Arrows point to expression inside the appendages. Panels A´-C´ represent SYBR green staining of the embryos shown in panels A-C. Abbreviations as in Supplementary Figure 2.

Supplementary Figure 69: Expression of *ptgr3*

In all panels, anterior is to the left, except panel D (dissected appendages, lateral view). Arrows point to expression inside the appendages. The asterisk marks expression in the posterior tail region of the embryo. Panels A´-C´ represent SYBR green staining of the embryos shown in panels A-C. Abbreviations as in Supplementary Figure 2.
